# Multistate Azobenzene–Norbornadiene Photoswitches for Molecular Solar Thermal Energy Storage

**DOI:** 10.1002/chem.202502520

**Published:** 2025-12-08

**Authors:** Glib Arago, Karl‐Heinz Glüsenkamp, Gebhard Haberhauer

**Affiliations:** ^1^ Institut Für Organische Chemie Universität Duisburg‐Essen Essen Germany; ^2^ Squarix GmbH Marl Germany

**Keywords:** Azo compounds, molecular solar thermal systems, norbornadiene, photoswitches

## Abstract

The combination of two photochromic molecules into a multimodal photoswitch offers the potential to generate new properties related to *molecular solar thermal* (MOST) systems. A system can only be considered suitable for MOST applications if it meets key criteria, including a high quantum yield and an extended half‐life of the high‐energy isomer. Achieving this combination remains a significant challenge. In this study, we present the coupling of an azobenzene unit (AZO) with a norbornadiene system (NBD) via ester functions to develop new bi‐ and trimodal AZO‐NBD photoswitches that exhibit synergistic effects. Photochemical studies reveal that both the NBD and AZO components of the hybrid are switchable. In one case, all isomer types could be selectively produced. Furthermore, each metastable unit can be switched back independently to its corresponding thermodynamically stable form. The quantum yields and half‐lives for the NBD and AZO components of the hybrid system could be determined separately. The integration of AZOs has demonstrated the potential to extend the half‐lives of NBDs to up to 122 days in certain cases, as confirmed through comparative analysis with reference systems. The enhanced rate of back‐conversion was further facilitated by the presence of TFA as a catalyst.

## Graphical abstract

Systems consisting of two different photoswitches (azobenzene and norbornadiene) are presented, which can be switched by light, store energy, and release it again. The combination of the switching units leads to synergistic effects.

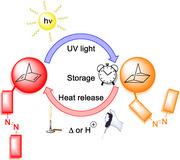

## 1 Introduction

**FIGURE 1:**
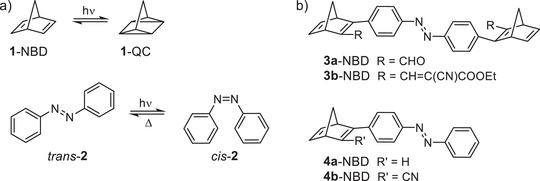
(a) Examples of photoswitchable MOST systems are the norbornadiene‐quadricyclane (NBD/QC) pair [5] (**1**) and the azobenzene (AZO) [7, 16, 17] (**2**). b) Examples of previously studied bi‐ and trimodal azobenzene‐norbornadiene hybrids [18, 19].

The global energy demand is increasing steadily, resulting in a growing need for sustainable and long‐term energy sources [1]. In this context, solar energy is increasingly being regarded as an almost inexhaustible source. The sun provides energy in the form of light and heat, which can be harnessed using various technologies. A promising strategy for storing this energy is the use of so‐called MOST (*molecular solar thermal*) systems [2, 3, 4]. These are photoswitchable molecules that are converted into a metastable, energy‐rich isomer by irradiation with sunlight [2, 3]. The energy stored in this isomer can later be released again in the form of heat in a controlled manner, for example, by using a catalyst [5]. For a photoswitch to be considered suitable for MOST, it must exhibit the following essential properties, as postulated by Yoshida et al. [6] First, there must be a significant overlap between the absorption spectrum of the low‐energy isomer and the solar spectrum. Light absorption by the high‐energy isomer, on the other hand, is undesirable. Secondly, the quantum yield for the photoisomerization reaction must be high. Thirdly, there must be a high energy difference between the metastable and the photoactive isomer, and finally, the energy density must be high. In order to facilitate a practical application that would enable seasonal energy storage, it is also necessary that the half‐life of the metastable form should be in the order of months to years. Some other key properties include fatigue resistance, which ensures that the molecular photoswitch can undergo many reversible photoisomerization cycles without significant degradation. Controlled release of the stored energy on demand is also of great importance and can be accomplished catalytically, thermally, or by light of a different wavelength. For this reason, it is crucial for the two states of the photoisomers to have a minimal spectral overlap to prevent reabsorption and back‐isomerization. Several molecular systems are currently being evaluated regarding their suitability for the application as MOST. The most prominent systems of these include azobenzene (AZO) [7, 8, 9, 10, 11, 12] and the norbornadiene‐quadricyclane systems (NBD/QC) [3, 13, 14, 15] (Figure 1a).

The suitability of various NBDs for MOST applications has already been pointed out several times in scientific literature. Multimodal NBD systems, which exhibit significant quantum yields [20], a high energy storage density [21], and remarkably long half‐lives [22], have been the subject of several reports. There are investigations regarding the combination of azobenzene with other photoswitches [23, 24, 25], however, to the best of our knowledge, only two studies have combined both systems (AZO and NBD) so far [18, 19]. In one study, two NBDs substituted with electron‐withdrawing groups (EWG) were bridged together via an azobenzene unit (Figure 1b) [18]. The authors demonstrated that both the NBD and the AZO components of the system could be switched with light. In comparison with a pure NBD system, these hybrid compounds exhibit a bathochromic shift due to the presence of the AZO unit. However, the quantum yields for the hybrids were below 3%, and thus significantly lower than the separate systems [18]. In a recent report, Wegner et al. presented a hybrid system consisting of one NBD unit connected to one or two azobenzene units (Figure 1b) [19]. However, subsequent investigations revealed that the switching behavior was limited to the AZO moiety, as the [2+2]‐cycloaddition of the NBD unit was hindered due to its direct linkage to the azobenzene. Given our extensive experience with azobenzenes in terms of switching properties [26, 27, 28] and applications [29, 30, 31], we were interested in investigating the possibility of developing bi‐ and trimodal AZO‐NBD hybrids in which both units can be effectively switched. In addition to the switchability of the system, the focus of this study was on the lifetime of the metastable states.

## 2 Results and Discussion

### 2.1 Concept and Synthesis

**FIGURE 2:**
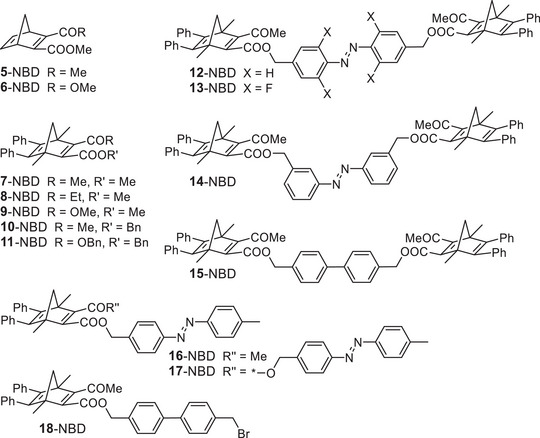
Overview of the norbornadienes **5**‐NBD to **11**‐NBD, **15**‐NBD and **18**‐NBD, as well as bi‐ and trimodal azobenzene‐norbornadiene hybrids **12**‐NBD to **14**‐NBD, **16**‐NBD, **17**‐NBD, investigated in this study.

In the 1980s, a study by Hirao et al. demonstrated that the norbornadienes **8**‐NBD and **9**‐NBD are highly promising MOST systems (Figure 2) [32]. These compounds are characterized by high thermal stability, synthetic accessibility, and a high quantum yield, in addition to a substantially prolonged half‐life relative to the corresponding QC system. However, the latter was not determined directly, but only indicated as stable. It is important to emphasize that both systems can be converted back into the NBD by acid. We therefore used these compounds as reference systems. Moreover, we utilized the derivatives **7**‐NBD, **10**‐NBD, and **11**‐NBD to determine the influence of the size of the alkyl group on the ketone and the ester. Furthermore, the smaller model compounds **5**‐NBD and **6**‐NBD were included in order to determine the extent to which donor substituents on the NBD scaffold are essential for norbornadiene stability. Designing the bi‐ and trimodal hybrid systems, the utilization of the ester functions of **7**‐NBD and **9**‐NBD as a bridging unit between the NBD subunit and the azobenzene (Figure 2) was considered. In the trimodal hybrids **12**‐NBD to **14**‐NBD, two NBD units are bridged together via an azobenzene unit. The bridging is facilitated either via the *para* positions (**12**‐NBD and **13**‐NBD) or the *meta* positions (**14**‐NBD). In the course of the study, both the non‐*ortho*‐substituted (**12**‐NBD and **14**‐NBD) and the tetrafluoro‐*ortho*‐substituted azobenzene (**13**‐NBD) were utilized. The objective here was to develop a system in which the NBD and AZO components could be switched independently of each other, with a view to investigating all possible photoisomers. The **15**‐NBD system was employed as a reference compound to ascertain the influence of the azobenzene moiety on the NBD units. Furthermore, the bimodal system **16**‐NBD and the trimodal species **17**‐NBD were prepared, in which an NBD unit is combined with one (**16**‐NBD) or two (**17**‐NBD) azobenzene components. System **18**‐NBD was selected as a reference substance, with the aim of revealing the influence of the azobenzene unit on the [2+2]‐cycloaddition of the NBD.

**SCHEME 1:**
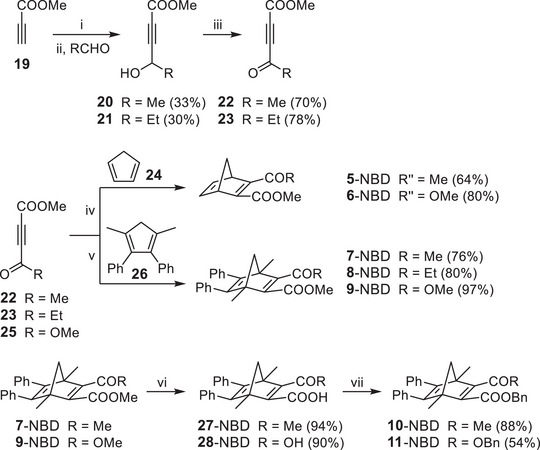
Synthesis of the norbornadienes **5**‐NBD to **11**‐NBD. Reaction conditions: i) 1‐Methylimidazole, ZnEt_2_, DCM. ii) DCM. iii) Dess‐Martin periodinane, DCM. iv) Chlorobenzene, dibutylhydroxytoluene, microwave. v) Benzene. vi) 1) NaOH_(aq.)_, MeOH; 2) HCl_(aq.)_. vii) BnBr, K_2_CO_3_, MeCN.

The synthesis of norbornadienes, from **5**‐NBD to **11**‐NBD, is shown in Scheme 1. Norbornadiene derivatives can, in principle, be accessed through various synthetic routes [5, 33]. The most significant synthetic method is the Diels‐Alder reaction between an acetylene and a cyclopentadiene derivative [3]. In many cases, this approach is not feasible. Consequently, the functionalization of norbornadiene scaffold by means of cross‐couplings is employed [19, 34, 35]. In all cases, an attempt was made to construct the norbornadienes **5**‐NBD to **11**‐NBD via a Diels‐Alder reaction of functionalized alkynes with the cyclopentadienes **24** and **26** (Scheme 1). The alkynes **22** and **23** employed for this purpose were synthesized from methyl propiolate in a two‐step process. The first step involves a C–C linkage via reaction of a zinc acetylide [36] with an aldehyde to form the secondary alcohols **20** and **21**. These can be converted into the ketones **22** and **23** using Dess‐Martin periodinane [37] (DMP). The alkyne **25** was synthesized from acetylenedicarboxylic acid by esterification. The compounds **5**‐NBD to **9**‐NBD can be prepared directly by reaction with cyclopentadiene **24** or **26** (Scheme 1). Since **24** is a weaker dienophile compared to cyclopentadiene **26**, the reaction with cyclopentadiene (**24**) had to be carried out in the microwave in chlorobenzene. At 64–80%, the yields were lower than those for the reaction with cyclopentadiene **26**, where yields of up to 97% were achieved. The model compounds **10**‐NBD and **11**‐NBD were prepared from **7**‐NBD and **9**‐NBD, respectively, by saponification of the methyl esters using a NaOH_(aq.)_/MeOH mixture and subsequent esterification. The latter was achieved via the reaction of the acids with benzyl bromide using K_2_CO_3_ as a base.

**SCHEME 2:**
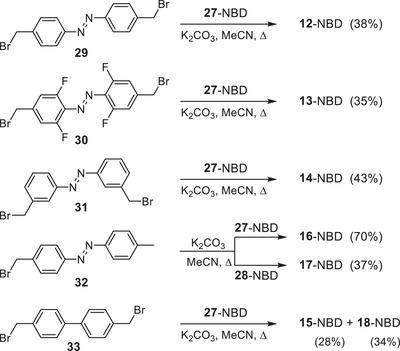
Synthesis of the norbornadienes **15** and **18**, as well as the bi‐ and trimodal azobenzene‐norbornadiene hybrids **12** to **14**, **16** and **17**.

The advantage of the proposed concept is that the desired bi‐ and trimodal systems **12**‐NBD to **17**‐NBD can be easily prepared from the carboxylic acids **27**‐NBD and **28**‐NBD by reaction with the benzyl bromides **29–33** in the presence of a base and acetonitrile as solvent under reflux (Scheme 2). It was found that K_2_CO_3_ was the most effective base. The resulting yields ranged from 34% to 88%. Attempts to prepare the esters by reacting the acids with the corresponding benzyl alcohols did not lead to any improvement in yields. As previously stated, the stability of the norbornadiene systems is of critical importance for their potential application as MOST systems. The storage of the compounds **5**‐NBD to **18**‐NBD demonstrated that the model substances **5**‐NBD and **6**‐NBD decomposed by up to 20% after a mere few weeks at room temperature. The other NBD derivatives demonstrated no decomposition, even after months of storage at room temperature. Thus, even after storage of **7**‐NBD for two years, no sign of change could be observed. This means that the introduction of donor groups on the NBD scaffold has a massive effect on the stability of the NBD unit.

### 2.2 Investigation of Photoswitching

**FIGURE 3:**
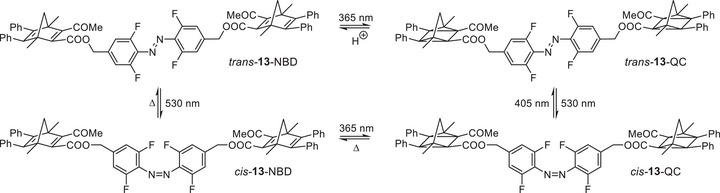
Overview of all possible photoisomers of the trimodal azobenzene‐norbornadiene hybrid **13**. All four isomers could be observed in ^1^H NMR.

**FIGURE 4:**
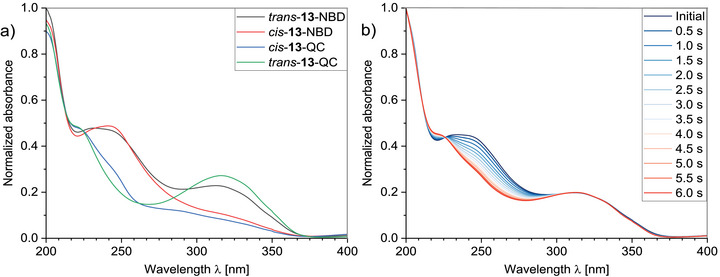
a) Normalized UV/Vis spectra of compound **13** in acetonitrile (10^−4^ m) after synthesis (*trans*‐NBD; black line). After irradiation with light of different wavelengths, the isomers *cis*‐NBD (red line), *trans*‐QC (green line), and *cis*‐QC (blue line) were observed (exact procedure see Figure 3). b) To determine the quantum yield of the NBD units, the sample was irradiated with light of the 365 nm wavelength.

The photochemical properties of the obtained basic norbornadienes (**5**‐NBD to **11**‐NBD and **18**‐NBD), as well as the bi‐ and trimodal hybrids **12**‐NBD to **17**‐NBD, were investigated by UV/Vis and ^1^H NMR spectroscopy. For the UV/Vis spectroscopy studies, acetonitrile was used as a solvent at a concentration of 50–100 µm. The ^1^H NMR spectra were recorded using chloroform (5 mm) and acetonitrile (1 mm). The samples were then exposed to light at a wavelength of 365 nm for approximately 10 s (UV spectroscopy) or 2 min (NMR spectroscopy), after which they were measured once more. The procedure was repeated until no further changes were observed in the spectra. This was usually achieved after a few repetitions (3–5). It was found that in the photostationary state (PSS), around 93–98% of the NBDs are cyclized to the QCs (see Table S1). The isomerization of the azobenzene units to the *cis* isomers was found to be around 83–91% (see Table S1, Figures S44 and S47). The quantum yields (Ф) for **5**‐NBD to **18**‐NBD were determined in acetonitrile using potassium ferrioxalate as a chemical actinometer (Tables 1 and 2) [38, 39]. The isomerization to the QCs is characterized by first‐order kinetics. The experiments were conducted using two 365 nm LED lamps from different manufacturers. The quantum yields obtained are independent of the lamps employed. While the compounds **5**‐NBD to **11**‐NBD, **15‐**NBD, and **18‐**NBD exhibited only one characteristic peak in the UV/Vis spectrum in the 210–400 nm range (Figures S6‐S12, S21 and S28), the hybrids **12–14**, **16,** and **17** each exhibited two peaks (Figures 3, 4, S13, S14, S16–S19, S22, S23, S25 and, S26). A comparison of the changes in the NMR spectra with those in the UV spectra revealed that the absorption band at 230–240 nm could, as expected, be assigned to the NBD unit, and that the band at 315–330 nm could be assigned to the azobenzene unit. Thus, in the first approximation, these can be regarded as independent of each other, which allows a separate determination of the quantum yields for both photoswitches. The validity of this approach will be shown below. However, it was not possible to investigate the photoswitching of the two NBD units within a hybrid system separately. The same applies to the AZOs in hybrid **17**, which could not be distinguished from each other. The highest quantum yields for the simple NBD systems were obtained for **7**‐NBD and **16**‐NBD, with values of 50% and 42%, respectively. Quantum yields of 30–54% were obtained for the NBD units in the bi‐ and trimodal hybrid systems. However, it should be noted that, except for **16**, the hybrid systems contain two NBD units, thereby reducing the quantum yield per NBD unit to 15–42%.

**TABLE 1:** Photochemical properties of the systems **5**‐NBD to **11**‐NBD, **15**‐NBD and **18**‐NBD.

Compound	*λ*_max_ [nm]	*λ*_onset_ [nm]	Ф (NBD)	t_1/2_ (QC) [d]
**5**‐NBD	237	398	0.03±0.01	1.14^[^ ^a^ ^]^
**6**‐NBD	232	319	<0.01±0.01	14.8^[^ ^a^ ^]^
**7**‐NBD	249	364	0.50±0.03	0.25
**8**‐NBD	243	374	0.17±0.01	1.62
**9**‐NBD	241	350	0.12±0.01	509
**10**‐NBD	244	385	0.23±0.01	1.10
**11**‐NBD	245	362	0.19±0.01	471
**15**‐NBD	255	371	0.52±0.03	2.10
**18**‐NBD	263	369	0.24±0.01	11.2

^[a]^ Measured at 100°C.

**TABLE 2:** Photochemical properties of **2**‐AZO and the hybrids **12**‐NBD to **14**‐NBD, **16**‐NBD, and **17**‐NBD.

Compound	*λ*_max_ [nm]	*λ*_onset_ [nm]	Ф (NBD)	Ф (AZO)	t_1/2_ (QC) [d]	t_1/2_ (*cis*) [h]
**2**‐AZO	277; 316	513	−	0.24±0.04	−	100
**12**‐NBD	230; 324	519	0.54±0.03	0.54±0.04	122	128
**13**‐NBD	234; 312	514	0.29±0.03	−	3.91	−
**14**‐NBD	233; 317	520	0.40±0.03	0.42±0.04	12.9	213
**16**‐NBD	231; 328	517	0.43±0.03	0.53±0.04	57.6	73
**17**‐NBD	233; 325	543	0.21±0.03	0.49±0.04	495	61

A comparison of the quantum yields per NBD unit reveals the following conclusions. Firstly, the norbornadienes **5** and **6**, which bear only two acceptor units, exhibit extremely low quantum yields of 1% and 3%, respectively. These values are comparable to those of the unsubstituted parent norbornadiene (**1**), which has a quantum yield of 5% [2]. This means that the donor substituents on the NBD scaffold massively increase the quantum yields. Secondly, linking NBDs to azobenzene units via the ester function does not lead to a significant change in quantum yields per NBD unit. A comparison of diesters **9** (12%), **11** (19%), and **17** (21%) shows that the introduction of the azobenzene unit even leads to an increase in yield.

Furthermore, the quantum yields for the AZO components of the hybrid systems were determined, taking PSS into account [40, 41]. Here, as mentioned above, it was assumed that the two absorption bands were independent of each other. However, it was not possible to determine a quantum yield for the azobenzene unit in the case of *trans*‐**13**, as it does not isomerize into the *cis* form when exposed to the light of the lamps used (365 nm). Furthermore, the quantum yield of unsubstituted azobenzene (**2**) was also determined (Table 2). The value obtained amounts to 24%, which is comparable to the data reported in literature [41] and is significantly lower than the values obtained for the combined systems (0.4–0.5). This phenomenon seems to be a synergistic effect. One possible explanation is the fact that the excited molecule can undergo three distinct photochemical processes.

A comparison of the UV/Vis spectra of hybrids **12–14**, **16,** and **17** demonstrates that the linkage of the AZO unit to the NBDs results in a bathochromic shift of the absorption end of *λ* > 510 nm compared to the NBD switches **5**‐NBD to **11**‐NBD and **18**‐NBD. In hybrid **13**, it was possible to achieve and characterize four different states by combining different stimuli. The structure of the states and the stimuli are shown in Figure 3, the corresponding UV/Vis and NMR spectra in Figures 4a and S30, S31.

Starting from *trans‐* **13**‐NBD, the NBD units can cyclize to the QCs upon irradiation with a wavelength of *λ* = 365 nm without changing the configuration of the azobenzene unit. Consequently, *trans*‐**13**‐QC is formed (Figure 3). This isomer can be converted into *cis*‐**13**‐QC by daylight (>530 nm). The latter is the most energetic isomer and can therefore be converted into *trans*‐**13**‐NBD by thermal energy, whereby *cis*‐**13**‐NBD is formed first. Compound *cis*‐**13**‐NBD can also be obtained directly from *trans*‐**13**‐NBD by irradiation with sunlight. It should be noted that irradiation of *cis*‐**13**‐NBD with light at a wavelength of *λ* = 405 nm leads to a *cis‐trans* isomerization of the AZO unit (80%) and a cyclization from NBD to QC (67%; see Figure S47). In all other model systems, cyclization of the NBDs to the QCs is not possible with this wavelength. This means that the azobenzene unit has an influence on the cyclization behavior of the NBDs.

Quantum chemical calculations using DFT were performed to evaluate energy storage (see Supporting Information). For the larger molecules, the starting geometries were obtained by conformation analysis using a force field method. The thus obtained geometric parameters were subsequently optimized by means of B3LYP [42, 43] with the empirical dispersion D3BJ [44]. As a basis set def2‐SVP [45, 46] was employed. The energy of the molecules was computed using B3LYP‐D3BJ and the basis set def2‐TZVP [45]. To take solvent effects into account, the solvent model SMD [47] (CHCl_3_ as solvent) was applied for the single‐point calculations. The B3LYP functional was chosen because, within the single‐reference methods, it provides values that agree well with experimental data.[48] Since the difference between enthalpy and electronic energy is quite small [48], only the storage energy (Δ*E*_storage_) of the systems was considered (Table 3). The latter corresponds to the energy difference between the initial system, in which only NBD and *trans*‐Azo units are present, and the product of the photoisomerization process from NBD to QC and from *trans*‐AZO to *cis*‐AZO. A comparison of the calculated data reveals that the highest values are obtained with 204–245 kJ/mol in the multimodal systems **12–14**. However, the calculated energy storage density [MJ/kg] is lowest at 0.21 MJ/kg for **13** due to its high molecular weight. The highest energy density is found for **14**, at 0.27 MJ/kg, which is larger than that of azobenzene (0.20 MJ/kg) and substituted azobenzenes (0.11 to 0.17 MJ/kg) [3], but significantly lower than that of doubly substituted NBD derivatives, which lies at approx. 0.4 MJ/kg [3]. Further optimization may be possible in the future by varying the substituents.

**TABLE 3:** Molecular weight (MW) and calculated storage energy (Δ*E*_storage_) after photoisomerization process from NBD to QC and from *trans*‐AZO to *cis*‐AZO.

Compound	MW [g/mol]	Δ*E*_storage_ [kJ/mol]	Δ*E*_storage_ [MJ/kg]
**7**	372.46	93.6	0.251
**9**	388.46	95.2	0.245
**12**	923.12	224.3	0.243
**13**	995.08	203.7	0.205
**14**	923.12	244.9	0.265
**16**	566.70	131.4	0.232
**17**	776.94	191.9	0.247

### 2.3 Kinetic Studies and Thermal Back‐Conversion

The kinetics of the back‐reaction of the systems **5**‐QC to **18**‐QC into the thermally more stable isomers **5**‐NBD to **18**‐NBD were investigated using ^1^H NMR measurements in CDCl_3_ at concentrations of 5 mm. In order to enable a more precise extrapolation of the values, the back‐conversion in some systems was monitored over a period of more than 100 days. The measurements were conducted at ambient temperature for all systems, except for **5** and **6**. The latter were measured at 100°C, as this was the only method of achieving back‐isomerization. It is apparent that, due to the separate signals in the ^1^H NMR, the back‐conversion of the azobenzene unit could be determined separately from the QC units in the hybrid systems. Please note that asymmetrically substituted NBDs such as **5**, **7**, **8**, and **10** are chiral and therefore exist as racemates. The multimodal systems, which exhibit two NBD units (**12–15**), therefore exist as diastereomers, which requires more complex NMR analysis. In the trimodal hybrids with two QC units, it was not possible to distinguish between the two. However, it is noteworthy that when the trimodal hybrid *cis,cis*‐**17**‐QC was isomerized back, not only the *trans,trans*‐**17**‐QC but also the mixed *trans,cis*‐**17**‐QC isomer could be observed in the NMR. It was therefore practically possible to distinguish between the two AZO units, which was not possible with two NBDs in the hybrid systems. In all cases, first‐order kinetics were assumed.

The data on the half‐lives are listed in Tables 1 and 2. The **5**‐QC and **6**‐QC systems have half‐lives of 1–15 days at 100°C. Extrapolation of these results to room temperature would result in half‐lives of more than 100 years. These were thus similar to the unsubstituted **1**‐QC, whose half‐life was extrapolated to ∼10,000 years at room temperature [16]. The measured half‐life of **1**‐QC was found to be 14 h at 140°C.[2] Therefore, from the standpoint of half‐lives, systems **5** and **6** would be considered optimal. However, the quantum yields are found to be very low, and of greater concern is the fact that the underlying norbornadienes undergo decomposition after a mere few weeks at room temperature.

An analysis of the half‐lives of the model compounds **7**‐QC to **11**‐QC, **15**‐QC, and **18**‐QC (Table 2) reveals a consistent trend: the diesters **9**‐QC and **11**‐QC exhibit half‐lives that, in some cases, exceed those of the corresponding keto esters (**7**, **8**, **10**, **15**, and **18**) by more than two orders of magnitude. This finding contrasts with earlier assumptions suggesting similar behavior of **8**‐QC and **9**‐QC, even though their half‐lives were not explicitly determined in those studies [49]. Within the hybrid systems, **17**‐QC, in which two ester units are bound to the QC scaffold, demonstrates a comparable half‐life for the QC unit (495 days) to that observed for the diesters **9**‐QC and **11**‐QC. This finding indicates that the azobenzene units do not affect the rate of back‐isomerization in these cases. A comparison of the half‐life values of the hybrids that have both an ester and a keto group bound to QC (**12**‐QC to **14**‐QC and **16**‐QC) is of high interest. The hybrids **13**‐QC and **14**‐QC have essentially similar stabilities to the reference compounds **15**‐QC and **18**‐QC. In contrast, the hybrid systems **12**‐QC and **16**‐QC demonstrate an almost 10‐ to 100‐fold increase compared to the reference compounds **7**, **15,** and **18**. The back‐conversion of **12**‐QC had to be monitored over a period of approx. 160 days in order to detect a change. This means that, unexpectedly, the linkage of an NBD ester with an AZO unit represents a possibility for a massive increase in the half‐lives of the QCs.

The half‐lives of the AZO components of the hybrid systems were found to range from 3 to 9 days at room temperature, which is typical for the simple AZOs. For example, a half‐life of 4 days was found for the unsubstituted AZO (**2**) [7,50]. Notably, *cis*‐**13** deviated from this trend, as its half‐life could not be reliably determined. This phenomenon can be attributed to two primary factors: Firstly, tetrafluoro‐substituted azobenzenes have very long half‐lives (e.g., up to 2 years at room temperature) [40]. Furthermore, as previously stated, the AZO unit in **13** switches from *trans* to *cis* even in daylight, so that despite the use of amber glass tubes, potential absorption of daylight cannot be ruled out. With the exception of **14** (half‐life of 9 or 12 days), the half‐lives of the AZO units are significantly shorter than those of the QC units. This facilitates the characterization of metastable *trans*‐QC isomers in the spectra.

**FIGURE 5:**
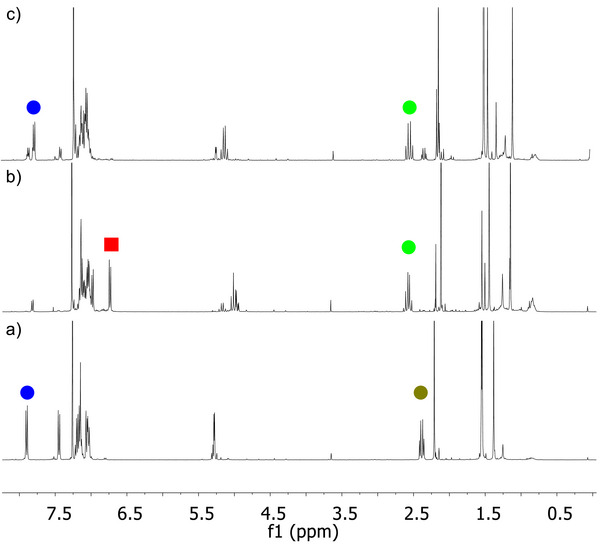
^1^H NMR spectra of compound **12** in CDCl_3_ (400 MHz, 5 mm): a) before irradiation; b) after irradiation with light of wavelength *λ* = 365 nm for 2 min; c) 50 days after irradiation. The *trans*‐**12**‐QC isomer was obtained due to a high difference in the half‐lives between the azo and the NBD. (blue: *trans*‐AZO; red: *cis*‐AZO; dark green: NBD; light green: QC).

**FIGURE 6:**
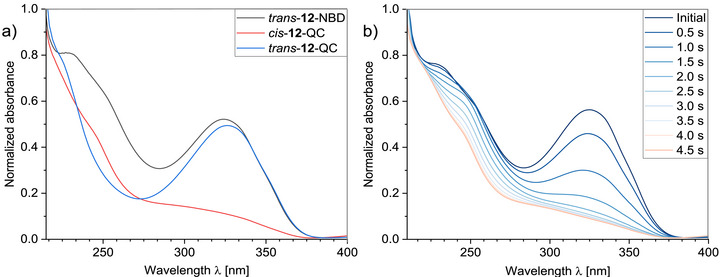
a) Normalized UV/Vis spectra of compound **12** in acetonitrile (10^−4^ m) after synthesis (*trans*‐NBD; black line). After irradiation with light of different wavelengths, the *cis*‐QC (365 nm; red line) and *trans*‐QC (405 nm; blue line) isomers were observed. b) Normalized UV/Vis spectra of compound **12** in acetonitrile (10^−4^ m). To determine the quantum yield of the NBD, the sample was irradiated with light at 365 nm.

For instance, the ^1^H NMR spectra of the various isomers of **12** are shown in Figure 5. The starting point is *trans*‐**12**‐NBD, which can be isolated after synthesis. By irradiation with 365 nm, *cis*‐**12**‐QC is formed to approx. 83%, the remaining 17% represents *trans*‐**12**‐QC. This can be recognized by the shift of the signals at originally 2.39 ppm for the NBD and at 7.90 ppm for the *trans* isomer. The typical signals for the *cis* isomer appear at 6.73 ppm. The methylene group of the QC scaffold is found at 2.56 ppm. Following a 50‐day period of room temperature storage and exclusion of light, the characteristic signals for the *cis*‐AZO unit are no longer evident at 6.73 ppm. Subsequently, the presence of *trans*‐**12**‐QC, accompanied by a negligible quantity of *trans*‐**12**‐NBD, is observed. The corresponding UV/Vis spectra are shown in Figure 6a. The absorption band of the NBD unit at 230 nm and the π–π* band of the *trans‐*azobenzene at 324 nm can be clearly seen. Furthermore, it can be concluded that both are independent of each other to a first approximation. The two isomers, *cis*‐**12**‐QC and *trans*‐**12**‐QC, have an isosbestic point at 234 nm. In other words, measuring at this point, starting from *trans*‐**12**‐NBD, enables the selective determination of the transition from NBD to QC. Substituting the value of 230 nm with that of 234 nm has only a negligible effect on the quantum yield of the NBD units, reducing it from 54% to 53%. The same applies to the determination of the quantum yield for the azobenzene unit. If a value of 336 nm is used instead of the maximum at 324 nm, at which *trans*‐**12**‐NBD and *trans*‐**12**‐QC have identical values (Figure 6a), the yield only increases by 1% to 55%. In view of this slight change, the assumption of selective determination of the quantum yields of NBD and azobenzene at different wavelengths is legitimate.

In addition, the back‐conversion barriers for both units (QC to NBD and *cis*‐AZO to *trans*‐AZO) were determined for *cis*‐**12**‐QC and *cis*‐**16**‐QC by the analysis of the kinetics of the back‐reaction. Therefore, we measured the half‐lives of the AZO and QC units separately at different temperatures (25°C, 40°C, and 50°C) by means of ^1^H NMR spectroscopy (Table 4) and used the thus obtained data for an Arrhenius plot (Table 4 and Figures S34 and S40). As expected on the basis of the half‐lives, the activation barriers for the reverse reaction of the QC unit to NBD are significantly higher than those for the isomerization of azobenzene (Table 4). Compared to disubstituted NBD derivatives, which typically range between 100 and 110 kJ/mol [48], compound **12** is one of the so‐called high performers [51]. In addition, quantum chemical calculations were performed for the back‐isomerization of *cis*‐**16**‐QC. The activation energy was computed in analogy to the calculation of the energy density using B3LYP and D3BJ. The only difference in this case was that the open‐shell variant of DFT was applied for the transition state. This is necessary to adequately describe the diradical character of the transition states. This method has already been used to accurately describe the re‐isomerization barriers of a number of substituted QC systems [51] and other diradical transition states [52, 53]. The activation energies calculated by this approach amount to 92 kJ/mol for the QC and 85 kJ/mol for the AZO unit. The value for the QC system in particular is significantly lower than that obtained experimentally (114 kJ/mol). This underestimation of the activation barrier using UB3LYP has already been demonstrated for other QC systems with long half‐lives [51].

**TABLE 4:** Back‐conversion barrier of the hybrids **12** and **16**.

Compound	*E*a (QC) [kJ/mol]	*E*a (*cis‐*AZO) [kJ/mol]
**12**	131 ± 28	107 ± 18
**16**	114 ± 9	93 ± 5

### 2.4 Energy Release Investigations

**FIGURE 7:**
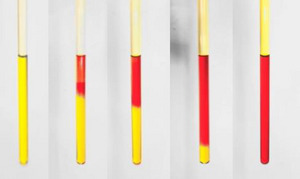
NMR sample of the irradiated molecule **17** after adding a drop of TFA to the solution: the yellow *cis*‐**17**‐QC changes over time (a few minutes) into the red *trans*‐**17**‐NBD.

The targeted back‐isomerization can be performed using an acid, such as trifluoroacetic acid (TFA), as a catalyst. ^1^H NMR studies demonstrate that an excess of acid facilitates the process of back‐conversion, within a few minutes. In hybrid systems **12–17**, QC‐NBD and *cis*/*trans* back‐isomerization occur. In the case of **17**, this phenomenon can also be observed with the naked eye (Figure 7), whereby the yellow *cis*‐**17**‐QC is converted into the red *trans*‐**17**‐NBD by a drop of TFA. No significant differences between systems were observed in the acid‐catalyzed back‐reaction studies.

Aside from the usage of TFA as a catalyst for back‐isomerization, its corresponding silver salt was also employed. The compounds **9**, **12**, and **16** were subjected to silver trifluoroacetate testing and characterized using ^1^H NMR spectroscopy. It was found that the use of silver trifluoroacetate allows the QCs to be quantitatively reversed back to NBDs. However, no *cis*‐*trans* back‐isomerization of the AZO units was observed in the cases of *cis*‐**12**‐QC and *cis*‐**16**‐QC. Please note that this allows selective back‐switching of the *cis*‐AZO unit by heat and the QC moiety using silver trifluoroacetate, making the systems powerful double switches.

## 3 Conclusion

In the course of the present study, a number of AZO‐NBD systems were examined, in which the NBDs exhibited a donor‐acceptor substitution pattern. This facilitated a bathochromic shift in the absorption end of the system, as well as enhancing the stability of the QCs within the system. It could be shown that both the NBD component and the AZO component were photoswitchable, and that the main photochemical parameters for each component could be determined separately. For the hybrid system **13**‐NBD, it was possible to selectively isomerize the two components, thereby enabling the characterization of all four possible isomers. The combination of AZO units with NBDs leads to synergistic effects in terms of extending the half‐lives of the QC and increasing the quantum yield of the AZO unit. For example, a significant increase in the half‐life of the hybrid **12**‐QC was observed, from 1–2 days to 122 days, as evidenced by a comparison with reference systems. It can therefore be concluded that the hybrid systems of the AZO‐NBD type can be considered as double switches and potential candidates for MOST systems due to their high half‐lives and overall quantum yields.

## Conflicts of Interest

Authors declare that they have no competing interests.

## Supporting information




**Supporting file 1**: chem70541‐sup‐0001‐SuppMat.pdf

## Data Availability

The data that support the findings of this study are available in the supplementary material of this article.
