# Supplementary material for: Multistate Azobenzene–Norbornadiene Photoswitches for Molecular Solar Thermal Energy Storage
Source: Chemistry. 2025 Dec 8;32(2):e02520. doi: 10.1002/chem.202502520 (PMC12790303; doi:10.1002/chem.202502520)
Supplement: Supplementary file 1 — Supporting file 1: chem70541‐sup‐0001‐SuppMat.pdf [file CHEM-32-e02520-s001.pdf]

Supporting Information

Multistate Azobenzene–Norbornadiene Photoswitches for Molecular Solar Thermal Energy Storage

Glib Arago[†], Karl-Heinz Glüsenkamp[‡] and Gebhard Haberhauer*[†]

[†] Institut für Organische Chemie, Universität Duisburg-Essen, Universitätsstr. 7, D-45117 Essen, Germany

[‡] Squarix GmbH, Elbestrasse 10, 45768 Marl, Germany

1. **Chemical Actinometry** S2
2. **UV/Vis Absorbance and Quantum Yield** S5
3. **NMR Experiments and Half-Life of the Compounds** S18
4. **NMR Spectra of Isomers** S26
5. **Synthesis of New Compounds** S31
6. **Computational Details** S46
7. **^{1}H NMR and ^{13}C NMR Spectra of the New Compounds** S73
8. **Supporting Information References** S94

1. Chemical Actinometry

Quantum yields were determined using actionometry with UV/Vis spectroscopy according to the method that has been previously described in the literature.[1] All work with potassium ferrioxalate and its solutions was conducted under red light conditions (λ > 580 nm) while all other light sources were extinguished, and the compound was shielded as much as possible with aluminum foil. LED lamps were utilized to irradiate the samples with light of wavelength λ = 365 nm. Both a 3×Nichia (NC4U133A) lamp with 3300 mW output and a 4×Hönle lamp with 1450 mW output were employed.

Photon flux measurement. Two separate solutions were prepared to determine the photon flux. One solution was prepared by dissolving potassium ferrioxalate trihydrate (98.25 mg, 0.20 mmol) in 20 mL of the 0.05 M sulfuric acid in a volumetric flask and stored in the dark. The other buffer-solution contained anhydrous sodium acetate (13.65 g, 165.0 mmol) and 1,10-phenanthroline monohydrate (1.10 g, 5.5 mmol), which were dissolved in 100 mL of 0.5 M sulfuric acid.
Two milliliters of the potassium ferrioxalate solution were irradiated with light of wavelength λ = 365 nm in a cuvette (path length: 1 cm) at a 1 cm distance in the dark. After irradiation, 1 mL of the solution was mixed with 10 mL of the buffer solution and left to sit for 30 minutes. A 2-mL sample was taken from the resulting solution, transferred to a cuvette and the absorbance was measured using UV/Vis spectroscopy (Figure S1). The procedure was performed with a separate sample for every desired irradiation time, including one blank sample that was not irradiated. Absorbance was determined at 510 nm, and the amount of Fe^{2+} ions formed was calculated according to the equation (1) (Figures S2 and S3).

$$n\,(Fe^{2+}) = \frac{\Delta A_{510\ nm} * V_1 * V_3}{\varepsilon_{510\ nm} * l * V_2} \qquad (1)$$

$\Delta A_{510\ nm}$ = difference in absorbance at 510 nm between blank and sample
l = cuvette path length (1 cm)
$\varepsilon_{510\ nm}$ = molar absorption coefficient of $Fe(phen)_3^{2+}$ complex at 510 nm (11.100 $M^{-1}\ cm^{-1}$)[2]
V_1 = total volume of irradiated sample (2 mL)
V_2 = volume of sample transferred to developer solution (1 mL)
V_3 = total volume of developer solution and transferred sample (11 mL)
The photon flux $q_{n,p}$ [Einstein/s] is given by equation (2):[2]

$$q_{n,p} = \frac{\Delta A_{510\ nm} * V_1 * V_3}{\Phi(\lambda) * \varepsilon_{510\ nm} * l * V_2 * t} \quad (2)$$

$\Phi(\lambda)$ = photoreaction quantum yield at specified wavelength

Given the known amount of Fe^{2+} complex, the photon flux can be determined using the following equation (3):

$$q_{n,p} = \frac{n\,(Fe^{2+})}{\Phi(\lambda) * t} \quad (3)$$

The data points obtained for the amount of Fe^{2+} complex were plotted against the irradiation time, and the slope was determined (Figures S2 and S3). Given the established quantum yield for potassium ferrioxalate at c = 0.01 M of 1.27 mol/Einstein, the photon fluxes for the two lamps can be calculated.[3] The photon flux was determined to be $q_{n,p}$ = 1.51×10^{-7} Einstein/s (for 4×Hönle lamp) and $q_{n,p}$ = 3.31×10^{-7} Einstein/s (for 3×Nichia NC4U133A lamp).

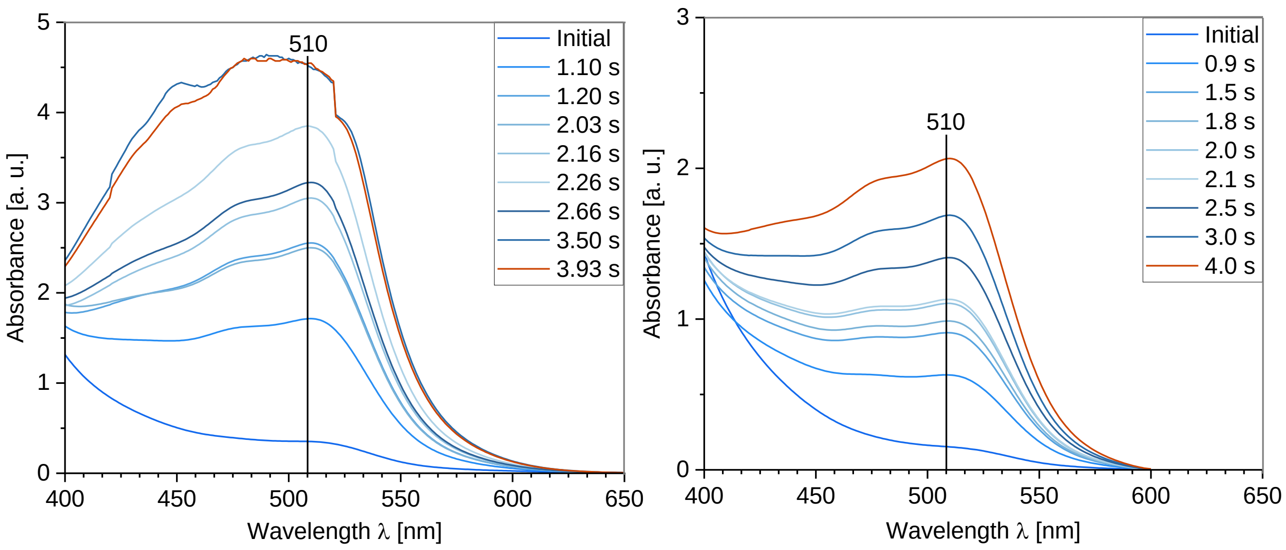

Figure S1. UV/Vis absorbance spectra of the Fe^{2+} complex after different time intervals of irradiation with 3×Nichia lamp (left) and 4×Hönle lamp (right).

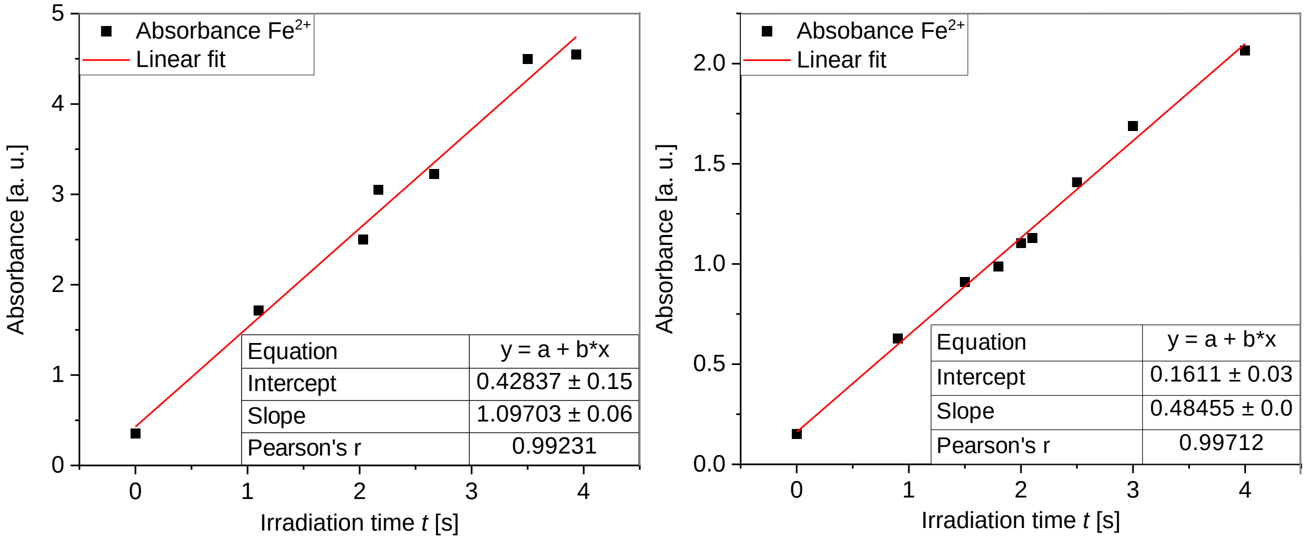

Figure S2. Absorbance at 510 nm plotted against the irradiation time of the potassium ferrioxalate solution (0.01 M) and linear fit parameters using the 3×Nichia NC4U133A lamp (left) and the 4×Hönle lamp (right).

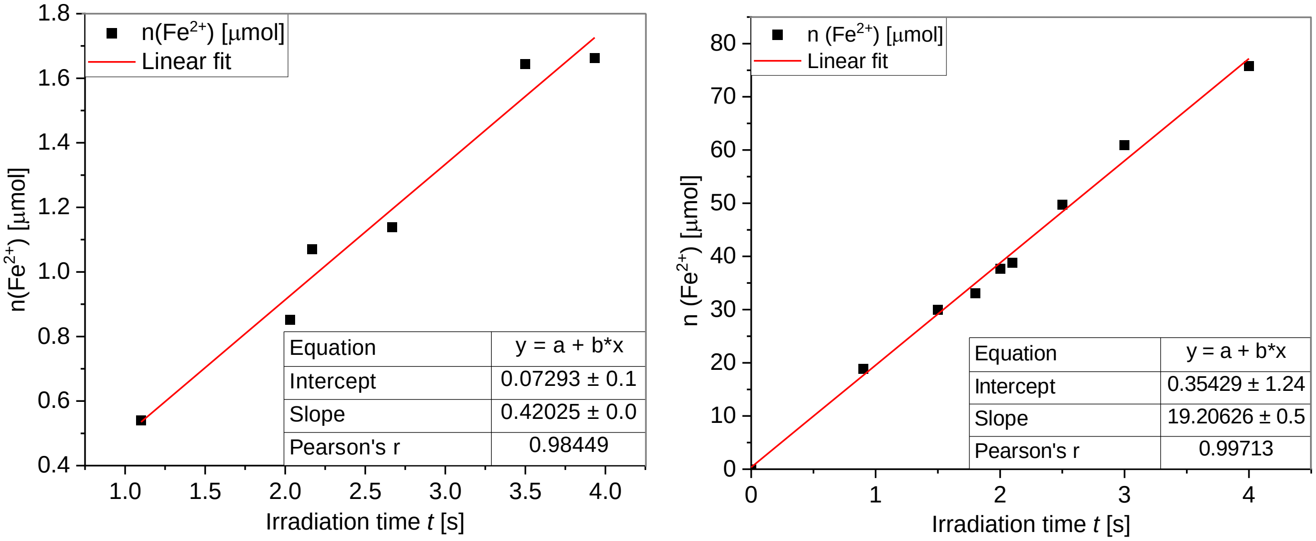

Figure S3. Amount of Fe^{2+} (calculated via equation (1)) plotted against the irradiation time and linear fit parameters using the 3×Nichia NC4U133A lamp (left) and the 4×Hönle lamp (right).

2. UV/Vis Absorbance and Quantum Yield

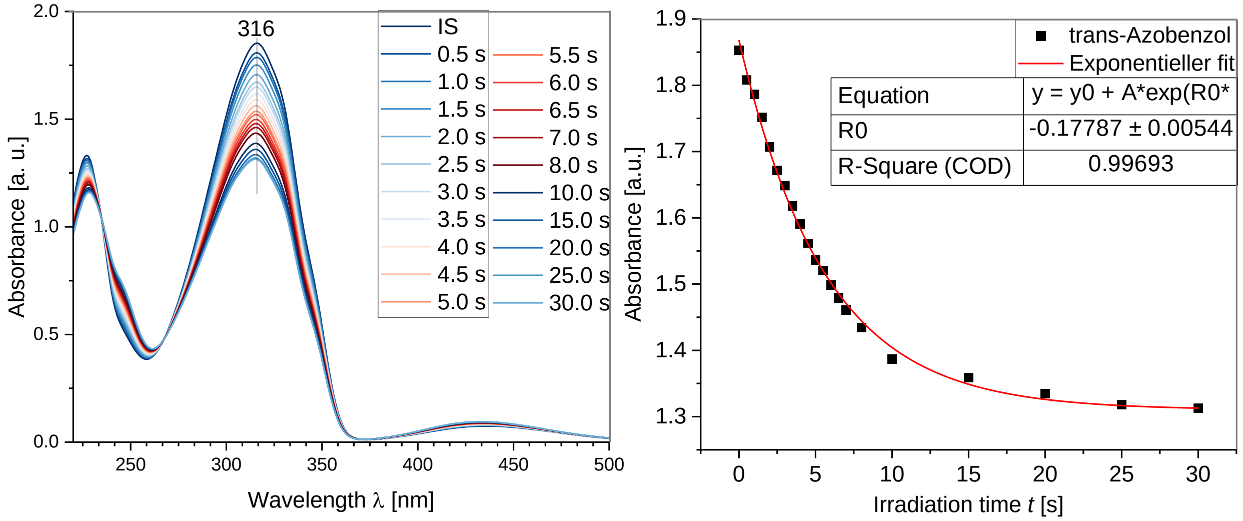

Figure S4. UV/Vis absorbance spectra of the *trans*-**2** (100 µM in CH_3CN; left) and absorbance at 316 nm after different time intervals of irradiation (right) (light source: 4×Hönle lamp).

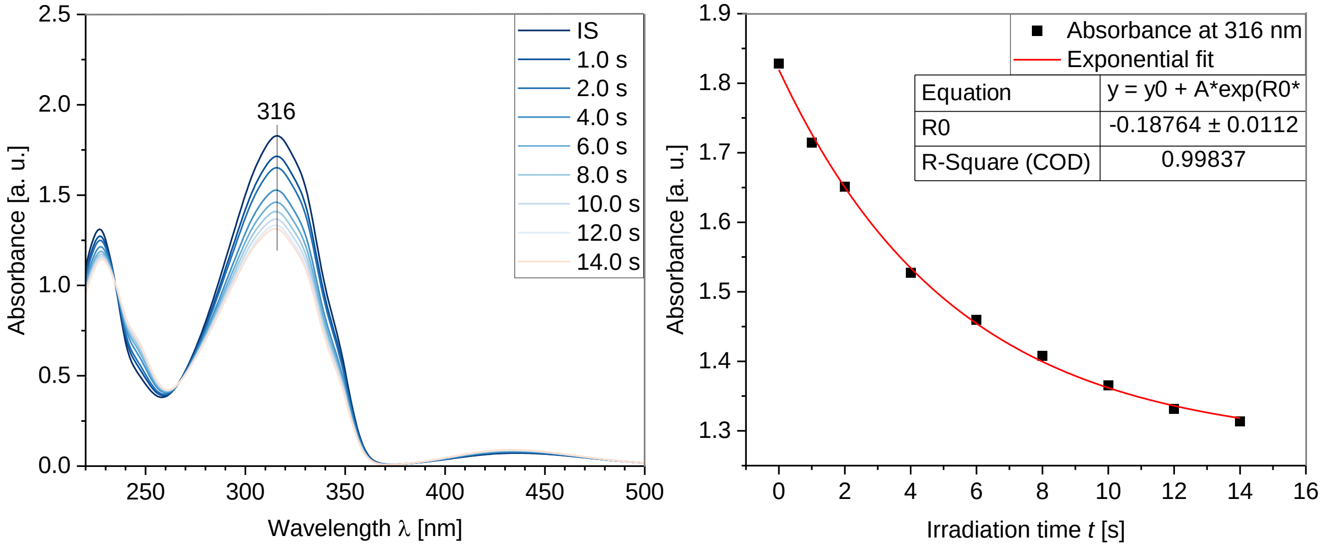

Figure S5. UV/Vis absorbance spectra of the *trans*-**2** (100 µM in CH_3CN; left) and absorbance at 316 nm after different time intervals of irradiation (right) (light source: 4×Hönle lamp).

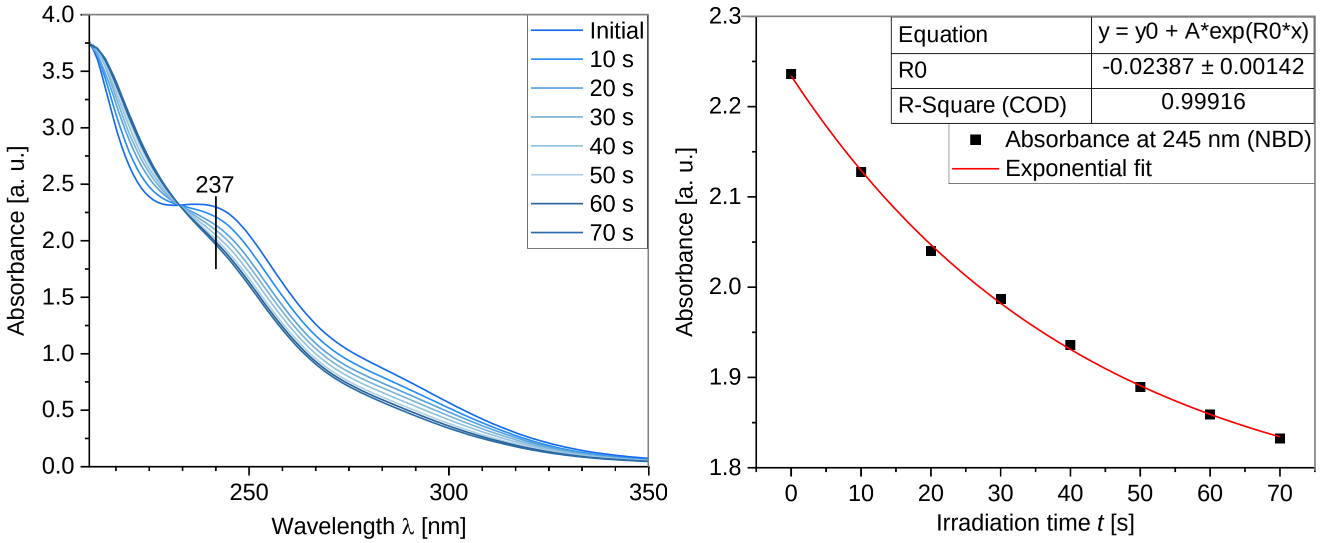

Figure S6. UV/Vis absorbance spectra of the **5**-NBD (100 µM in CH_3CN; left) and absorbance at 237 nm after different time intervals of irradiation (right) (light source: 4×Hönle lamp).

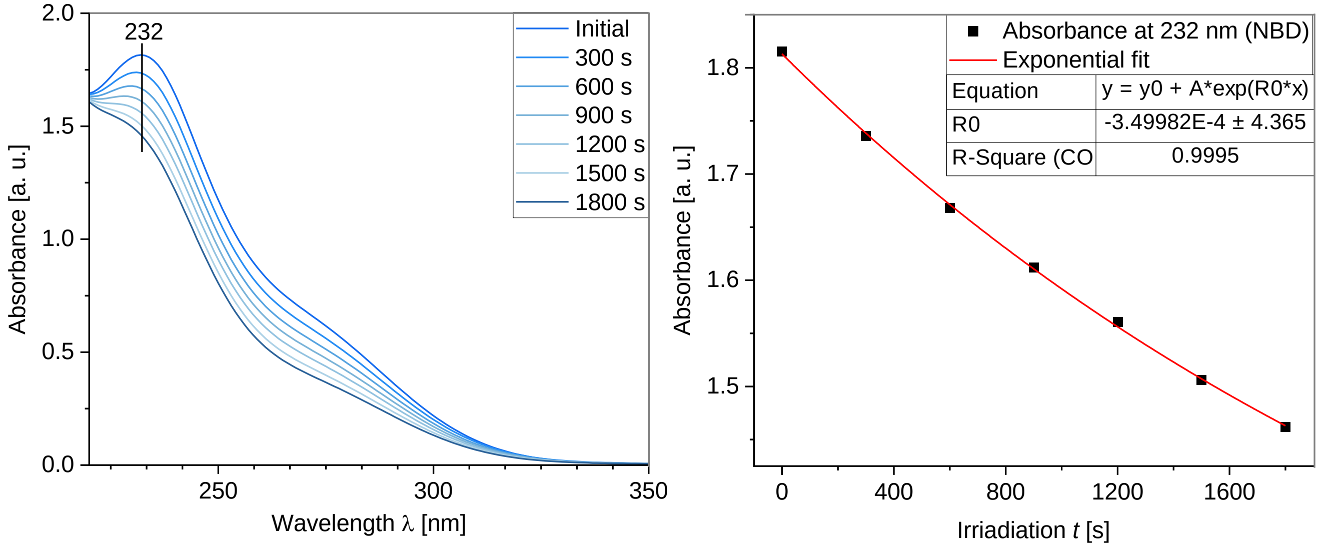

Figure S7. UV/Vis absorbance spectra of the **6**-NBD (100 µM in CH_3CN; left) and absorbance at 232 nm after different time intervals of irradiation (right) (light source: 4×Hönle lamp).

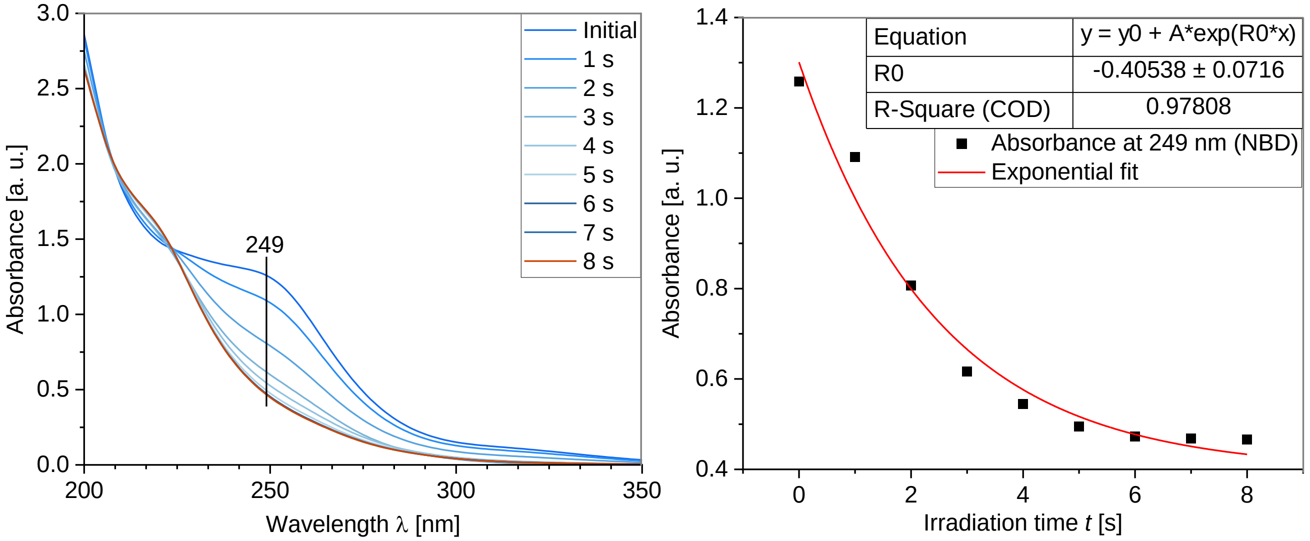

Figure S8. UV/Vis absorbance spectra of the **7**-NBD (100 µM in CH_3CN; left) and absorbance at 249 nm after different time intervals of irradiation (right) (light source: 4×Hönle lamp).

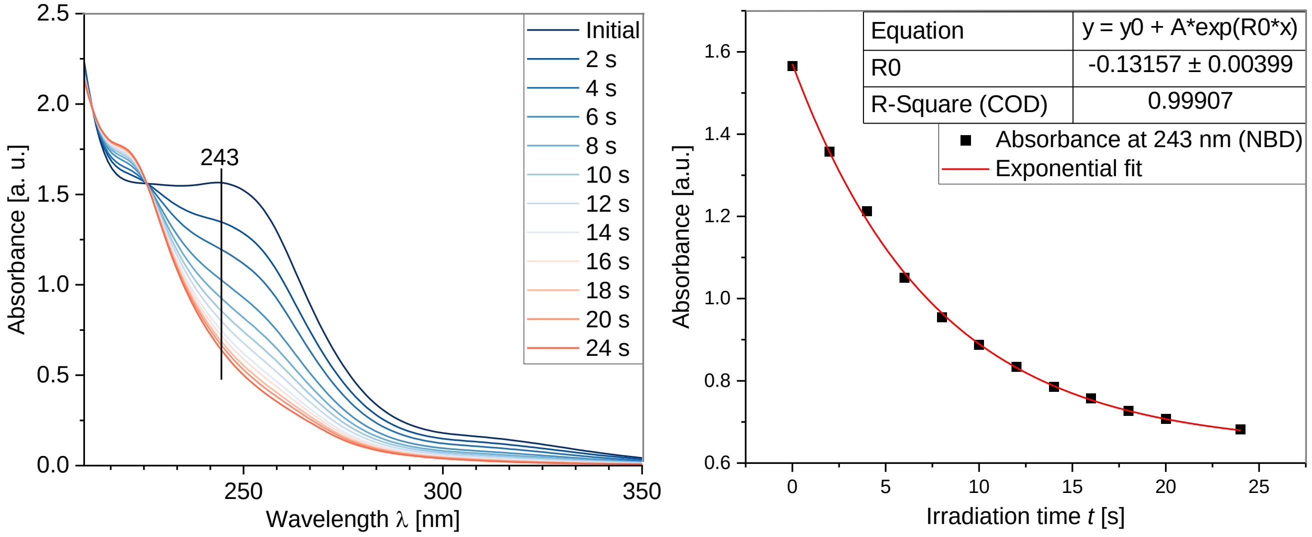

Figure S9. UV/Vis absorbance spectra of the **8**-NBD (100 µM in CH_3CN; left) and absorbance at 243 nm after different time intervals of irradiation (right) (light source: 4×Hönle lamp).

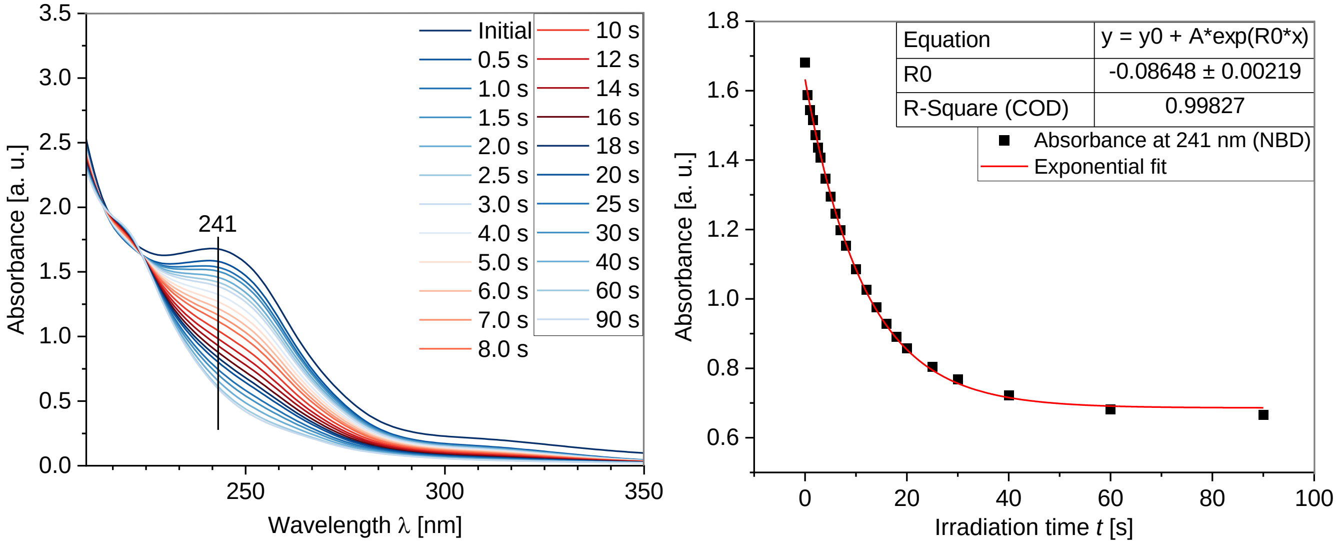

Figure S10. UV/Vis absorbance spectra of the **9**-NBD (100 µM in CH_3CN; left) and absorbance at 241 nm after different time intervals of irradiation (right) (light source: 4×Hönle lamp).

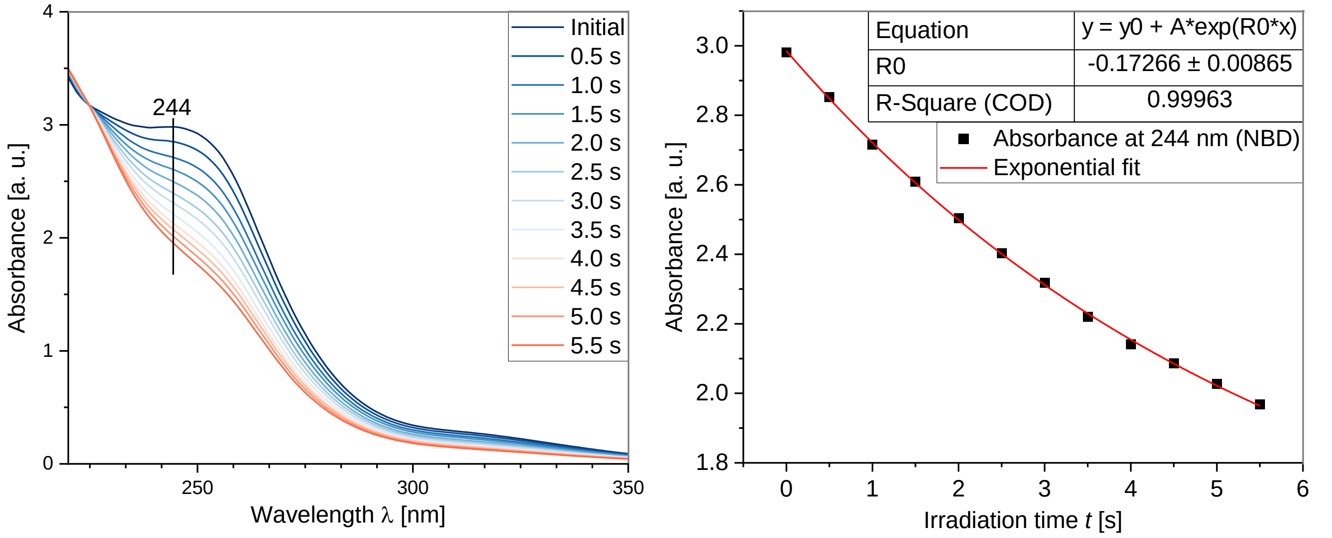

Figure S11. UV/Vis absorbance spectra of the **10**-NBD (100 µM in CH_3CN; left) and absorbance at 244 nm after different time intervals of irradiation (right) (light source: 4×Hönle lamp).

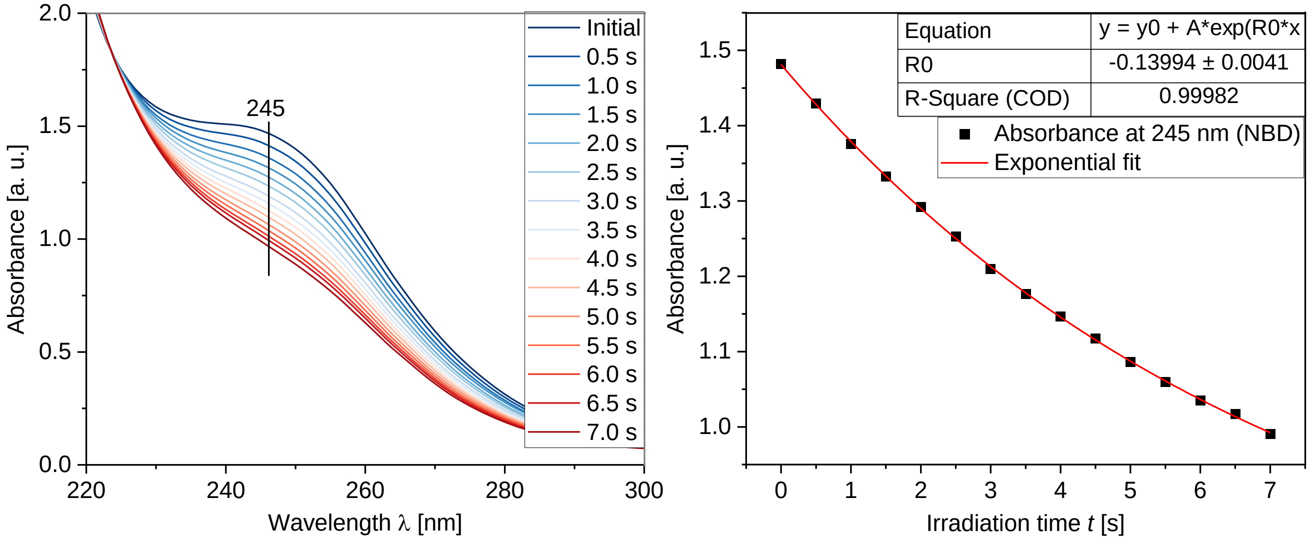

Figure S12. UV/Vis absorbance spectra of the **11**-NBD (100 µM in CH_3CN; left) and absorbance at 245 nm after different time intervals of irradiation (right) (light source: 4×Hönle lamp).

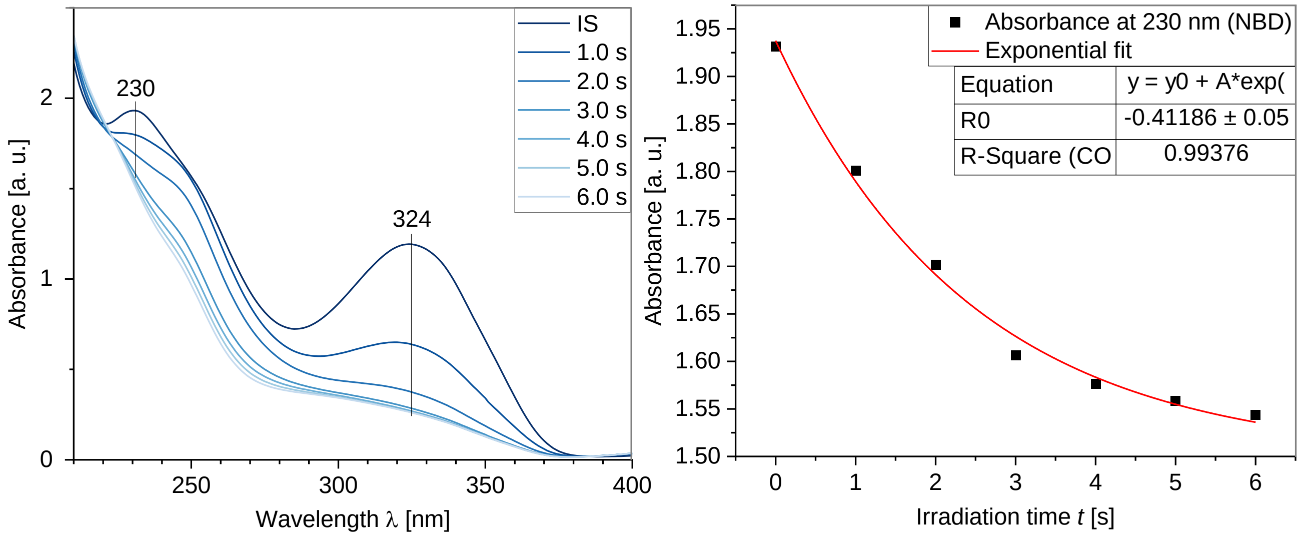

Figure S13. UV/Vis absorbance spectra of the **12**-NBD (50 µM in CH_3CN; left) and absorbance at 230 nm after different time intervals of irradiation (right) (light source: 4×Hönle lamp).

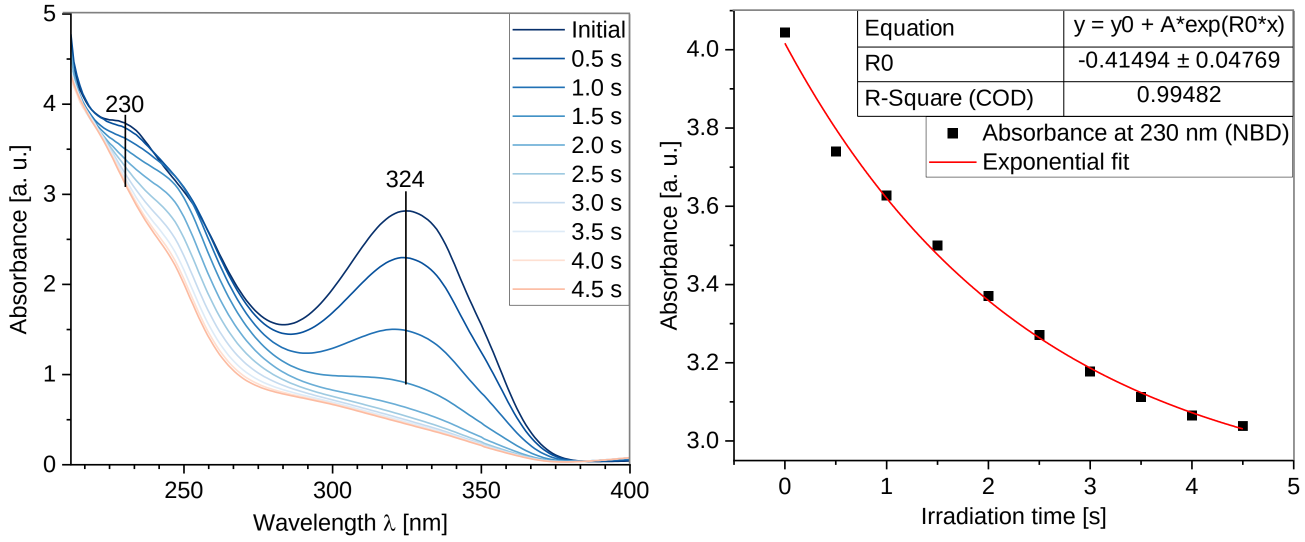

Figure S14. UV/Vis absorbance spectra of the **12**-NBD (100 µM in CH_3CN; left) and absorbance at 230 nm after different time intervals of irradiation (right) (light source: 4×Hönle lamp).

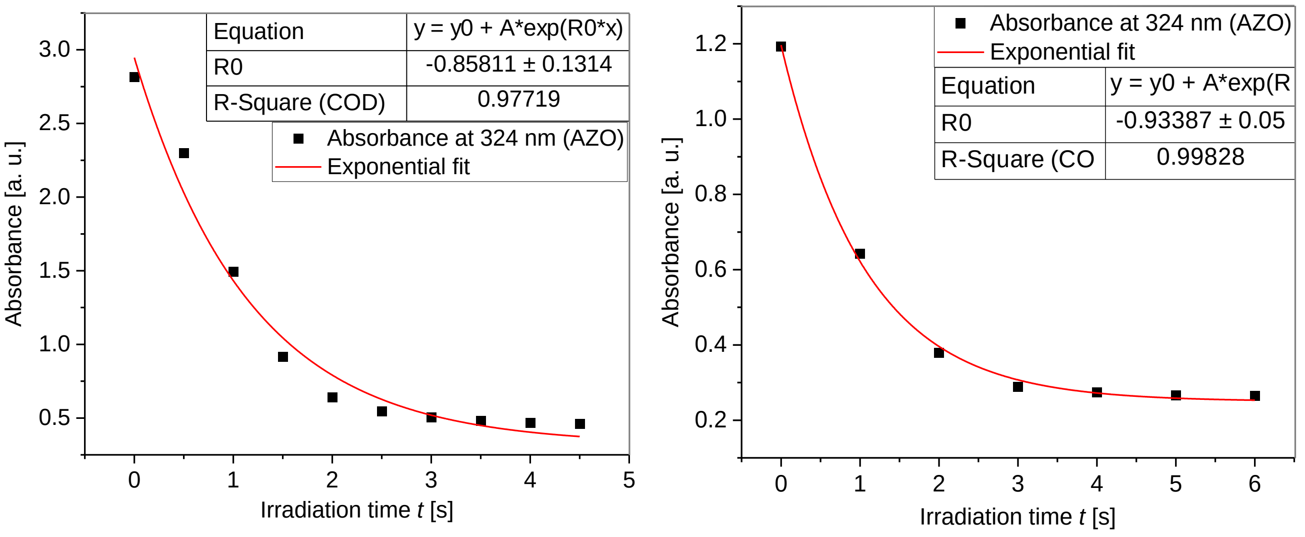

Figure S15. Absorbance at 324 nm of *trans*-**12** in (100 in CH_3CN µM; left) and (50 µM in CH_3CN; right) after different time intervals of irradiation (light source: 4×Hönle lamp).

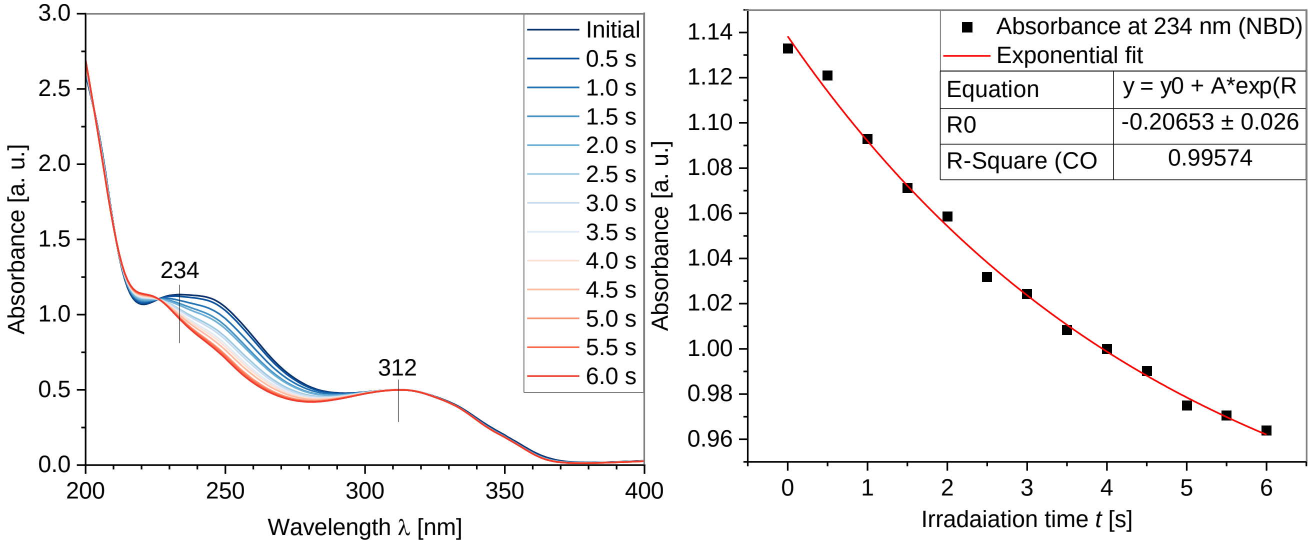

Figure S16. UV/Vis absorbance spectra of the **13**-NBD (100 µM in CH_3CN; left) and absorbance at 234 nm after different time intervals of irradiation (right) (light source: 4×Hönle lamp).

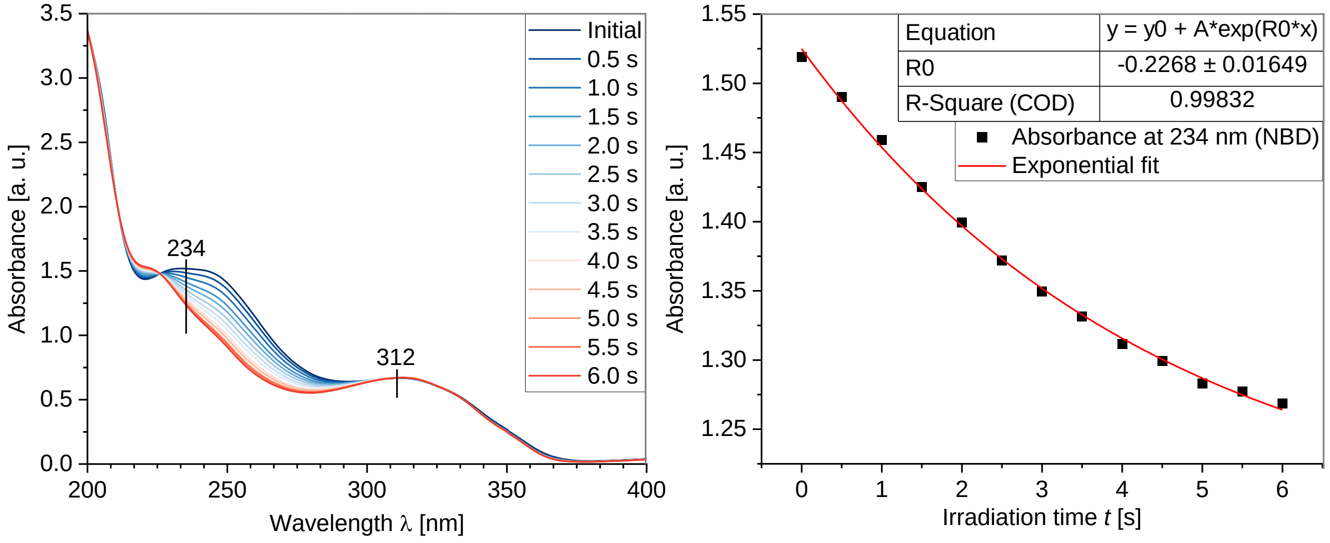

Figure S17. UV/Vis absorbance spectra of the **13**-NBD (100 µM in CH_3CN; left) and absorbance at 234 nm after different time intervals of irradiation (right) (light source: 4×Hönle lamp).

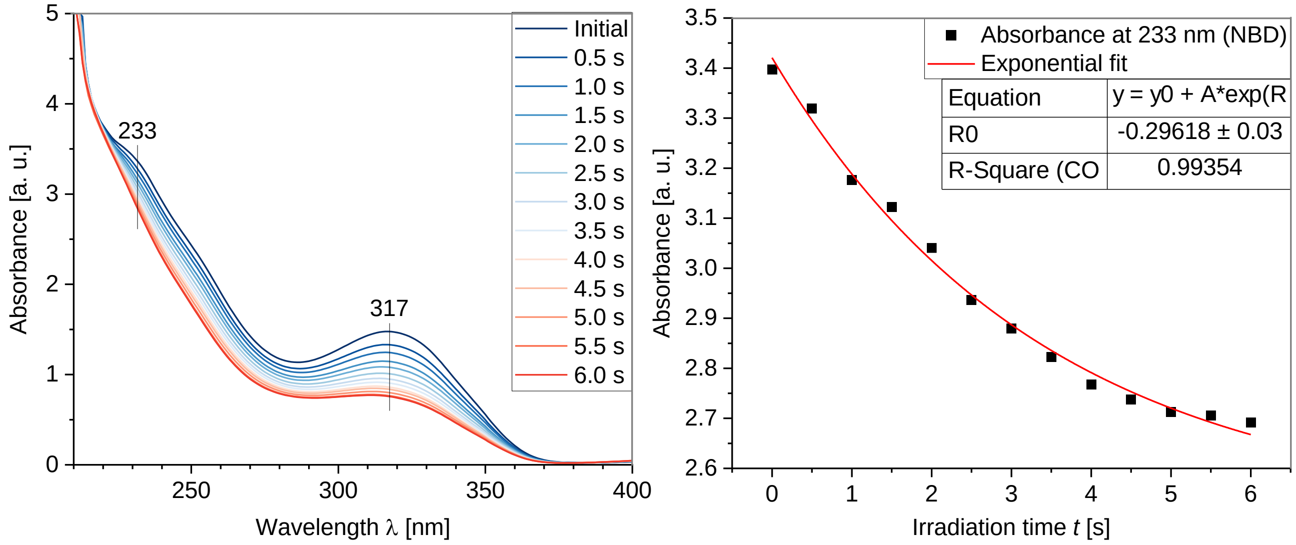

Figure S18. UV/Vis absorbance spectra of the **14**-NBD (100 µM in CH_3CN; left) and absorbance at 233 nm after different time intervals of irradiation (right) (light source: 4×Hönle lamp).

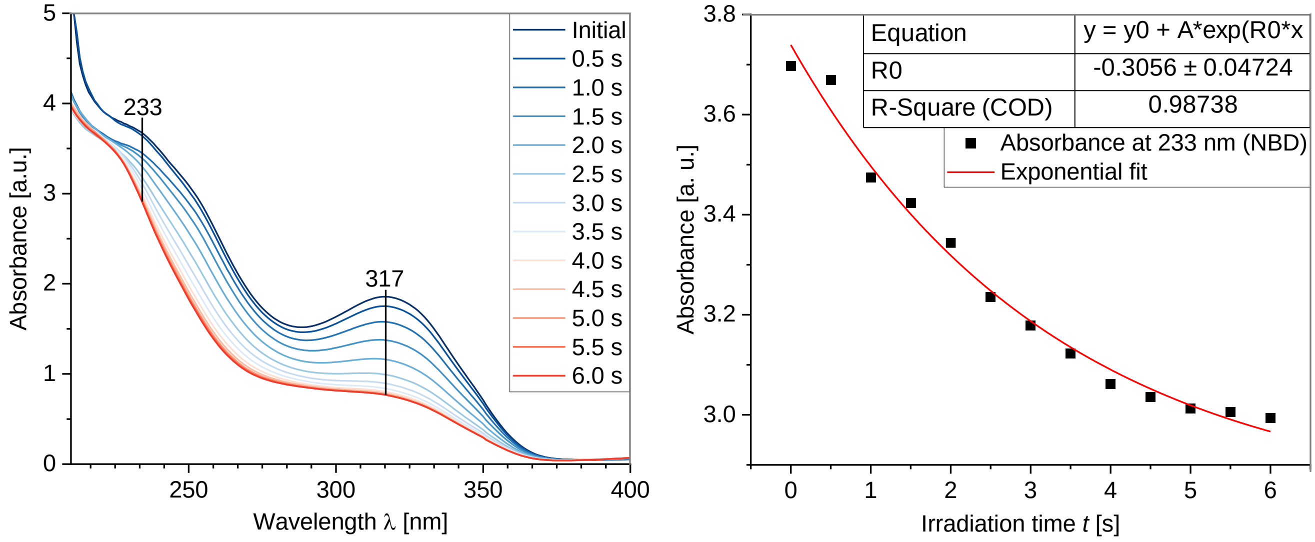

Figure S19. UV/Vis absorbance spectra of the **14**-NBD (100 µM in CH_3CN; left) and absorbance at 233 nm after different time intervals of irradiation (right) (light source: 4×Hönle lamp).

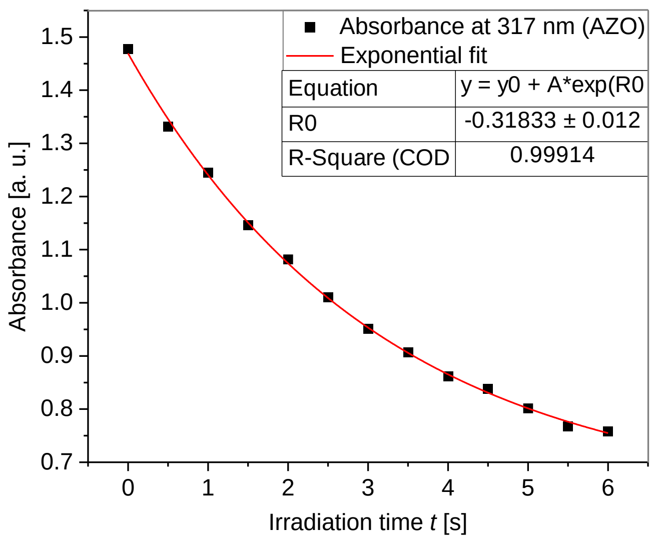

Figure S20. Absorbance at 317 nm of *trans*-**14** (100 µM in CH_3CN) after different time intervals of irradiation (light source: 4×Hönle lamp).

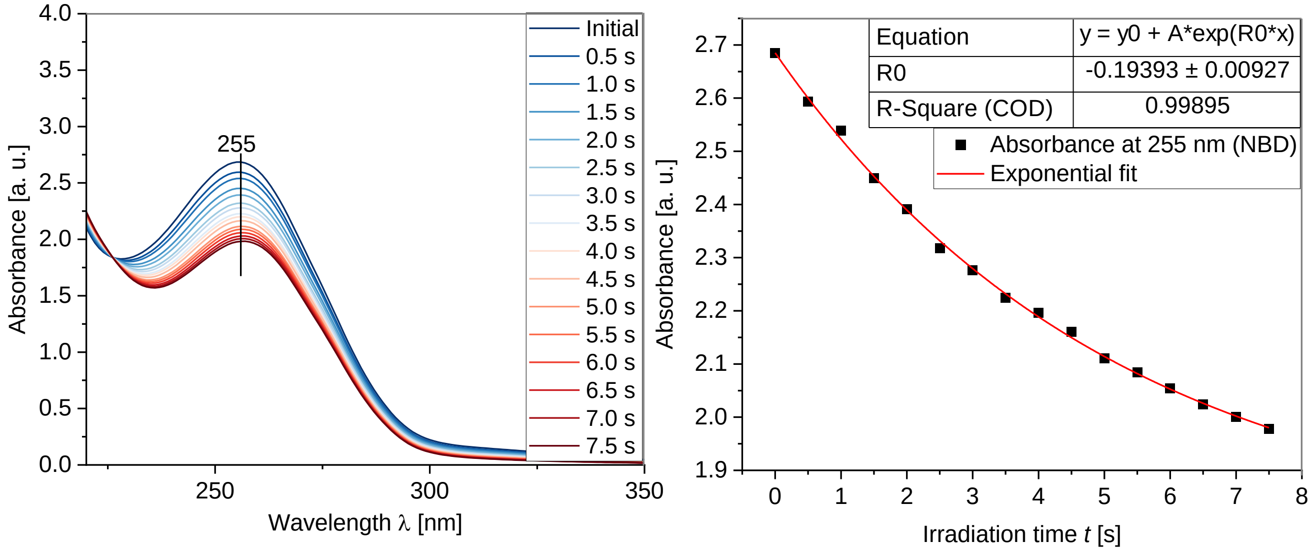

Figure S21. UV/Vis absorbance spectra of the **15**-NBD (100 µM in CH_3CN; left) and absorbance at 255 nm after different time intervals of irradiation (right) (light source: 4×Hönle lamp).

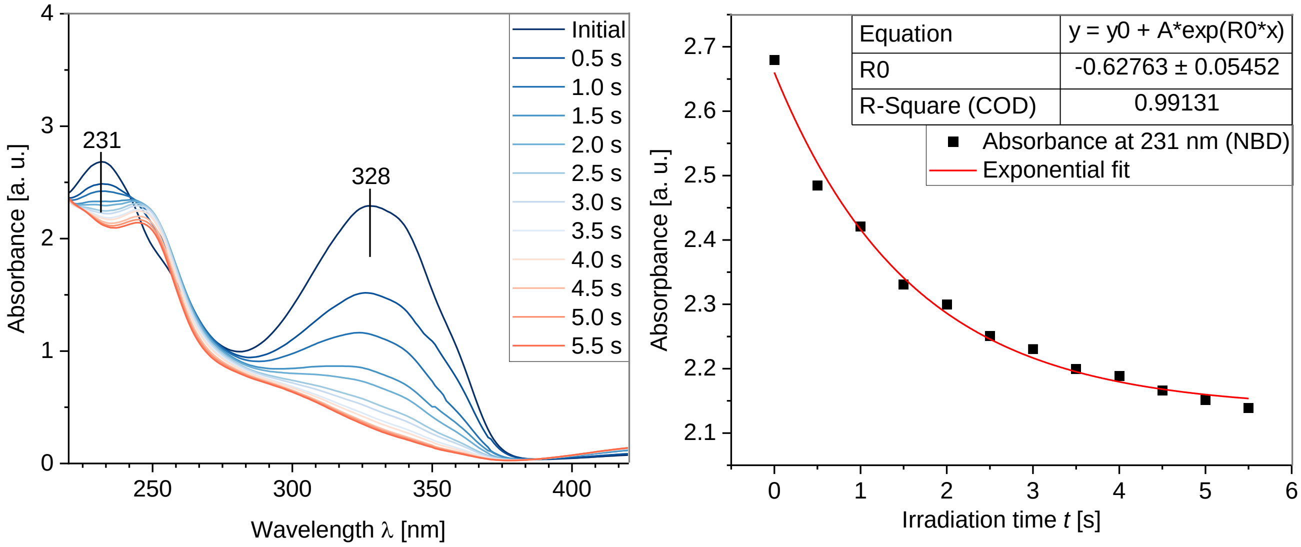

Figure S22. UV/Vis absorbance spectra of the **16**-NBD (100 µM in CH_3CN; left) and absorbance at 231 nm after different time intervals of irradiation (right) (light source: 4×Hönle lamp).

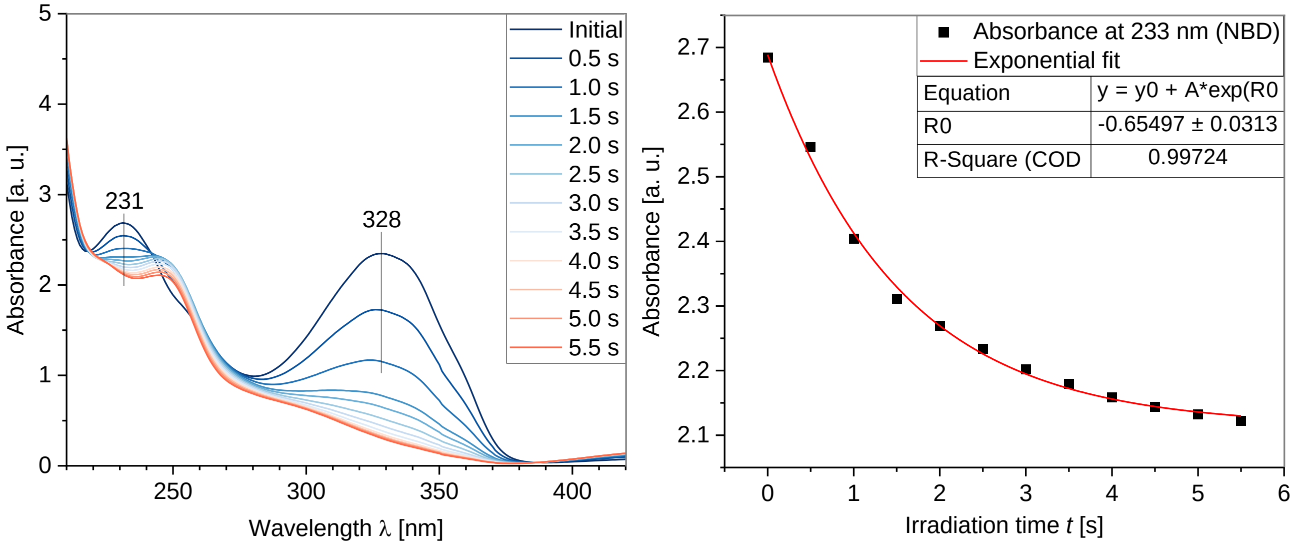

Figure S23. UV/Vis absorbance spectra of the **16**-NBD (100 µM in CH_3CN; left) and absorbance at 231 nm after different time intervals of irradiation (right) (light source: 4×Hönle lamp).

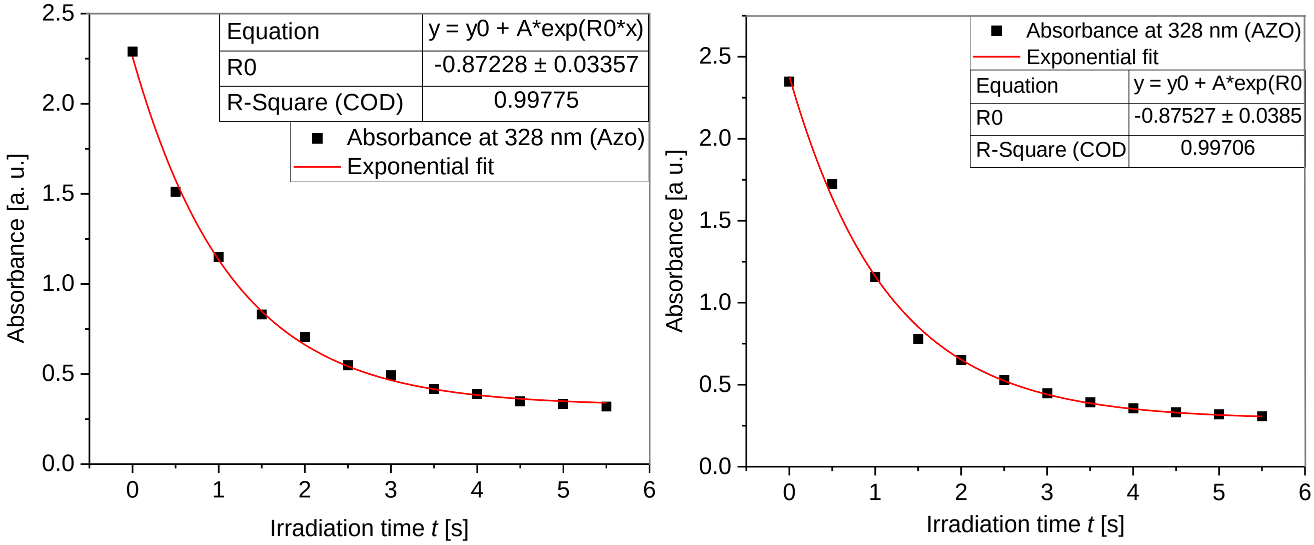

Figure S24. Absorbance at 328 nm of *trans*-**16** (100 µM in CH_3CN) after different time intervals of irradiation (light source: 4×Hönle lamp).

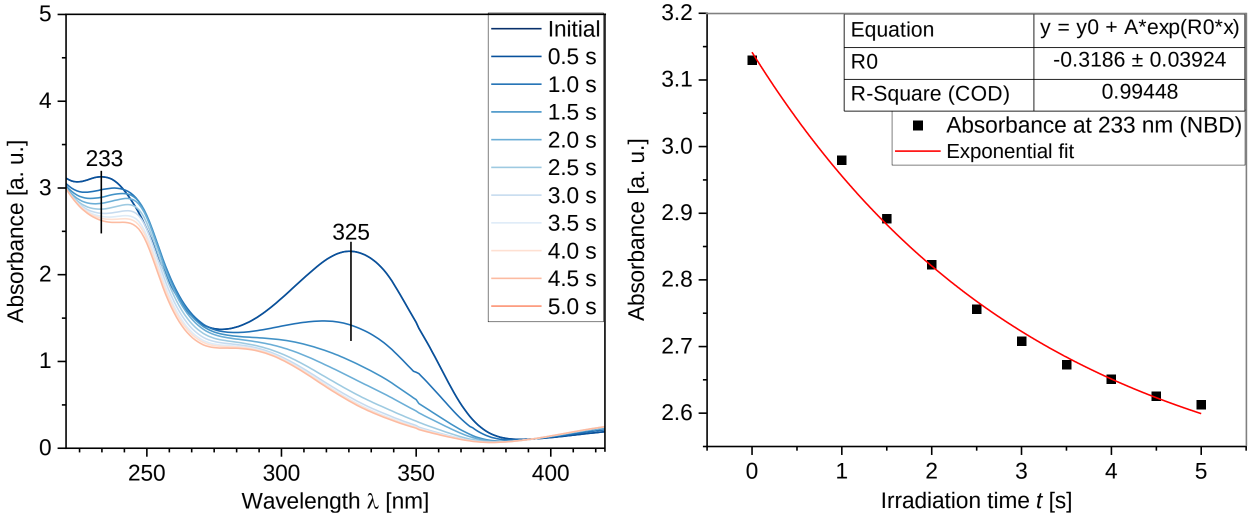

Figure S25. UV/Vis absorbance spectra of the **17**-NBD solution (100 µM in CH_3CN; left) and absorbance at 233 nm after different time intervals of irradiation (right) (light source: 4×Hönle lamp).

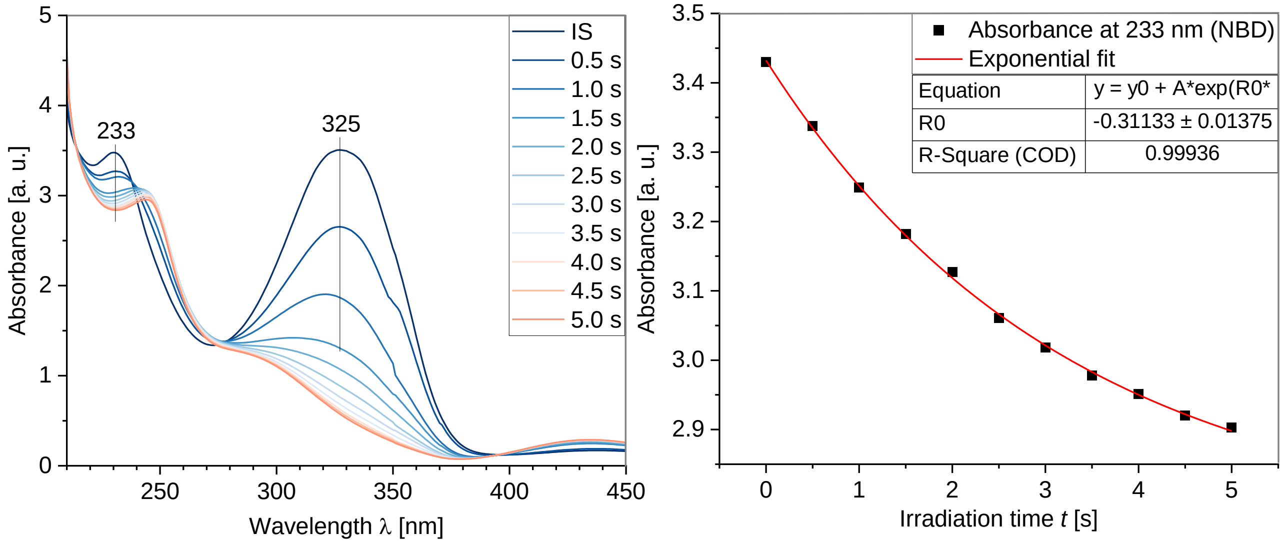

Figure S26. UV/Vis absorbance spectra of the **17**-NBD solution (100 µM in CH_3CN; left) and absorbance at 233 nm after different time intervals of irradiation (right) (light source: 4×Hönle lamp).

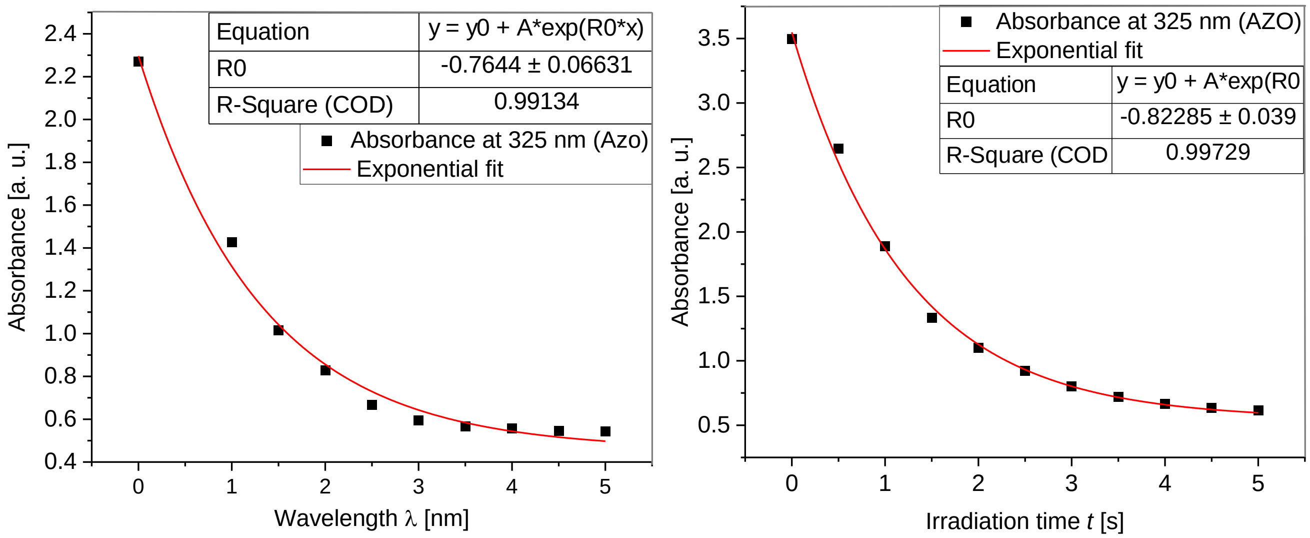

Figure S27. Absorbance at 325 nm of *trans*-**17** (100 µM in CH_3CN) after different time intervals of irradiation (light source: 4×Hönle lamp).

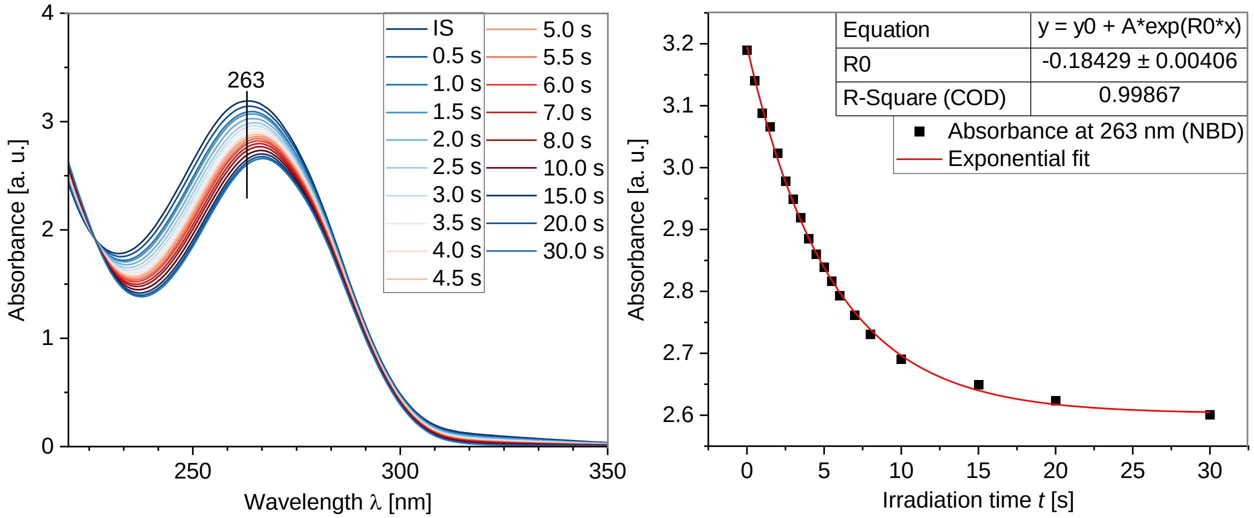

Figure S28. UV/Vis absorbance spectra of the **18**-NBD (100 µM in CH_3CN; left) and absorbance at 263 nm after different time intervals of irradiation (right) (light source: 4×Hönle lamp).

3. NMR Experiments and Half-Life of the Compounds

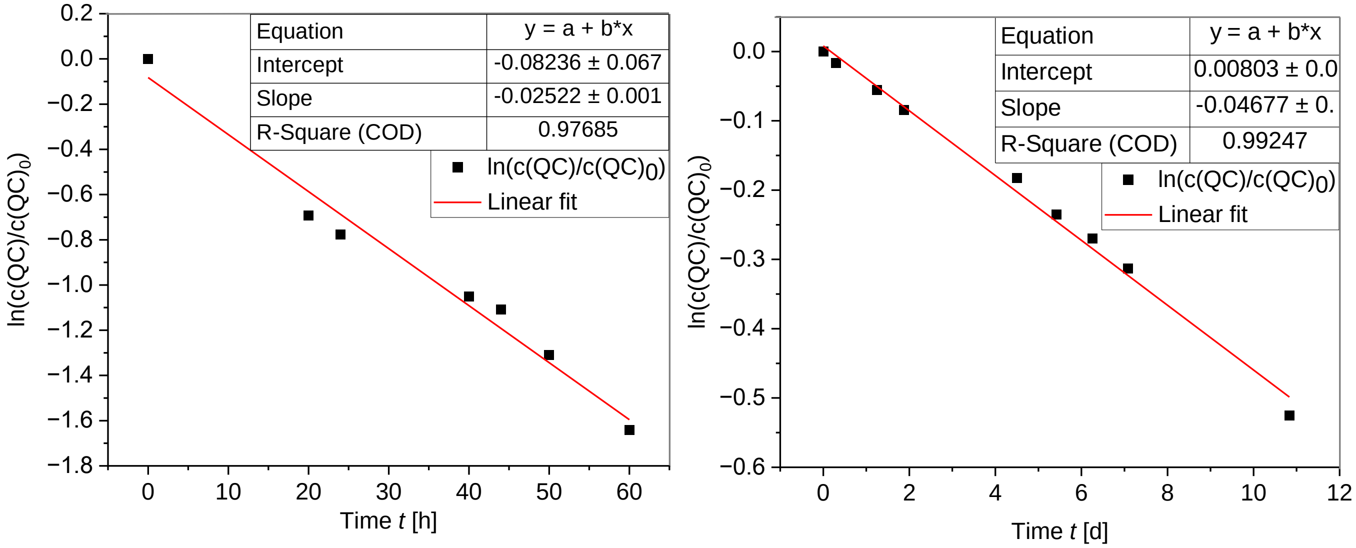

Figure S29. Logarithmized fraction of **5**-QC (left) and **6**-QC (right) determined by NMR spectroscopy at 100 °C plotted against time.

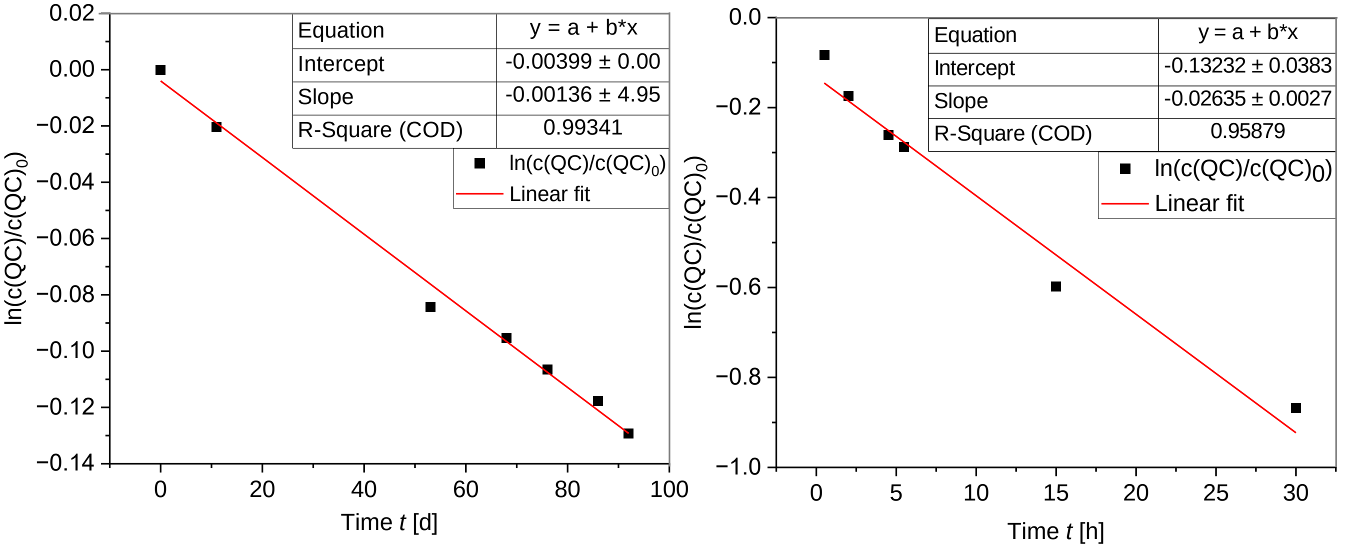

Figure S30. Logarithmized fraction of **7**-QC (left) and **8**-QC (right) determined by NMR spectroscopy plotted against time.

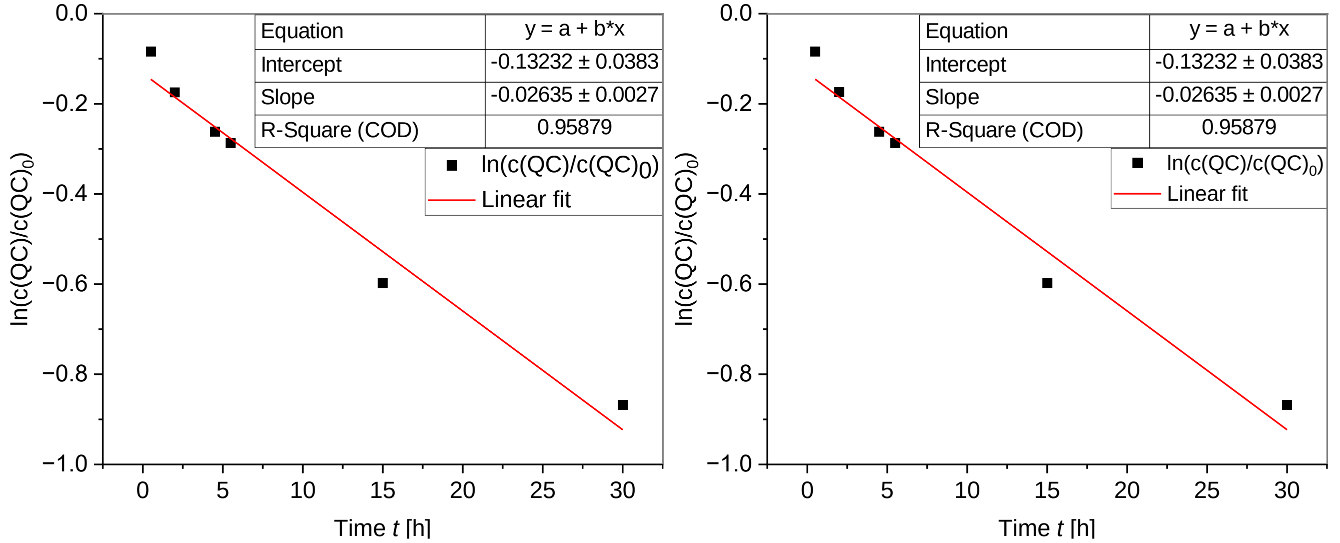

Figure S31. Logarithmized fraction of **9**-QC (left) and **10**-QC (right) determined by NMR spectroscopy plotted against time.

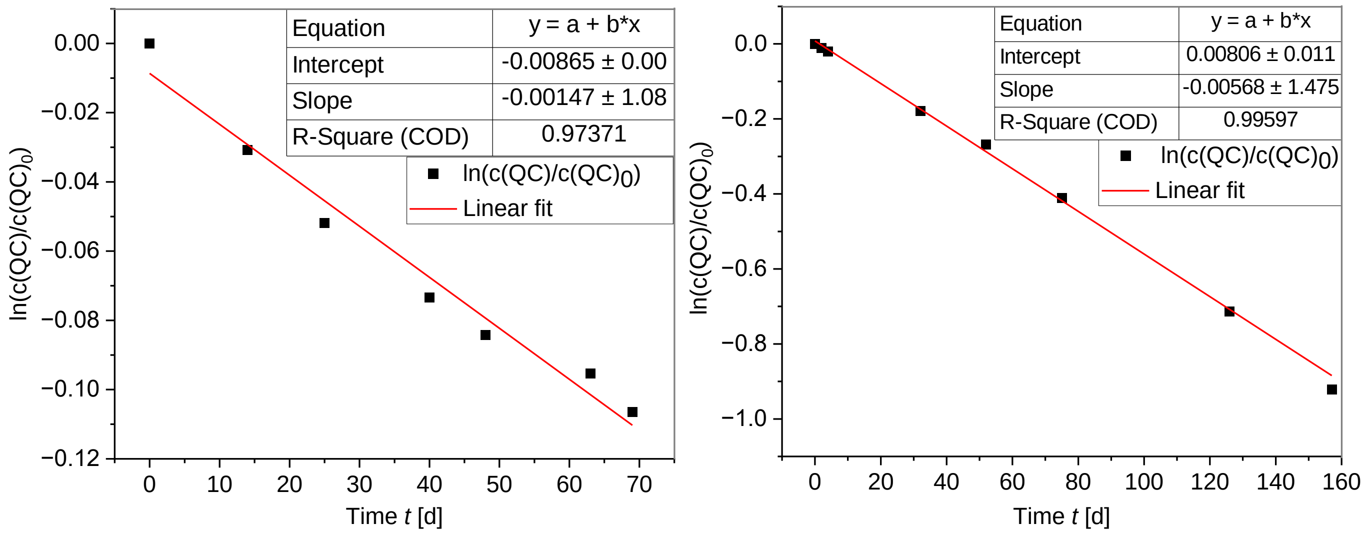

Figure S32. Logarithmized fraction of **11**-QC (left) and **12**-QC (right) determined by NMR spectroscopy plotted against time.

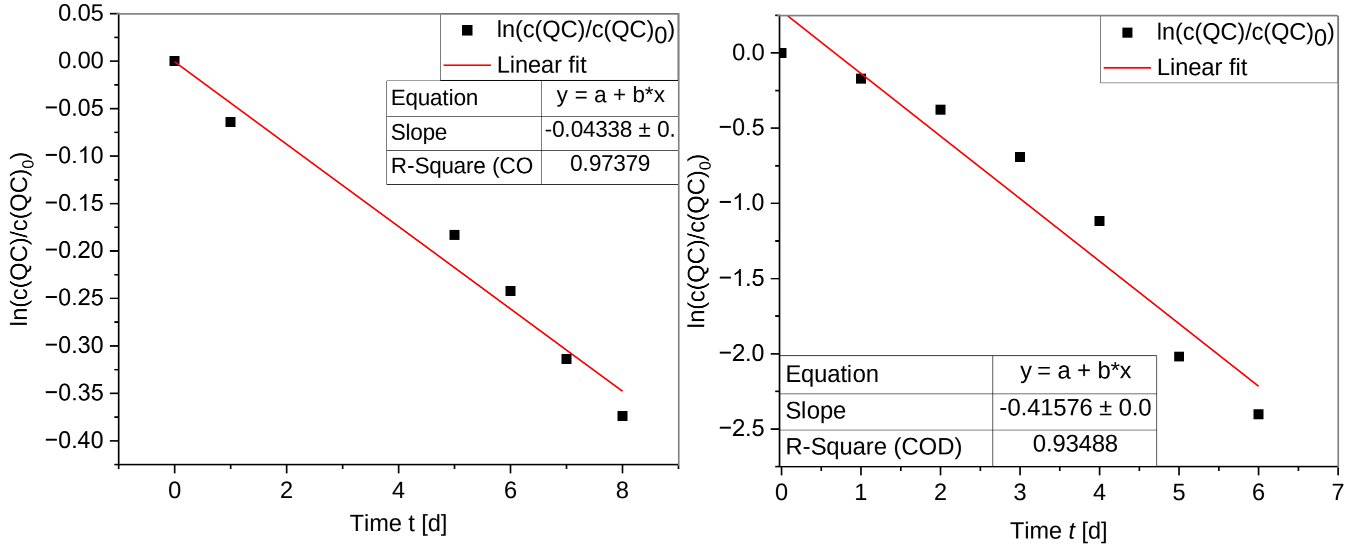

Figure S33. Logarithmized fraction of **12**-QC determined by NMR spectroscopy at 40 °C (left) and at 50 °C (right) plotted against time.

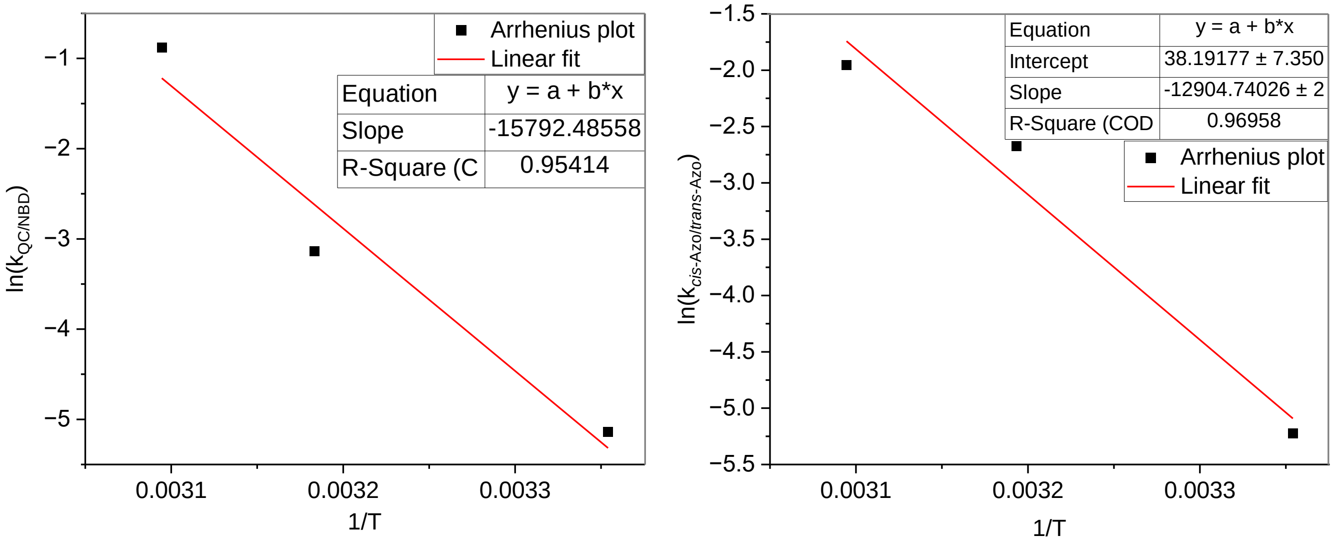

Figure S34. Arrhenius plot of **12**-QC (left) and *cis*-**12** (right) for the determination of the activation energy E_a.

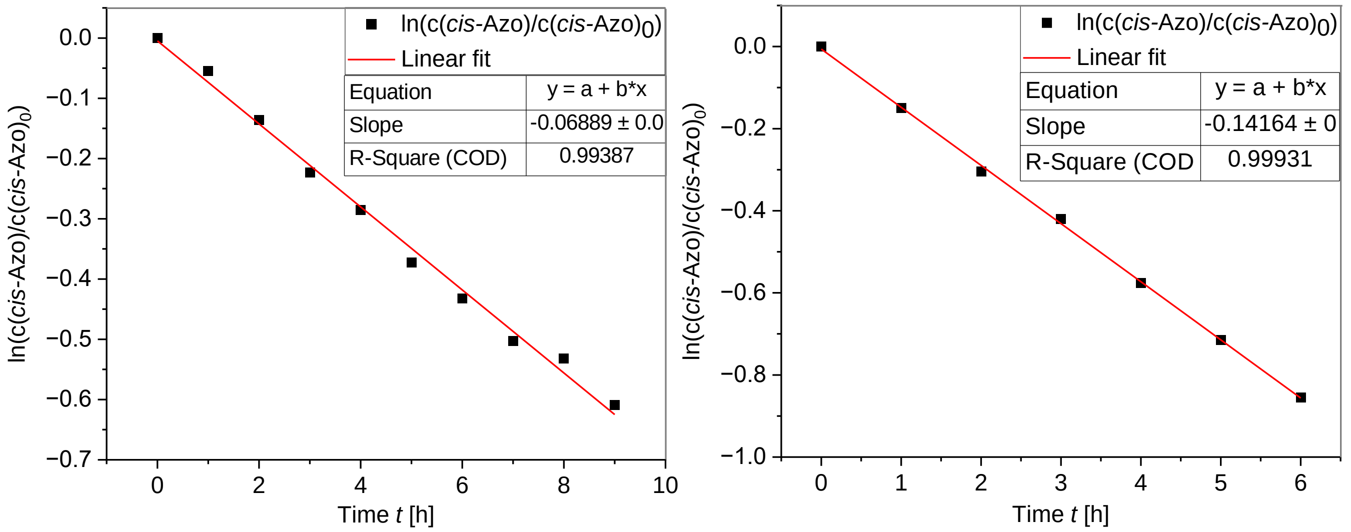

Figure S35. Logarithmized fraction of *cis*-**12** determined by NMR spectroscopy at 40 °C (left) and at 50°C (right) plotted against time.

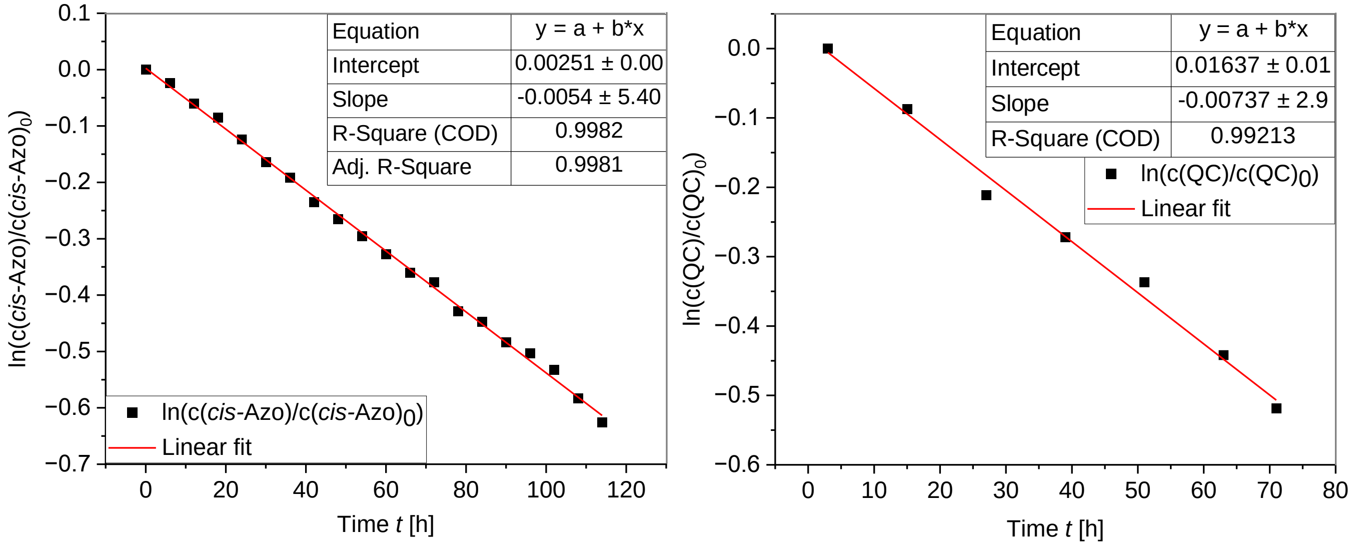

Figure S36. Logarithmized fraction of *cis*-**12** (left) and **13**-QC (right) determined by NMR spectroscopy plotted against time.

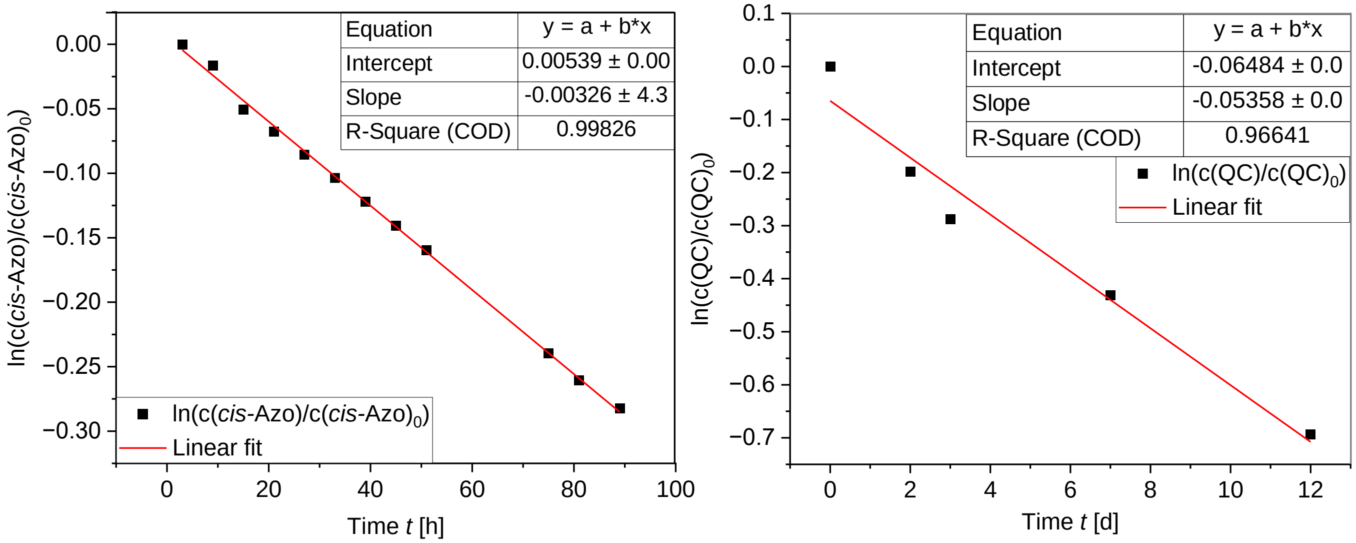

Figure S37. Logarithmized fraction of *cis*-**14** (left) and **14**-QC (right) determined by NMR spectroscopy plotted against time.

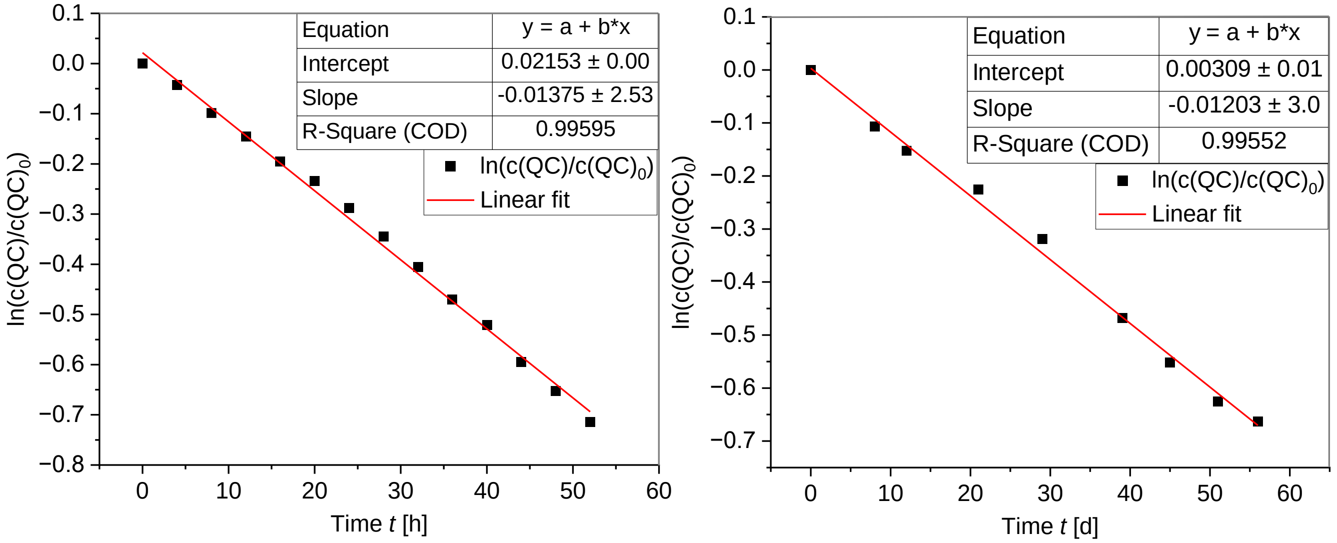

Figure S38. Logarithmized fraction of **15**-QC (left) and **16**-QC (right) determined by NMR spectroscopy plotted against time.

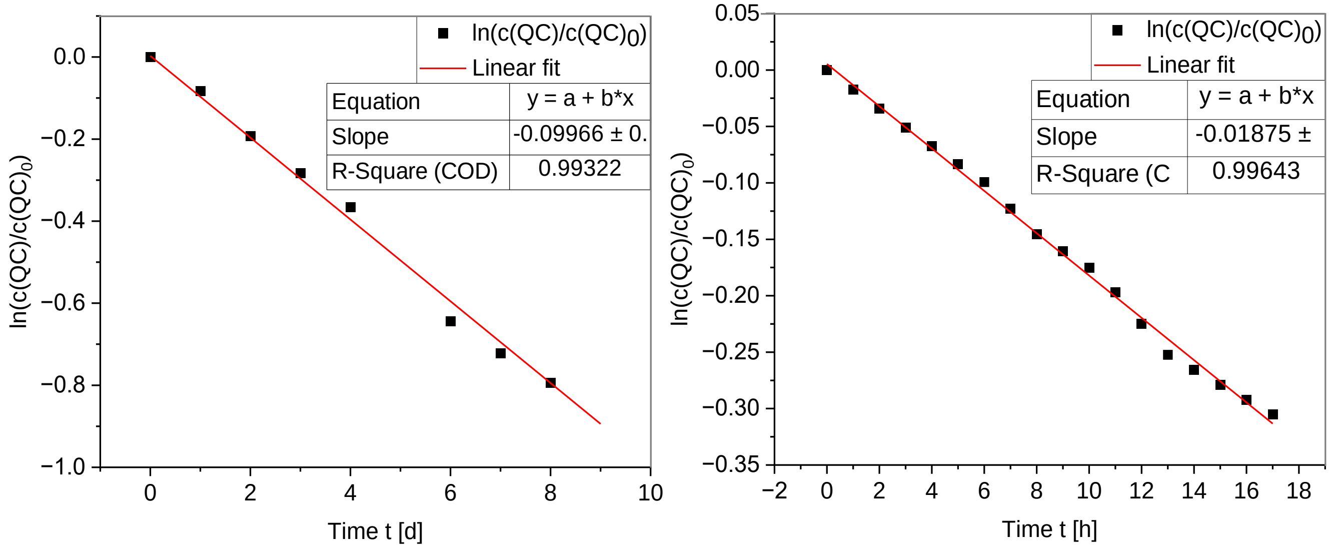

Figure S39. Logarithmized fraction of **16**-QC determined by NMR spectroscopy at 40 °C (left) and at 50°C (right) plotted against time.

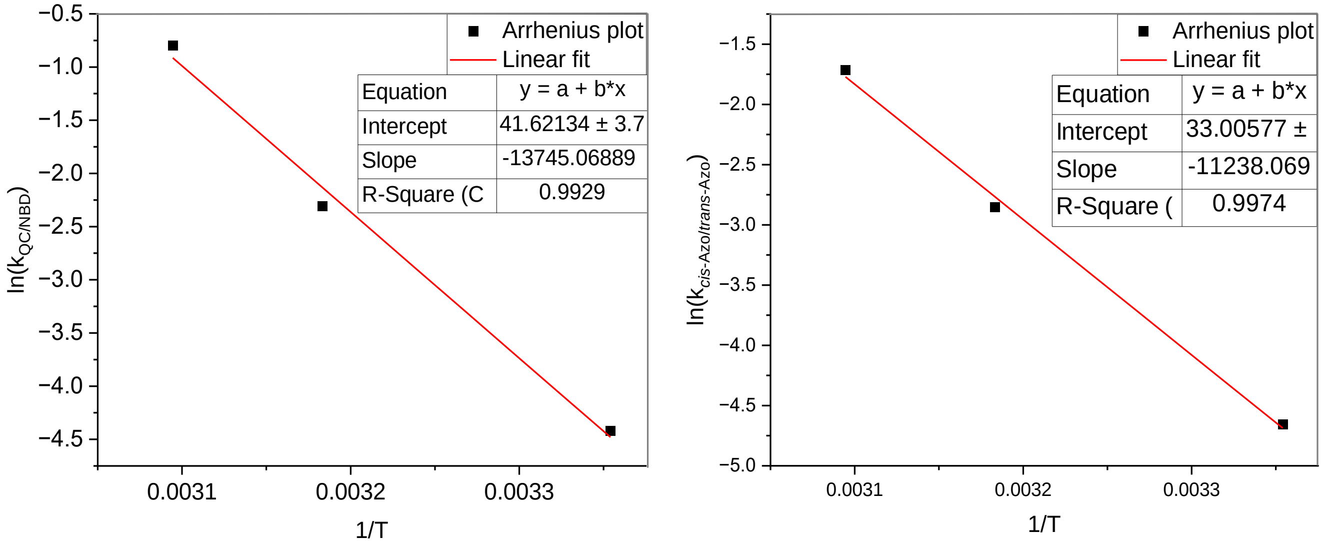

Figure S40. Arrhenius plot of **16**-QC (left) and *cis*-**16** (right) for the determination of the activation energy E_a.

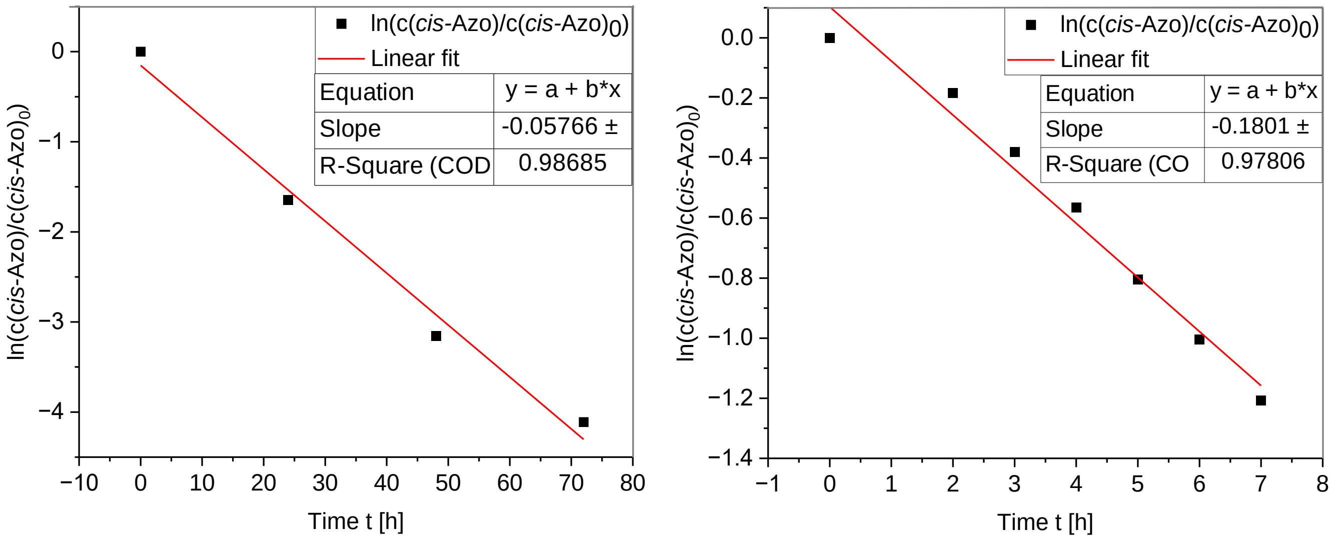

Figure S41. Logarithmized fraction of *cis*-**16** determined by NMR spectroscopy at 40 °C (left) and at 50°C (right) plotted against time.

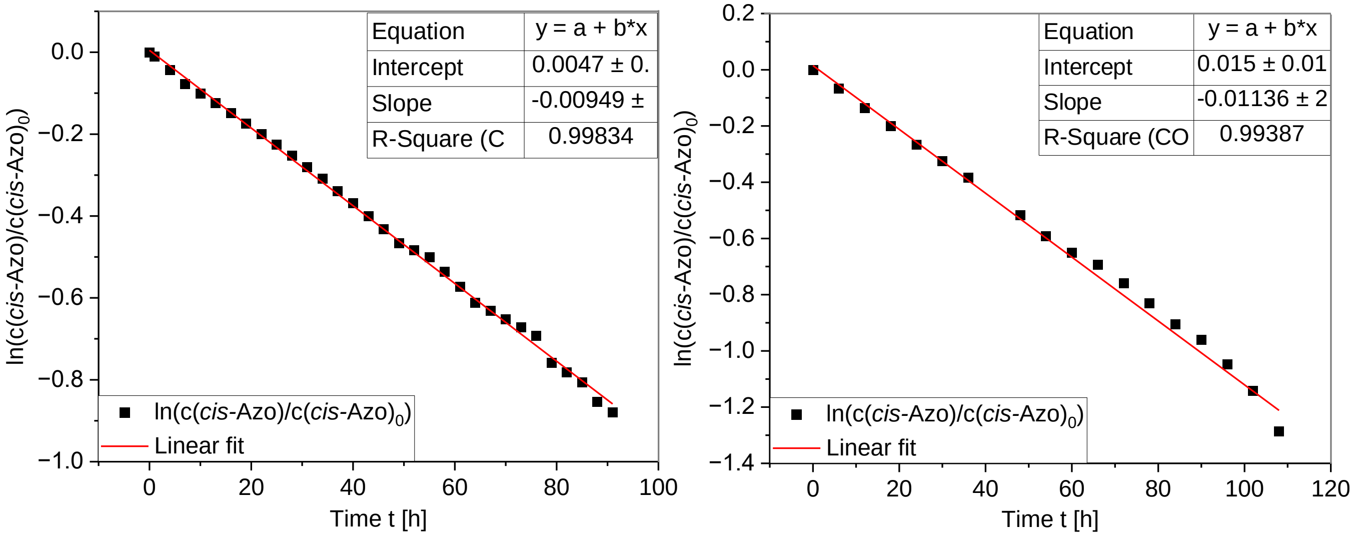

Figure S42. Logarithmized fraction of *cis*-**16** (left) and *cis*-**17** (right) determined by NMR spectroscopy plotted against time.

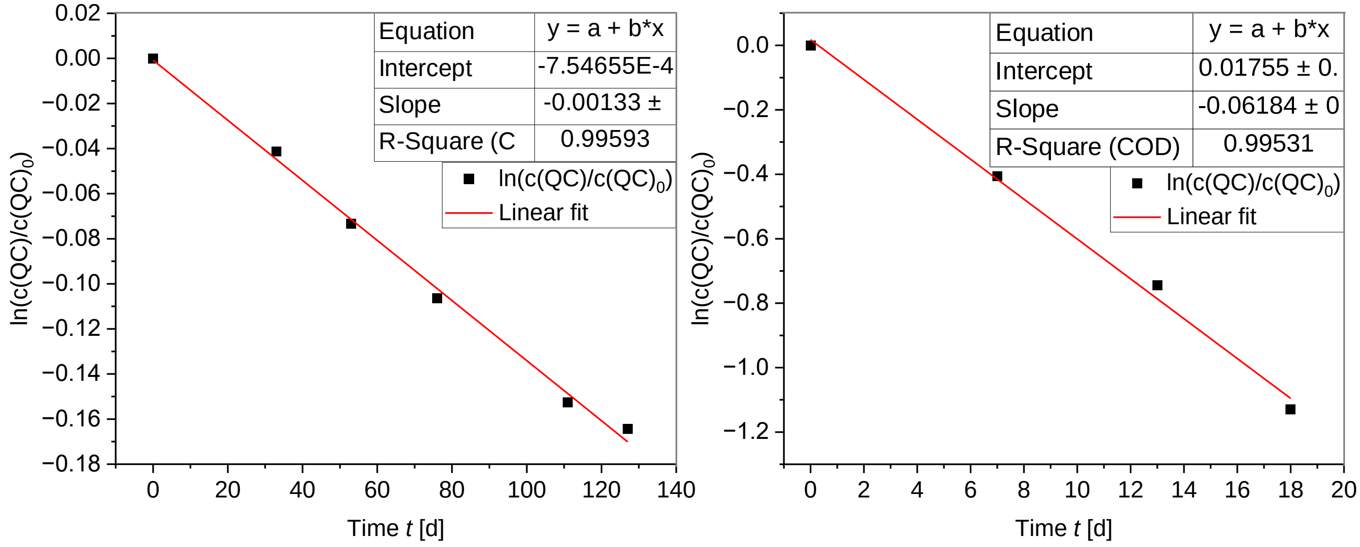

Figure S43. Logarithmized fraction of **17**-QC (left) and **18**-QC (right) determined by NMR spectroscopy plotted against time.

Table S1. Photostationary ratios (PSS) for all systems after the irradiation of the solutions in $CDCl_3$ with light of the wavelength λ = 365 nm (λ = 540 nm for *trans*-**13**) determined by NMR spectroscopy.

Compound	QC (PSS) [%]	*cis*-AZO (PSS) [%]
**5**-NBD	98	-
**6**-NBD	96	-
**7**-NBD	93	-
**8**-NBD	97	-
**9**-NBD	98	-
**10**-NBD	90	-
**11**-NBD	98	-
**12**-NBD	97	83
**13**-NBD	86	86
**14**-NBD	96	71
**15**-NBD	95	-
**16**-NBD	94	90
**17**-NBD	93	91
**18**-NBD	98	-

4. NMR Spectra of Isomers

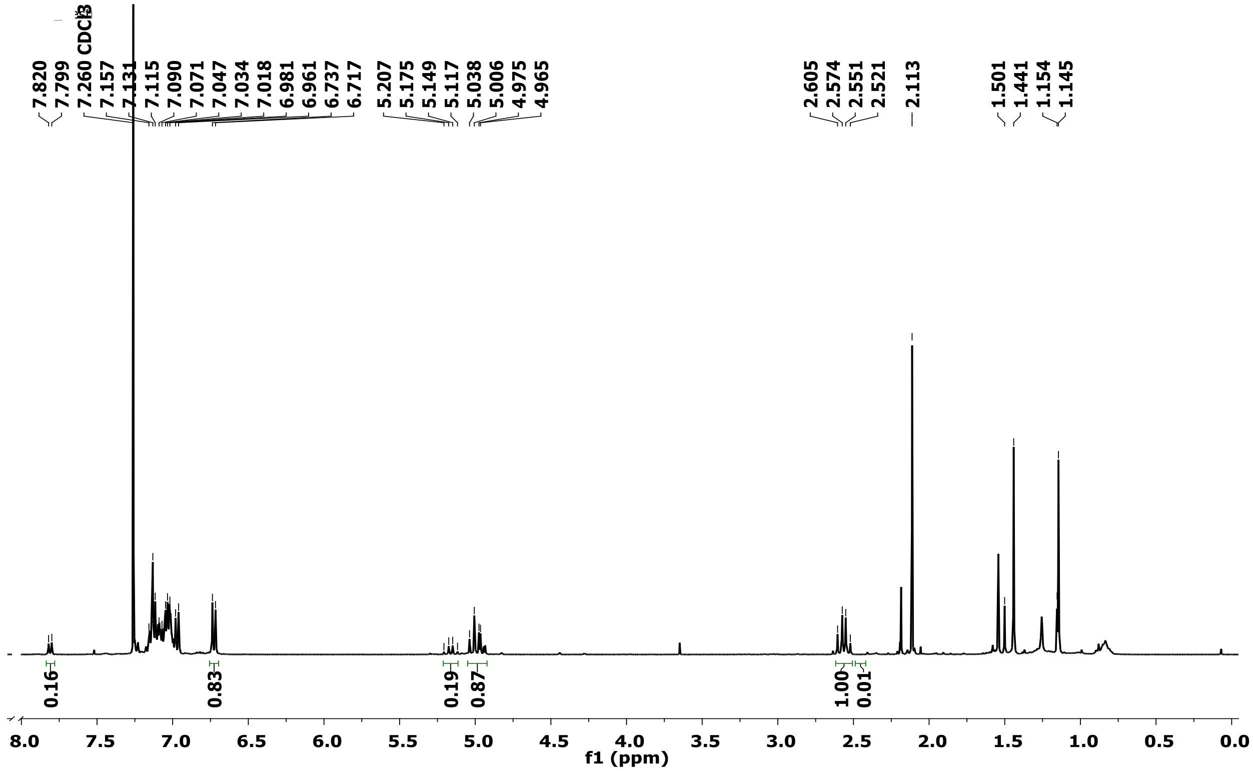

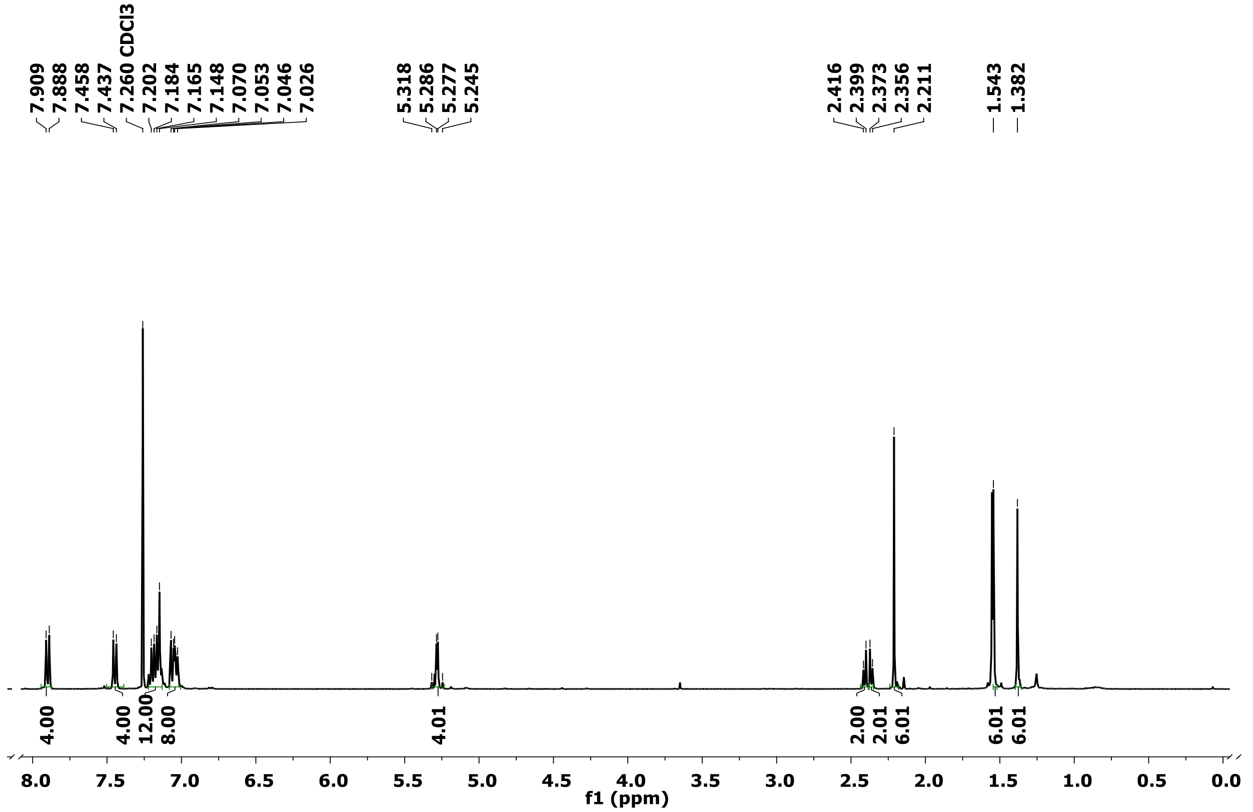

Figure S44. ^{1}H NMR spectrum of *trans*-**12**-NBD (below) and *cis*-**12**-QC (above) (400 MHz, $CDCl_3$, 5 mM, 298 K).

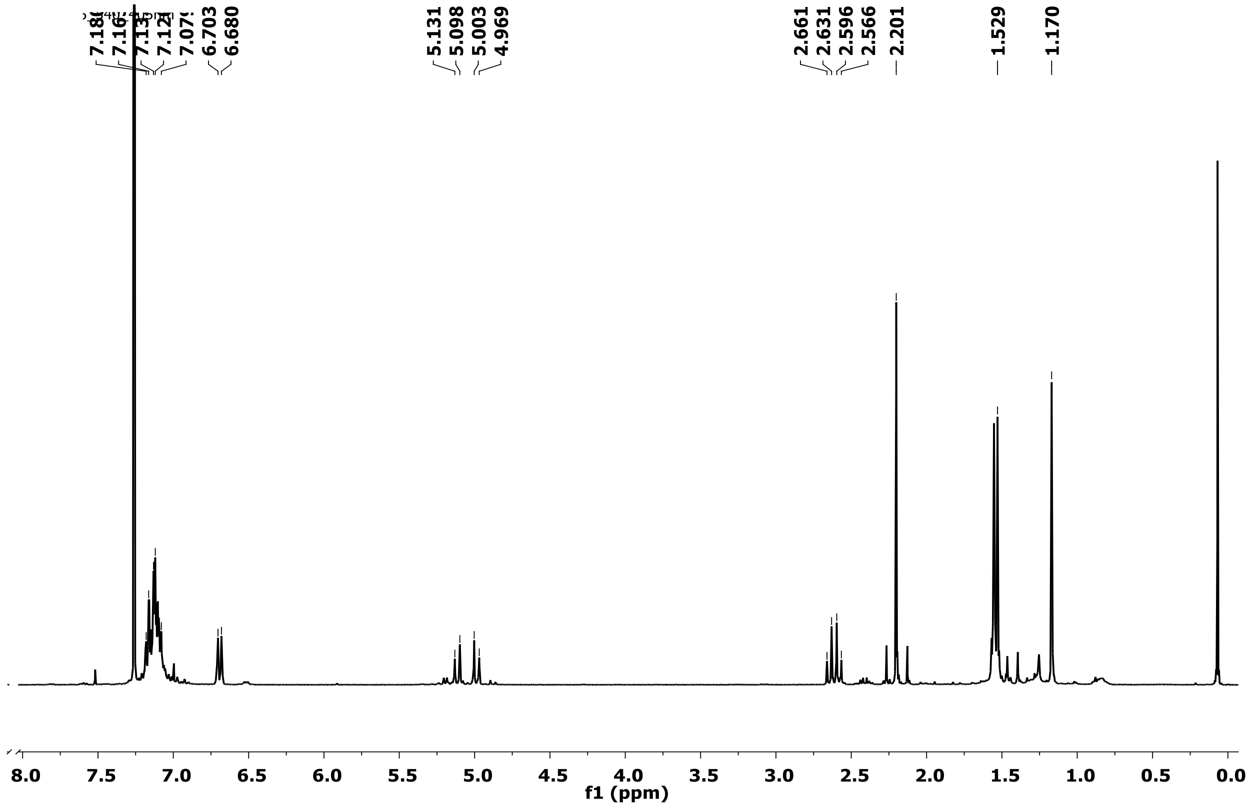

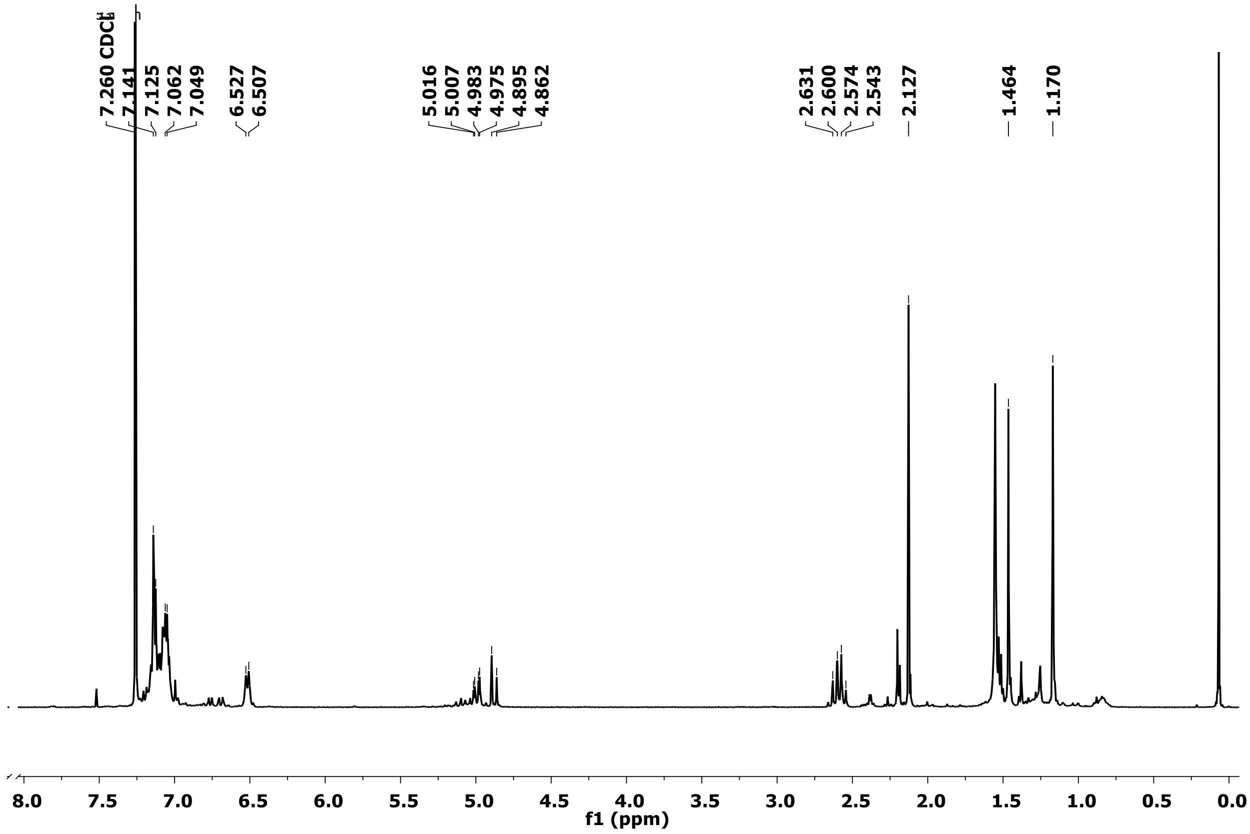

Figure S45. ^{1}H NMR spectrum of *cis*-**13**-QC (below) and *trans*-**13**-QC (above) (400 MHz, $CDCl_3$, 5 mM, 298 K).

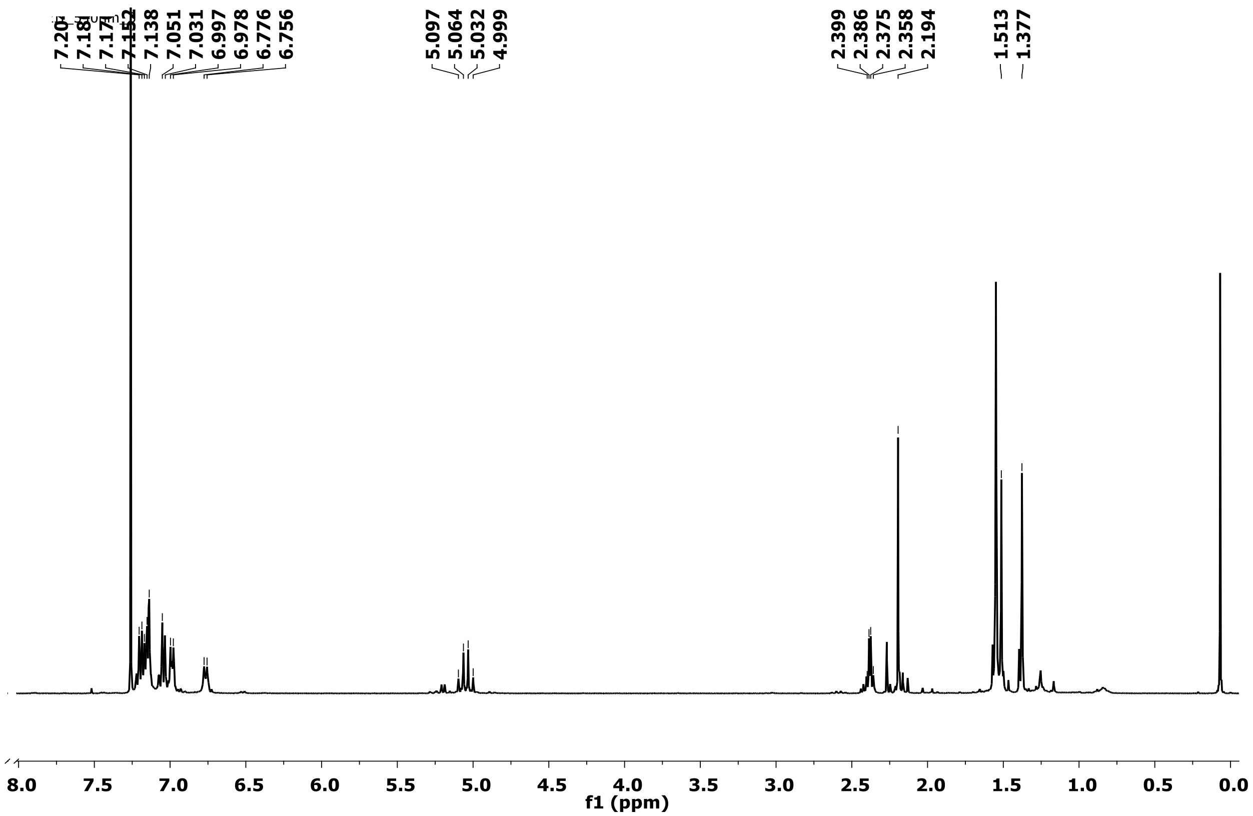

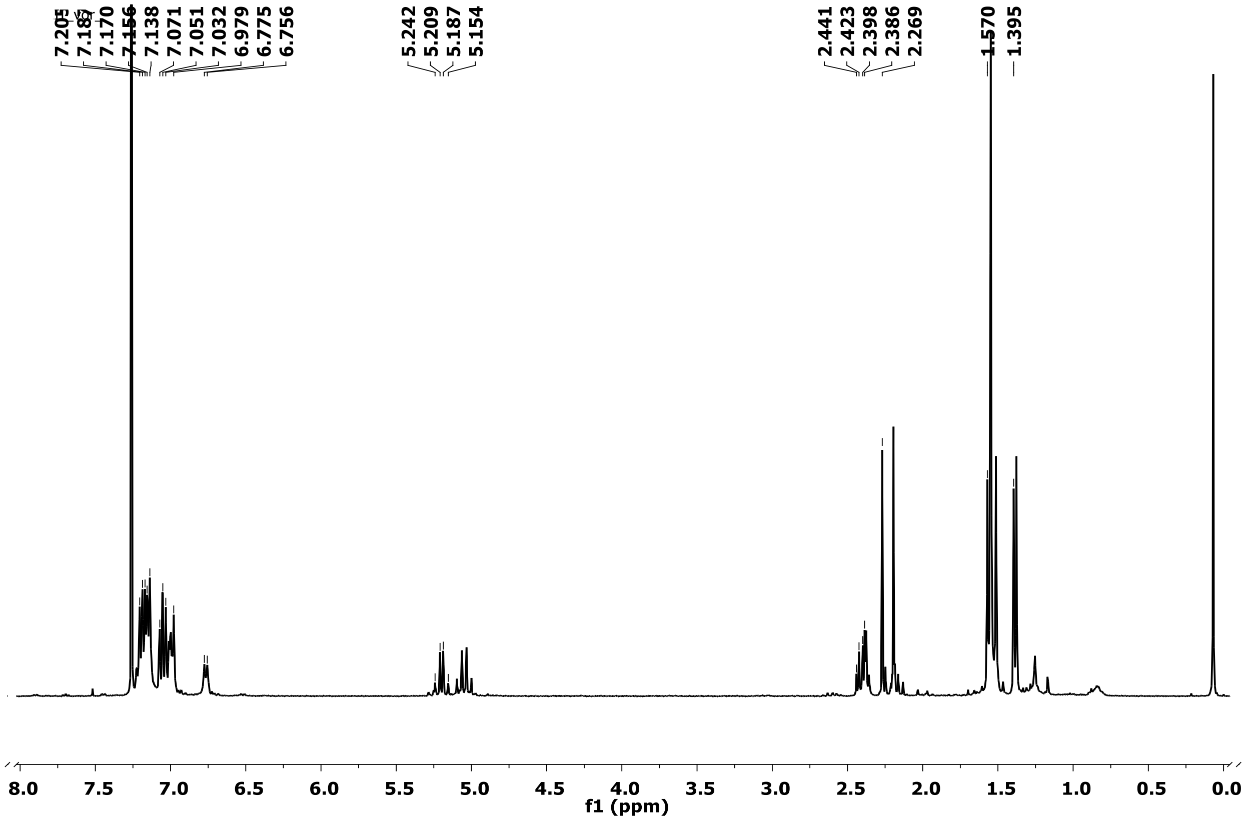

Figure S46. ^{1}H NMR spectrum of *trans*-**13**-NBD (below) and *cis*-**13**-NBD (above) (400 MHz, $CDCl_3$, 5 mM, 298 K).

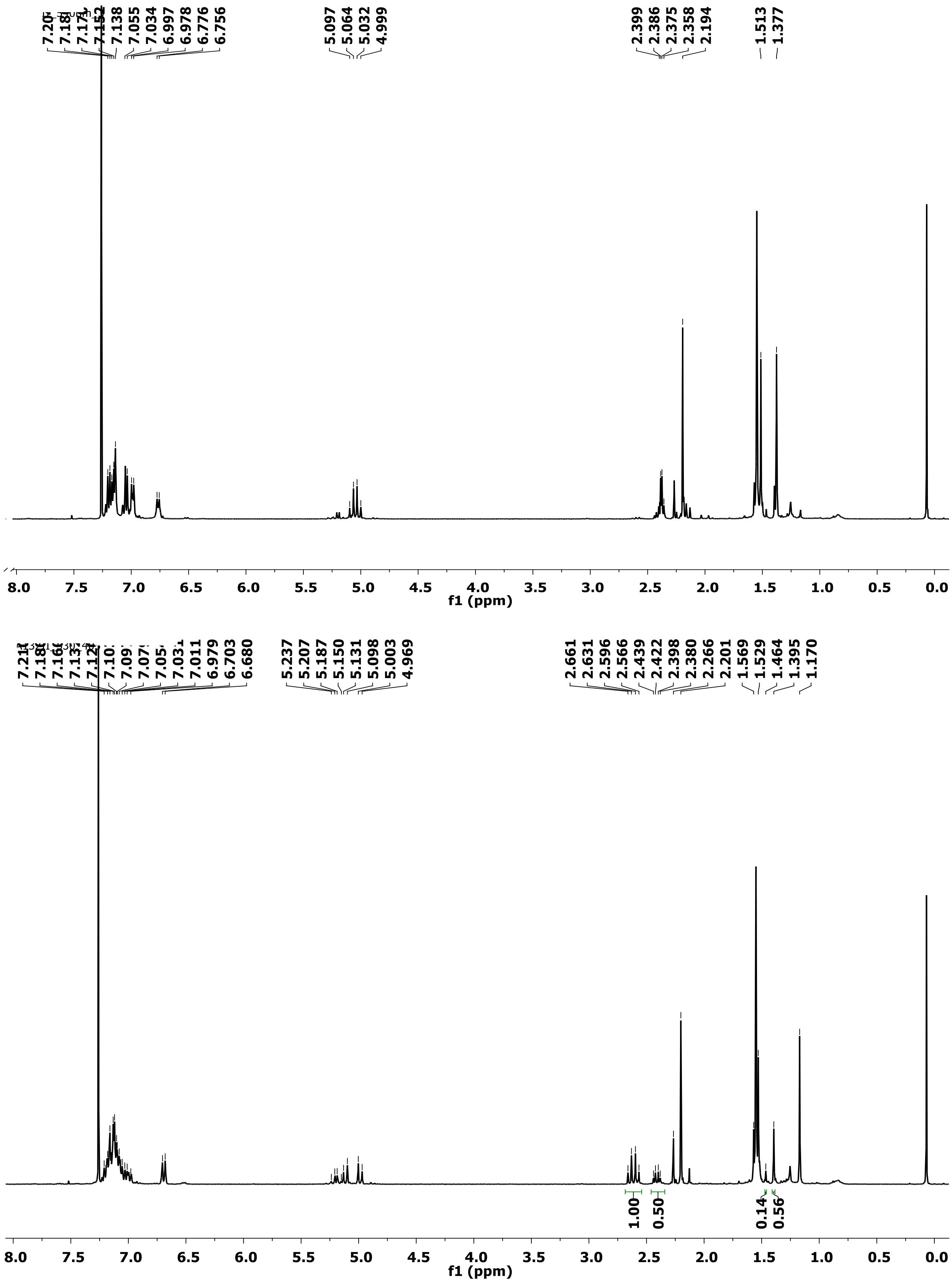

Figure S47. ^{1}H NMR spectrum of a *cis*-**13**-NBD before (above) and after (below) irradiation with the wavelength of λ = 405 nm (400 MHz, $CDCl_3$, 5 mM, 298 K).

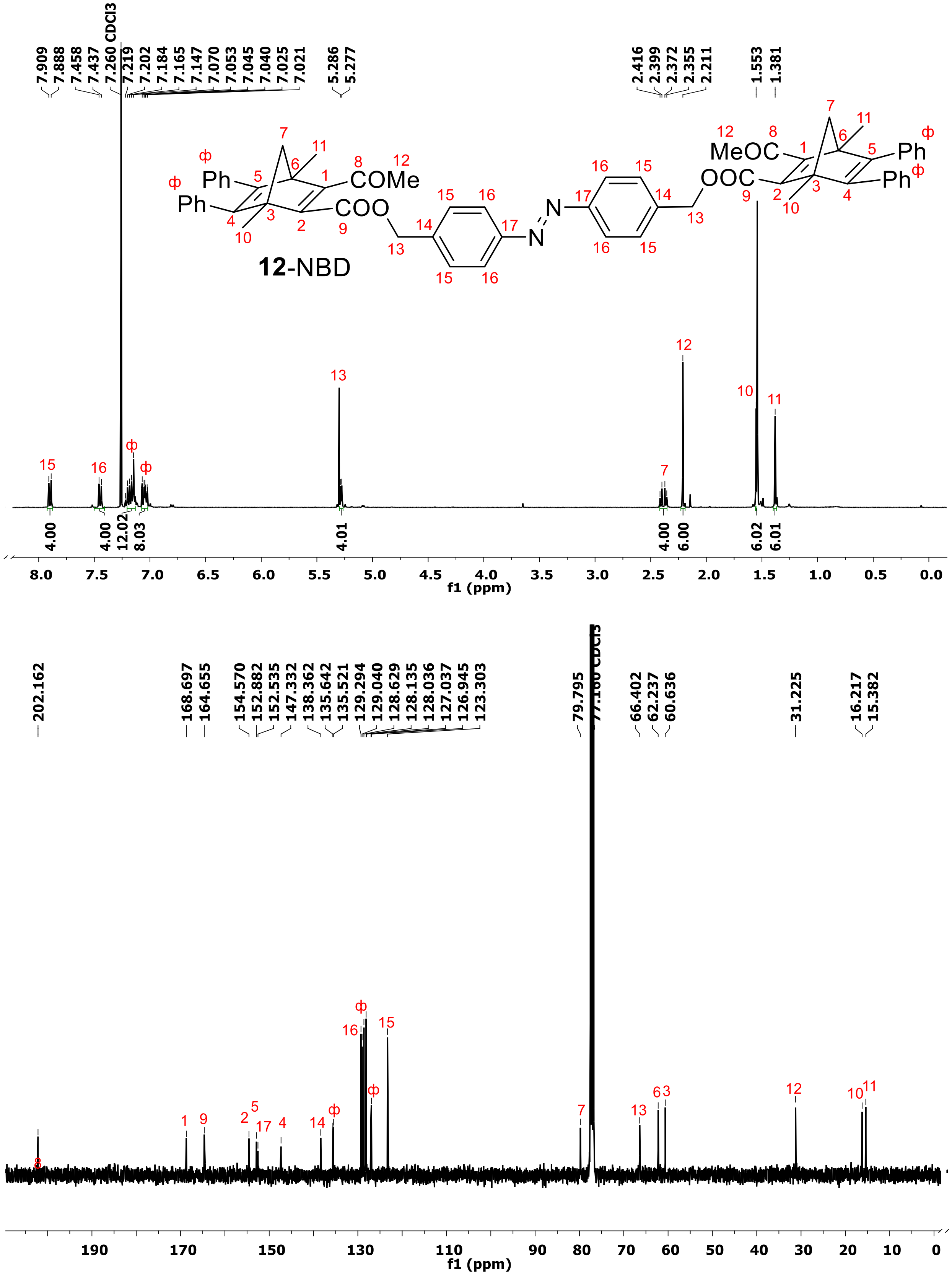

Figure S48. ^{1}H and ^{13}C NMR spectra with full assignment of *trans*-**12**-NBD (400 MHz, $CDCl_3$, 5 mм, 298 K).

5. Synthesis of New Compounds

General remarks. All chemicals were reagent grade and used without further purification. Reactions were monitored by TLC analysis with silica gel 60 F254 thin-layer plates. Flash chromatography was carried out on silica 60 (40-63 µm, 230-400 mesh). ^{1}H and ^{13}C NMR spectra were measured with Bruker Avance DRX 400, 500 and Avance HD 600 spectrometers. All chemical shifts () are given in ppm. The spectra were referenced to the peak for the protium impurity in the deuterated solvents indicated in brackets in the analytical data. Signal multiplicity for ^{1}H NMR was determined as s (singlet), d (doublet), t (triplet), sext (sextet), sept (septet), m (multiplet), dd (doublet of doublets) and td (triplet of doublets). ^{13}C NMR spectra were measured with ^{1}H decoupling and the ^{13}C assignment was achieved via DEPT 135, HSQC, HMBC, and COSY spectra. ^{13}C signal multiplicity was determined as p (primary), s (secondary), t (tertiary), q (quaternary). HR-MS spectra were recorded with a Bruker BioTOF III Instrument with ESI as ionization source. UV/Vis absorption spectra were obtained with a Jasco V-550 spectrophotometer. IR spectroscopy was perfomed on a Shimadzu IR Tracer-100. Cyclopentadiene **26** was provided by Squarix GmbH.

General procedure of esterification *A*-*C*

To a solution of an NBD (number of eq. see below) in MeCN (40 mL), K_2CO_3 (5 eq.) was added, and the mixture was stirred at an ambient temperature for 1 h. The solution of the corresponding bromide (number of eq. see below) in MeCN (20 mL) was then added and the reaction mixture was refluxed overnight. The solvent was then removed in vacuo and the residue dissolved in DCM. The solution was washed with water (3×10 mL). The aqueous phase was extracted with DCM (3×10 mL). The combined organic phases were combined, washed with brine (10 mL), dried over Na_2SO_4 and concentrated in vacuo. The crude product was purified by column chromatography (hexanes/EtOAc = 6:1) to afford the desired product.

General procedure *A*: NBD (1.1. eq) and bromide (1.0 eq.)

General procedure *B*: NBD (2.2. eq) and bromide (1.0 eq.)

General procedure *C*: NBD (1.0. eq) and bromide (1.8 eq.)

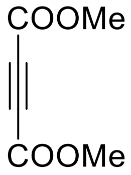

25

Synthesis of alkyne 25. Acetylenedicarboxylic acid (25.0 g, 219.0 mmol) was dissolved in MeOH (130 mL) and the solution was cooled to 0 °C. 28 mL of conc. H_2SO_4 were added at this temperature and the mixture stirred for four days at room temperature. The reaction was terminated by the addition of a 1 M NaOH (300 mL) solution. The residue was extracted with diethyl ether (3×20 mL). The combined organic phases were washed with water (20 mL) and an aqueous saturated $NaHCO_3$ solution (20 mL). The organic layers were combined, dried over $MgSO_4$ and concentrated in vacuo. The crude product was purified using a silica plug (hexanes/DCM = 1:1) to yield diester **25** as a yellow liquid (13.5 g, 94.5 mmol, 43%).

^{1}H-NMR (400 MHz, $CDCl_3$): δ = 3.85 ppm (s, 6 H). ^{13}C NMR (100 MHz, $CDCl_3$): δ = 152.3 (CO), 74.8 (C_{alkyne}), 53.6 ppm ($CO_2\mathit{C}H_3$).

The spectroscopic data are consistent with the literature.[4]

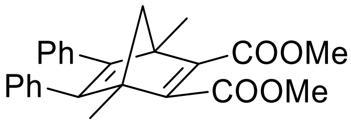

9-NBD

Synthesis of 9-NBD. A solution containing cyclopentadiene **26** (345 mg, 1.40 mmol) and alkyne **25** (152 mg, 1.07 mmol) was prepared by dissolving the reagents in benzene (25 mL). The solution was heated to reflux for 48 hours. Afterwards, the solvent was removed in vacuo. The residue was purified by chromatography on silica gel (hexanes/EtOAc = 24:1) to yield diester **9**-NBD as a yellow liquid (405 mg, 1.04 mmol, 97%).

^{1}H NMR (400 MHz, $CDCl_3$): δ = 7.24–7.12 (m, 6 H; C_{ar}H), 7.07 (dd, $^4J_{H,H}$ = 8.2, 1.5 Hz, 4 H; C_{ar}H), 3.80 (s, 6 H; CO_2CH_3), 2.42 (d, $^2J_{H,H}$ = 6.8 Hz, 1 H; CH_2), 2.38 (d, $^2J_{H,H}$ = 6.8 Hz, 1 H; CH_2), 1.46 ppm (s, 6 H; CH_3). ^{13}C NMR (100 MHz, $CDCl_3$): δ = 166.1 (CO), 154.7 (C_{alkene}), 153.7 (C_{alkene}), 135.7 (C_{ar}), 128.7 (C_{ar}), 128.0 (C_{ar}), 126.9 (C_{ar}), 80.2 (CH_2), 61.3 ($CO_2\mathit{C}H_3$), 52.1 (C), 15.5 ppm (CH_3).

The spectroscopic data are consistent with the literature.[5]

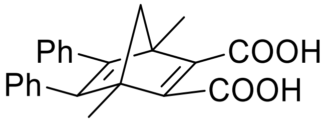

28-NBD

Synthesis of 28-NBD. Compound **9**-NBD (723 mg, 1.86 mmol) was dissolved in MeOH (10 mL), mixed with an 1 M NaOH (9 mL, 9.00 mmol) and heated to reflux overnight. Subsequently, the reaction mixture was acidified with 2 M HCl, to pH = 1. The aqueous phase was extracted with diethyl ether (3×20 mL). The combined organic layers were washed with water (20 mL), dried over Na_2SO_4, and the solvent was removed in vacuo to afford the desired compound **28**-NBD as a yellow powder (600 mg, 1.67 mmol, 90%), which was used in the next step without further purification.

M.p.: 181 °C. ^{1}H NMR (400 MHz, $CDCl_3$): δ = 7.23–7.12 (m, 6 H; C_{ar}H), 7.09–7.06 (m, 4 H; C_{ar}H), 2.46–2.41 (m, 2 H; CH_2), 1.53 ppm (s, 6 H; CH_3). ^{13}C NMR (100 MHz, $CDCl_3$): δ = 170.2 (CO), 156.8 (C_{alkene}), 153.5 (C_{alkene}), 135.3 (C_{ar}), 130.6 (C_{ar}), 128.8 (C_{ar}), 128.3 (C_{ar}), 128.1 (C_{ar}), 127.1 (C_{ar}), 80.1 (CH_2), 61.6 (C), 15.8 ppm (CH_3). IR (ATR): $\tilde{\nu}$ = 1724, 1672, 1593, 1458, 1445, 1385, 1342, 1192, 1165, 1092, 1055, 1020, 982, 949, 930, 891, 868, 802, 770, 748, 729, 694, 667 cm^{-1}. UV/Vis (CH_3CN): λ_{max} (log ε) = 230 nm (3.84). HRMS (ESI-TOF) m/z: $[C_{23}H_{20}O_4+H]^+$ calculated: 361.1434; found: 361.1437; $[C_{23}H_{20}O_4+Na]^+$ calculated: 383.1254; found: 383.1257.

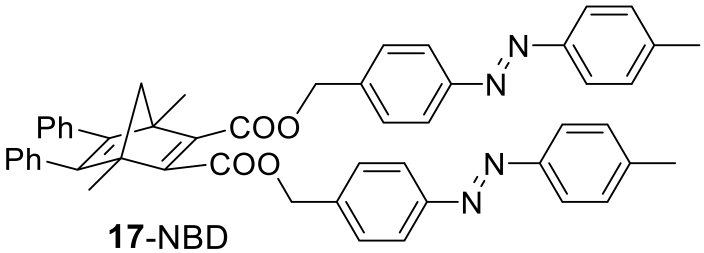

17-NBD

Synthesis of 17-NBD. Compound **28**-NBD (54 mg, 0.15 mmol) and azobenzene **32** (78 mg, 0.27 mmol) were treated according to general procedure *C*. The crude product was purified by column chromatography (hexanes/EtOAc = 6:1) to provide **17**-NBD as a red solid (39 mg, 0.05 mmol, 37%).

M.p.: 161 °C. ^{1}H NMR (400 MHz, $CDCl_3$): δ = 7.82 (dd, $^3J_{H,H}$ = 8.3, 3.6 Hz, 8 H; C_{azo}H), 7.36 (d, $^3J_{H,H}$ = 8.4 Hz, 4 H; C_{ar}H), 7.30 (d, $^3J_{H,H}$ = 8.1 Hz, 4 H; C_{ar}H), 7.22—7.12 (m, 6 H; C_{ar}H), 7.06

(dd, $^{3}J_{H,H}$ = 7.6 Hz, $^{4}J_{H,H}$ = 1.7 Hz, 4 H; $C_{ar}H$), 5.15 (m, 4 H; CO_2CH_2), 2.46–2.40 (m, 2 H; CH_2), 2.44 (s, 6 H; $C_{ar}CH_3$), 1.47 ppm (s, 6 H; CH_3). ^{13}C NMR (100 MHz, $CDCl_3$): δ = 165.3 (CO), 154.7 (C_{azo}), 153.7 (C_{azo}), 152.6 (C_{ar}), 150.9 (C_{ar}), 141.9 (C_{azo}), 138.0 (C_{azo}), 135.6 (C_{ar}), 129.9 (C_{azo}), 129.1 (C_{azo}), 128.7 (C_{ar}), 128.0 (C_{ar}), 127.0 (C_{ar}), 123.1 (C_{azo}), 123.0 (C_{azo}), 80.1 (CH_2), 66.4 ($CO_2\mathit{C}H_2$), 61.5 (C), 21.7 ($C_{ar}\mathit{C}H_3$), 15.6 ppm (CH_3). IR (ATR): ṽ = 2359, 1709, 1288, 1219, 1155, 1080, 1013, 824, 698 cm^{-1}. UV/Vis (CH_3CN): λ_{max} (log ε) = 233 (4.69), 325 nm (4.59). HRMS (ESI-TOF) m/z: $[C_{51}H_{44}N_4O_4+H]^+$ calculated: 777.3435; found: 777.3443.

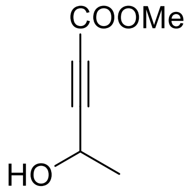

20

Synthesis of alcohol 20. In a dried Schlenk flask, methyl propiolate (0.8 mL, 9.0 mmol) and 1-methylimidazole (0.03 mL, 0.375 mmol) were dissolved in dry DCM (45 mL) and cooled to 0 °C. Subsequently, a diethylzinc solution (1.0 M in hexanes; 9.0 mL, 9.0 mmol) was added to the mixture and stirred for 3 hours at a room temperature. Acetaldehyde (0.4 mL, 7.5 mmol) was then added to the reaction mixture and stirred overnight. Afterwards, an aqueous NH_4Cl solution was added (20 mL) to terminate the reaction. The organic phase was extracted with DCM (3×20 mL). The combined organic phases were combined, washed with brine (20 mL), dried over $MgSO_4$ and concentrated in vacuo. The crude product was purified by column chromatography (hexanes/EtOAc = 9:1) to provide alcohol **20** as a yellow liquid (318 mg, 2.5 mmol, 33%).

^{1}H NMR (400 MHz, $CDCl_3$): δ = 4.64 (q, $^{3}J_{H,H}$ = 6.7 Hz, 1 H; CH), 3.79 ppm (s, 3 H; CO_2CH_3), 1.52 ppm (d, $^{3}J_{H,H}$ = 6.7 Hz, 3 H; CH_3). ^{13}C NMR (100 MHz, $CDCl_3$): δ = 153.9 (CO), 88.8 (C_{alkyne}), 75.7 (C_{alkyne}), 58.2 (CH), 53.0 ($CO_2\mathit{C}H_3$), 23.4 ppm (CH_3).

The spectroscopic data are consistent with the literature.[6]

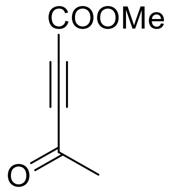

22

Synthesis of ketone 22. To a solution of the alcohol **20** (304 mg, 2.37 mmol) Dess-Martin periodinane (DMP) (1.41 g, 3.32 mmol) was added, and the mixture was stirred at a room temperature for 12 hours. Afterwards, another portion of DMP (703 mg, 1.66 mmol) was added and stirred for another 6 hours. The reaction was then terminated by the addition of aqueous, saturated $NaHCO_3$ solution (20 mL) to the reaction, after which a white precipitate was filtered off. The filtrate was extracted with DCM (3×20 mL). The organic phases were combined, washed with saturated NH_4Cl solution (20 mL), dried over Na_2SO_4 and concentrated in vacuo. The crude product was purified using a silica plug (hexanes/EtOAc = 9:1) to provide ketone **22** as a yellow liquid (210 mg, 1.66 mmol, 70%).

^{1}H NMR (400 MHz, $CDCl_3$): δ = 3.85 ppm (s, 3 H; CO_2CH_3), 2.43 ppm (s, 3 H; CH_3). ^{13}C NMR (100 MHz, $CDCl_3$): δ = 182.6 (CO) 152.8 (CO_2), 81.2 (C_{alkyne}), 53.6 ($CO_2\textit{C}H_3$), 32.5 ppm (CH_3). The spectroscopic data are consistent with the literature.[7]

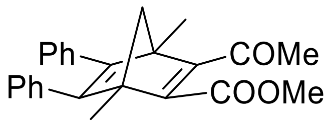

7-NBD

Synthesis of 7-NBD. A solution containing cyclopentadiene **26** (448 mg, 1.82 mmol) and alkyne **23** (152 mg, 1.30 mmol) was prepared by dissolving the reagents in benzene (10 mL). The solution was heated to reflux for 4 hours. Afterwards, the solvent was removed in vacuo. The residue was purified by chromatography on silica gel (solvent: hexanes/EtOAc = 10:1) to yield **7**-NBD as a yellow solid (368 mg, 0.99 mmol, 76%).

M.p.: 94 °C. ^{1}H NMR (400 MHz, $CDCl_3$): δ = 7.24–7.12 (m, 6 H; $C_{ar}H$), 7.06 (d, $^3J_{H,H}$ = 7.7 Hz, 4 H; $C_{ar}H$), 3.78 (s, 3 H; CO_2CH_3), 2.39 (d, $^2J_{H,H}$ = 6.8 Hz, 1 H; CH_2), 2.35 (d, $^2J_{H,H}$ = 6.8 Hz, 1 H; CH_2), 2.28 (s, 3 H; $COCH_3$), 1.55 (s, 3 H; CH_3), 1.38 ppm (s, 3 H; CH_3). ^{13}C NMR (100 MHz, $CDCl_3$): δ = 202.1 (CO) 167.8 (CO_2), 154.7 (C_{alkene}), 152.8 (C_{alkene}), 147.7 (C_{alkene}), 135.8 (C_{ar}), 135.5 (C_{ar}), 129.1 (C_{ar}), 128.6 (C_{ar}), 128.1 (C_{ar}), 128.0 (C_{ar}), 127.0 (C_{ar}), 126.9 (C_{ar}), 79.9 (CH_2),

62.1 (C), 60.6 (C), 51.9 ($CO_2\mathit{C}H_3$), 31.0 ($CO\mathit{C}H_3$), 16.1 (CH_3), 15.5 ppm (CH_3). IR (ATR): $\tilde{\nu}$ = 2928, 1726, 1663, 1292, 1225, 698 cm^{-1}. UV/Vis (CH_3CN): λ_{max} (log ε) = 249 nm (4.31). HRMS (ESI-TOF) m/z: $[C_{25}H_{24}O_3+H]^+$ calculated: 373.1798; found: 373.1802; $[C_{25}H_{24}O_3+Na]^+$ calculated: 395.1618; found: 395.1613.

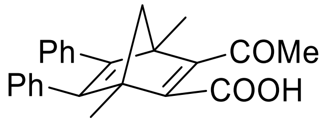

27-NBD

Synthesis of 27-NBD. Compound **7**-NBD (216 mg, 0.58 mmol) was dissolved in MeOH (5 mL), mixed with a 1 M NaOH (3 mL, 3.00 mmol) and heated to reflux overnight. Subsequently, the reaction mixture was acidified with 2 M HCl to pH = 1. The aqueous layer was extracted with diethyl ether (3×20 mL). The combined organic layers were washed with water (20 mL), dried over Na_2SO_4, and the solvent was removed in vacuo to afford the desired compound **27**-NBD as a pale yellow solid (196 mg, 0.55 mmol, 94%), which was used in the next step without further purification.

M.p.: 158 °C. ^{1}H NMR (400 MHz, $CDCl_3$): δ = 7.23–7.09 (m, 8 H; $C_{ar}H$), 7.04–6.99 (m, 2 H; $C_{ar}H$), 2.40 (br. s, 2 H; CH_2), 2.25 (s, 3 H; $COCH_3$), 1.60 (s, 3 H; CH_3), 1.44 ppm (s, 3 H; CH_3). ^{13}C NMR (100 MHz, $CDCl_3$): δ = 206.3 (CO), 185.1 (CO_2), 155.3 (C_{alkene}), 151.9 (C_{alkene}), 147.3 (C_{alkene}), 135.67 (C_{ar}), 135.2 (C_{ar}), 130.3 (C_{ar}), 129.2 (C_{ar}), 128.6 (C_{ar}), 128.3 (C_{ar}), 128.1 (C_{ar}), 127.2 (C_{ar}), 127.0 (C_{ar}), 79.9 (CH_2), 62.3 (C), 60.7 (C), 30.9 ($CO\mathit{C}H_3$), 16.2 (CH_3), 15.8 ppm (CH_3). IR (ATR): $\tilde{\nu}$ = 2924, 2583, 2359, 1595, 1495, 1481, 1443, 1371, 1331, 1302, 1254, 1221, 1182, 1155, 1101, 1070, 1030, 1011, 968, 934, 847, 770, 762, 727, 687, 621 cm^{-1}. UV/Vis (CH_3CN): λ_{max} (log ε) = 235 nm (3.89). HRMS (ESI-TOF) m/z: $[C_{24}H_{22}O_3+H]^+$ calculated: 359.1642; found: 359.1646; $[C_{24}H_{22}O_3+Na]^+$ calculated: 381.1461; found: 381.1466.

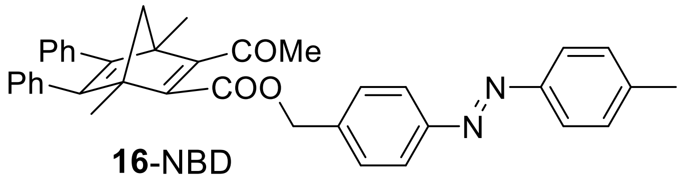

16-NBD

Synthesis of 16-NBD. Compound **27**-NBD (164 mg, 0.46 mmol) and azobenzene **32** (120 mg, 0.42 mmol) were treated according to general procedure *A*. The crude product was purified by column chromatography (hexanes/EtOAc = 6:1) to provide **16**-NBD as a red oil (182 mg, 0.32 mmol, 70%). ^{1}H NMR (400 MHz, $CDCl_3$): δ = 7.85 (dd, $^3J_{H,H}$ = 16.7 Hz, 8.3 Hz, 4 H; C_{azo}H), 7.44 (d, $^3J_{H,H}$ = 8.4 Hz, 2 H; C_{azo}H), 7.32 (d, $^3J_{H,H}$ = 8.1 Hz, 2 H; C_{azo}H), 7.17 (m, 6 H; C_{ar}H), 7.09–7.02 (m, 4 H; C_{ar}H), 5.27 (m, 2 H; CO_2CH_2), 2.44 (s, 3 H; $C_{ar}CH_3$), 2.40 (d, $^2J_{H,H}$ = 6.8 Hz, 1 H; CH_2), 2.36 (d, $^2J_{H,H}$ = 6.4 Hz, 1 H; CH_2), 2.21 (s, 3 H; $COCH_3$), 1.55 (s, 3 H; CH_3), 1.38 ppm (s, 3 H; CH_3). ^{13}C NMR (100 MHz, $CDCl_3$): δ = 202.1 (CO), 168.6 (C_{alkene}), 164.7 (CO_2), 154.6 (C_{alkene}), 152.9 (C_{alkene}), 152.7 (C_{azo}), 150.9 (C_{azo}), 147.4 (C_{alkene}), 142.0 (C_{azo}), 137.8 (C_{azo}), 135.7 (C_{ar}), 135.5 (C_{ar}), 129.9 (C_{azo}), 129.3 (C_{azo}), 129.0 (C_{ar}), 128.6 (C_{ar}), 128.1 (C_{ar}), 128.0 (C_{ar}), 127.0 (C_{ar}), 126.9 (C_{ar}), 123.1 (C_{azo}), 121.1 (C_{ar}), 120.6 (C_{ar}), 79.8 (CH_2), 66.5 (OCH_2), 62.2 (C), 60.6 (C), 31.2 ($COCH_3$), 21.7 ($C_{ar}CH_3$), 16.2 (CH_3), 15.4 ppm (CH_3). IR (ATR): ṽ = 2961, 2928, 1694, 1601, 1497, 1450, 1418, 1383, 1371, 1352, 1288, 1240, 1209, 1157, 1074, 1047, 1030, 1013, 920, 829, 789, 770, 746, 721, 701, 642, 627 cm^{-1}. UV/Vis (CH_3CN): λ_{max} (log ε) = 231 (4.43), 328 nm (4.37). HRMS (ESI-TOF) m/z: $[C_{38}H_{34}N_2O_3+H]^+$ calculated: 567.2642; found: 567.2645; $[C_{38}H_{34}N_2O_3+Na]^+$ calculated: 589.2462; found: 589.2466.

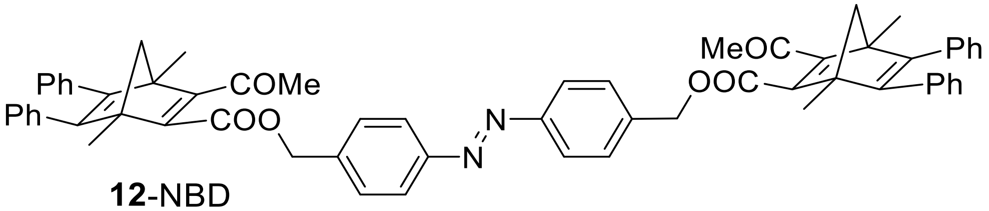

12-NBD

Synthesis of 12-NBD. Compound **27**-NBD (88 mg, 0.25 mmol) and azobenzene **29** (41 mg, 0.11 mmol) were treated according to general procedure *B*. The crude product was purified by column chromatography (hexanes/EtOAc = 6:1) to provide compound **12**-NBD as an orange solid (39 mg, 0.04 mmol, 38%).

M.p.: 90 °C. ^{1}H NMR (400 MHz, $CDCl_3$): δ = 7.90 (d, $^3J_{H,H}$ = 8.4 Hz, 4 H; C_{azo}H), 7.45 (d, $^3J_{H,H}$ = 8.5 Hz, 4 H; C_{azo}H), 7.28–7.12 (m, 12 H; C_{ar}H), 7.09–7.00 (m, 8 H; C_{ar}H), 5.28 (m, 4 H; CO_2CH_2), 2.41 (d, $^2J_{H,H}$ = 6.8 Hz, 1 H; CH_2), 2.36 (d, $^2J_{H,H}$ = 6.8 Hz, 1 H; CH_2), 2.21 (s, 6 H; $COCH_3$), 1.55 (s, 6 H; CCH_3), 1.38 ppm (s, 6 H; CCH_3). ^{13}C NMR (100 MHz, $CDCl_3$): δ = 202.2 (CO), 168.7 (C_{alkene}), 164.7 (CO_2), 154.6 (C_{alkene}), 152.9 (C_{alkene}), 152.5 (C_{azo}), 147.3 (C_{alkene}), 138.4 (C_{azo}), 135.6 (C_{ar}), 135.5 (C_{ar}), 129.3 (C_{azo}), 129.0 (C_{ar}), 128.6 (C_{ar}), 128.1 (C_{ar}), 128.0 (C_{ar}),

127.0 (C_{ar}), 126.9 (C_{ar}), 123.3 (C_{azo}), 79.8 (CH_2), 66.4 (OCH_2), 62.2 (C), 60.6 (C), 31.2 (CO*C*H_3), 16.2 (CH_3), 15.4 ppm (CH_3). IR (ATR): ṽ = 2928, 1694, 1601, 1447, 1383, 1352, 1288, 1207, 1163, 1136, 1115, 1072, 1047, 1028, 1011, 839, 791, 745, 719, 698 cm^{-1}. UV/Vis (CH_3CN): λ_{max} (log ε) = 230 (4.58), 324 nm (4.45). HRMS (ESI-TOF) m/z: $[C_{62}H_{54}N_2O_6+H]^+$ calculated: 923.4055; found: 923.4051; $[C_{62}H_{54}N_2O_6+Na]^+$ calculated: 945.3874; found: 945.3870.

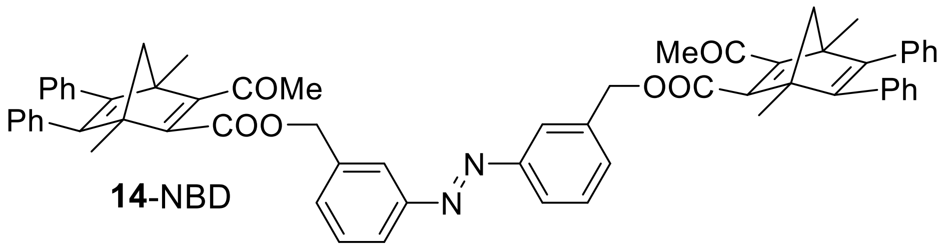

14-NBD

Synthesis of 14-NBD. Compound **27**-NBD (44 mg, 0.12 mmol) and azobenzene **31** (21 mg, 0.06 mmol) were treated according to general procedure *B*. The crude product was purified by column chromatography (hexanes/EtOAc = 6:1) to provide compound **14**-NBD as an orange solid (22 mg, 0.02 mmol, 43%).

M.p.: 87 °C. ^{1}H NMR (400 MHz, $CDCl_3$): δ = 7.90–7.86 (m, 4 H; C_{azo}H), 7.50 (m, 2 H; C_{azo}H), 7.43 (m, 2 H; C_{azo}H), 7.21–7.09 (m, 12 H; C_{ar}H), 7.05 (m, 8 H; C_{ar}H), 5.31 (m, 4 H; CO_2CH_2), 2.40 (d, $^2J_{H,H}$ = 6.8 Hz, 1 H; CH_2), 2.36 (d, $^2J_{H,H}$ = 6.8 Hz, 1 H; CH_2), 2.20 (s, 6 H; $COCH_3$), 1.55 (s, 6 H; CH_3), 1.37 ppm (s, 6 H; CH_3). ^{13}C NMR (100 MHz, $CDCl_3$): δ = 202.08 (CO), 168.58 (C_{alkene}), 164.71 (CO_2), 154.64 (C_{alkene}), 152.84 (C_{alkene}), 152.77 (C_{azo}), 147.42 (C_{alkene}), 136.57 (C_{azo}), 135.68 (C_{ar}), 135.48 (C_{ar}), 131.19 (C_{azo}), 129.64 (C_{azo}), 129.05 (C_{ar}), 128.58 (C_{ar}), 128.12 (C_{ar}), 128.02 (C_{ar}), 127.02 (C_{ar}), 126.89 (C_{ar}), 123.55 (C_{azo}), 122.56 (C_{azo}), 79.80 (CH_2), 66.52 (OCH_2), 62.22 (C), 60.64 (C), 31.19 (CO*C*H_3), 16.20 (CH_3), 15.40 ppm (CH_3). IR (ATR): ṽ = 2928, 1694, 1601, 1447, 1383, 1352, 1288, 1207, 1163, 1136, 1115, 1072, 1047, 1028, 1011, 839, 791, 745, 719, 698 cm^{-1}. UV/Vis (CH_3CN): λ_{max} (log ε) = 233 (4.57), 317 nm (4.27). HRMS (ESI-TOF) m/z: $[C_{62}H_{54}N_2O_6+H]^+$ calculated: 923.4055; found: 923.4057; $[C_{62}H_{54}N_2O_6+Na]^+$ calculated: 945.3874; found: 945.3877.

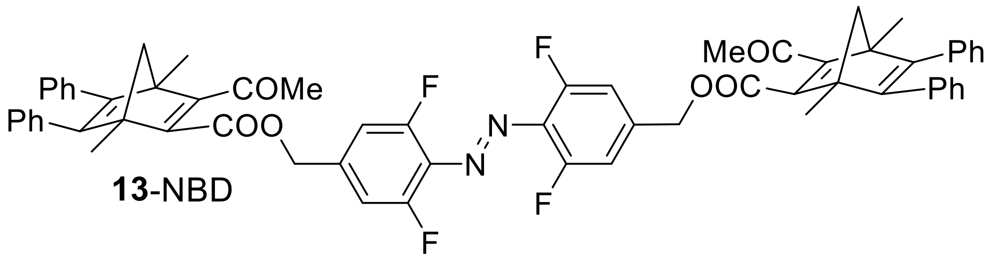

13-NBD

Synthesis of 13-NBD. Compound **27**-NBD (50 mg, 0.14 mmol) and azobenzene **30** (28 mg, 0.06 mmol) were treated according to general procedure *B*. The crude product was purified by column chromatography (hexanes/EtOAc = 3:1) to provide compound **13**-NBD as a red solid (22 mg, 0.02 mmol, 35%).

M.p.: 135 °C. ^{1}H NMR (400 MHz, $CDCl_3$): δ = 7.26–7.12 (m, 13 H; $C_{ar}H$), 7.11–6.93 (m, 11 H; $C_{ar}H$), 5.20 (m, 4 H; CO_2CH_2), 2.43 (d, $^{2}J_{H,H}$ = 6.9 Hz, 1 H; CH_2), 2.39 (d, $^{2}J_{H,H}$ = 6.9 Hz, 2 H; CH_2), 2.27 (s, 6 H; $COCH_3$), 1.57 (s, 6 H; CH_3), 1.40 ppm (s, 6 H; CH_3). ^{13}C NMR (100 MHz, $CDCl_3$): δ = 202.3 (CO), 170.0 (C_{alkene}), 164.2 (CO_2), 154.5 (C_{alkene}), 152.8 (C_{azo}), 146.4 (C_{alkene}),138.6 (C_{azo}) 135.4 (C_{ar}), 129.0 (C_{ar}), 129.0 (C_{ar}), 128.5 (C_{ar}), 128.2 (C_{ar}), 128.1 (C_{ar}), 127.1 (C_{ar}), 112.4–111.4 (m; C_{azo}), 79.8 (CH_2), 64.9 (OCH_2), 62.4 (C), 60.6 (C), 31.2 ($COCH_3$), 16.2 (CH_3), 15.3 (CH_3). IR (ATR): ṽ = 2963, 2930, 2872, 1694, 1628, 1601, 1574, 1497, 1441, 1385, 1352, 1329, 1288, 1206, 1165, 1115, 1074, 1047, 1032, 984, 908, 849, 791, 727, 648, 627 cm^{-1}. UV/Vis (CH_3CN): λ_{max} (log ε) = 234 (4.18), 312 nm (3.82). HRMS (ESI-TOF) m/z: $[C_{62}H_{50}N_2O_6F_2+Na]^+$ calculated: 1017.3497; found: 1017.3499.

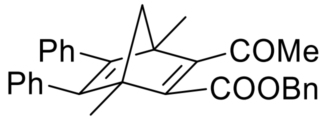

10-NBD

Synthesis of 10-NBD. Compound **27**-NBD (88 mg, 0.25 mmol) and benzyl bromide (38 mg, 0.22 mmol) were treated according to general procedure *A*. The crude product was purified by column chromatography (hexanes/EtOAc = 9:1) to provide **10**-NBD as a yellow solid (88 mg, 0.20 mmol, 88%).

M.p.: 87 °C. ^{1}H NMR (400 MHz, $CDCl_3$): δ = 7.33 (tdd, $^{3}J_{H,H}$ = 7.1, 4.9 Hz, $^{4}J_{H,H}$ = 1.8 Hz, 5 H; $C_{ar}H$), 7.21–7.13 (m, 6 H; $C_{ar}H$), 7.07–7.01 (m, 4 H; $C_{ar}H$), 5.26–5.18 (m, 2 H; CO_2CH_2), 2.60 (d, $^{2}J_{H,H}$ = 6.9 Hz, 1 H; CH_2), 2.54 (d, $^{2}J_{H,H}$ = 6.8 Hz, 1 H; CH_2), 2.17 (s, 3 H; $COCH_3$), 1.53 (s, 3 H;

CH_3), 1.37 ppm (s, 3 H; CH_3). ^{13}C NMR (100 MHz, $CDCl_3$): δ = 202.0 (CO), 167.9 (C_{alkene}), 164.8 (CO_2), 154.6 (C_{alkene}), 152.9 (C_{alkene}), 147.9 (C_{alkene}), 135.7 (C_{ar}), 135.5 (C_{ar}), 135.3 (C_{ar}), 129.0 (C_{ar}), 128.8 (C_{ar}), 128.7 (C_{ar}), 128.6 (C_{ar}), 128.6 (C_{ar}), 128.1 (C_{ar}), 128.0 (C_{ar}), 127.0 (C_{ar}), 126.9 (C_{ar}), 79.8 (CH_2), 67.0 (OCH_2), 62.1 (C), 60.6 (C), 31.1 (CO*C*H_3), 16.1 (CH_3), 15.4 ppm (CH_3). IR (ATR): ṽ = 2953, 2930, 2859, 1709, 1692, 1605, 1497, 1449, 1383, 1354, 1300, 1213, 1113, 1076, 1047, 1028, 924, 810, 791, 772, 743, 727, 631 cm^{-1}. UV/Vis (CH_3CN): λ_{max} (log ε) = 244 nm (4.47). HRMS (ESI-TOF) m/z: $[C_{31}H_{28}O_3+H]^+$ calculated: 471.1931; found: 471.1932; $[C_{31}H_{28}O_3+Na]^+$ calculated: 449.2111; found: 449.2112.

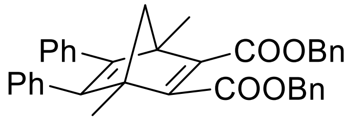

11-NBD

Synthesis of 11-NBD. Compound **28**-NBD (92 mg, 0.26 mmol) and benzyl bromide (79 mg, 0.46 mmol) were treated according to general procedure *C*. The crude product was purified by column chromatography (hexanes/EtOAc = 9:1) to provide **11**-NBD as a yellow oil (75 mg, 0.14 mmol, 54%).

^{1}H NMR (400 MHz, $CDCl_3$): δ = 7.33–7.28 (m, 6 H; $C_{ar}H$), 7.24 (dd, $^3J_{H,H}$ = 6.8 Hz, $^4J_{H,H}$ = 3.0 Hz, 4 H; $C_{ar}H$), 7.13 (dd, $^3J_{H,H}$ = 5.2 Hz, $^4J_{H,H}$ = 1.9 Hz, 5 H; $C_{ar}H$), 7.08–7.0 (m, 5 H; $C_{ar}H$), 5.05 (d, $^2J_{H,H}$ = 5.7 Hz, 4 H; OCH_2), 2.43 (d, $^2J_{H,H}$ = 6.8 Hz, 1 H; CH_2), 2.36 (d, $^2J_{H,H}$ = 6.8 Hz, 1 H; CH_2), 1.43 (s, 6 H; CH_3). ^{13}C NMR (100 MHz, $CDCl_3$): δ = 165.4 (CO_2), 154.6 (C_{alkene}), 153.7 (C_{alkene}), 135.6 (C_{ar}), 135.5 (C_{ar}), 130.5 (C_{ar}), 128.7 (C_{ar}), 128.7 (C_{ar}), 128.5 (C_{ar}), 128.4 (C_{ar}), 128.4 (C_{ar}), 128.0 (C_{ar}), 128.0 (C_{ar}), 126.9 (C_{ar}), 80.1 (CH_2), 66.9 (OCH_2), 61.4 (C), 15.5 ppm (CH_3). IR (ATR): ṽ = 2961, 2928, 1709, 1607, 1497, 1383, 1287, 1221, 1153, 1119, 1078, 1047, 1020, 1003, 957, 908, 847, 822, 795, 750, 635 cm^{-1}. UV/Vis (CH_3CN): λ_{max} (log ε) = 245 nm (4.17). HRMS (ESI-TOF) m/z: $[C_{37}H_{32}O_4+H]^+$ calculated: 541.2373; found: 541.2373; $[C_{37}H_{32}O_4+Na]^+$ calculated: 563.2193; found: 563.2192.

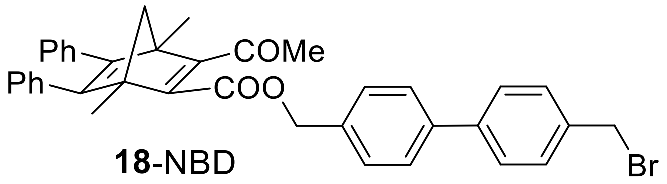

18-NBD

Synthesis of 18-NBD. Compound **27**-NBD (120 mg, 0.34 mmol) and diphenyl dibromide **33** (104 mg, 0.31 mmol) were treated according to general procedure *A*. The crude product was purified by column chromatography (hexanes/EtOAc = 6:1) to provide **18**-NBD as a pale yellow oil (70 mg, 0.11 mmol, 34%).

^{1}H NMR (400 MHz, $CDCl_3$): δ = 7.56 (dd, $^3J_{H,H}$ = 8.4, $^4J_{H,H}$ = 2.1 Hz, 4 H; C_{ar}H), 7.47 (d, $^3J_{H,H}$ = 8.4 Hz, 2 H; C_{ar}H), 7.38 (d, $^3J_{H,H}$ = 8.3 Hz, 2 H; C_{ar}H), 7.18 (m, 6 H; C_{ar}H), 7.08–7.00 (m, 4 H; C_{ar}H), 5.25 (m, 2 H; OCH_2), 4.55 (s, 2 H; CH_2Br), 2.40 (d, $^2J_{H,H}$ = 6.8 Hz, 1 H; CH_2), 2.36 (d, $^2J_{H,H}$ = 6.9 Hz, 1 H; CH_2), 2.21 (s, 3 H; CH_3), 1.54 (s, 3 H; CH_3), 1.38 ppm (s, 3 H; CH_3). ^{13}C NMR (100 MHz, $CDCl_3$): δ = 202.1 (CO), 168.1 (C_{alkene}), 164.8 (CO_2), 154.6 (C_{alkene}), 152.9 (C_{alkene}), 147.8 (C_{alkene}), 140.9 (C_{ar}), 140.7 (C_{ar}), 137.2 (C_{ar}), 135.7 (C_{ar}), 135.5 (C_{ar}), 134.7 (C_{ar}), 129.7 (C_{ar}), 129.2 (C_{ar}), 129.0 (C_{ar}), 128.64 (C_{ar}), 128.1 (C_{ar}), 128.0 (C_{ar}), 127.7 (C_{ar}), 127.7 (C_{ar}), 127.4 (C_{ar}), 127.0 (C_{ar}), 126.9 (C_{ar}), 79.8 (CH_2), 66.7 (OCH_2), 62.2 (C), 60.6 (C), 33.4 (CH_2Br), 31.2 ($COCH_3$), 16.2 (CH_3), 15.4 ppm (CH_3). IR (ATR): ṽ = 2963, 2928, 2359, 2162, 1694, 1601 1574, 1499, 1400, 1383, 1371, 1352, 1288, 1209, 1165, 1115, 1074, 1047, 1030, 1005, 941, 920, 897, 810, 772, 746, 721, 646 cm^{-1}. UV/Vis (CH_3CN): λ_{max} (log ε) = 263 nm (4.50). HRMS (ESI-TOF) m/z: $[C_{38}H_{33}O_3Br+H]^+$ calculated:619.1673; found: 619.1677; $[C_{38}H_{33}O_3Br+Na]^+$ calculated: 641.1492; found: 641.1494.

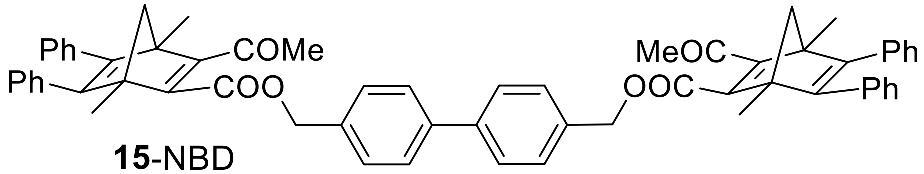

15-NBD

Synthesis of 15-NBD. Compound **27**-NBD (120 mg, 0.34 mmol) and diphenyl dibromide **33** (104 mg, 0.31 mmol) were treated according to general procedure *B*. The crude product was purified by column chromatography (hexanes/EtOAc = 6:1) to provide **15**-NBD as a colorless solid (42 mg, 0.05 mmol, 28%).

M.p.: 81 °C. ^{1}H NMR (400 MHz, $CDCl_3$): δ = 7.57 (d, $^3J_{H,H}$ = 8.3 Hz, 4 H; C_{ar}H), 7.39 (d, $^3J_{H,H}$ = 8.3 Hz, 2 H; C_{ar}H), 7.23–7.11 (m, 12 H; C_{ar}H), 7.09–7.00 (m, 8 H; C_{ar}H), 5.26 (m, 4 H; OCH_2),

2.40 (d, $^{2}J_{H,H}$ = 6.8 Hz, 1 H; CH_2), 2.35 (d, $^{2}J_{H,H}$ = 6.8 Hz, 1 H; CH_2), 2.21 (s, 6 H; $COCH_3$), 1.55 (s, 6 H; CCH_3), 1.38 ppm (s, 6 H; CCH_3). ^{13}C NMR (100 MHz, $CDCl_3$): δ = 202.1 (CO), 168.1 (C_{alkene}), 164.8 (CO_2), 154.6 (C_{alkene}), 152.9 (C_{alkene}), 147.8 (C_{alkene}), 140.8 (C_{ar}), 135.7 (C_{ar}), 135.5 (C_{ar}), 134.6 (C_{ar}), 129.2 (C_{ar}), 129.0 (C_{ar}), 128.6 (C_{ar}), 128.1 (C_{ar}), 128.0 (C_{ar}), 127.4 (C_{ar}), 127.0 (C_{ar}), 126.9 (C_{ar}), 79.8 (CH_2), 66.7 (OCH_2), 62.12 (C), 60.7 (C), 31.2 ($COCH_3$), 16.2 (CH_3), 15.4 ppm (CH_3). IR (ATR): ṽ = 2949, 1694, 1601, 1447, 1383, 1352, 1288, 1209, 1165, 1138, 1115, 1072, 1047, 1028, 1005, 910, 804, 723 cm^{-1}. UV/Vis (CH_3CN): λ_{max} (log ε) = 255 nm (4.43). HRMS (ESI-TOF) m/z: $[C_{62}H_{54}O_6+Na]^+$ calculated: 917.3817; found: 917.3813.

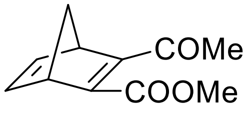

5-NBD

Synthesis of 5-NBD. Alkyne **22** (300 mg, 2.38 mmol) was dissolved in chlorobenzene (3 mL) with dibutylhydroxytoluene (2 mg) and cyclopentadiene (**24**) (0.2 mL, 2.38 mmol) in a vessel suitable for microwave reaction. The vial was heated to 110 °C for 7 hours in a microwave (150 W). Fresh cyclopentadiene was added every hour (0.2 mL, 2.38 mmol) each time. Afterwards, the solvent was removed in vacuo and the residue was purified by column chromatography (hexanes/EtOAc = 9:1) to afford **5**-NBD as a pale-yellow oil (293 mg, 1.52 mmol, 64%).

^{1}H NMR (400 MHz, $CDCl_3$): δ = 6.93–6.86 (m, 2 H; $C_{alkene}H$), 3.98 (m, 1 H; CH), 3.92 (m, 1 H; CH), 3.79 (s, 3 H; CO_2CH_3), 2.38 (s, 3 H; CH_3) 2.20 (dt, $^{2}J_{H,H}$ = 6.9 Hz, $^{3}J_{H,H}$ = 1.6 Hz, 1 H; CH_2), 2.05 ppm (dt, $^{2}J_{H,H}$ = 6.9 Hz, $^{3}J_{H,H}$ = 1.5 Hz, 1 H; CH_2). ^{13}C NMR (100 MHz, $CDCl_3$): δ = 199.4 (CO), 165.4 (CO_2), 162.4 (C_{alkene}), 149.1 (C_{alkene}), 142.8 (C_{alkene}), 142.3 (C_{alkene}), 72.1 (CH_2), 54.1 (C), 53.7 (C), 52.2 (CO_2CH_3) 29.8 ppm ($COCH_3$). IR (ATR): ṽ = 2949, 1713, 1663, 1609, 1558, 1435, 1358, 1312, 1290, 1246, 1198, 1150, 1099, 1086, 1043, 1020, 989, 951, 916, 878, 868, 822, 781, 719, 667 cm^{-1}. UV/Vis (CH_3CN): λ_{max} (log ε) = 245 nm (4.35). HRMS (ESI-TOF) m/z: $[C_{11}H_{22}O_3+H]^+$ calculated: 193.0858; found: 193.0859; $[C_{11}H_{22}O_3+Na]^+$ calculated: 215.0678; found: 215.0679.

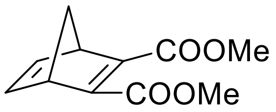

6-NBD

Synthesis of 6-NBD. Alkyne **25** (314 mg, 2.20 mmol) was dissolved in chlorobenzene (3 mL) with dibutylhydroxytoluene (2 mg) and cyclopentadiene (**24**) (0.18 mL, 2.20 mmol) in a vessel suitable for microwave reaction. The vial was heated to 110 °C for 7 hours in a microwave (150 W). Fresh cyclopentadiene was added every hour (0.2 mL, 2.38 mmol) each time. Afterwards, the solvent was removed in vacuo and the residue was purified by column chromatography (hexanes/EtOAc = 9:1) to afford **6**-NBD as a pale-yellow oil (365 mg, 1.75 mmol, 80%).

^{1}H NMR (400 MHz, $CDCl_3$): δ = 7.01–6.83 (m, 2 H; $C_{alkene}H$), 3.94 (dt, $^{2}J_{H,H}$ = 3.5 Hz, $^{3}J_{H,H}$ = 1.6 Hz, 2 H; CH), 3.78 (s, 6 H; CO_2CH_3), 2.28 (dt, $^{2}J_{H,H}$ = 6.8 Hz, $^{3}J_{H,H}$ = 1.5 Hz, 1 H; CH_2), 2.10 ppm (dt, $^{2}J_{H,H}$ = 6.8 Hz, $^{3}J_{H,H}$ = 1.5 Hz, 1 H; CH_2). ^{13}C NMR (100 MHz, $CDCl_3$): δ = 165.6 (CO), 152.6 (C_{alkene}), 142.6 (C_{alkene}), 73.1 (CH_2), 53.6 (C), 52.2 ppm ($CO_2\mathit{C}H_3$).

The spectroscopic data are consistent with the literature.[8]

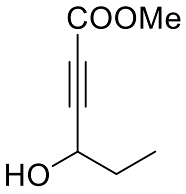

21

Synthesis of alcohol 21. In a dried Schlenk flask, methyl propiolate (2.0 mL, 22.0 mmol) and 1-methylimidazole (0.08 mL, 0.925 mmol) were dissolved in dry DCM (110 mL) and cooled to 0 °C. Subsequently, a diethylzinc solution (1.0 M in hexanes; 22.0 mL, 22.0 mmol) was added to the mixture and stirred for 3 hours at a room temperature. Propionaldehyde (1.33 mL, 18.5 mmol) was then added to the reaction mixture and stirred overnight. Afterwards, an aqueous NH_4Cl solution was added (20 mL) to terminate the reaction. The organic phase was extracted with DCM (3×20 mL). The combined organic phases were combined, washed with brine (20 mL), dried over $MgSO_4$ and concentrated in vacuo. The crude product was purified by column chromatography (hexanes/EtOAc = 6:1) to provide alcohol **21** as a yellow liquid (790 mg, 5.57 mmol, 30%).

^{1}H NMR (400 MHz, $CDCl_3$): δ = 4.45 (t, $^{3}J_{H,H}$ = 6.5 Hz, 1 H; CH), 3.79 ppm (s, 3 H; CO_2CH_3), 1.86–1.75 (m, 2 H; CH_2), 1.04 ppm (t, $^{3}J_{H,H}$ = 7.4 Hz, 3 H; CH_3). ^{13}C NMR (100 MHz, $CDCl_3$): δ = 153.9 (CO), 88.1 (C_{alkyne}), 76.5 (C_{alkyne}), 63.5 (CH), 53.0 ($CO_2\textit{C}H_3$), 30.2 (CH_2), 9.4 ppm (CH_3). The spectroscopic data are consistent with the literature.[6]

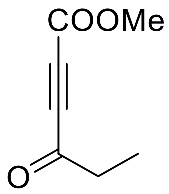

23

Synthesis of ketone 23. To a solution of the alcohol **21** (730 mg, 5.12 mmol) Dess-Martin periodinane (DMP) (2,910 mg, 6.86 mmol) was added and the mixture was stirred at a room temperature for 12 hours. Afterwards, another portion of DMP (1450 mg, 3.43 mmol) was added and stirred for another 6 hours. The reaction was then terminated by the addition of aqueous, saturated $NaHCO_3$ solution (30 mL) to the reaction, after which a white precipitate was filtered off. The filtrate was extracted with DCM (3×20 mL). The combined organic phases were combined, washed with aq., saturated NH_4Cl solution (20 mL), dried over Na_2SO_4 and concentrated in vacuo. The crude product was purified using a silica plug (hexanes/EtOAc = 3:1) to provide ketone **23** as a yellow liquid (557 mg, 3.97 mmol, 78%).[5]

^{1}H NMR (400 MHz, $CDCl_3$): δ = 3.85 ppm (s, 3 H; CO_2CH_3), 2.67 (q, $^{3}J_{H,H}$ = 7.3 Hz, 2 H; CH_2), 1.17 ppm (t, $^{3}J_{H,H}$ = 7.3 Hz, 3 H; CH_3). ^{13}C NMR (100 MHz, $CDCl_3$): δ = 186.5 (CO) 152.8 (CO_2), 80.9 (C_{alkyne}), 78.0 (C_{alkyne}), 53.5 ($CO_2\textit{C}H_3$), 38.9 (CH_2), 7.6 ppm (CH_3).

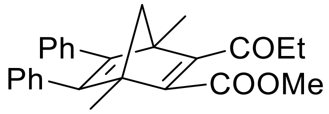

8-NBD

Synthesis of 8-NBD. A solution containing cyclopentadiene **26** (224 mg, 0.91 mmol) and alkyne **23** (91 mg, 0.65 mmol) was prepared by dissolving the reagents in benzene (12 mL). The solution was heated to reflux for 4 hours. Afterwards, the solvent was removed in vacuo. The residue was

purified by chromatography on silica gel (hexanes/EtOAc = 10:1) to yield **8**-NBD as a yellow solid (201 mg, 0.52 mmol, 80%).

^{1}H NMR (400 MHz, $CDCl_3$): δ = 7.24–7.13 (m, 6 H; $C_{ar}H$), 7.06 (ddd, $^3J_{H,H}$ = 8.0, 3.9 Hz, $^4J_{H,H}$ = 1.4 Hz, 4 H; $C_{ar}H$), 3.76 (s, 3 H; OCH_3), 2.63–2.45 (m, 2 H; $COCH_2$), 2.39 (d, $^2J_{H,H}$ = 6.8 Hz, 1 H; CH_2), 2.35 (d, $^2J_{H,H}$ = 6.8 Hz, 1 H; CH_2), 1.56 (s, 3 H; CCH_3), 1.34 (s, 3 H; CCH_3), 1.08 (t, $^3J_{H,H}$ = 7.2 Hz, 3 H; CH_3). ^{13}C NMR (100 MHz, $CDCl_3$): δ = 205.7 (CO), 169.3 (C_{alkene}), 165.2 (CO_2), 154.8 (C_{alkene}), 152.6 (C_{alkene}), 146.5 (C_{alkene}), 135.8 (C_{ar}), 135.6 (C_{ar}), 129.1 (C_{ar}), 128.6 (C_{ar}), 128.1 (C_{ar}), 128.0 (C_{ar}), 127.0 (C_{ar}), 126.9 (C_{ar}), 79.9 (CH_2), 62.3 (C), 60.4 (C), 51.9 ($CO_2\mathit{C}H_3$), 36.9 ($CO\mathit{C}H_2$), 16.2 (CH_3), 15.3 (CH_3), 7.5 ppm (CH_2CH_3).

The spectroscopic data are consistent with the literature.[5]

6. Computational Details

All calculations were performed by using the program packages Maestro[9] and Gaussian 16[10]. For the larger molecules **12-14**, **16** and **17** conformational analyses were conducted using OPLS4[11] force field in $CHCl_3$. Subsequently, the three OPLS4-optimized showing the lowest energy were used as starting points for geometry optimization. The geometrical parameters of the stationary points were optimized by means of the density functional methods functionals B3LYP[12-13] with the empirical dispersion D3BJ[14]. As basis set def2-SVP[15-16] was employed. In order to adequately describe the diradical character of the transition states, we used the open-shell variant, which is implemented in Gaussian via the "guess=mix" command. This command requests the HOMO and LUMO to be mixed to break α-β and spatial symmetries, which is useful for generating UHF wave functions for singlet states. For all stationary points no symmetry restriction was applied. Frequency calculations were carried out at each of the structures to verify the nature of the stationary point. It turned out that all transition states have exactly one imaginary frequency, whereas all other structures have none. Furthermore, the energies of the stationary points were calculated using the density functionals B3LYP-D3BJ and the basis set def2-TZVP[15]. To take solvent effects into account, the solvent model SMD[17] ($CHCl_3$ as solvent) was used for the single point calculations.

Table S2. Absolute energies [au] of the calculated compounds by means of different methods.

Compound	E^a	G^a	E^b
**7**-NBD	-1192.015501	-1191.646632	-1193.3410590
**7**-QC	-1191.989508	-1191.619311	-1193.3054230
**9**-NBD	-1267.192957	-1266.819691	-1268.6052740
**9**-QC	-1267.164650	-1266.791076	-1268.5690220
trans-**12**-NBD	-2954.076256	-2953.167886	-2957.3310730
cis-**12**-QC	-2954.010032	-2953.099343	-2957.2456330
trans-**13**-NBD	-3350.700970	-3349.826106	-3354.4307020
cis-**13**-QC	-3350.636927	-3349.762864	-3354.3531160
trans-**14**-NBD	-2954.091500	-2953.180573	-2957.3435760
cis-**14**-QC	-2954.014152	-2953.104156	-2957.2502810
trans-**16**-NBD	-1802.531225	-1801.976870	-1804.5187990
cis-**16**-QC	-1802.486273	-1801.931634	-1804.4687460
TS-**16**$_{(cis\text{-}trans)}$	-1802.455166	-1801.901747	-1804.4362930
trans-**16**-QC	-1802.500022	-1801.948498	-1804.4836670
TS-**16**$_{(\text{QC-NBD})}$	-1802.461185	-1801.911330	-1804.4484720
trans,trans-**17**-NBD	-2488.229498	-2487.485893	-2490.9710420
cis,cis-**17**-QC	-2488.159527	-2487.414873	-2490.8979690

[a] B3LYP-D3BJ/def2-SVP.

[b] B3LYP-D3BJ($CHCl_3$ as solvent)/def2-TZVP//PBE0-D3BJ/def2-SVP

Cartesian coordinates of the optimized geometry of **7**-NBD at B3LYP-D3BJ/def2-SVP level of theory:

C	-1.86145000	-0.21214300	0.32440900
C	-1.38103600	-1.47434100	0.28318600
C	-0.23772100	-1.54320300	1.33720600
C	0.86944200	-0.59348900	0.78508000
C	0.37485700	0.66468500	0.82287300
C	-1.06821000	0.57544800	1.40015600
C	-0.85278200	-0.58636400	2.39293300
C	-1.72115800	1.85322500	1.89045800
C	0.18395700	-2.90212400	1.86926200
C	-2.79510900	0.50036400	-0.58372200
O	-2.44669600	1.08728100	-1.57988400
C	0.92919700	1.88811800	0.21388900
C	1.22872300	3.02634900	0.98207500
C	1.74642500	4.17583100	0.38149100
C	1.96665500	4.20798700	-0.99834400
C	1.66530800	3.08425000	-1.77357900
C	1.15063600	1.93466800	-1.17385900
C	2.14837900	-1.03842300	0.21727000
C	2.21612300	-2.18229900	-0.60251100
C	3.42882000	-2.57255700	-1.17340400
C	4.59360900	-1.83846300	-0.93310000
C	4.53804300	-0.70339500	-0.11787600
C	3.32858300	-0.30651900	0.45075100
C	-1.69574300	-2.53073900	-0.70122600
O	-0.95883100	-3.49355500	-0.84595200
H	-0.14415000	-0.32845900	3.19355600
H	-1.79144900	-0.97431300	2.81835400
H	-1.74855900	2.61504200	1.09668400
H	-1.16523700	2.27729100	2.73938400
H	-2.75286000	1.65700100	2.21939300
H	0.59160300	-3.55388400	1.09084800
H	-0.68140100	-3.41820100	2.31373000
H	0.94562200	-2.77243000	2.65378300
H	1.07286900	3.00069100	2.06258900
H	1.98281600	5.04906500	0.99458100
H	2.37120400	5.10750300	-1.46863500
H	1.82963900	3.10385100	-2.85366000

H	0.90337100	1.05985000	-1.77716100
H	1.30946300	-2.75222800	-0.81226800
H	3.46127700	-3.45736200	-1.81393500
H	5.54181500	-2.14955600	-1.37830300
H	5.44398900	-0.12437900	0.07781700
H	3.28805800	0.57735600	1.08921300
C	-5.02055800	1.18515300	-0.91890200
H	-5.97687200	1.08606900	-0.39226100
H	-5.09168800	0.75383700	-1.92853500
H	-4.73621300	2.24358800	-1.00983300
O	-4.06001100	0.47074300	-0.13484000
C	-2.93830700	-2.37005300	-1.55628000
H	-3.13547300	-3.31513600	-2.07774800
H	-3.80946300	-2.07171300	-0.95569700
H	-2.76414900	-1.58103100	-2.30590800

Cartesian coordinates of the optimized geometry of **7**-QC at B3LYP-D3BJ/def2-SVP level of theory:

C	0.42414900	-1.69023300	0.00064600
C	-1.01889200	-1.20876900	0.24099200
C	-1.09776800	-0.77667100	1.71454700
C	-0.47263300	0.16609600	0.72112300
C	0.97935600	-0.34390600	0.46971300
C	1.09042000	-1.54357400	1.38775200
C	0.01424100	-1.50054000	2.45009800
C	2.43271900	-2.16194300	1.66339500
C	-2.39505900	-0.50527100	2.43296400
C	0.88544900	-2.76758700	-0.91306800
O	1.98642200	-2.76149400	-1.43384000
C	-4.20969300	-0.49919400	-1.40232400
C	2.08866800	0.53661400	0.03901400
C	2.25815100	1.76438200	0.70318700
C	3.28885600	2.63223300	0.34320900
C	4.16926700	2.28560000	-0.68655600
C	4.00715700	1.06733700	-1.35038500
C	2.97198400	0.19706400	-0.99675200
C	-0.97130300	1.48401800	0.27020400
C	-0.91402000	1.81486800	-1.09417600
C	-1.34585800	3.06382300	-1.53925500
C	-1.83788700	4.00322700	-0.62656700
C	-1.89062900	3.68643300	0.73284300
C	-1.45716900	2.43484500	1.17859600
C	-2.05949400	-1.23777300	-0.79514700
O	-1.88084800	-1.63124200	-1.92935400
O	-3.22273700	-0.69356100	-0.39094500
H	0.34277200	-0.93297700	3.33744200
H	-0.29232700	-2.50906300	2.77436600
H	2.95603600	-1.59231700	2.44788800
H	2.32255400	-3.20212400	2.00967700
H	3.06403800	-2.16718300	0.76557300
H	-2.19388100	0.01608000	3.38254800
H	-3.07878700	0.10320400	1.83303800
H	-2.90392000	-1.45270700	2.67117900
H	-5.08063400	-0.06503900	-0.89671600
H	-4.47664800	-1.45350800	-1.87921600
H	1.56943600	2.04360300	1.50310600
H	3.40401700	3.58445700	0.86696300
H	4.97596300	2.96548000	-0.97150000
H	4.68927800	0.78945700	-2.15777700
H	2.84271600	-0.75662400	-1.50896200
H	-0.52826700	1.08197700	-1.80611600
H	-1.29565900	3.30776100	-2.60310300
H	-2.17503500	4.98231100	-0.97519100
H	-2.26767500	4.41811800	1.45155500

H	-1.48864300	2.19433500	2.24268100
H	-3.83703900	0.18890000	-2.17577000
C	-0.05877500	-3.93676400	-1.08635700
H	-0.79233400	-3.67537000	-1.86367300
H	0.51470900	-4.81941200	-1.39863000
H	-0.62079400	-4.14485900	-0.16243900

Cartesian coordinates of the optimized geometry of **9**-NBD at B3LYP-D3BJ/def2-SVP level of theory:

C	1.57986300	-0.72353000	0.65956800
C	1.65567700	0.62299800	0.65523600
C	0.52664800	1.15426400	1.57247600
C	-0.79746500	0.73672800	0.86130400
C	-0.85721300	-0.61422200	0.88397500
C	0.42381500	-1.12315400	1.61490900
C	0.57493300	0.02797400	2.63835200
C	0.43385600	-2.54572900	2.14029400
C	0.64239700	2.57672500	2.08835200
C	2.34846300	-1.69252300	-0.15392500
O	2.84301100	-2.70781500	0.27800600
C	4.60117100	1.67620800	-1.24581500
C	-1.78053800	-1.48794800	0.13821700
C	-2.51404000	-2.51097900	0.76501200
C	-3.37618000	-3.32545500	0.02827100
C	-3.51621900	-3.13749500	-1.34955900
C	-2.78883800	-2.12732800	-1.98613500
C	-1.93003700	-1.31082700	-1.25034400
C	-1.72314700	1.67076500	0.20585100
C	-1.24204600	2.71594000	-0.60658100
C	-2.13335300	3.57325500	-1.25473000
C	-3.51298400	3.41009200	-1.10059200
C	-4.00005000	2.37665400	-0.29388200
C	-3.11455500	1.51529300	0.35269200
C	2.51412600	1.47797400	-0.18447400
O	2.15713300	2.52856500	-0.67563200
O	3.74739500	0.97186700	-0.34686400
H	-0.26665800	0.07843100	3.34469200
H	1.53278600	-0.00246200	3.18064100
H	0.23659200	-3.27597700	1.34295900
H	-0.33562100	-2.67114800	2.91693200
H	1.41373500	-2.78798800	2.57376100
H	0.67688700	3.31189000	1.27592600
H	1.56149000	2.69019300	2.68487300
H	-0.21657100	2.81600700	2.73332100
H	4.73234200	2.72012700	-0.92492800
H	5.56161500	1.14715100	-1.23218100
H	-2.42376400	-2.65439600	1.84275000
H	-3.94476300	-4.10955600	0.53433500
H	-4.19026800	-3.77629600	-1.92529900
H	-2.89074100	-1.97437700	-3.06337300
H	-1.35870500	-0.52372600	-1.74562000
H	-0.16684700	2.83499200	-0.75270200
H	-1.74432900	4.37359700	-1.88915300
H	-4.20696200	4.08574500	-1.60656300
H	-5.07704500	2.24234200	-0.16599500
H	-3.49449100	0.71024000	0.98413300
H	4.17737400	1.67483700	-2.26176000
C	3.17236100	-2.17727400	-2.30023500
H	3.07615100	-1.76299400	-3.31108300
H	2.79449500	-3.20994500	-2.27036100
H	4.22779800	-2.18394800	-1.98740300
O	2.40316300	-1.33051400	-1.44892500

Cartesian coordinates of the optimized geometry of **9**-QC at B3LYP-D3BJ/def2-SVP level of theory:

C	-1.35206800	0.51957300	0.40630400
C	-1.02369200	-0.96972300	0.57829300
C	-0.38744700	-1.11606000	1.95591300
C	0.46353400	-0.60999600	0.81195600
C	0.10748400	0.90042900	0.63194800
C	-0.90328100	1.15506700	1.73409800
C	-0.82200600	0.07343500	2.79122100
C	-1.30716700	2.56209800	2.07813300
C	-0.18377200	-2.46192300	2.60298200
C	-2.47354300	1.07494900	-0.37566200
O	-2.49854800	2.13670600	-0.95892200
C	-1.99272500	-2.51865400	-2.57673900
C	1.02494100	1.89676600	0.03677900
C	2.36060500	1.91664700	0.47549200
C	3.26741300	2.83850600	-0.04680500
C	2.85335700	3.75763200	-1.01613100
C	1.52830800	3.74279200	-1.45779200
C	0.61744900	2.81661900	-0.94130500
C	1.62621000	-1.26114900	0.17040200
C	1.75807500	-1.21351300	-1.22792000
C	2.86744200	-1.77914400	-1.85522600
C	3.86361200	-2.40029600	-1.09450800
C	3.74419300	-2.44814400	0.29641600
C	2.63393200	-1.87847000	0.92522600
C	-1.38697300	-2.05546000	-0.34838700
O	-1.35511800	-3.24090900	-0.11266900
O	-1.68328100	-1.55825100	-1.57112100
H	-0.07664200	0.31939400	3.56646500
H	-1.79505300	-0.09996200	3.27985900
H	-0.57007000	3.01297900	2.76151800
H	-2.29000900	2.57551500	2.57603100
H	-1.37100700	3.19293500	1.18171000
H	0.55382900	-2.38689700	3.41806200
H	0.15340500	-3.21367000	1.88076800
H	-1.13120700	-2.82074300	3.03403900
H	-2.21769600	-1.94771800	-3.48594600
H	-1.13906700	-3.19152700	-2.74814500
H	2.69100400	1.19564600	1.22579000
H	4.30301700	2.83776400	0.30214000
H	3.56337700	4.47919200	-1.42766400
H	1.19711200	4.45510600	-2.21763900
H	-0.41633800	2.80200700	-1.28736300
H	0.97834500	-0.72827500	-1.81830600
H	2.95771900	-1.73453800	-2.94327200
H	4.73273500	-2.84399300	-1.58603000
H	4.52084700	-2.92846300	0.89654400
H	2.54935800	-1.90656900	2.01278400
C	-4.68427800	0.64557700	-1.05508500
H	-5.43961000	-0.13178100	-0.88723400
H	-4.44251200	0.71808000	-2.12645000
H	-5.05855200	1.62362900	-0.71747000
O	-3.53677900	0.25294400	-0.30799200
H	-2.86080000	-3.12737900	-2.28218800

Cartesian coordinates of the optimized geometry of *trans*-**12**-NBD at B3LYP-D3BJ/def2-SVP level of theory:

C	5.45545300	2.17739900	-0.06906300
C	6.14636500	1.14436600	0.45966900
C	5.68500500	0.99231700	1.93914800
C	4.19394200	0.54267800	1.83265000
C	3.50492400	1.56573200	1.27008100

C	4.52026100	2.73110100	1.03811500
C	5.44391100	2.49058000	2.25045300
C	3.99556100	4.13826000	0.82238200
C	6.59420900	0.26948800	2.91810300
C	5.41396000	2.72738400	-1.46011400
C	7.11941400	-0.75921300	-2.48432400
C	5.78116800	-1.44801300	-2.58811500
C	4.82304300	-1.00124100	-3.50944500
C	3.49614100	-1.41986300	-3.41182100
C	3.11090500	-2.28177800	-2.37510400
C	4.08333800	-2.80862200	-1.50592600
C	5.40461000	-2.40016900	-1.62222300
N	1.72441300	-2.51026200	-2.22039000
N	1.41687400	-3.08005800	-1.14785300
C	0.04231300	-3.25297700	-0.90207200
C	-0.27917000	-3.72431800	0.37885500
C	-1.61145200	-3.88250400	0.75272900
C	-2.63673900	-3.58533200	-0.15400600
C	-2.30855600	-3.12474100	-1.44307500
C	-0.98227900	-2.95759100	-1.82183300
C	-4.07872400	-3.66666900	0.26729600
O	6.22945200	3.54513500	-1.82536900
C	4.24411700	2.28033300	-2.30205800
C	-4.75029400	-0.45992100	1.89992100
C	-6.20250600	-0.10709100	1.47549400
C	-6.01556600	0.59319500	0.09694900
C	-4.08381600	0.69578300	2.13951400
C	-5.10507900	1.84802000	1.86643800
C	-5.37444500	1.76234100	0.33131300
C	-6.38839300	1.13785300	2.36099000
C	-2.63306700	0.92844600	2.36882000
O	-2.22216400	2.07152800	2.50407900
C	-1.67058700	-0.23710200	2.37849400
C	-7.23575500	-1.21192100	1.59318700
C	-4.87597600	3.22324400	2.47221200
C	2.15921800	1.52614500	0.67001900
C	1.82799900	0.48409600	-0.22038100
C	0.58507400	0.44963900	-0.85109700
C	-0.35821200	1.45250900	-0.60120300
C	-0.04780200	2.48319600	0.28895100
C	1.19846300	2.52204600	0.91610900
C	6.89040000	0.12050600	-0.27988200
O	7.32915300	-0.90222400	0.20816800
O	6.95262500	0.38135500	-1.60976800
C	3.68409400	-0.78942100	2.17956500
C	2.41115100	-0.93258600	2.76815000
C	1.90089600	-2.19210600	3.07407600
C	2.65357600	-3.34032900	2.80256900
C	3.91727200	-3.21376400	2.22032900
C	4.43142600	-1.95265200	1.91285000
C	-4.26888400	-1.85972100	1.81922000
O	-3.72679400	-2.49585700	2.69145200
O	-4.56701400	-2.34997300	0.60085200
C	-4.85562000	2.70738500	-0.66363700
C	-5.61596000	3.05261300	-1.79758500
C	-5.09390200	3.89835900	-2.77500300
C	-3.80071200	4.41569700	-2.64316300
C	-3.03743600	4.08567300	-1.52004400
C	-3.56127500	3.24694500	-0.53497600
C	-6.21092800	-0.09405100	-1.19261300
C	-5.10205600	-0.31089400	-2.03248400
C	-5.24291400	-0.99215300	-3.24071000
C	-6.49593700	-1.47417100	-3.63505000
C	-7.60489500	-1.26538900	-2.81126500
C	-7.46323900	-0.58350000	-1.59992700
H	4.92738000	2.64886400	3.20862400

H	6.37089700	3.08266600	2.20862400
H	3.29012800	4.19372700	-0.01926000
H	3.47288900	4.50106200	1.71976300
H	4.83013000	4.82345000	0.61096700
H	6.79970800	-0.76442800	2.62458700
H	7.56214700	0.79028800	2.98246500
H	6.13774300	0.26704200	3.91999500
H	7.89873600	-1.41921200	-2.08173500
H	7.44337000	-0.33536800	-3.44275000
H	5.10985900	-0.27953600	-4.27818700
H	2.72761100	-1.04540200	-4.09042500
H	3.76585300	-3.49002100	-0.71828600
H	6.15310000	-2.76712200	-0.91942800
H	0.54083800	-3.92854000	1.06741900
H	-1.86676100	-4.21215200	1.76106400
H	-3.11250800	-2.88684900	-2.14448300
H	-0.71079600	-2.60153700	-2.81603900
H	-4.72360300	-3.99264300	-0.55841000
H	-4.20996100	-4.33111100	1.13158200
H	4.32271900	2.71703400	-3.30687100
H	4.21268400	1.18386900	-2.35593700
H	3.29989000	2.59577800	-1.82936700
H	-7.30191400	1.69738200	2.11244900
H	-6.35674100	0.91236800	3.43815800
H	-1.69147300	-0.76193500	1.41004600
H	-0.65839700	0.15009800	2.54514800
H	-1.94380900	-0.97297600	3.14690700
H	-7.06951800	-2.00495000	0.85256300
H	-7.20157000	-1.66257700	2.59766000
H	-8.24802200	-0.80801400	1.44186200
H	-3.99265200	3.72998100	2.07517100
H	-5.75886800	3.85302200	2.27836200
H	-4.74312600	3.14108400	3.56120500
H	2.56170700	-0.29773500	-0.42019900
H	0.36108200	-0.36179300	-1.54572800
H	-1.33297400	1.43171700	-1.09391100
H	-0.78362900	3.25337900	0.51985200
H	1.41683900	3.32071000	1.62564900
H	1.82708100	-0.03832000	2.99036800
H	0.91133500	-2.27768400	3.52972700
H	2.25918900	-4.32905200	3.05002700
H	4.51021200	-4.10523900	2.00083500
H	5.40878500	-1.86733500	1.43826500
H	-6.62442700	2.64965200	-1.90329000
H	-5.70003400	4.15657500	-3.64679700
H	-3.39154000	5.07581200	-3.41160600
H	-2.02360500	4.47808500	-1.41057300
H	-2.95671300	2.98463200	0.33262000
H	-4.12289700	0.05388300	-1.71824500
H	-4.37029600	-1.14654300	-3.88069200
H	-6.60736300	-2.00703600	-4.58237000
H	-8.58855400	-1.63221500	-3.11450700
H	-8.33741300	-0.41349000	-0.96915700

Cartesian coordinates of the optimized geometry of *cis*-**12**-QC at B3LYP-D3BJ/def2-SVP level of theory:

C	-3.49228200	0.50309900	0.97890000
C	-4.15159300	0.74678000	-0.39101500
C	-5.19934300	1.84793300	-0.17808500
C	-3.74989400	2.24028200	-0.31663200
C	-3.08429800	1.98331900	1.07677000
C	-4.23813300	1.49012100	1.92193300
C	-5.56234100	1.85778400	1.29129600
C	-4.10452500	1.38278000	3.41522700

C	-6.24400300	2.18177500	-1.21157300
C	-3.01978600	-0.77658200	1.54543300
O	-2.13509600	-0.84535500	2.38634500
C	-3.76573100	-2.02475900	1.11795600
C	2.15502700	0.62564100	1.01191100
C	1.22076900	0.79918000	-0.19938900
C	2.61928200	0.26028400	-0.40820900
C	3.03708100	1.89183000	0.98696400
C	2.56670400	2.70097800	-0.20096900
C	3.52525000	1.53265300	-0.42761200
C	1.17127300	2.26304100	-0.58102700
C	3.84128300	2.42256100	2.13756400
O	3.64213700	3.53525100	2.58218600
C	4.95804100	1.54724900	2.65614300
C	0.02695600	-0.08257500	-0.43119000
C	2.96571100	4.14563600	-0.35943700
C	-2.56349800	-1.65324700	-2.69190700
C	0.98421900	-2.12700300	3.19221300
C	-2.12040600	-3.02086400	-2.25964300
C	-3.07353600	-4.02307200	-2.01956200
C	-2.69326400	-5.27161100	-1.53211900
C	-1.34778500	-5.51488400	-1.22664200
C	-0.37900700	-4.54379000	-1.51814500
C	-0.76699500	-3.31032600	-2.03449400
N	-0.99469500	-6.79534000	-0.69514000
N	-0.34453800	-6.90541900	0.36187300
C	-0.05133700	-5.76241700	1.16750300
C	-1.05993100	-4.89524800	1.61395100
C	-0.72153700	-3.75849200	2.33960600
C	0.61865800	-3.46907700	2.62866900
C	1.60921700	-4.38971400	2.25874700
C	1.27774800	-5.54576000	1.55241800
C	-1.88213600	2.69574000	1.57472700
C	-0.88774800	2.06719200	2.34398800
C	0.21652200	2.78961200	2.80237000
C	0.36075600	4.14282900	2.48729400
C	-0.62639600	4.77815600	1.72913800
C	-1.74041300	4.06403100	1.28208600
C	-3.16398100	3.03553400	-1.42032700
C	-3.72086800	4.27095200	-1.78409700
C	-3.14659900	5.03903100	-2.79901100
C	-2.00751600	4.57951000	-3.46581600
C	-1.44757800	3.34876300	-3.11187500
C	-2.02167500	2.58408200	-2.09633100
C	-3.94362700	0.00082400	-1.64465600
O	-4.52227100	0.20369000	-2.68736000
O	-2.94670700	-0.90652800	-1.51491400
C	1.89238200	-0.12681700	2.25063800
O	2.22346200	0.25865800	3.35318500
O	1.25720500	-1.29004400	2.04566700
C	4.89578600	1.53669400	-0.98568300
C	5.30944400	2.47628600	-1.94309400
C	6.59887000	2.42988600	-2.48035300
C	7.49335500	1.43519300	-2.07946700
C	7.08781900	0.48174400	-1.13979700
C	5.80301500	0.53216700	-0.60074200
C	2.92408500	-1.00776800	-1.10661400
C	3.27985900	-0.94125600	-2.46618900
C	3.54000200	-2.10266400	-3.19387700
C	3.45525800	-3.35485800	-2.57565800
C	3.11148400	-3.42957900	-1.22400100
C	2.84730700	-2.26722400	-0.49459700
H	-5.89707400	2.85743300	1.61647400
H	-6.35445400	1.12907400	1.53078000
H	-4.27027100	2.36992000	3.87513200
H	-4.85099400	0.68375500	3.82497700

H	-3.10917500	1.03244100	3.71370400
H	-6.68442300	3.16840300	-0.99645800
H	-5.82894300	2.18612200	-2.22479100
H	-7.05498300	1.43717000	-1.18616900
H	-4.69950100	-1.80508600	0.58349200
H	-3.12064300	-2.61413200	0.45061700
H	-3.98036900	-2.62672000	2.01313200
H	0.99058900	2.38069100	-1.66209800
H	0.39356300	2.81369300	-0.03638700
H	4.68254000	0.48498200	2.64880100
H	5.82771300	1.67596700	1.98927300
H	5.23607000	1.86081900	3.67072300
H	-0.18872300	-0.12381700	-1.50959300
H	0.18125100	-1.10556300	-0.07383300
H	-0.85604800	0.32062400	0.07800300
H	2.83103100	4.47539100	-1.40221900
H	2.33137900	4.77317400	0.28265900
H	4.00917400	4.32370100	-0.06964100
H	-1.75015800	-1.10315300	-3.18954700
H	-3.42249500	-1.68808500	-3.37465800
H	0.15911200	-1.67743800	3.76167200
H	1.87965600	-2.15711200	3.82934200
H	-4.12941500	-3.81834200	-2.21541100
H	-3.42957900	-6.05638200	-1.34795200
H	0.67351300	-4.74098500	-1.32097000
H	-0.00156300	-2.55863000	-2.23865700
H	-2.10048300	-5.09523900	1.35775700
H	-1.48780400	-3.04167200	2.63761600
H	2.65508900	-4.18849000	2.50560700
H	2.04125600	-6.26436900	1.24801800
H	-0.98072000	1.00341500	2.56153400
H	0.98484200	2.28337400	3.38700600
H	1.25503200	4.67664700	2.81278300
H	-0.52785700	5.83632500	1.47428100
H	-2.49989700	4.57254100	0.68808200
H	-4.60726700	4.63381500	-1.26004700
H	-3.59028400	5.99973300	-3.07089300
H	-1.55843100	5.17858400	-4.26141500
H	-0.55920900	2.98197100	-3.63158700
H	-1.58261400	1.62764400	-1.81562200
H	4.61268300	3.24463900	-2.27758800
H	6.90256200	3.17262000	-3.22219300
H	8.50031400	1.39759200	-2.50171700
H	7.77623400	-0.30738000	-0.82777600
H	5.48842700	-0.22155200	0.12311800
H	3.35744900	0.03384500	-2.95146500
H	3.81456300	-2.03046100	-4.24904800
H	3.66121000	-4.26468600	-3.14426000
H	3.04947800	-4.39900800	-0.72349100
H	2.56102400	-2.33729500	0.55123800

Cartesian coordinates of the optimized geometry of *trans*-**13**-NBD at B3LYP-D3BJ/def2-SVP level of theory:

C	4.45562400	1.23500800	-1.82100100
C	5.56708700	0.65659200	-1.31178100
C	6.18643800	1.65895300	-0.29203000
C	5.13146400	1.72727500	0.85284600
C	4.00595100	2.27345600	0.32932700
C	4.31725700	2.63258800	-1.15634400
C	5.81581900	2.97237600	-1.02173600
C	3.42725300	3.62897200	-1.87150500
C	7.65170700	1.50694400	0.07637700
C	3.38941800	0.73824600	-2.74752200
C	5.11897700	-2.85586500	-2.18001600

C	3.84832000	-3.15762900	-1.42211500
C	2.72554900	-3.63885600	-2.10023300
C	1.51048300	-3.72633400	-1.43402800
C	1.34021000	-3.31943500	-0.09096800
C	2.51231300	-2.88268500	0.57671200
C	3.74145400	-2.81056100	-0.06459100
N	0.02624800	-3.35305000	0.37501600
N	-0.21047200	-2.83673600	1.49698400
C	-1.56590400	-2.78718500	1.84595800
C	-1.92543800	-1.84266700	2.83293800
C	-3.24347800	-1.54798900	3.14757000
C	-4.27204500	-2.23266100	2.48906000
C	-3.95758400	-3.24789100	1.57703500
C	-2.63140000	-3.52826800	1.27853300
C	-5.70199400	-1.78532800	2.65650700
O	3.38706900	1.10655100	-3.90477200
C	2.27383800	-0.06899500	-2.13615700
C	-5.60647800	1.31767500	0.70831200
C	-6.10272800	0.92360900	-0.71188900
C	-4.80251000	0.55355800	-1.49139200
C	-4.90361100	2.46747000	0.58625100
C	-4.88099900	2.83665600	-0.91951900
C	-4.08257100	1.69193100	-1.62023300
C	-6.30975800	2.35327900	-1.27067700
C	-4.10147700	3.22473400	1.59677600
O	-2.89656000	3.31536400	1.44996800
C	-4.84336000	3.92205200	2.70388800
C	-7.30407300	-0.00129800	-0.80441000
C	-4.50929500	4.26307300	-1.27691500
C	2.63338800	2.14521000	0.85410400
C	2.19775600	0.86559300	1.25037300
C	0.89188000	0.64787800	1.68197900
C	-0.00930700	1.71564700	1.73863400
C	0.40914700	2.99614800	1.36442000
C	1.71726700	3.21006200	0.91943400
C	5.91825600	-0.76039000	-1.38060200
O	6.68575500	-1.32234900	-0.62364500
O	5.21680200	-1.42821400	-2.33319600
C	5.28454500	1.13740800	2.18854900
C	4.73887200	1.78741000	3.31297000
C	4.81716500	1.21177700	4.57949900
C	5.44344200	-0.02689500	4.75261100
C	5.99440000	-0.67982000	3.64711000
C	5.92294400	-0.10455300	2.37675800
C	-5.59313900	0.47563000	1.91583200
O	-5.21716400	0.83476600	3.01389400
O	-5.99004200	-0.78797400	1.65578500
C	-2.76035200	1.85827800	-2.24403800
C	-2.51920100	1.36398900	-3.53960900
C	-1.25231700	1.46207800	-4.11514300
C	-0.20085300	2.05694300	-3.40945600
C	-0.43424900	2.55391400	-2.12376900
C	-1.70045100	2.46194400	-1.54435000
C	-4.35195400	-0.81340400	-1.80348600
C	-3.05011600	-1.21265600	-1.43742100
C	-2.57763400	-2.48680900	-1.74189100
C	-3.40242900	-3.40153600	-2.40393700
C	-4.69787700	-3.02634800	-2.76637500
C	-5.16843000	-1.74397900	-2.47153100
H	5.98800100	3.86517700	-0.40311300
H	6.32169200	3.07216400	-1.99424100
H	2.37671000	3.31005700	-1.87369500
H	3.47849100	4.61321800	-1.38264300
H	3.74395500	3.73968100	-2.91858800
H	7.87091500	0.54476400	0.55051500
H	8.27723600	1.58307600	-0.82676400

H	7.94809400	2.31104500	0.76740100
H	6.01046300	-3.22370000	-1.65530800
H	5.08251300	-3.27124000	-3.19439300
H	2.76562300	-3.90828800	-3.15644800
H	4.59993200	-2.44133300	0.49738000
H	-3.45680200	-0.75148800	3.85926300
H	-4.73381500	-3.81210400	1.05804800
H	-6.40644600	-2.60141300	2.45468400
H	-5.88383400	-1.36690800	3.65464600
H	1.68836800	-0.55790700	-2.92542800
H	2.65351100	-0.79718200	-1.41261400
H	1.61189900	0.61674600	-1.58316300
H	-6.49994800	2.35855600	-2.35366500
H	-7.09631600	2.91295200	-0.73969400
H	-5.39322600	3.17438600	3.29278500
H	-4.13500100	4.47114500	3.33828200
H	-5.57964400	4.61863300	2.26858600
H	-7.05500900	-1.03034100	-0.52285400
H	-8.10604800	0.34869800	-0.13600800
H	-7.70064400	-0.00332900	-1.83126000
H	-3.49371200	4.51581300	-0.94475500
H	-4.54783900	4.40369100	-2.36729700
H	-5.21082700	4.97530100	-0.81382300
H	2.89809200	0.03118100	1.21378100
H	0.58960400	-0.35695700	1.97584100
H	-1.03737500	1.56075500	2.07068900
H	-0.30158400	3.82316400	1.41633300
H	2.03623500	4.21409900	0.63289000
H	4.24861600	2.75305000	3.17931300
H	4.38600300	1.73236500	5.43806600
H	5.50217900	-0.47956500	5.74530000
H	6.48272700	-1.64957700	3.77133600
H	6.34034700	-0.63394700	1.51986000
H	-3.33697900	0.89139900	-4.08711100
H	-1.08114100	1.06784200	-5.11983100
H	0.79555100	2.11172000	-3.85485700
H	0.37534200	3.00056600	-1.54498000
H	-1.86262100	2.81560800	-0.52578600
H	-2.40815500	-0.50852800	-0.90590100
H	-1.57349900	-2.78587100	-1.44116200
H	-3.03109600	-4.40399000	-2.62771400
H	-5.34678100	-3.73153200	-3.29209300
H	-6.17000800	-1.45495800	-2.78997700
F	0.44760500	-4.15775300	-2.10850400
F	-2.37622300	-4.52897900	0.44271700
F	-0.96125400	-1.15970700	3.44726400
F	2.44850200	-2.50348000	1.84849600

Cartesian coordinates of the optimized geometry of *cis*-**13**-QC at B3LYP-D3BJ/def2-SVP level of theory:

C	-2.26365800	2.70825400	1.00234800
C	-2.66813800	3.29841400	-0.35999400
C	-2.86304200	4.80412100	-0.14181100
C	-1.46132800	4.26898200	-0.29440900
C	-1.06157700	3.66261500	1.09216500
C	-2.27696900	3.94139400	1.94978800
C	-3.13639300	5.02107400	1.33123500
C	-2.21859900	3.76806300	3.44173700
C	-3.52270900	5.69013500	-1.16698400
C	-2.64347700	1.39597800	1.56357000
O	-1.96004400	0.79597600	2.38132900
C	-4.00318500	0.86216200	1.16194200
C	2.38890400	-0.51791300	0.99922600
C	1.72913100	0.16684400	-0.21760300

C	2.53746700	-1.09091000	-0.41480900
C	3.84773800	-0.01476200	0.96684900
C	3.93736400	0.90853800	-0.22784400
C	4.02094300	-0.60150600	-0.44662800
C	2.55027400	1.37821400	-0.60238800
C	4.81486000	-0.05047200	2.11360900
O	5.31522600	0.96853300	2.54583800
C	5.19487900	-1.41213300	2.64555600
C	0.24417200	0.15023400	-0.44454200
C	5.11249900	1.83664000	-0.39930000
C	-2.74228400	0.43042500	-2.66242000
C	-0.18668200	-2.06409500	3.13998400
C	-3.24557700	-0.92000700	-2.23590100
C	-4.60229800	-1.07322400	-1.91481400
C	-5.06147800	-2.28359300	-1.41532500
C	-4.19437900	-3.36209500	-1.18999400
C	-2.85828000	-3.20168800	-1.58209200
C	-2.37054700	-2.00272900	-2.08807000
N	-4.70440400	-4.58437400	-0.65874600
N	-4.21667500	-5.06770400	0.38008000
C	-3.25470300	-4.32850800	1.13072900
C	-3.51188300	-3.05739000	1.66595300
C	-2.55430300	-2.34163700	2.36736100
C	-1.29264700	-2.90762400	2.57319800
C	-1.02962600	-4.20676500	2.12015500
C	-2.00766800	-4.90061400	1.42192400
C	0.33387700	3.52804700	1.57627000
C	0.77171300	2.43240800	2.33992400
C	2.09303600	2.36359200	2.78800300
C	3.00533000	3.37161200	2.46706400
C	2.57739300	4.46805000	1.71366000
C	1.25304200	4.54967300	1.27752300
C	-0.52930300	4.56664300	-1.40634700
C	-0.26926500	5.89217700	-1.78566900
C	0.63810300	6.17429700	-2.80894400
C	1.29685100	5.13272600	-3.46790600
C	1.04322300	3.80912700	-3.09716400
C	0.13793900	3.52940400	-2.07358000
C	-2.94147800	2.57385900	-1.61136900
O	-3.31844000	3.06797400	-2.64817800
O	-2.63060800	1.25738900	-1.48839500
C	1.72490800	-0.97656600	2.22744300
O	2.19397200	-0.85381800	3.34026100
O	0.53824800	-1.56527700	1.99980800
C	5.11830400	-1.40987300	-1.02201200
C	5.98588700	-0.89950200	-2.00044900
C	6.99063900	-1.69878900	-2.55238400
C	7.13692700	-3.02674000	-2.14565600
C	6.26694300	-3.55351800	-1.18549800
C	5.26851200	-2.75341700	-0.63117100
C	2.01529700	-2.30679100	-1.07659200
C	2.01161100	-2.34386800	-2.48143200
C	1.49919900	-3.44912800	-3.16269400
C	0.97652700	-4.53248100	-2.44910400
C	0.98365600	-4.50835100	-1.05294300
C	1.50792200	-3.40692700	-0.37265300
H	-2.81463900	6.02485300	1.65655600
H	-4.20335300	4.89812900	1.58045200
H	-1.76541600	4.66006100	3.90249400
H	-3.22982900	3.64193700	3.86022900
H	-1.61866800	2.89645300	3.72998900
H	-3.29691500	6.74686800	-0.95297900
H	-3.19546300	5.45180800	-2.18444000
H	-4.61604900	5.56430400	-1.12971100
H	-4.61255900	1.60202200	0.62613800
H	-3.87178200	-0.01246500	0.51024800

H	-4.53305000	0.52848300	2.06578900
H	2.47001700	1.57878300	-1.68355300
H	2.24961400	2.28232800	-0.05761100
H	4.33239100	-2.09014900	2.68066500
H	5.94094100	-1.84930700	1.96016200
H	5.63988900	-1.31164100	3.64396600
H	0.04491900	0.25118700	-1.52241900
H	-0.22145000	-0.77780600	-0.09736300
H	-0.24046300	0.98662300	0.07241100
H	5.18938200	2.17984400	-1.44354300
H	4.97960900	2.71912600	0.24266100
H	6.06150200	1.36231000	-0.11829500
H	-1.74682300	0.35836900	-3.12551000
H	-3.42116200	0.92082400	-3.37254300
H	-0.57419400	-1.21245700	3.71547100
H	0.50019500	-2.63814000	3.77867200
H	-5.30824200	-0.24853700	-2.02565300
H	-1.31039500	-1.93188700	-2.33700600
H	-2.76144200	-1.32272500	2.69443500
H	-0.05614400	-4.67642500	2.26837400
H	0.07066100	1.62773700	2.56127900
H	2.41873500	1.50091200	3.36938800
H	4.04486400	3.27455000	2.78415400
H	3.27942300	5.26447000	1.45444500
H	0.93489600	5.40937700	0.68752600
H	-0.77781000	6.70738800	-1.26745200
H	0.83151100	7.21135400	-3.09313000
H	2.00565600	5.35212400	-4.26974100
H	1.55366800	2.99023200	-3.60967200
H	-0.05472000	2.49874000	-1.77886700
H	5.86757700	0.12968100	-2.33898700
H	7.65818700	-1.28175000	-3.31044600
H	7.92082200	-3.65209600	-2.57937500
H	6.36570400	-4.59464900	-0.86888400
H	4.58669500	-3.17349300	0.11003300
H	2.41915100	-1.49607600	-3.03778300
H	1.50409800	-3.46432100	-4.25541100
H	0.55689700	-5.38990700	-2.97962900
H	0.55682900	-5.34213500	-0.49150800
H	1.50224300	-3.38479000	0.71501000
F	-1.73179400	-6.11094800	0.93119900
F	-4.71620900	-2.50264700	1.45736000
F	-2.03019200	-4.24260600	-1.44345100
F	-6.34139800	-2.41627700	-1.07466000

Cartesian coordinates of the optimized geometry of *trans*-**14**-NBD at B3LYP-D3BJ/def2-SVP level of theory:

C	-3.37470100	1.35624700	-1.79801800
C	-4.05714800	1.82229500	-0.72811900
C	-5.37300700	1.00568800	-0.61545400
C	-4.92429300	-0.44507000	-0.27978600
C	-4.24556500	-0.91501300	-1.35493500
C	-4.23956900	0.21923300	-2.42768200
C	-5.63412800	0.81932400	-2.13105600
C	-3.93272600	-0.13794300	-3.86972600
C	-6.52146800	1.60397900	0.17754800
C	-2.03946700	1.74961700	-2.34983600
C	-1.37869200	3.94090700	2.87118600
C	-0.29595500	3.41043600	3.57640500
C	0.91349600	3.17092000	2.92403200
C	1.04484300	3.47934700	1.56203600
C	-0.03045100	4.05589700	0.86746700
C	-1.24786400	4.27277200	1.51159900
N	2.27175300	3.13637200	0.95538900

N	2.35216400	3.37307100	-0.27246100
C	3.56493400	2.98935500	-0.88254000
C	3.65386900	3.16164100	-2.27195300
C	4.80274900	2.74831100	-2.94740200
C	5.85083500	2.14546300	-2.24647600
C	5.77389200	1.98313200	-0.85424800
C	4.63883000	2.42420600	-0.17830200
O	-1.95034000	2.07657000	-3.51820500
C	-0.84941500	1.62343200	-1.44142400
C	4.78358300	-1.79907800	0.29662500
C	3.90679400	-2.79699100	-0.50500100
C	2.47477000	-2.17964100	-0.45636300
C	4.34655500	-1.82472400	1.57286700
C	3.20301900	-2.87707700	1.65723800
C	2.04236200	-2.24197900	0.82354000
C	3.73457000	-3.87012900	0.59916800
C	4.70692400	-0.97601800	2.75107000
O	5.62229900	-1.27324900	3.48592400
C	3.78006500	0.19198300	3.00441900
C	4.38899300	-3.29172300	-1.85616900
C	2.91708700	-3.41091200	3.04858400
C	-2.41765900	4.79325200	0.71469400
C	6.81439900	1.21037700	-0.07719900
C	-3.49882300	-2.18267200	-1.44571100
C	-2.63984700	-2.56534200	-0.39732700
C	-1.98299800	-3.79439100	-0.42485600
C	-2.14522700	-4.65903100	-1.51131000
C	-2.97566900	-4.28284700	-2.57033900
C	-3.64924500	-3.05998500	-2.53614600
C	-5.14896800	-1.10128700	1.01621800
C	-5.53047800	-2.45515600	1.08217200
C	-5.69360900	-3.09148500	2.31148100
C	-5.48516200	-2.38766200	3.50268600
C	-5.12038000	-1.03964800	3.45044300
C	-4.95626300	-0.39837200	2.22082900
C	-3.65341600	2.81511700	0.27910500
O	-3.97248800	2.76250400	1.45131000
O	-2.89732400	3.80816700	-0.22626200
C	5.72986200	-0.86697600	-0.32204900
O	6.03948200	-0.87267800	-1.49527800
O	6.19227800	0.06899700	0.54208800
C	0.83184500	-1.64559900	1.41909300
C	0.51513800	-0.29275900	1.19198500
C	-0.61652100	0.28243400	1.77318200
C	-1.45728600	-0.47762400	2.58823900
C	-1.16177100	-1.82421800	2.81804400
C	-0.02618100	-2.39886000	2.24466800
C	1.82821400	-1.55148400	-1.62008400
C	2.48617600	-0.54227900	-2.35049900
C	1.85645900	0.06648700	-3.43779200
C	0.57461600	-0.33197200	-3.83115700
C	-0.07651500	-1.34530300	-3.12277500
C	0.54153300	-1.94317800	-2.02327100
H	-6.45007600	0.11033100	-2.33622000
H	-5.81116900	1.77270100	-2.65254100
H	-2.96093300	-0.64008300	-3.97015000
H	-4.70779600	-0.80705400	-4.27195900
H	-3.89570200	0.77076400	-4.48425500
H	-6.26662700	1.77347100	1.22922000
H	-6.81495300	2.57266700	-0.25713400
H	-7.39335600	0.93354600	0.13718200
H	-2.34246800	4.08071500	3.36385200
H	-0.40566100	3.15616700	4.63259700
H	1.76461500	2.72319500	3.43953500
H	0.10317900	4.29769000	-0.18686700
H	2.80499900	3.60264700	-2.79735000

H	4.87160300	2.86825900	-4.03074000
H	6.72277800	1.76652900	-2.78314600
H	4.55028000	2.30814900	0.90078900
H	-0.67041500	0.55360000	-1.25291500
H	0.04274300	2.05668100	-1.90943900
H	-1.04276600	2.09556300	-0.47354900
H	2.99135100	-4.63780900	0.33701300
H	4.68901800	-4.33794700	0.88561600
H	4.14912400	0.77993400	3.85577300
H	3.68091200	0.82337900	2.11021800
H	2.76711900	-0.18502300	3.22435600
H	4.46505800	-2.48213300	-2.59013000
H	5.38492900	-3.75040500	-1.76628500
H	3.68846200	-4.04874100	-2.24210400
H	2.44127500	-2.65558300	3.69134400
H	2.24677700	-4.28191600	3.00533200
H	3.85419600	-3.72509000	3.53173800
H	-3.24368600	5.09397500	1.37272600
H	-2.11992300	5.64028300	0.08366300
H	7.63926500	0.88527300	-0.72570300
H	7.21431300	1.79188400	0.76394000
H	-2.48969600	-1.89104700	0.44498700
H	-1.32675800	-4.06603600	0.40376200
H	-1.62310700	-5.61839200	-1.53559800
H	-3.11077100	-4.95054000	-3.42469100
H	-4.32267800	-2.79740100	-3.35183500
H	-5.69485000	-3.00663600	0.15519600
H	-5.98844600	-4.14330900	2.34127600
H	-5.61477000	-2.88655500	4.46619000
H	-4.95689200	-0.48060300	4.37536300
H	-4.64559900	0.64708300	2.18829500
H	1.17353300	0.31090200	0.56546300
H	-0.84571300	1.33215800	1.59650800
H	-2.34399500	-0.02587100	3.03227500
H	-1.82983900	-2.43017600	3.43443000
H	0.18943800	-3.45361400	2.41991100
H	3.48874200	-0.22165900	-2.06153500
H	2.37398700	0.86254200	-3.97738300
H	0.07754600	0.16132100	-4.66866000
H	-1.07602300	-1.67498900	-3.41300400
H	0.02509500	-2.72594400	-1.47215800

Cartesian coordinates of the optimized geometry of *cis*-**14**-QC at B3LYP-D3BJ/def2-SVP level of theory:

C	-2.26096300	-0.63469200	-1.15065100
C	-2.10462600	-0.75085500	0.37418300
C	-2.15882300	0.67432500	0.93644600
C	-3.45241700	0.00099800	0.55496300
C	-3.57815600	0.11253100	-0.99742000
C	-2.32792100	0.88481600	-1.38871900
C	-1.76524500	1.61313200	-0.18831900
C	-2.15850200	1.43693300	-2.77666900
C	-1.80075800	0.99189800	2.36583600
C	-1.65763500	-1.55032700	-2.15179100
C	-1.67354300	-4.36015400	0.80737300
C	3.45030800	-1.01232200	-2.41602700
O	-2.23282200	-1.93039900	-3.15419200
C	-0.19736300	-1.87294100	-1.92233000
C	0.07809900	-5.37629300	-0.70064900
C	-0.23962400	-4.69675100	0.48338600
C	0.79404300	-4.30896200	1.34386200
C	2.13206100	-4.55278800	1.00431700
C	2.43969500	-5.25654900	-0.17287500
C	1.40856500	-5.66957700	-1.01393600

N	3.10652300	-4.19403000	1.98323100
N	4.21746800	-3.70475200	1.71498900
C	4.60155200	-3.27057300	0.41285600
C	3.81990100	-2.40128700	-0.35991500
C	4.29172300	-1.95125100	-1.59757400
C	5.54793300	-2.37187800	-2.05485500
C	6.33817200	-3.21752600	-1.27094200
C	5.87887500	-3.64822600	-0.02783600
C	2.80322800	2.40510100	-1.21311900
C	4.08496500	2.87957800	-0.50581200
C	2.95112200	2.36385600	0.33930200
C	1.77488600	3.54208700	-1.07933700
C	2.56001200	4.64771300	-0.34588800
C	1.91157400	3.52581600	0.44787900
C	4.04248400	4.39467000	-0.49611500
C	0.70800700	3.91070300	-2.04621200
O	-0.37115200	4.35839500	-1.69993600
C	1.07351900	3.78228000	-3.50787200
C	5.42361400	2.19875400	-0.64895700
C	2.02910000	6.05234500	-0.26637000
C	-4.87150700	0.11537000	-1.71485900
C	-5.11503000	-0.67787700	-2.84627300
C	-6.35439200	-0.62094100	-3.49067800
C	-7.36659200	0.21395600	-3.01151400
C	-7.13276900	1.00095600	-1.87916800
C	-5.89539200	0.95278700	-1.23780900
C	-4.56297500	-0.42488400	1.43425900
C	-5.18773500	-1.66250000	1.20520900
C	-6.26902100	-2.06664800	1.98703900
C	-6.74474000	-1.23831700	3.00904600
C	-6.13433000	-0.00339700	3.24023300
C	-5.05190300	0.40243400	2.45528600
C	-1.82421200	-1.99122500	1.10074000
O	-1.49538900	-2.09636000	2.26422500
O	-2.01613700	-3.06126000	0.29043700
C	2.55727100	1.16237600	-1.94851000
O	1.50961700	0.89521600	-2.50736500
O	3.59634800	0.31494000	-1.87915900
C	0.99056000	3.72764700	1.59058600
C	-0.29685900	4.26813100	1.43855000
C	-1.09663200	4.51353800	2.55657700
C	-0.63836500	4.20594900	3.83985200
C	0.63460400	3.65175300	3.99874100
C	1.44421000	3.42016900	2.88575200
C	2.89983100	1.13560900	1.16512900
C	3.99371300	0.70960900	1.93289600
C	3.88512800	-0.40902200	2.76360900
C	2.67491000	-1.09921800	2.85838900
C	1.57786900	-0.68603800	2.09580600
C	1.69711800	0.41413100	1.24622700
H	-2.22464800	2.60599200	-0.06936400
H	-0.67777700	1.75767700	-0.26432700
H	-2.57706800	2.45316700	-2.83559800
H	-1.08883500	1.49388600	-3.02845700
H	-2.65965000	0.80777400	-3.52482900
H	-2.26749700	1.93943600	2.66952500
H	-2.11782200	0.19586300	3.04876700
H	-0.71521500	1.12142100	2.47449800
H	-2.36080200	-5.05465800	0.30775800
H	-1.85325300	-4.37265100	1.88994900
H	3.77600600	-0.99769800	-3.46762600
H	2.39235700	-1.29459200	-2.38783800
H	0.05290100	-2.83965400	-2.37789400
H	0.37117300	-1.07258000	-2.42243700
H	0.09181700	-1.87030900	-0.86371000
H	-0.72321200	-5.68919900	-1.37452200

H	0.56873400	-3.80356200	2.28377400
H	3.47605400	-5.48835700	-0.41768600
H	1.64422200	-6.22447700	-1.92492700
H	2.84911500	-2.06993000	0.00991600
H	5.91279700	-2.02402200	-3.02441100
H	7.32234800	-3.53121700	-1.62613500
H	6.48638000	-4.28981600	0.61335900
H	4.61018800	4.80665300	0.35524600
H	4.44794600	4.82416900	-1.42744400
H	2.12351900	4.06419900	-3.68384500
H	0.96248200	2.72981100	-3.80836100
H	0.39912700	4.40941500	-4.10519800
H	6.11728900	2.58602100	0.11430100
H	5.35508800	1.11211000	-0.54703000
H	5.85641300	2.41935900	-1.63751400
H	2.39287000	6.54241500	0.65067700
H	2.37068400	6.64469100	-1.13023100
H	0.93269300	6.07725400	-0.25042700
H	-4.32156500	-1.33216000	-3.20963300
H	-6.52996800	-1.24218900	-4.37270400
H	-8.33613600	0.24983500	-3.51457600
H	-7.91894700	1.65462900	-1.49305800
H	-5.71923800	1.56542500	-0.35121100
H	-4.81615300	-2.30490700	0.40408100
H	-6.74584900	-3.03144900	1.79786800
H	-7.59222300	-1.55471400	3.62189600
H	-6.50417800	0.65018100	4.03406600
H	-4.58442800	1.37237100	2.63025300
H	-0.66299500	4.48105300	0.43477900
H	-2.09428800	4.93792700	2.41841700
H	-1.27173900	4.38831500	4.71123000
H	1.00295600	3.39862900	4.99575700
H	2.43772400	2.99216300	3.02439400
H	4.93167800	1.26450500	1.89167400
H	4.74803500	-0.74242500	3.34393900
H	2.59102400	-1.96881900	3.51181800
H	0.62343000	-1.20709100	2.17924100
H	0.84103900	0.73371400	0.65221500

Cartesian coordinates of the optimized geometry of *trans*-**16**-NBD at B3LYP-D3BJ/def2-SVP level of theory:

C	3.88841400	-0.00516700	-0.38890500
C	3.22393000	-0.99592000	0.58279200
C	3.54186500	-0.51009000	1.99737200
C	2.43983200	0.18551800	1.23438000
C	3.14299200	1.16783300	0.24451200
C	4.61299800	0.98783000	0.55491600
C	4.79291300	0.34268500	1.91124300
C	5.62325000	1.95957200	0.01160800
C	3.31969000	-1.37353900	3.21374800
C	4.48053800	-0.30339900	-1.71463500
C	5.13678600	-1.66412000	-1.85210700
O	4.52016000	0.50442400	-2.62509100
C	1.34768300	-3.33798200	-1.52208300
C	-0.08882800	-2.95765500	-1.27853900
C	-0.75850700	-3.38414800	-0.12363500
C	-2.05705800	-2.95252900	0.13495100
C	-2.68960500	-2.05995500	-0.74051700
C	-2.03764300	-1.65339800	-1.91898500
C	-0.75003800	-2.10416200	-2.17932500
N	-3.95988300	-1.59296100	-0.34622600
N	-4.32977800	-0.53176800	-0.90125200
C	-5.59833000	-0.04855100	-0.51873200
C	-6.45371900	-0.69687400	0.39365200

C	-7.68199000	-0.13146700	0.70648300
C	-8.09928000	1.08542000	0.12888300
C	-7.23829500	1.71928600	-0.77763400
C	-6.00137900	1.16093100	-1.10030100
C	-9.43851200	1.67536600	0.48436900
C	2.51250100	2.40332100	-0.27272200
C	2.53821200	2.75908900	-1.62940800
C	1.92875800	3.94233500	-2.05691000
C	1.28098300	4.77726400	-1.14348200
C	1.24816400	4.42548500	0.20967200
C	1.85965100	3.24883200	0.64118100
C	0.98554700	0.22954000	1.50536200
C	0.09398900	0.34944800	0.42400900
C	-1.27598300	0.47524300	0.64206300
C	-1.78403800	0.46024900	1.94471900
C	-0.90960800	0.33651000	3.02621800
C	0.46767900	0.23318100	2.80892300
C	2.49557000	-2.22510800	0.24312500
O	2.10076800	-3.05709500	1.02986200
O	2.23451700	-2.28439700	-1.08480600
H	4.82406700	1.09735700	2.71540900
H	5.71102000	-0.26579800	1.96677500
H	5.65887800	2.85952700	0.64611300
H	6.62979600	1.51170700	-0.00571500
H	5.37142800	2.27285000	-1.00984800
H	3.31891900	-0.75391900	4.12474400
H	2.38141900	-1.93410900	3.15376800
H	4.13701800	-2.10602900	3.30575200
H	5.49308200	-2.05295200	-0.88638700
H	4.39493300	-2.37617400	-2.24280400
H	5.96583300	-1.58856100	-2.56858400
H	1.60944500	-4.26806800	-1.00067500
H	1.55622600	-3.44198600	-2.59485400
H	-0.23990200	-4.02890700	0.58656100
H	-2.58993400	-3.25730800	1.03707200
H	-2.55464000	-0.97541400	-2.59822400
H	-0.23518400	-1.78080200	-3.08823600
H	-6.12564200	-1.63712500	0.83739300
H	-8.34319400	-0.63837800	1.41534400
H	-7.54183600	2.66360300	-1.23666600
H	-5.32287100	1.64621200	-1.80456300
H	-9.61189400	2.62988900	-0.03282200
H	-10.25850700	0.98899000	0.21452900
H	-9.51742700	1.85664200	1.56920600
H	3.04750500	2.10452100	-2.33717800
H	1.95767700	4.20880100	-3.11645500
H	0.79936000	5.69740700	-1.48343500
H	0.73971100	5.06878600	0.93203900
H	1.82056600	2.97236000	1.69682000
H	0.48416700	0.35119900	-0.59477200
H	-1.95739000	0.58033300	-0.20299400
H	-2.86089700	0.54341600	2.10979400
H	-1.29769400	0.33038800	4.04779500
H	1.14524300	0.16595100	3.66017000

Cartesian coordinates of the optimized geometry of *cis*-**16**-QC at B3LYP-D3BJ/def2-SVP level of theory:

C	2.65530000	-0.83907000	-1.03364700
C	1.92081800	-1.74872900	-0.02769100
C	2.80400500	-1.79675600	1.23662200
C	1.91999800	-0.59946100	1.01435800
C	2.65902300	0.30646900	-0.02325500
C	3.95003200	-0.45123100	-0.29174000
C	4.21607200	-1.44907400	0.81068900

C	5.08340200	0.21807700	-1.01927400
C	2.60571500	-2.81037500	2.33517300
C	2.69602800	-0.94499800	-2.51911200
C	2.86168500	-2.34112700	-3.07617500
O	2.69149400	0.03020900	-3.24772600
C	-1.03182600	-3.88404300	0.50573700
C	-2.22391300	-3.04364900	0.13338200
C	-2.85854800	-3.20903900	-1.10434500
C	-3.95888100	-2.42881400	-1.45113300
C	-4.39151500	-1.41522300	-0.58634300
C	-3.77129400	-1.24892100	0.66175600
C	-2.70437700	-2.06831700	1.01848300
N	-5.54260000	-0.65848600	-0.95210900
N	-5.55610500	0.58535800	-0.97133300
C	-4.38405900	1.38576700	-0.82216600
C	-4.51823700	2.56285600	-0.07343900
C	-3.43771500	3.43296400	0.06587500
C	-2.21783000	3.18502200	-0.58202000
C	-2.12164200	2.03723500	-1.38869500
C	-3.17711300	1.13705000	-1.50037900
C	-1.03004100	4.08621800	-0.39476400
C	2.52427100	1.77942600	-0.09486100
C	2.55194800	2.51200600	1.10558900
C	2.51241000	3.90676100	1.09004400
C	2.44918800	4.59419700	-0.12600000
C	2.40602100	3.87308700	-1.32131100
C	2.43179300	2.47563500	-1.31067600
C	0.77623200	-0.13838100	1.83249600
C	0.79721500	-0.21338900	3.23358400
C	-0.27368700	0.27332100	3.98797800
C	-1.37286900	0.85476500	3.35034400
C	-1.39668100	0.94565400	1.95534200
C	-0.33199000	0.45032400	1.20425400
C	0.74171000	-2.56251600	-0.35591200
O	0.31085400	-2.72399700	-1.47855900
O	0.15504900	-3.09772100	0.73273700
H	4.78498300	-0.99588900	1.64043700
H	4.76892200	-2.33295900	0.45049700
H	5.66662900	0.83961500	-0.32129100
H	5.76132500	-0.53032000	-1.46024300
H	4.72087200	0.86673200	-1.82698400
H	3.17923800	-2.51208300	3.22728800
H	1.55291300	-2.92151900	2.61159500
H	2.97885100	-3.79574300	2.01321700
H	3.28306000	-2.27711800	-4.08802200
H	3.50255100	-2.95808500	-2.42685100
H	1.87301500	-2.82136300	-3.10962300
H	-0.82670000	-4.63051700	-0.27435700
H	-1.18890200	-4.39666200	1.46484100
H	-2.47766900	-3.95324700	-1.80691900
H	-4.47132100	-2.56596000	-2.40528300
H	-4.12692400	-0.47605800	1.34379200
H	-2.21808900	-1.92600400	1.98592700
H	-5.47872800	2.77199100	0.40185000
H	-3.54434600	4.32955500	0.68195900
H	-1.19097100	1.84025400	-1.92795400
H	-3.07022900	0.24878400	-2.12238100
H	-0.28071400	3.60818300	0.25769500
H	-0.52304300	4.29387500	-1.34829000
H	-1.31313000	5.04412500	0.06534100
H	2.60771900	1.98282400	2.05870000
H	2.53489400	4.45944000	2.03248900
H	2.42201200	5.68639300	-0.13914100
H	2.34488400	4.40081000	-2.27641400
H	2.39693400	1.90645100	-2.24027500
H	1.66264300	-0.64507600	3.73772800

H	-0.24402900	0.20563600	5.07823000
H	-2.20855300	1.24114600	3.93878600
H	-2.24754600	1.39850500	1.44760000
H	-0.36268100	0.51592700	0.11592800

Cartesian coordinates of the optimized geometry of TS-**16**$_{(cis\text{-}trans)}$ at B3LYP-D3BJ/def2-SVP level of theory:

C	2.74454900	0.36071100	-1.03590500
C	2.61730300	-0.67872500	0.09770600
C	3.38739900	-0.07280500	1.29557400
C	1.96782300	0.34786400	1.04057600
C	2.07444100	1.39736300	-0.10993300
C	3.57099600	1.48125400	-0.44383300
C	4.35088900	0.95784300	0.74274700
C	4.12094700	2.61913400	-1.26206200
C	3.79963800	-0.92833500	2.46586300
C	2.38144200	0.17549300	-2.47342300
C	1.16204300	-0.67367300	-2.76753500
O	2.98371100	0.74702700	-3.36181800
C	1.34064400	-4.05581800	0.77300300
C	-0.00453700	-3.89809600	0.12150000
C	-1.08343700	-3.42536100	0.89137100
C	-2.26014500	-3.01448700	0.29176300
C	-2.38322800	-3.04388900	-1.12609900
C	-1.33240700	-3.61232900	-1.89319900
C	-0.16000500	-4.01793900	-1.27298800
N	-3.42196400	-2.45566900	-1.78532600
N	-4.35696000	-1.86488800	-1.10568200
C	-4.26493000	-0.53955700	-0.80443600
C	-5.29023900	0.03783900	-0.01078700
C	-5.17851100	1.34851100	0.42944700
C	-4.06440200	2.14362100	0.09654100
C	-3.06529200	1.58064700	-0.72583200
C	-3.15538400	0.27381200	-1.17675900
C	-3.92247300	3.54438700	0.62420600
C	1.08740700	2.46398700	-0.37875700
C	0.56623900	3.19153300	0.70769900
C	-0.31133400	4.25562500	0.50182300
C	-0.69207200	4.61564400	-0.79452800
C	-0.19354700	3.89246600	-1.88025400
C	0.68343400	2.82373600	-1.67572300
C	0.78008000	0.20706700	1.91396400
C	0.86442700	0.37645000	3.30423500
C	-0.28472800	0.32775000	4.09800600
C	-1.53609000	0.12559800	3.50922900
C	-1.63223900	-0.03527300	2.12342500
C	-0.48206800	-0.00223500	1.33622200
C	2.40340800	-2.11824800	-0.09914400
O	2.65305200	-2.69946100	-1.13367600
O	1.89005500	-2.73139200	0.98731400
H	4.55666800	1.75300400	1.47924300
H	5.30823500	0.50255400	0.43962600
H	4.32884400	3.48356800	-0.61078200
H	5.05778600	2.31784000	-1.75398500
H	3.42898200	2.93579800	-2.04998100
H	4.08825300	-0.28924600	3.31551000
H	2.99515900	-1.59884100	2.78793200
H	4.67415500	-1.54395400	2.20082100
H	1.47834500	-1.72025200	-2.88068900
H	0.42991200	-0.64550900	-1.94659600
H	0.70342900	-0.33150400	-3.70511000
H	2.03351000	-4.64760900	0.16135000
H	1.26508800	-4.48599600	1.77973500
H	-0.97134700	-3.33452300	1.97465200

H	-3.08611400	-2.62380100	0.88540800
H	-1.44954800	-3.65685000	-2.97727500
H	0.67578000	-4.38327700	-1.87191200
H	-6.14516700	-0.58466000	0.25912800
H	-5.96780000	1.77326800	1.05541400
H	-2.19675800	2.18397400	-0.99897900
H	-2.37621000	-0.15341700	-1.80932900
H	-3.12364600	3.59313000	1.38347900
H	-3.63882400	4.24744700	-0.17397600
H	-4.85459000	3.89940700	1.08729700
H	0.85847600	2.92159900	1.72325500
H	-0.69722000	4.81052900	1.36026800
H	-1.37502600	5.45261600	-0.95654300
H	-0.48804200	4.15797100	-2.89828000
H	1.06403900	2.28030700	-2.53955100
H	1.83517200	0.56256000	3.76588600
H	-0.20274200	0.45952500	5.17971200
H	-2.43656200	0.09925700	4.12754700
H	-2.60479800	-0.17959100	1.65063700
H	-0.56577700	-0.13019400	0.25637700

Cartesian coordinates of the optimized geometry of *trans*-**16**-QC at B3LYP-D3BJ/def2-SVP level of theory:

C	3.88841400	-0.00516700	-0.38890500
C	3.22393000	-0.99592000	0.58279200
C	3.54186500	-0.51009000	1.99737200
C	2.43983200	0.18551800	1.23438000
C	3.14299200	1.16783300	0.24451200
C	4.61299800	0.98783000	0.55491600
C	4.79291300	0.34268500	1.91124300
C	5.62325000	1.95957200	0.01160800
C	3.31969000	-1.37353900	3.21374800
C	4.48053800	-0.30339900	-1.71463500
C	5.13678600	-1.66412000	-1.85210700
O	4.52016000	0.50442400	-2.62509100
C	1.34768300	-3.33798200	-1.52208300
C	-0.08882800	-2.95765500	-1.27853900
C	-0.75850700	-3.38414800	-0.12363500
C	-2.05705800	-2.95252900	0.13495100
C	-2.68960500	-2.05995500	-0.74051700
C	-2.03764300	-1.65339800	-1.91898500
C	-0.75003800	-2.10416200	-2.17932500
N	-3.95988300	-1.59296100	-0.34622600
N	-4.32977800	-0.53176800	-0.90125200
C	-5.59833000	-0.04855100	-0.51873200
C	-6.45371900	-0.69687400	0.39365200
C	-7.68199000	-0.13146700	0.70648300
C	-8.09928000	1.08542000	0.12888300
C	-7.23829500	1.71928600	-0.77763400
C	-6.00137900	1.16093100	-1.10030100
C	-9.43851200	1.67536600	0.48436900
C	2.51250100	2.40332100	-0.27272200
C	2.53821200	2.75908900	-1.62940800
C	1.92875800	3.94233500	-2.05691000
C	1.28098300	4.77726400	-1.14348200
C	1.24816400	4.42548500	0.20967200
C	1.85965100	3.24883200	0.64118100
C	0.98554700	0.22954000	1.50536200
C	0.09398900	0.34944800	0.42400900
C	-1.27598300	0.47524300	0.64206300
C	-1.78403800	0.46024900	1.94471900
C	-0.90960800	0.33651000	3.02621800
C	0.46767900	0.23318100	2.80892300
C	2.49557000	-2.22510800	0.24312500

O	2.10076800	-3.05709500	1.02986200
O	2.23451700	-2.28439700	-1.08480600
H	4.82406700	1.09735700	2.71540900
H	5.71102000	-0.26579800	1.96677500
H	5.65887800	2.85952700	0.64611300
H	6.62979600	1.51170700	-0.00571500
H	5.37142800	2.27285000	-1.00984800
H	3.31891900	-0.75391900	4.12474400
H	2.38141900	-1.93410900	3.15376800
H	4.13701800	-2.10602900	3.30575200
H	5.49308200	-2.05295200	-0.88638700
H	4.39493300	-2.37617400	-2.24280400
H	5.96583300	-1.58856100	-2.56858400
H	1.60944500	-4.26806800	-1.00067500
H	1.55622600	-3.44198600	-2.59485400
H	-0.23990200	-4.02890700	0.58656100
H	-2.58993400	-3.25730800	1.03707200
H	-2.55464000	-0.97541400	-2.59822400
H	-0.23518400	-1.78080200	-3.08823600
H	-6.12564200	-1.63712500	0.83739300
H	-8.34319400	-0.63837800	1.41534400
H	-7.54183600	2.66360300	-1.23666600
H	-5.32287100	1.64621200	-1.80456300
H	-9.61189400	2.62988900	-0.03282200
H	-10.25850700	0.98899000	0.21452900
H	-9.51742700	1.85664200	1.56920600
H	3.04750500	2.10452100	-2.33717800
H	1.95767700	4.20880100	-3.11645500
H	0.79936000	5.69740700	-1.48343500
H	0.73971100	5.06878600	0.93203900
H	1.82056600	2.97236000	1.69682000
H	0.48416700	0.35119900	-0.59477200
H	-1.95739000	0.58033300	-0.20299400
H	-2.86089700	0.54341600	2.10979400
H	-1.29769400	0.33038800	4.04779500
H	1.14524300	0.16595100	3.66017000

Cartesian coordinates of the optimized geometry of TS-**16**$_{(QC\text{-}NBD)}$ at B3LYP-D3BJ/def2-SVP level of theory:

C	4.38414600	-0.70932700	0.01317900
C	3.18291400	-1.08254100	0.80140400
C	3.31905200	-0.45522500	2.19326700
C	2.52145000	0.35277800	1.19064000
C	3.46391800	1.31535000	0.52817700
C	4.80046500	0.59233700	0.69723900
C	4.70609400	0.17156200	2.19272100
C	6.11250100	1.27013200	0.34752400
C	2.78522300	-1.08406200	3.45224100
C	4.96278300	-1.27532800	-1.18095400
C	4.73773200	-2.74056800	-1.51657000
O	5.67068100	-0.58424900	-1.92346600
C	0.98181600	-3.22381400	-1.23951100
C	-0.43489800	-2.71548300	-1.18478400
C	-1.04460500	-2.20056100	-2.33638700
C	-2.33647600	-1.68057200	-2.28055600
C	-3.02579700	-1.64277100	-1.06017400
C	-2.42942500	-2.18132100	0.09478800
C	-1.15047000	-2.71618800	0.02734000
N	-4.29304900	-1.02466100	-1.08279000
N	-4.78898300	-0.77603600	0.04151200
C	-6.05655300	-0.15911700	0.02916900
C	-6.77841500	0.15426300	-1.13940900
C	-8.01947400	0.76702200	-1.03864500
C	-8.58189900	1.08663500	0.21446400

C	-7.85348600	0.76851700	1.36906800
C	-6.60531100	0.15207400	1.28074000
C	-9.93121400	1.75049500	0.29452900
C	3.14133400	2.46099700	-0.26649400
C	3.90548500	2.80112400	-1.41629500
C	3.58800800	3.91917900	-2.18049200
C	2.50929700	4.74050200	-1.82980700
C	1.73955000	4.41924500	-0.70458100
C	2.03942200	3.29884200	0.06197000
C	1.04583600	0.51557900	1.14483800
C	0.40486200	0.56933700	-0.10584600
C	-0.96304200	0.81185000	-0.19264500
C	-1.72325200	0.98206200	0.96851900
C	-1.09823700	0.92589000	2.21591000
C	0.27925400	0.70321800	2.30435600
C	2.21933400	-2.16672400	0.50311100
O	1.71363800	-2.89915800	1.32504900
O	1.92350300	-2.21441600	-0.81109800
H	4.76680400	1.04414900	2.86246300
H	5.49184400	-0.55297700	2.45934500
H	6.26302300	1.37460900	-0.72948900
H	6.15887500	2.26118700	0.82391400
H	6.94943300	0.66629500	0.73095100
H	1.78184500	-1.50035300	3.31899900
H	3.44630900	-1.90764100	3.76451500
H	2.76713600	-0.33920100	4.26458200
H	3.94633700	-2.82889000	-2.27561800
H	5.66977400	-3.13065000	-1.94989000
H	4.45133700	-3.34826500	-0.64683300
H	1.10947100	-4.11332300	-0.60839900
H	1.27446700	-3.45653400	-2.27154300
H	-0.49974900	-2.20353300	-3.28401900
H	-2.82758700	-1.26990200	-3.16468100
H	-2.98428400	-2.15036400	1.03193700
H	-0.67170400	-3.11489100	0.92247800
H	-6.33863400	-0.09449200	-2.10545500
H	-8.57626500	1.00845100	-1.94876500
H	-8.27118200	1.00680200	2.35058400
H	-6.03036600	-0.10204700	2.17340200
H	-10.22824200	1.94257900	1.33546300
H	-9.93252900	2.71335100	-0.24300800
H	-10.71002100	1.12358800	-0.17094800
H	4.70976600	2.14006600	-1.73506400
H	4.18126700	4.14619100	-3.06979200
H	2.26660200	5.61954400	-2.43128600
H	0.89553800	5.05277100	-0.42077100
H	1.43932200	3.07376900	0.94277200
H	0.99392000	0.42042400	-1.01139300
H	-1.44484200	0.84853400	-1.17044200
H	-2.80210500	1.13499700	0.89930900
H	-1.68424100	1.06022400	3.12831400
H	0.76279100	0.69065400	3.28116900

Cartesian coordinates of the optimized geometry of *trans,trans*-**17**-NBD at B3LYP-D3BJ/def2-SVP level of theory:

C	4.14230200	-0.65815000	-1.64174700
C	5.08641400	-0.51431300	-0.68928200
C	5.62385100	0.93712800	-0.79609600
C	4.41589300	1.83258300	-0.38492700
C	3.45313100	1.66804700	-1.32349100
C	4.02174300	0.68673700	-2.39868900
C	5.50963200	1.10266300	-2.33524100
C	3.34320400	0.61424500	-3.75287900
C	6.98360100	1.24378500	-0.19453400

C	1.11611200	-2.73702000	-1.69989900
C	4.20912500	-3.29030300	1.47461600
C	-0.28663000	-2.24740300	-1.47663800
C	2.75761700	-2.95543100	1.71281500
C	-1.30540500	-3.18393900	-1.25104700
C	-2.62887300	-2.77263800	-1.12274700
C	-2.95529800	-1.41161100	-1.21385100
C	-1.93353000	-0.46823300	-1.40988600
C	-0.61377000	-0.88428500	-1.54171000
C	1.76182000	-3.93437000	1.61358100
C	0.41450400	-3.59190800	1.73703400
C	0.04169300	-2.25522100	1.92878400
C	1.03865700	-1.27190800	2.06440900
C	2.37703500	-1.62362200	1.96685000
N	-4.32374800	-1.08968600	-1.10801400
N	-4.60226500	0.12968800	-1.17960300
C	-5.97677100	0.44058000	-1.11726300
N	-1.34308000	-1.99098900	1.97704400
N	-1.65925700	-0.77841900	1.95261400
C	-3.03934100	-0.50535600	2.02252900
C	-4.03799400	-1.48987500	2.11757600
C	-5.37348700	-1.11435700	2.20504900
C	-5.75615200	0.23875900	2.20231500
C	-4.75115100	1.21110400	2.08112300
C	-3.41121300	0.84758300	1.99127700
C	-6.31110700	1.79886300	-1.18269700
C	-7.64706100	2.19997000	-1.13996000
C	-8.67908600	1.25677900	-1.03301500
C	-8.32905100	-0.10681700	-0.96891800
C	-7.00306400	-0.51641900	-1.00775000
C	-7.19859200	0.64478900	2.33232700
C	-10.12407700	1.67638900	-0.96978100
C	2.03723000	2.06282900	-1.21821400
C	1.33675400	1.76323000	-0.03370600
C	-0.00638900	2.09902200	0.11377800
C	-0.68183100	2.75360600	-0.92082800
C	-0.00421500	3.05296400	-2.10589600
C	1.34156300	2.70682300	-2.25666300
C	4.32791500	2.60659400	0.86076500
C	4.75108400	2.06346500	2.08993800
C	4.60858000	2.79279800	3.27227700
C	4.05424500	4.07538400	3.25134600
C	3.64007100	4.62786500	2.03491100
C	3.77504100	3.90174100	0.85283500
C	5.25625900	-1.39180500	0.47382800
O	5.78806800	-1.06066000	1.51448500
O	4.65320200	-2.58843200	0.29715000
C	3.27646800	-1.82141700	-1.96650200
O	3.60090000	-2.70800600	-2.71560400
O	2.06162700	-1.70079000	-1.40728700
H	5.67088600	2.13983900	-2.66333100
H	6.17215400	0.41555300	-2.88380400
H	2.27727400	0.36073900	-3.65339700
H	3.41430000	1.57588000	-4.28196600
H	3.81979400	-0.15691800	-4.37651700
H	7.01543500	1.05229600	0.88348400
H	7.75518000	0.61665400	-0.66783600
H	7.24340400	2.29910500	-0.36921100
H	1.33745300	-3.60233600	-1.05673200
H	1.26189700	-3.06010000	-2.74345500
H	4.84774400	-2.99798800	2.31991100
H	4.34322900	-4.35926300	1.26591400
H	-1.05709300	-4.24556000	-1.17347200
H	-3.43499400	-3.48754300	-0.94958000
H	-2.19683000	0.58588100	-1.46122400
H	0.17566400	-0.14753500	-1.68435900

H	2.04337400	-4.97362800	1.42384200
H	-0.37564500	-4.33970800	1.65300400
H	0.73261400	-0.24238400	2.24371400
H	3.14295300	-0.85276400	2.07518800
H	-3.73751900	-2.53728700	2.12147900
H	-6.14596600	-1.88454400	2.27595900
H	-5.02848500	2.26786400	2.05297900
H	-2.62780800	1.60147100	1.90134400
H	-5.50005200	2.52453600	-1.26702500
H	-7.89478500	3.26334800	-1.19280100
H	-9.12187100	-0.85598200	-0.88518600
H	-6.72535200	-1.56918100	-0.95762800
H	-7.87189700	-0.21820800	2.23693000
H	-7.38575400	1.11527800	3.31291200
H	-7.47346500	1.37674200	1.55840200
H	-10.56314600	1.43551200	0.01348100
H	-10.24046600	2.75742500	-1.13338500
H	-10.72714800	1.14934100	-1.72698700
H	1.85945700	1.24028600	0.76585400
H	-0.53355900	1.81074300	1.02465700
H	-1.73736600	3.01327200	-0.80947400
H	-0.52614600	3.55963200	-2.92127500
H	1.86268700	2.96048800	-3.18071500
H	5.17175700	1.05735600	2.12153900
H	4.93258000	2.35147700	4.21802300
H	3.94739000	4.64405100	4.17823100
H	3.21002000	5.63210300	2.00738000
H	3.45143600	4.33345100	-0.09576700

Cartesian coordinates of the optimized geometry of *cis,cis*-**17**-QC at B3LYP-D3BJ/def2-SVP level of theory:

C	-0.13621000	1.66126600	-1.54198400
C	1.24428700	2.34702000	-1.59414200
C	0.97975700	3.83606800	-1.26630100
C	1.08598800	2.83502000	-0.15129100
C	-0.30581200	2.12661700	-0.10422500
C	-1.10552200	2.77916700	-1.24373000
C	-0.47738100	4.11436700	-1.58361000
C	-2.59029500	2.55806200	-1.33880300
C	2.03921500	4.87785500	-1.50553200
C	-6.10650700	1.35717700	0.14058400
C	-5.06685400	0.72555200	0.84156100
C	-5.02245000	-0.66008100	0.96721000
C	-6.03570600	-1.45215200	0.40938500
C	-7.11304800	-0.83267700	-0.23923000
C	-7.12747600	0.55210600	-0.39330900
N	-6.08981200	-2.86500400	0.59734800
N	-5.10554200	-3.60590000	0.41919500
C	-3.90030300	-3.16006000	-0.19896300
C	-3.91005900	-2.52813800	-1.45302000
C	-2.71040300	-2.19792600	-2.07418900
C	-1.47963900	-2.47234500	-1.45544900
C	-1.47993600	-3.13510900	-0.21839500
C	-2.67736900	-3.50199400	0.39550400
C	-0.19003200	-1.99309500	-2.06918400
C	-6.12673500	2.85238200	-0.04260700
C	3.68067100	-0.99642600	-2.80297100
C	3.20147200	-2.28234900	-3.08406300
C	3.46213100	-3.34782500	-2.22296700
C	4.16226600	-3.12282000	-1.03082100
C	4.68989100	-1.84854200	-0.76234800
C	4.45683100	-0.80084100	-1.64683700
N	4.41700000	-4.24092400	-0.18621900
N	4.18877000	-4.23409100	1.03799100

C	3.48514400	-3.18726700	1.70297100
C	3.92937900	-2.86010900	2.99129700
C	3.27859900	-1.87199900	3.72954200
C	2.13838500	-1.22450800	3.23060300
C	1.66827900	-1.60503300	1.96080700
C	2.32857300	-2.56365100	1.20079500
C	1.42439600	-0.16568300	4.02485800
C	3.31651000	0.16711400	-3.68591800
C	-0.90289400	1.53659800	1.11127100
C	-1.04740500	2.35538300	2.24693100
C	-1.62481000	1.86097700	3.41627000
C	-2.05307400	0.53018700	3.48019800
C	-1.89955000	-0.29323800	2.36428200
C	-1.34166900	0.20740600	1.18475900
C	2.10460400	2.75597900	0.91906800
C	2.32895400	3.83289800	1.78864800
C	3.27072900	3.73021000	2.81537800
C	4.00017500	2.54912100	2.98010700
C	3.78068800	1.47005600	2.11871900
C	2.83658300	1.57220700	1.09782900
C	2.46974900	1.85659000	-2.23114200
O	3.58964600	2.25755600	-1.99437500
O	2.19488200	0.88248100	-3.12332900
C	-0.47585200	0.37026200	-2.19946300
O	-1.21978300	0.25363800	-3.14342000
O	0.13604000	-0.66667200	-1.59193600
H	-0.87980200	4.92769300	-0.95608000
H	-0.62043700	4.38377900	-2.64307000
H	-3.13032100	3.28290700	-0.71008700
H	-2.93215300	2.67036500	-2.37879500
H	-2.87468700	1.55039300	-1.00609200
H	1.83552100	5.77074200	-0.89306600
H	3.03656300	4.49668800	-1.25481100
H	2.04692500	5.18713500	-2.56269500
H	-4.27210000	1.32176800	1.29642200
H	-4.20667700	-1.12667600	1.51349000
H	-7.92293100	-1.45349900	-0.62768000
H	-7.95902700	1.02309300	-0.92466500
H	-4.86064200	-2.29579200	-1.93421800
H	-2.71860400	-1.68561500	-3.03670300
H	-0.53154400	-3.36752900	0.27262300
H	-2.68262700	-4.04093800	1.34501000
H	0.66059400	-2.61097700	-1.76000000
H	-0.24620000	-1.97166000	-3.16542100
H	-5.49163300	3.35724800	0.69995200
H	-5.75083500	3.13095900	-1.04231000
H	-7.14711100	3.25681900	0.04423900
H	2.61548200	-2.45260300	-3.99118500
H	3.10604100	-4.35611100	-2.44341400
H	5.27253000	-1.68525200	0.14534400
H	4.85028000	0.19387100	-1.43099000
H	4.80101100	-3.38363800	3.38920000
H	3.65835100	-1.60239900	4.71861000
H	0.78111200	-1.11953400	1.55413600
H	1.95027000	-2.82432100	0.21379600
H	0.52368400	-0.57640100	4.51042800
H	2.07184800	0.25285400	4.80870600
H	1.09071200	0.65789100	3.37821200
H	4.15581200	0.86457900	-3.80535600
H	2.97108000	-0.17284900	-4.67030600
H	-0.70100800	3.39017100	2.20634300
H	-1.73255500	2.51332000	4.28605800
H	-2.49387400	0.13725200	4.39912200
H	-2.19939800	-1.34230700	2.40675100
H	-1.22920400	-0.44671700	0.32418500
H	1.76145400	4.75697800	1.65517600

H	3.43846300	4.57582100	3.48679100
H	4.74030200	2.46994400	3.78005000
H	4.33731600	0.54008900	2.24738700
H	2.66123600	0.72520100	0.43269400

7. ^{1}H NMR and ^{13}C NMR Spectra of the New Compounds

The field strengths and solvents of the following spectra are given in the Experimental Section.

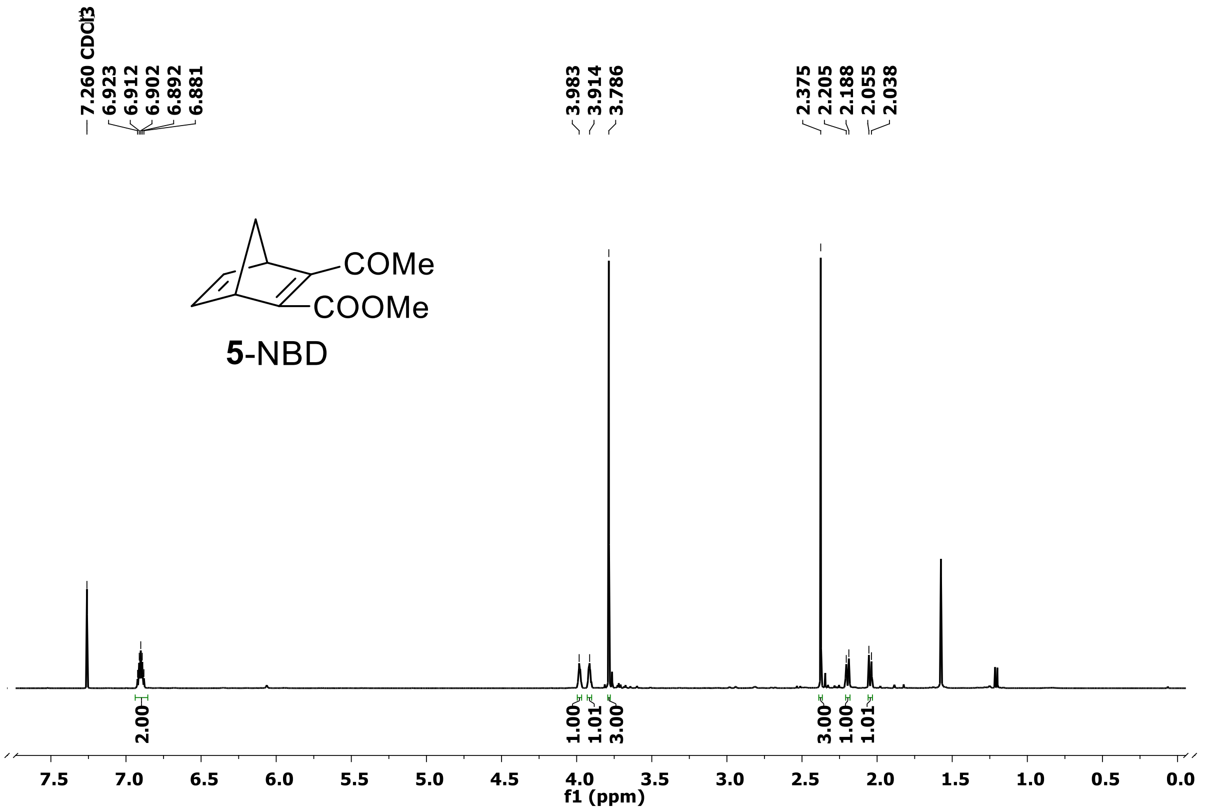

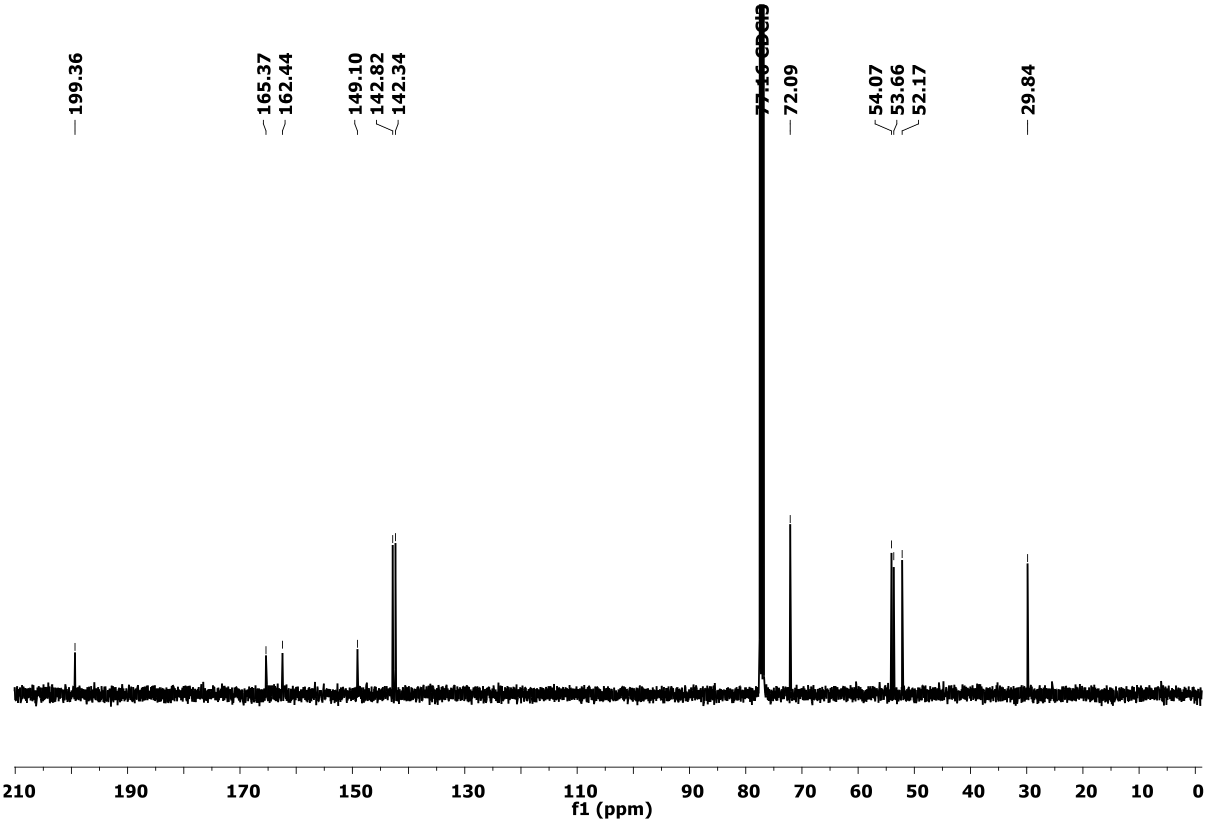

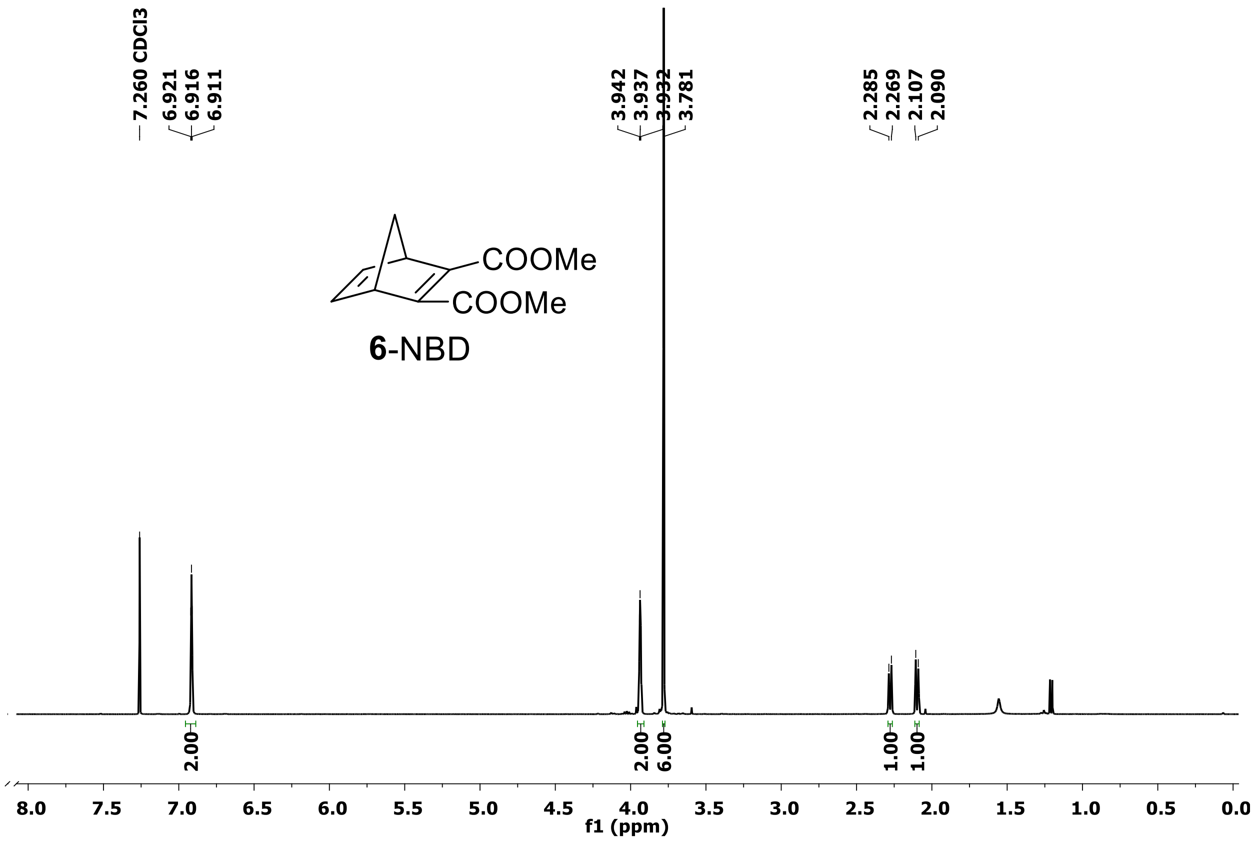
7.260 CDCl3
6.921
6.916
6.911
3.942
3.937
3.932
3.781
2.285
2.269
2.107
2.090
COOMe
COOMe
6-NBD
2.00
2.00
6.00
1.00
1.00
8.0
7.5
7.0
6.5
6.0
5.5
5.0
4.5
4.0
3.5
3.0
2.5
2.0
1.5
1.0
0.5
0.0
f1 (ppm)

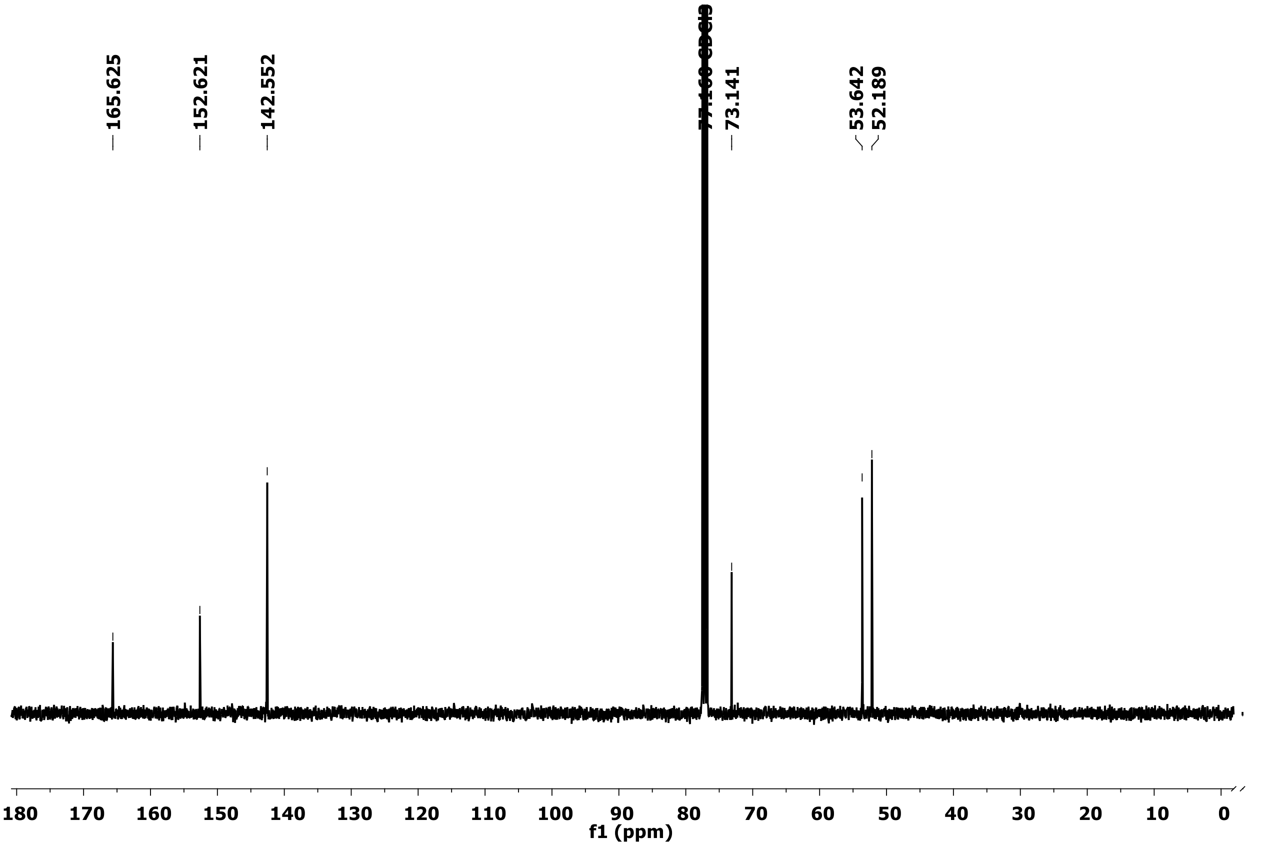
165.625
152.621
142.552
73.141
53.642
52.189
180
170
160
150
140
130
120
110
100
90
80
70
60
50
40
30
20
10
0
f1 (ppm)

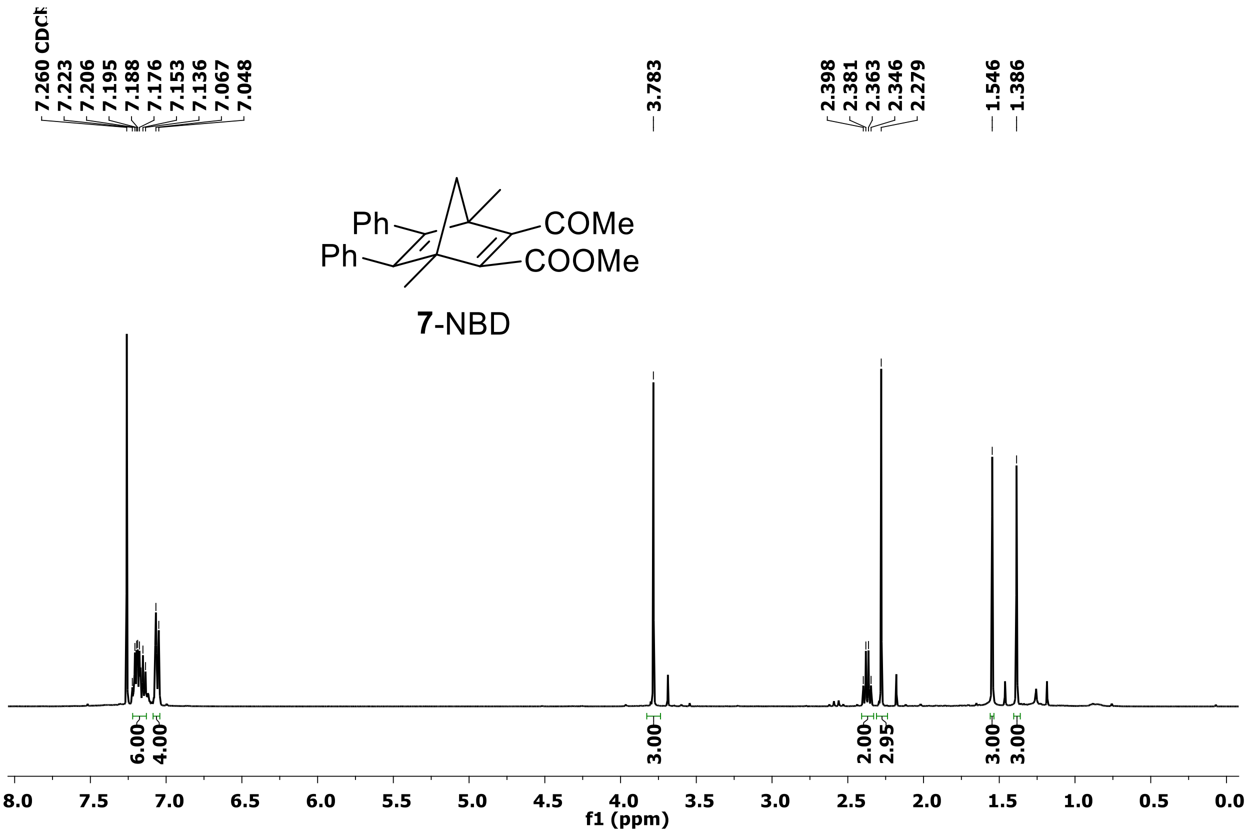
7.260 CDCl3
7.223
7.206
7.195
7.188
7.176
7.153
7.136
7.067
7.048
3.783
2.398
2.381
2.363
2.346
2.279
1.546
1.386
Ph
Ph
COMe
COOMe
7-NBD
6.00
4.00
3.00
2.00
2.95
3.00
3.00
8.0 7.5 7.0 6.5 6.0 5.5 5.0 4.5 4.0 3.5 3.0 2.5 2.0 1.5 1.0 0.5 0.0
f1 (ppm)

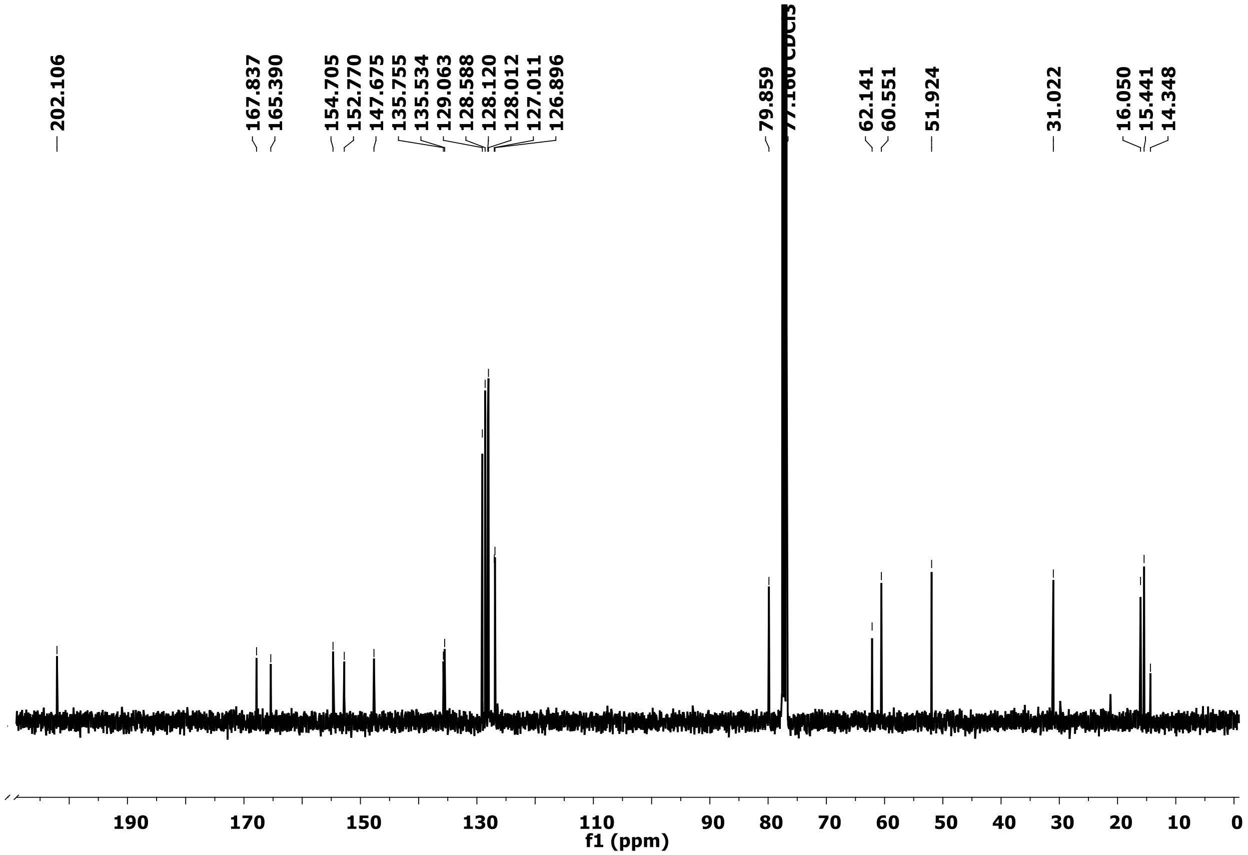
202.106
167.837
165.390
154.705
152.770
147.675
135.755
135.534
129.063
128.588
128.120
128.012
127.011
126.896
79.859
77.160 CDCl3
62.141
60.551
51.924
31.022
16.050
15.441
14.348
190 170 150 130 110 90 80 70 60 50 40 30 20 10 0
f1 (ppm)

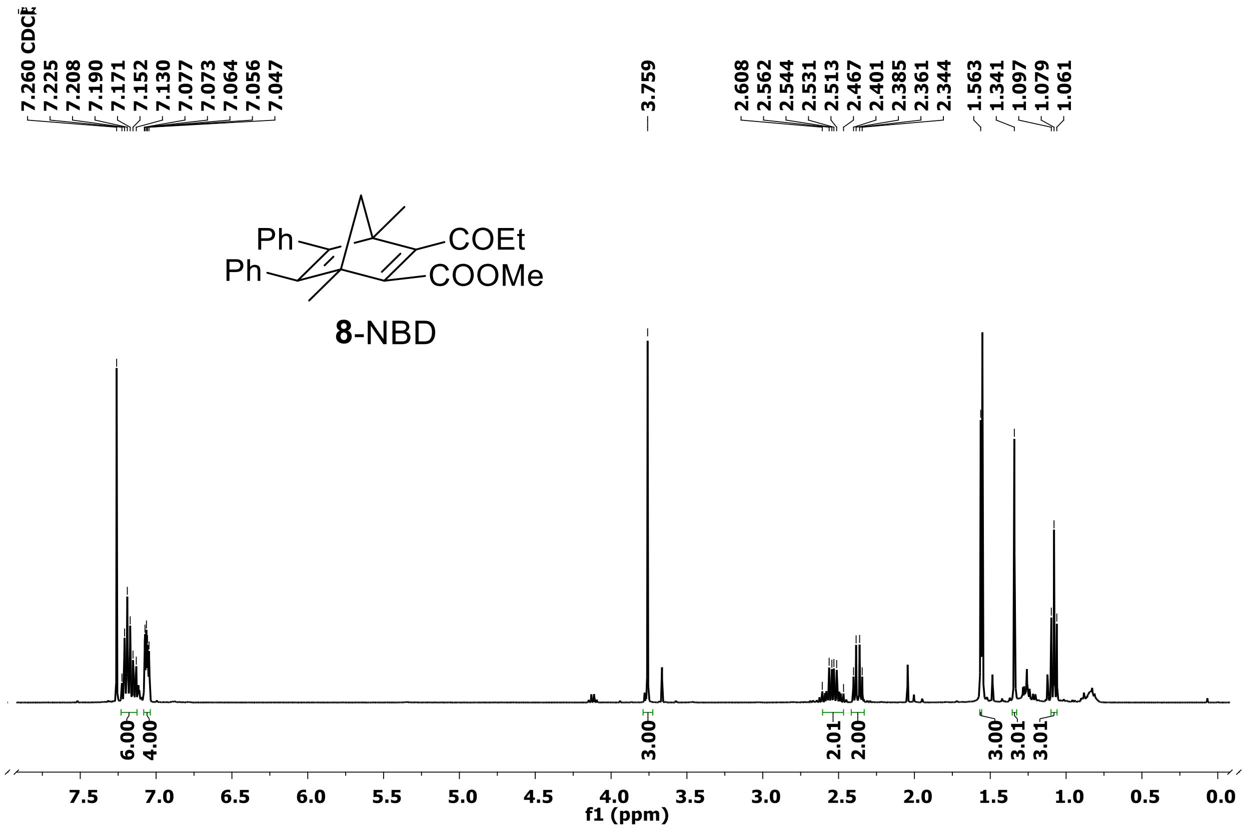
7.260 CDCl3
7.225
7.208
7.190
7.171
7.152
7.130
7.077
7.073
7.064
7.056
7.047
3.759
2.608
2.562
2.544
2.531
2.513
2.467
2.401
2.385
2.361
2.344
1.563
1.341
1.097
1.079
1.061
Ph
Ph
COEt
COOMe
8-NBD
6.00
4.00
3.00
2.01
2.00
3.00
3.01
3.01
7.5 7.0 6.5 6.0 5.5 5.0 4.5 4.0 3.5 3.0 2.5 2.0 1.5 1.0 0.5 0.0
f1 (ppm)

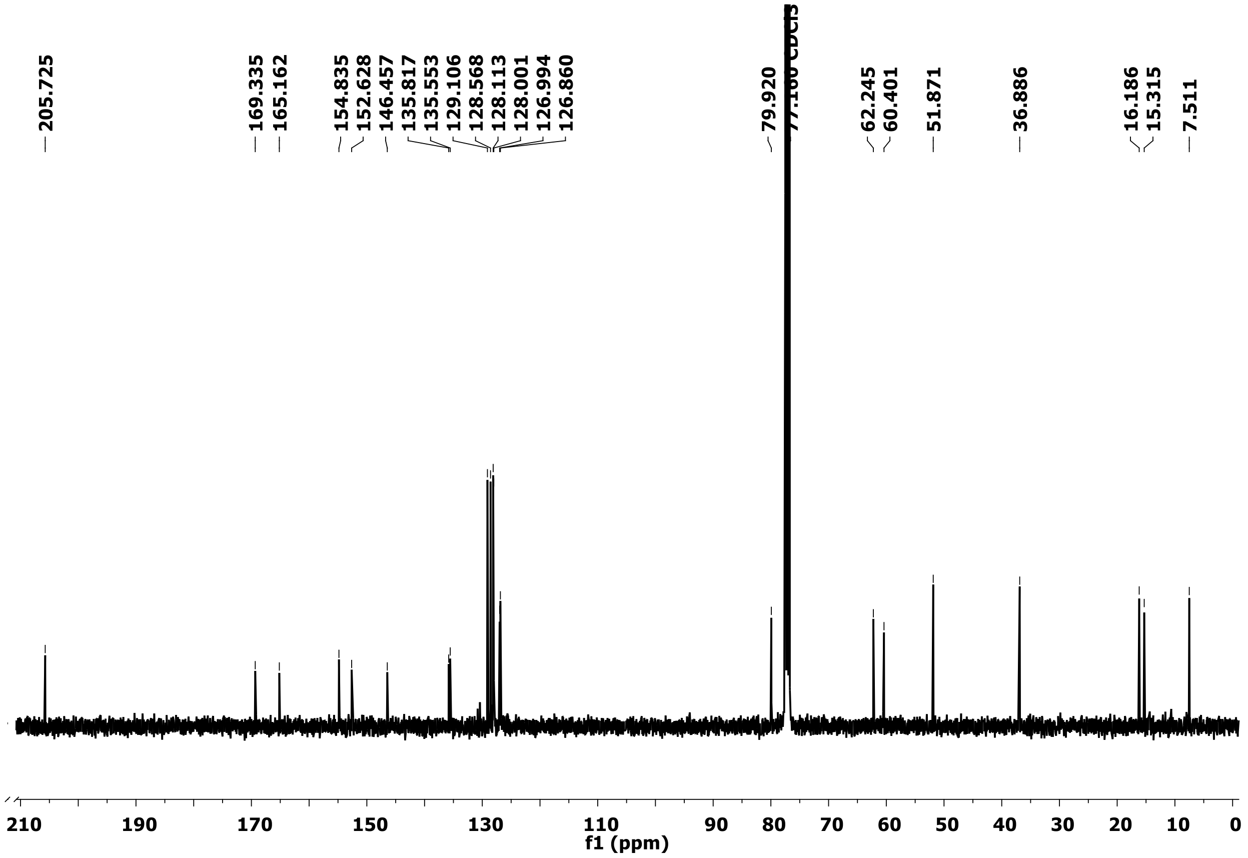
205.725
169.335
165.162
154.835
152.628
146.457
135.817
135.553
129.106
128.568
128.113
128.001
126.994
126.860
79.920
77.160 CDCl3
62.245
60.401
51.871
36.886
16.186
15.315
7.511
210 190 170 150 130 110 90 80 70 60 50 40 30 20 10 0
f1 (ppm)

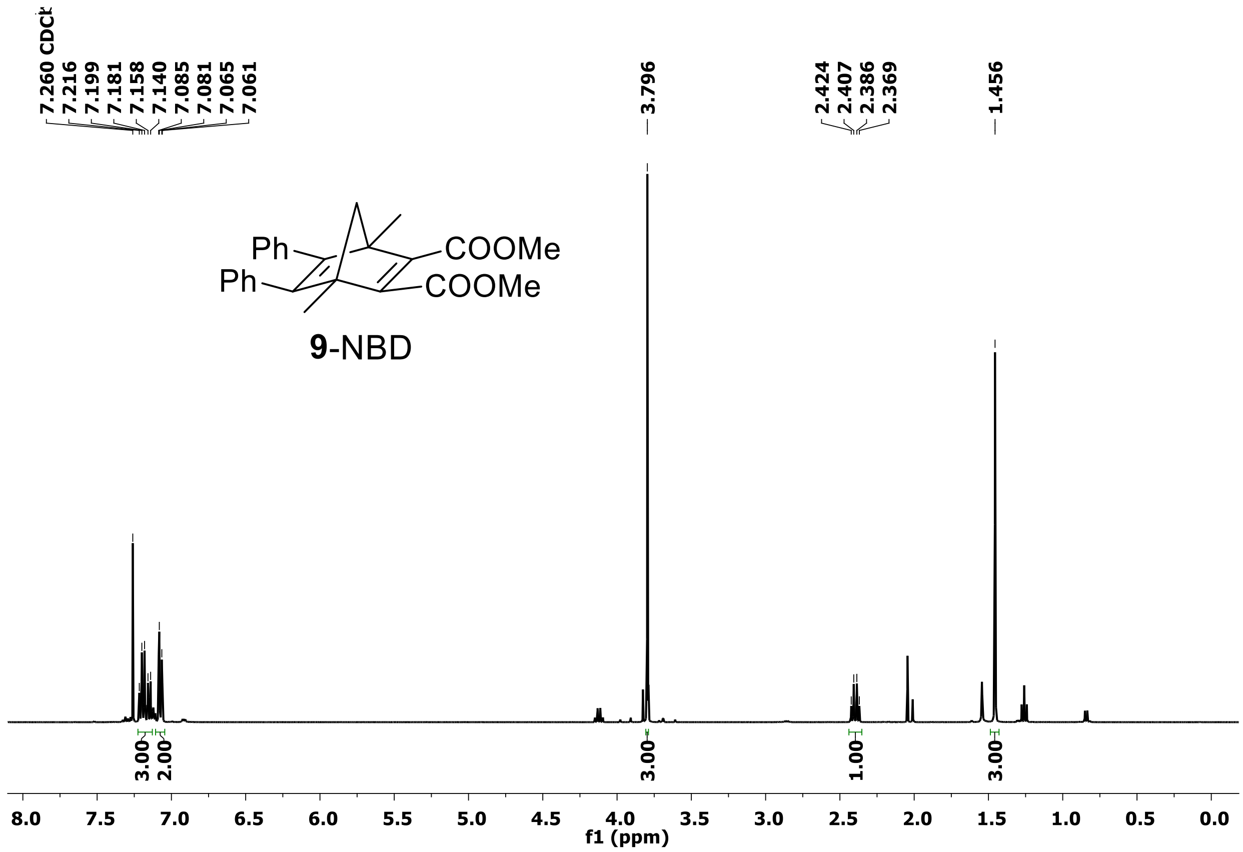
7.260 CDCl
7.216
7.199
7.181
7.158
7.140
7.085
7.081
7.065
7.061
3.796
2.424
2.407
2.386
2.369
1.456
Ph
Ph
COOMe
COOMe
9-NBD
3.00
2.00
3.00
1.00
3.00
8.0 7.5 7.0 6.5 6.0 5.5 5.0 4.5 4.0 3.5 3.0 2.5 2.0 1.5 1.0 0.5 0.0
f1 (ppm)

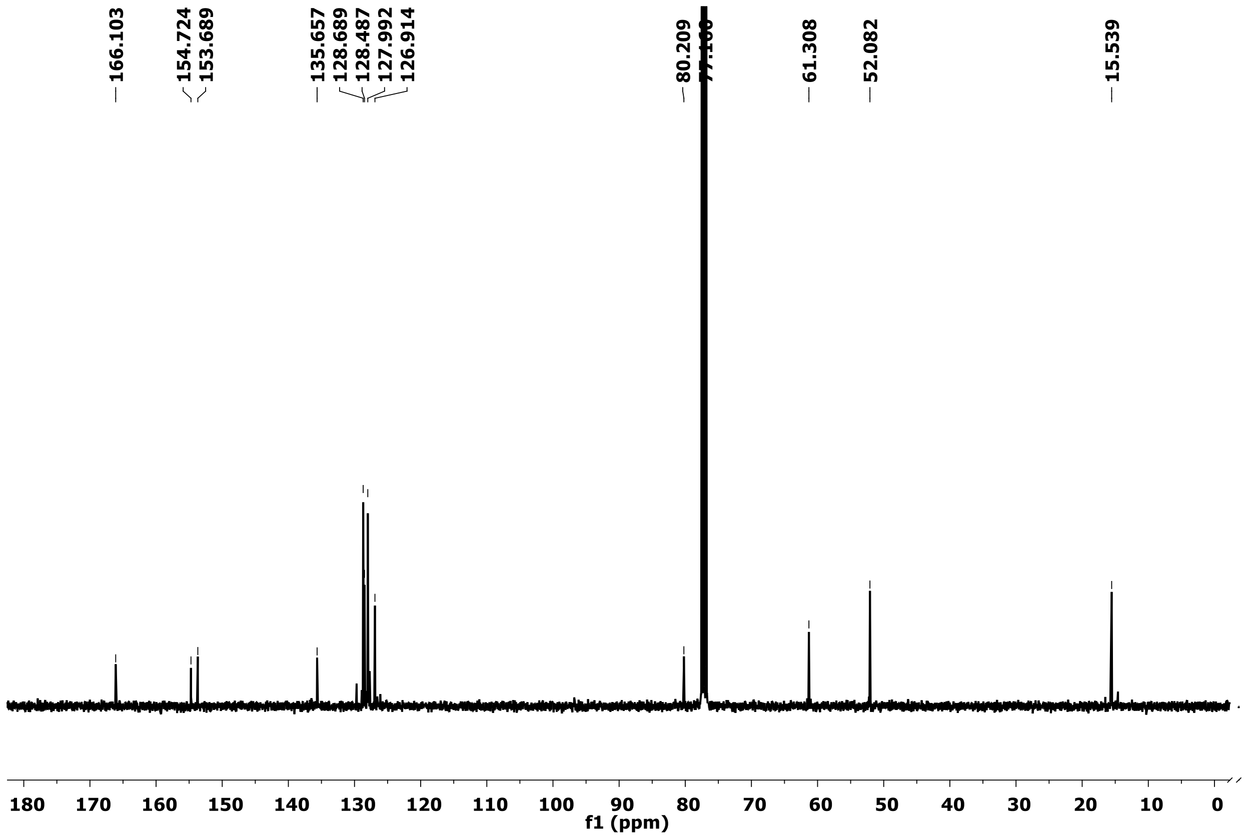
166.103
154.724
153.689
135.657
128.689
128.487
127.992
126.914
80.209
61.308
52.082
15.539
180 170 160 150 140 130 120 110 100 90 80 70 60 50 40 30 20 10 0
f1 (ppm)

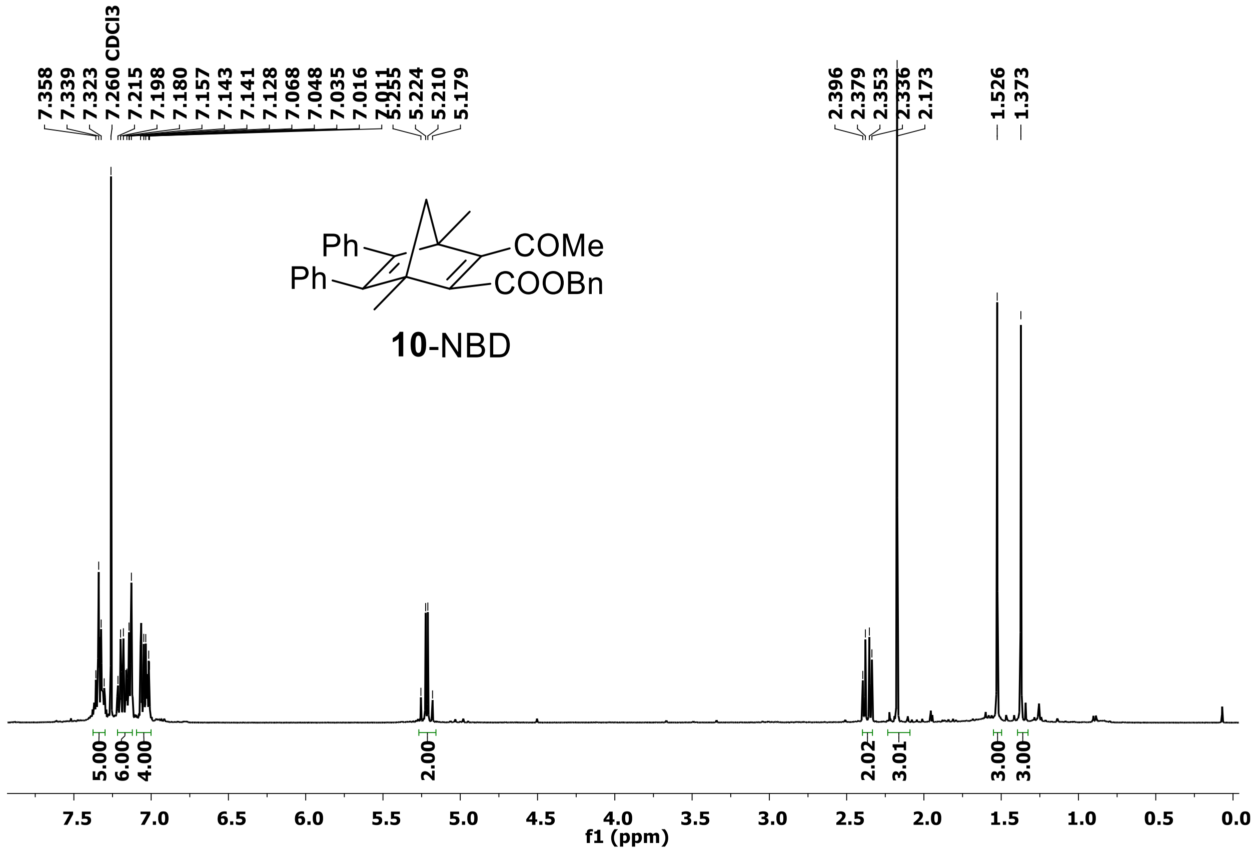
7.358
7.339
7.323
7.260 CDCl3
7.215
7.198
7.180
7.157
7.143
7.141
7.128
7.068
7.048
7.035
7.016
7.011
5.255
5.224
5.210
5.179
2.396
2.379
2.353
2.336
2.173
1.526
1.373
Ph
Ph
COMe
COOBn
10-NBD
5.00
6.00
4.00
2.00
2.02
3.01
3.00
3.00
f1 (ppm)

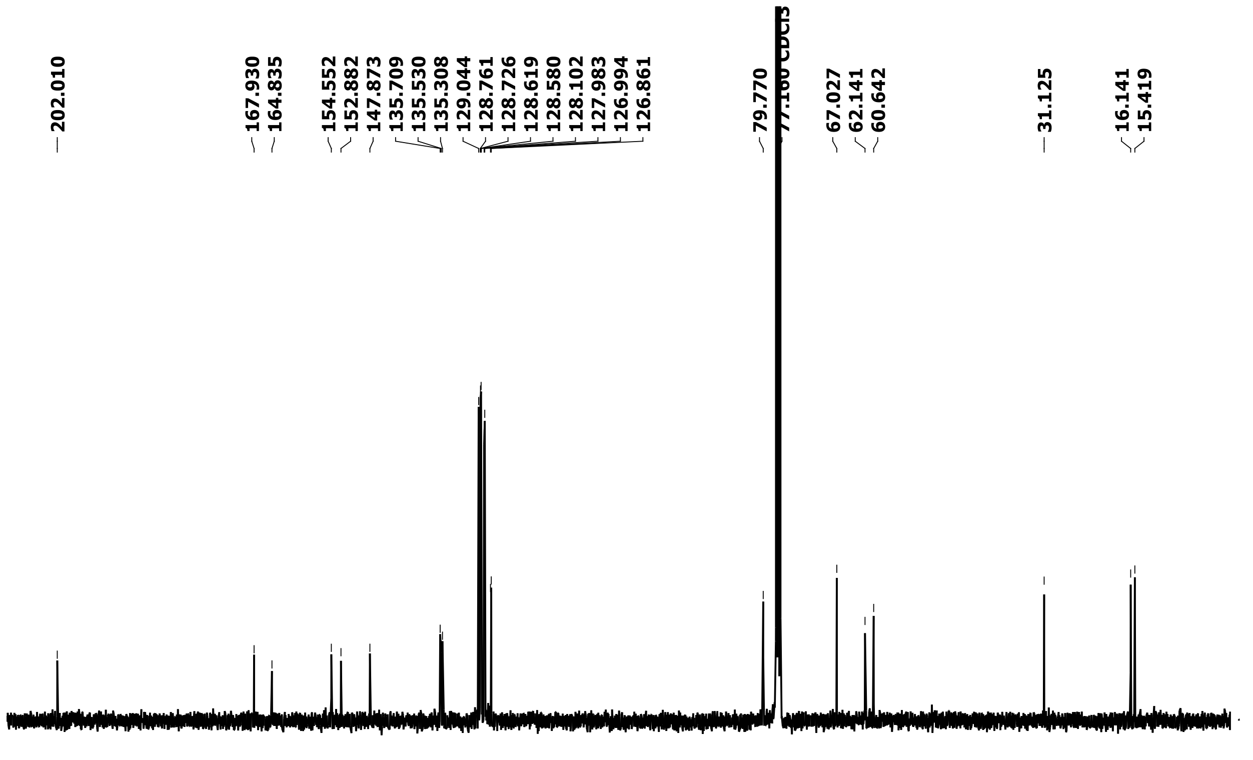
202.010
167.930
164.835
154.552
152.882
147.873
135.709
135.530
135.308
129.044
128.761
128.726
128.619
128.580
128.102
127.983
126.994
126.861
79.770
77.160 CDCl3
67.027
62.141
60.642
31.125
16.141
15.419
f1 (ppm)

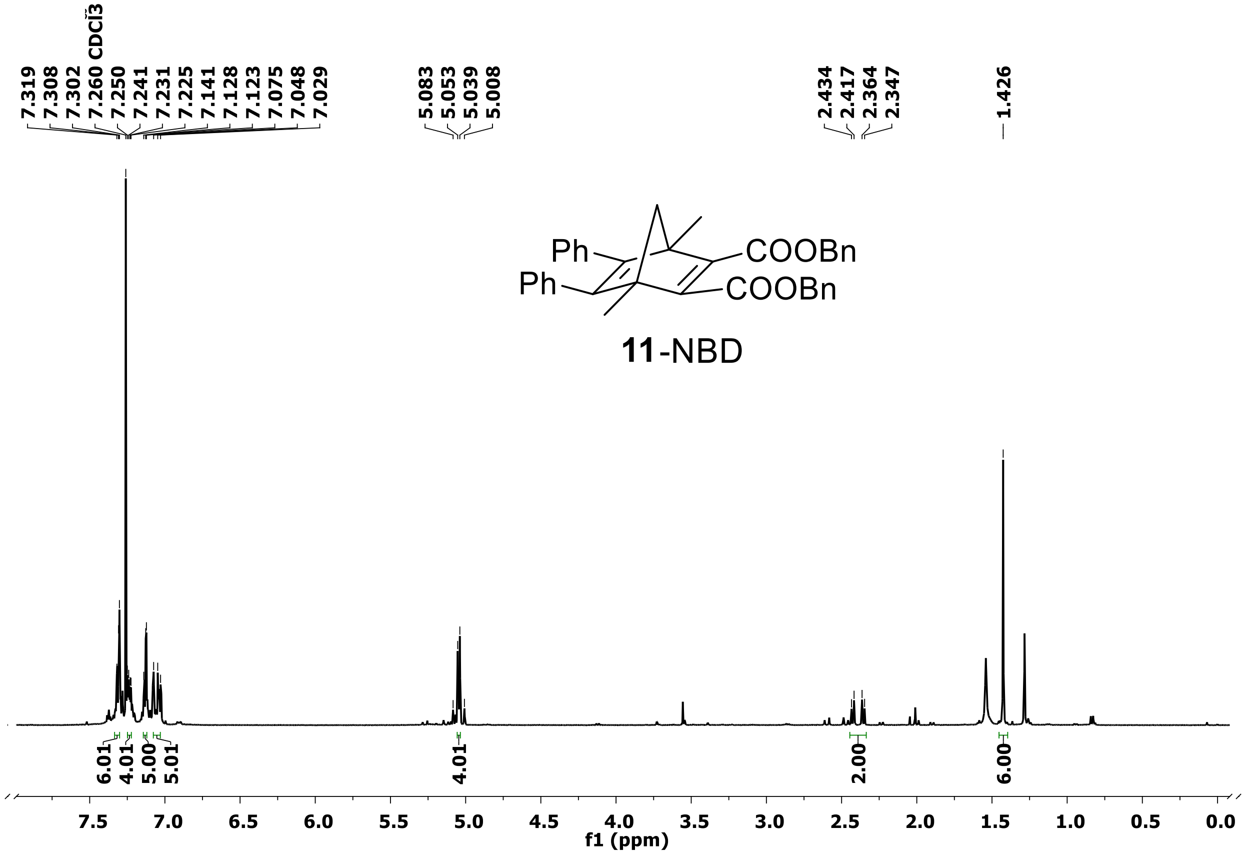
7.319
7.308
7.302
7.260 CDCl3
7.250
7.241
7.231
7.225
7.141
7.128
7.123
7.075
7.048
7.029
5.083
5.053
5.039
5.008
2.434
2.417
2.364
2.347
1.426
Ph
Ph
COOBn
COOBn
11-NBD
6.01
4.01
5.00
5.01
4.01
2.00
6.00
7.5 7.0 6.5 6.0 5.5 5.0 4.5 4.0 3.5 3.0 2.5 2.0 1.5 1.0 0.5 0.0
f1 (ppm)

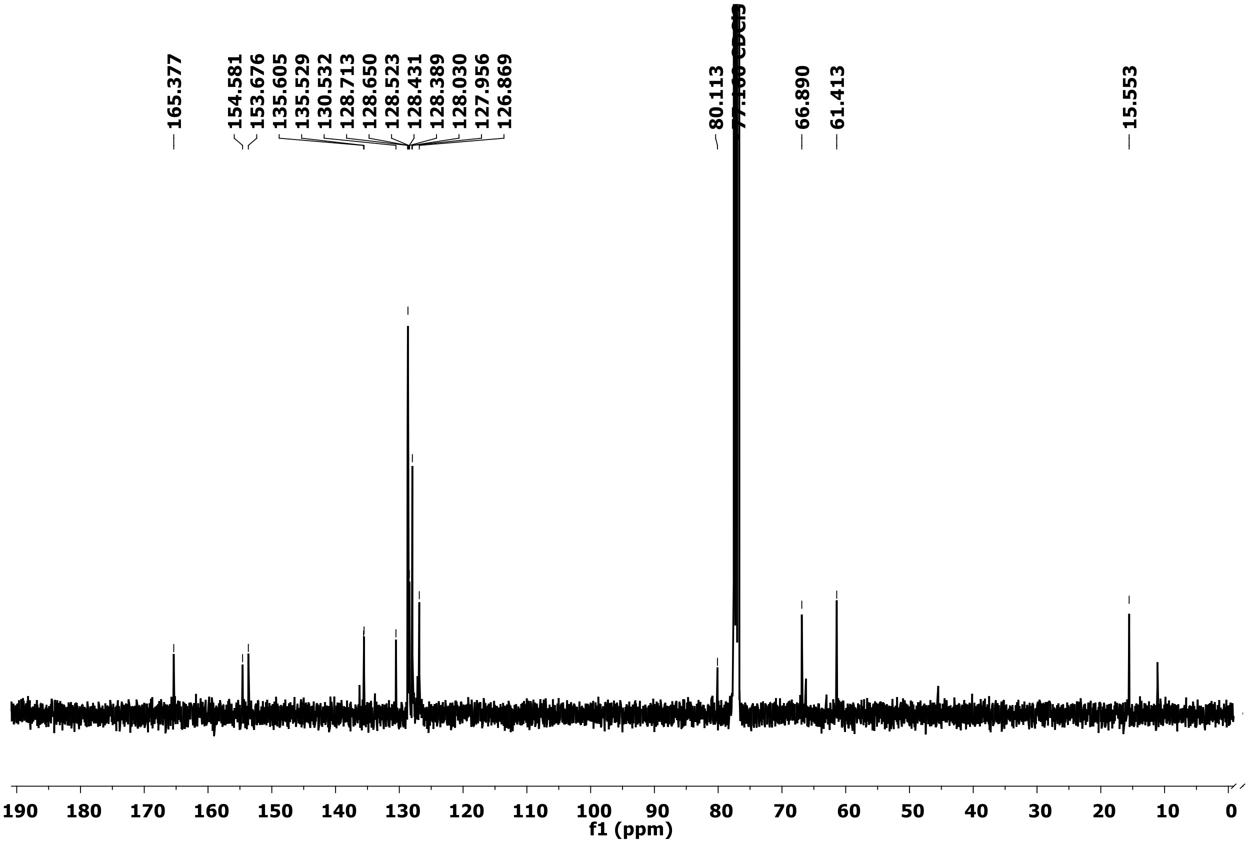
165.377
154.581
153.676
135.605
135.529
130.532
128.713
128.650
128.523
128.431
128.389
128.030
127.956
126.869
80.113
77.160 CDCl3
66.890
61.413
15.553
190 180 170 160 150 140 130 120 110 100 90 80 70 60 50 40 30 20 10 0
f1 (ppm)

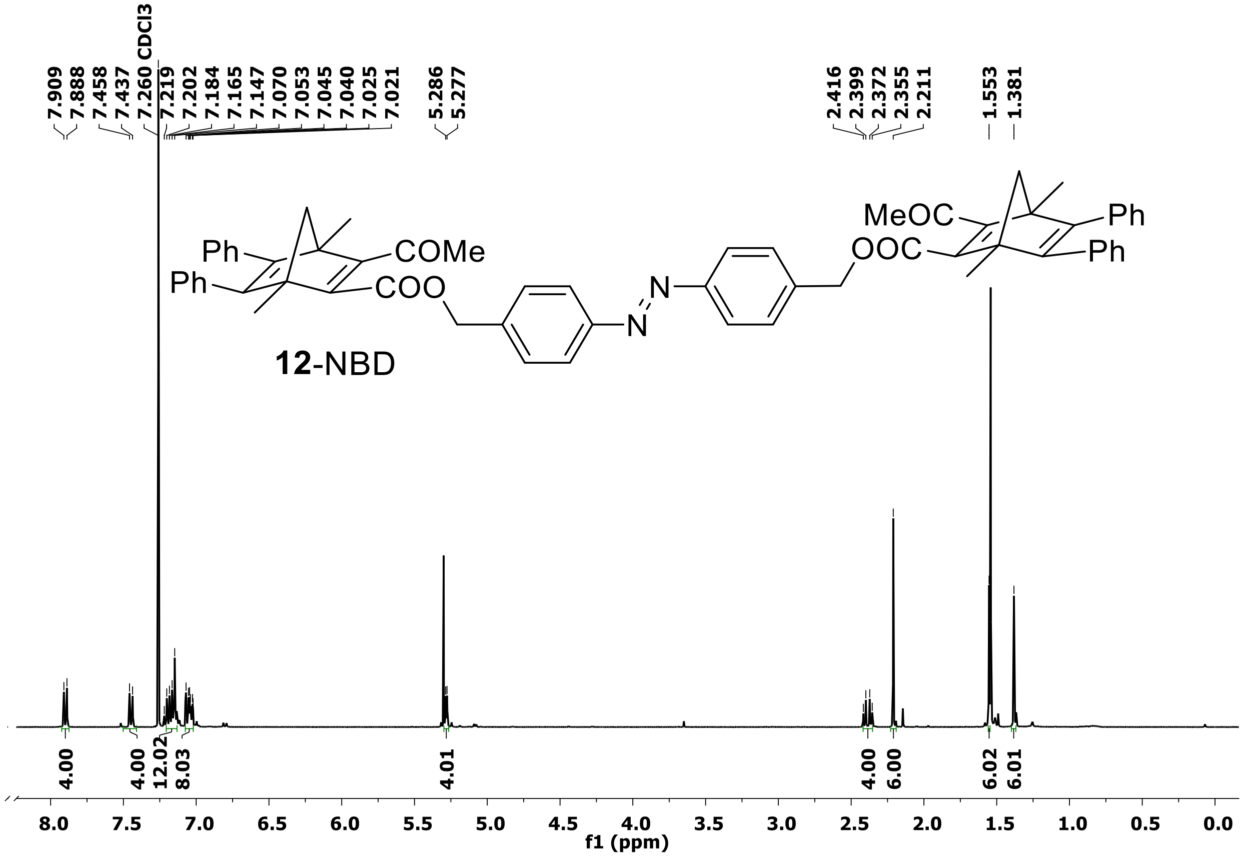
7.909
7.888
7.458
7.437
7.260 CDCl3
7.219
7.202
7.184
7.165
7.147
7.070
7.053
7.045
7.040
7.025
7.021
5.286
5.277
2.416
2.399
2.372
2.355
2.211
1.553
1.381
Ph
Ph
COMe
COO
N
N
MeOC
OOC
Ph
Ph
12-NBD
4.00
4.00
12.02
8.03
4.01
4.00
6.00
6.02
6.01
8.0 7.5 7.0 6.5 6.0 5.5 5.0 4.5 4.0 3.5 3.0 2.5 2.0 1.5 1.0 0.5 0.0
f1 (ppm)

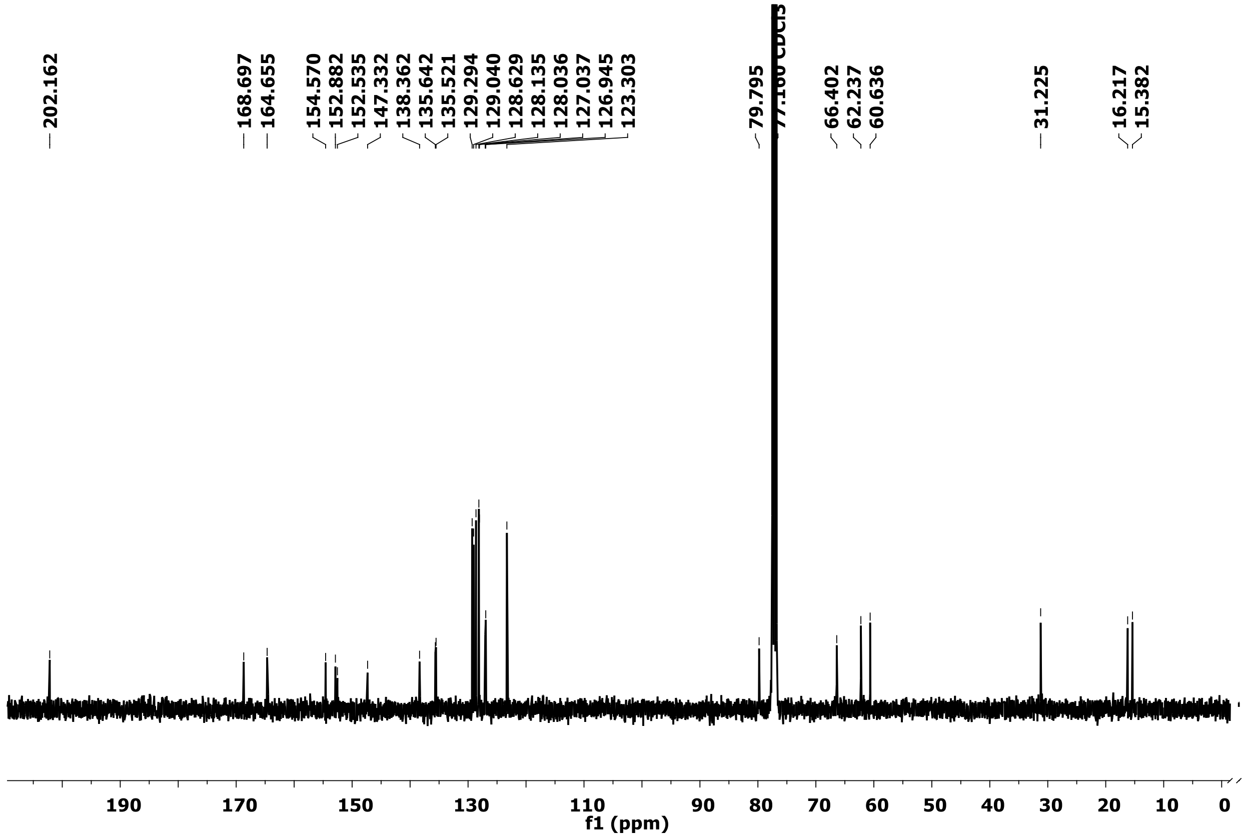
202.162
168.697
164.655
154.570
152.882
152.535
147.332
138.362
135.642
135.521
129.294
129.040
128.629
128.135
128.036
127.037
126.945
123.303
79.795
66.402
62.237
60.636
31.225
16.217
15.382
190 170 150 130 110 90 80 70 60 50 40 30 20 10 0
f1 (ppm)

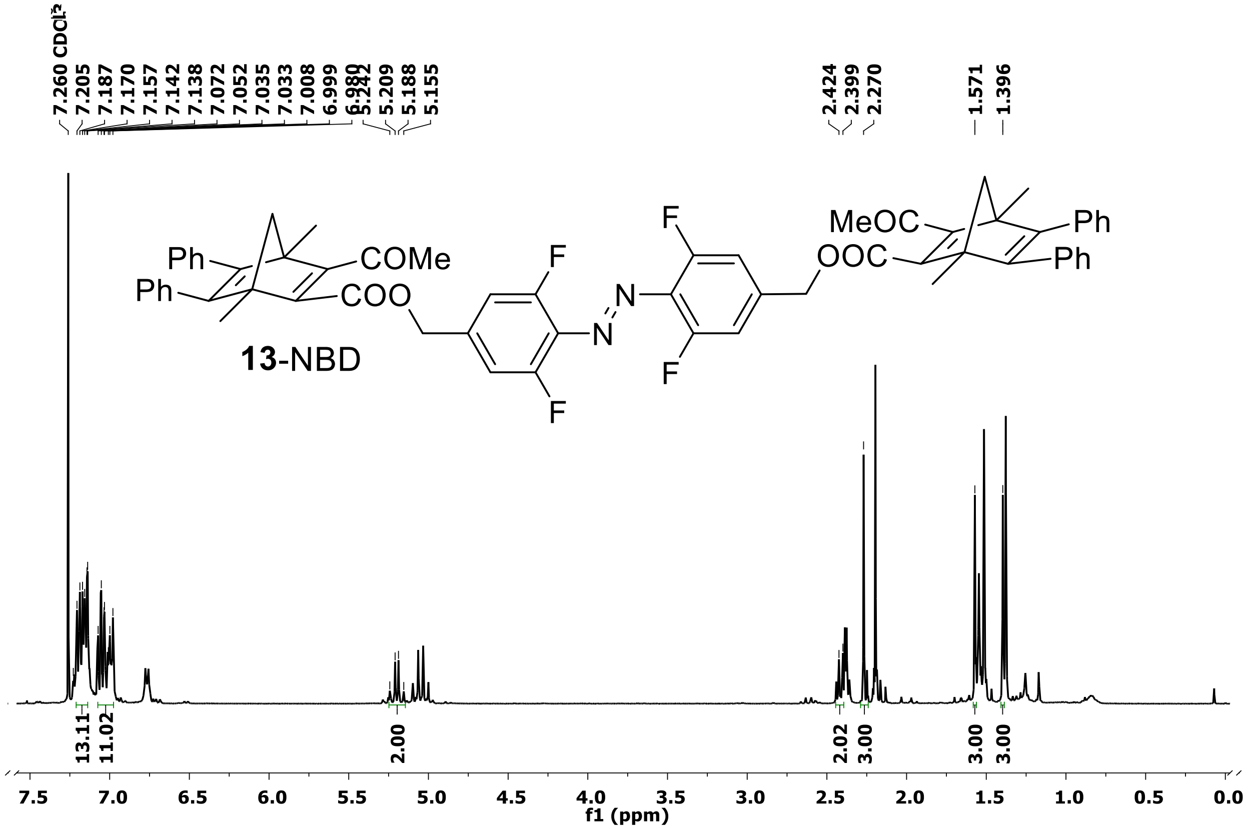
7.260 CDCl3
7.205
7.187
7.170
7.157
7.142
7.138
7.072
7.052
7.035
7.033
7.008
6.999
5.209
5.188
5.155
2.424
2.399
2.270
1.571
1.396
MeOC
OOC
Ph
Ph
COMe
COO
F
N
N
F
F
F
13-NBD
13.11
11.02
2.00
2.02
3.00
3.00
3.00
7.5 7.0 6.5 6.0 5.5 5.0 4.5 4.0 3.5 3.0 2.5 2.0 1.5 1.0 0.5 0.0
f1 (ppm)

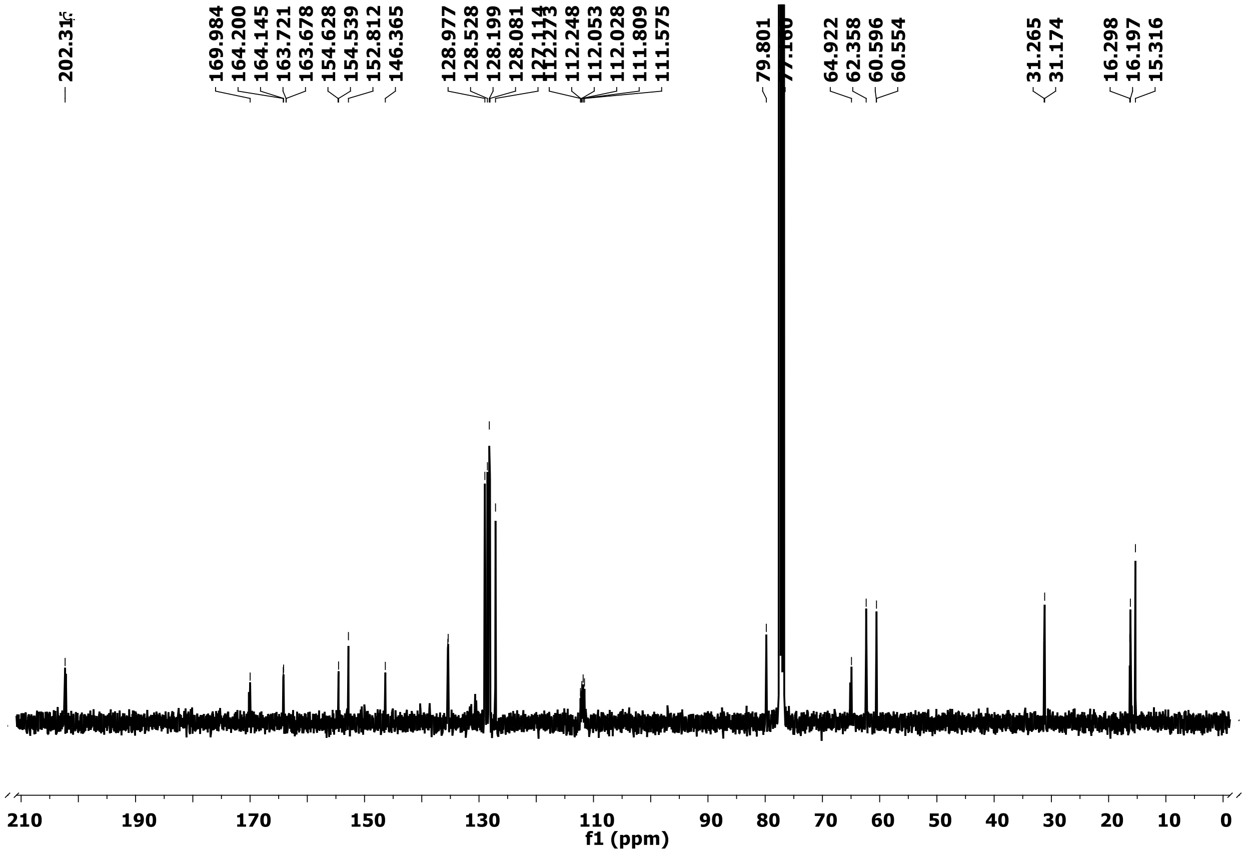
169.984
164.200
164.145
163.721
163.678
154.628
154.539
152.812
146.365
128.977
128.528
128.199
128.081
112.248
112.053
112.028
111.809
111.575
79.801
64.922
62.358
60.596
60.554
31.265
31.174
16.298
16.197
15.316
210 190 170 150 130 110 90 80 70 60 50 40 30 20 10 0
f1 (ppm)

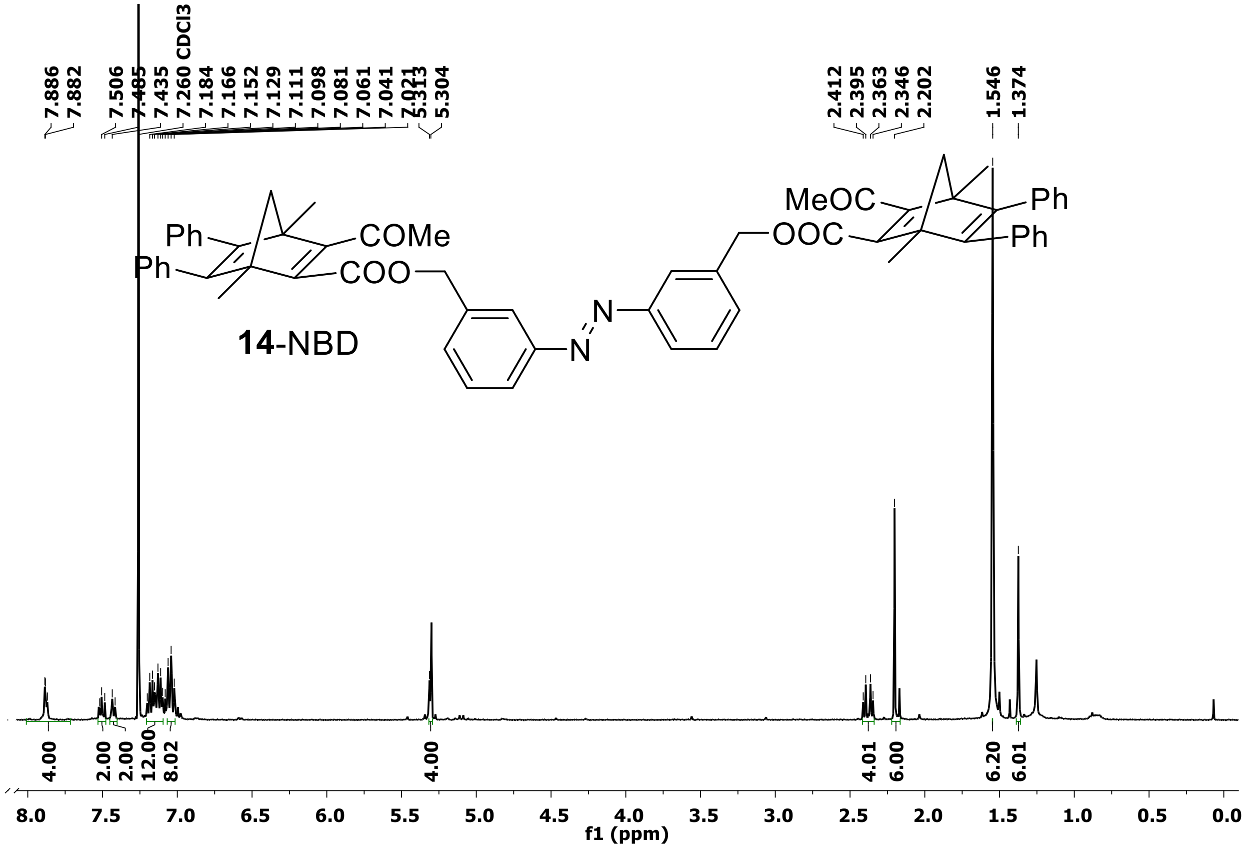
7.886
7.882
7.506
7.485
7.435
7.260 CDCl3
7.184
7.166
7.152
7.129
7.111
7.098
7.081
7.061
7.041
7.021
5.313
5.304
2.412
2.395
2.363
2.346
2.202
1.546
1.374
Ph
Ph
COMe
COO
14-NBD
N
N
MeOC
OOC
Ph
Ph
4.00
2.00
2.00
12.00
8.02
4.00
4.01
6.00
6.20
6.01
8.0 7.5 7.0 6.5 6.0 5.5 5.0 4.5 4.0 3.5 3.0 2.5 2.0 1.5 1.0 0.5 0.0
f1 (ppm)

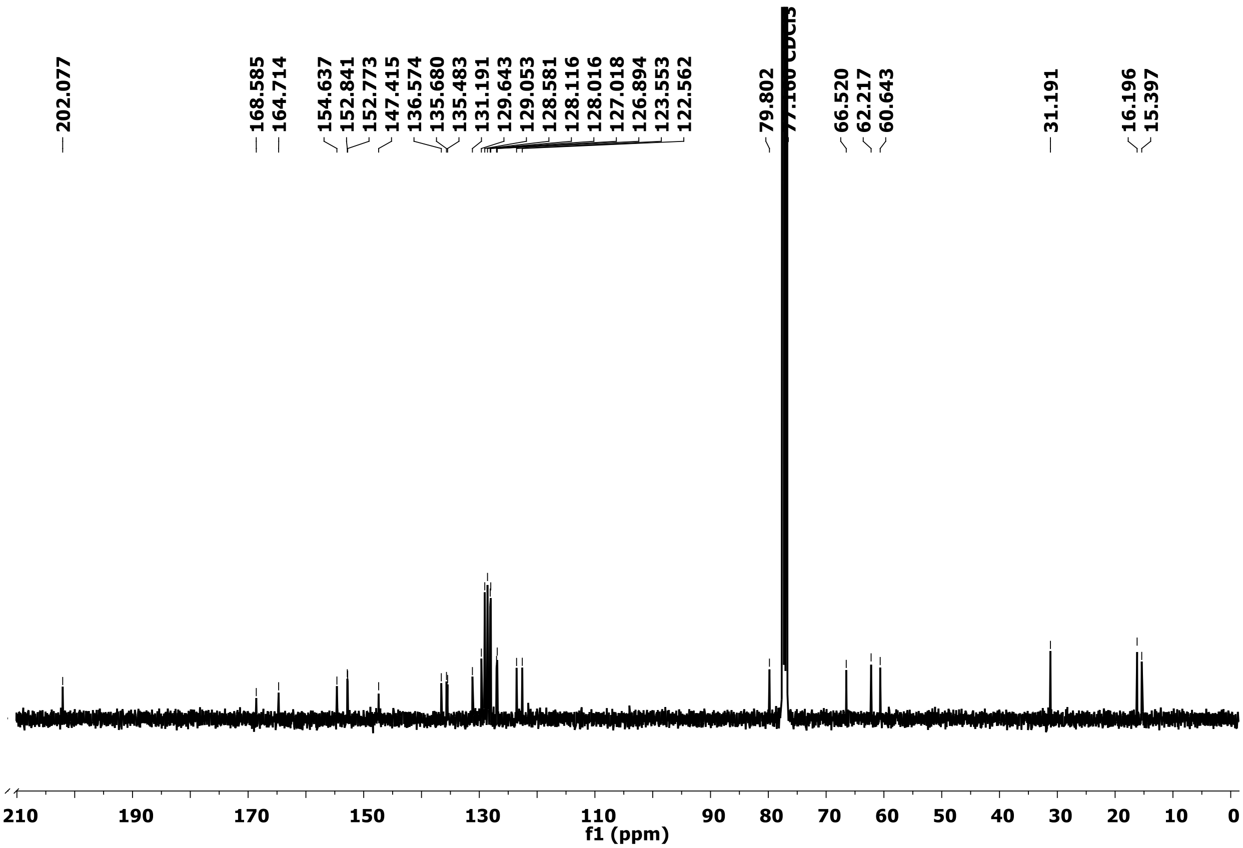
202.077
168.585
164.714
154.637
152.841
152.773
147.415
136.574
135.680
135.483
131.191
129.643
129.053
128.581
128.116
128.016
127.018
126.894
123.553
122.562
79.802
77.160 CDCl3
66.520
62.217
60.643
31.191
16.196
15.397
210 190 170 150 130 110 90 80 70 60 50 40 30 20 10 0
f1 (ppm)

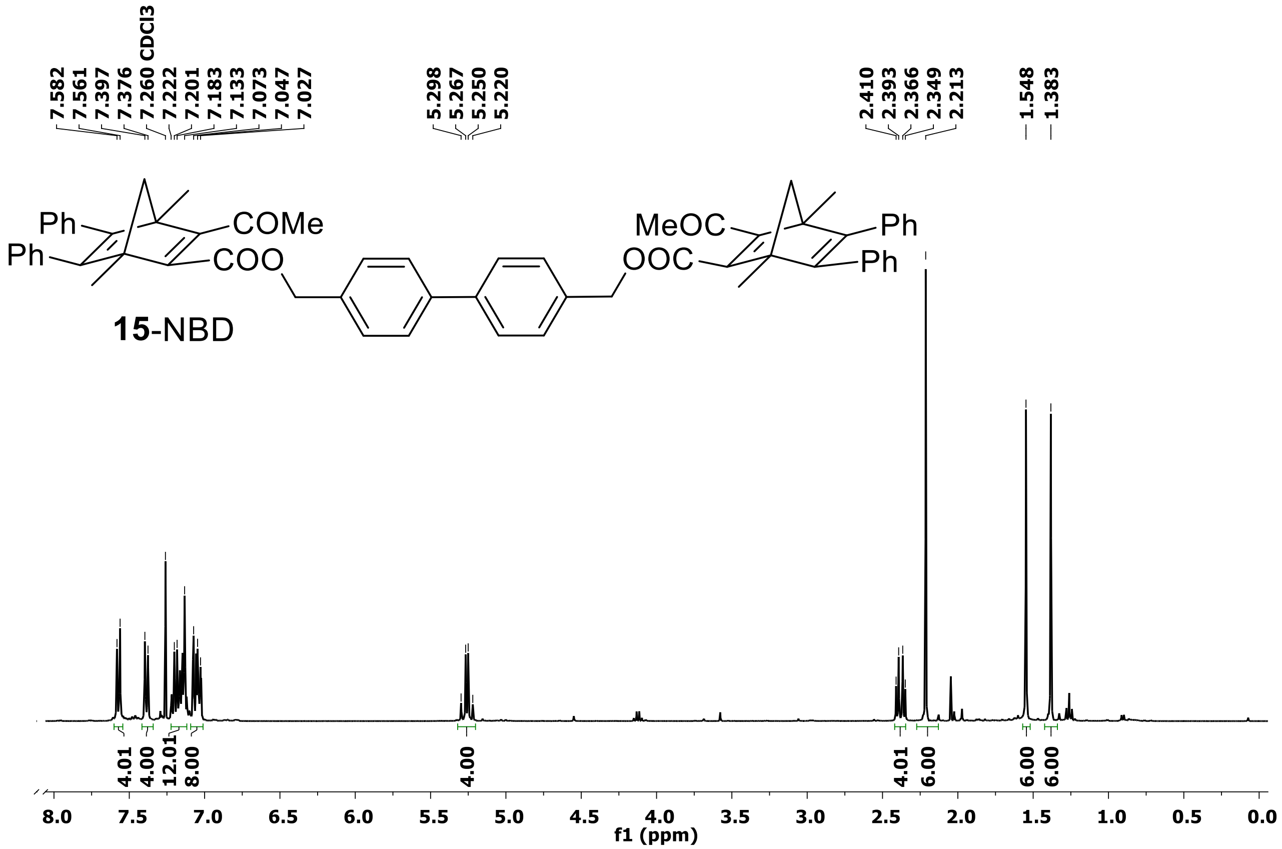
7.582
7.561
7.397
7.376
7.260 CDCl3
7.222
7.201
7.183
7.133
7.073
7.047
7.027
5.298
5.267
5.250
5.220
2.410
2.393
2.366
2.349
2.213
1.548
1.383
Ph
Ph
COMe
COO
MeOC
OOC
Ph
Ph
15-NBD
4.01
4.00
12.01
8.00
4.00
4.01
6.00
6.00
6.00
8.0 7.5 7.0 6.5 6.0 5.5 5.0 4.5 4.0 3.5 3.0 2.5 2.0 1.5 1.0 0.5 0.0
f1 (ppm)

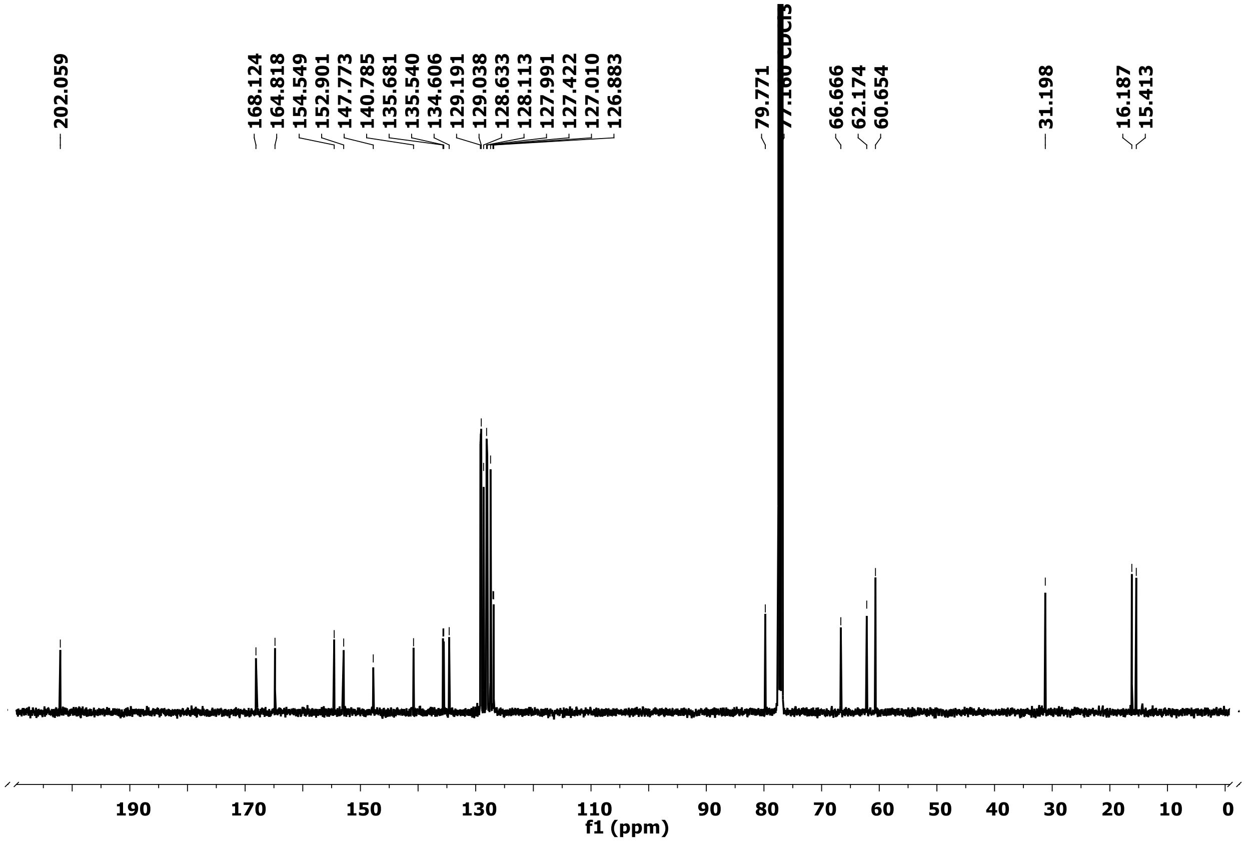
202.059
168.124
164.818
154.549
152.901
147.773
140.785
135.681
135.540
134.606
129.191
129.038
128.633
128.113
127.991
127.422
127.010
126.883
79.771
77.160 CDCl3
66.666
62.174
60.654
31.198
16.187
15.413
190 170 150 130 110 90 80 70 60 50 40 30 20 10 0
f1 (ppm)

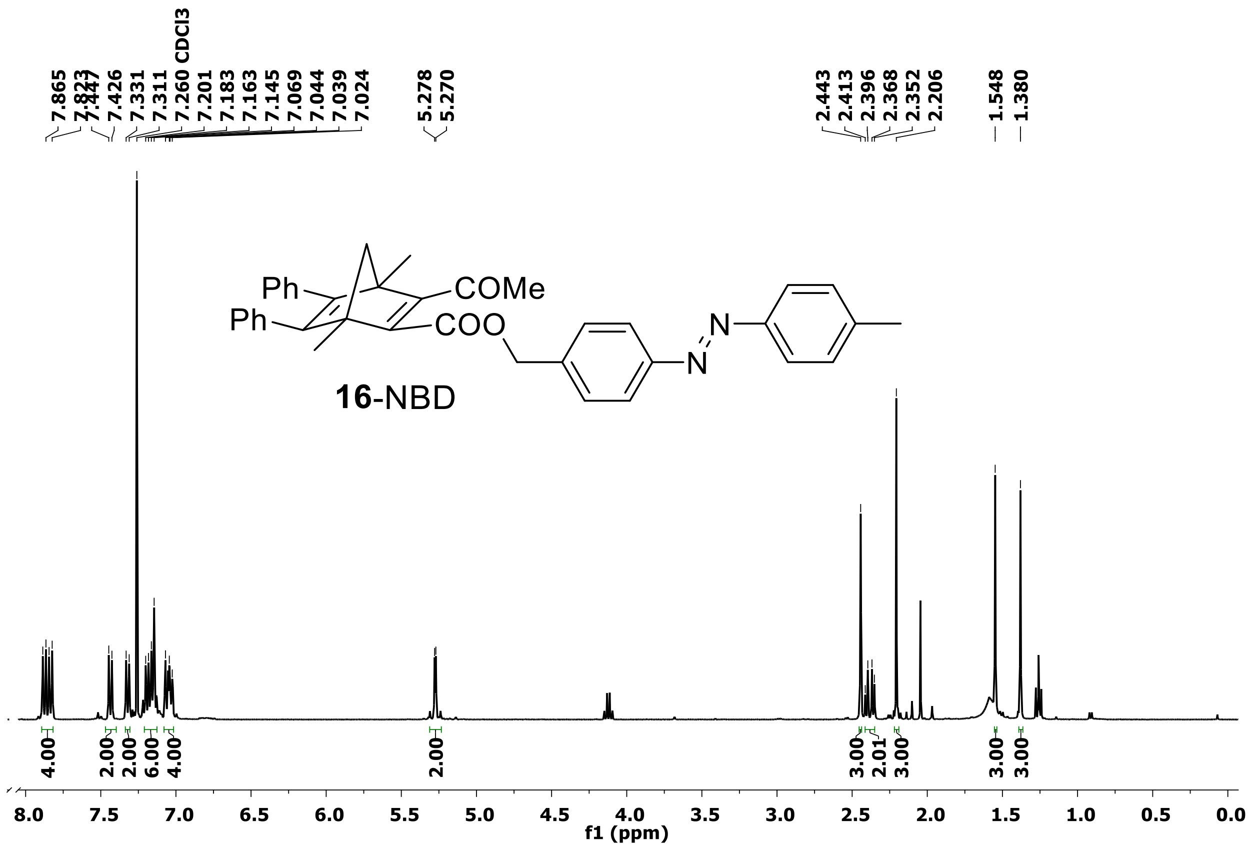
7.865
7.823
7.447
7.426
7.331
7.311
7.260 CDCl3
7.201
7.183
7.163
7.145
7.069
7.044
7.039
7.024
5.278
5.270
2.443
2.413
2.396
2.368
2.352
2.206
1.548
1.380
Ph
Ph
COMe
COO
N
N
16-NBD
4.00
2.00
2.00
6.00
4.00
2.00
3.00
2.01
3.00
3.00
3.00
8.0
7.5
7.0
6.5
6.0
5.5
5.0
4.5
4.0
3.5
3.0
2.5
2.0
1.5
1.0
0.5
0.0
f1 (ppm)

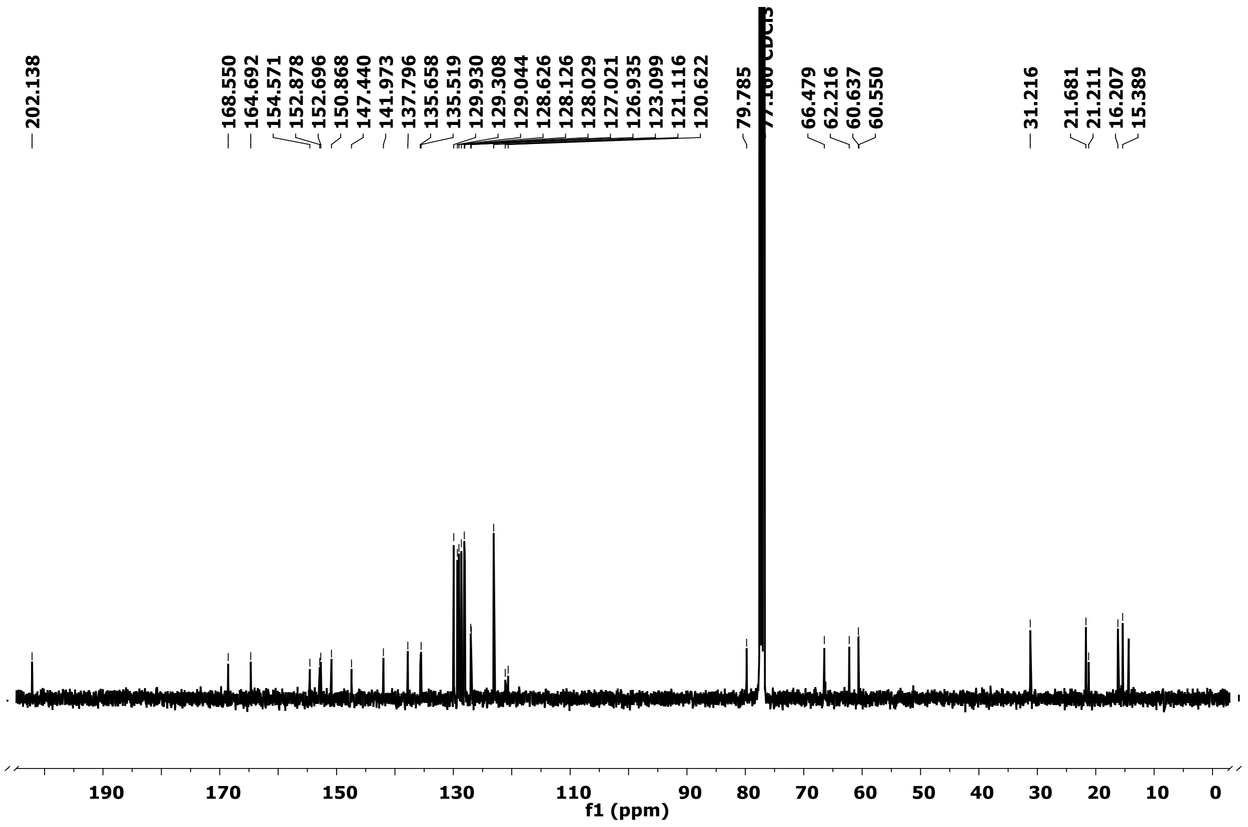
202.138
168.550
164.692
154.571
152.878
152.696
150.868
147.440
141.973
137.796
135.658
135.519
129.930
129.308
129.044
128.626
128.126
128.029
127.021
126.935
123.099
121.116
120.622
79.785
66.479
62.216
60.637
60.550
31.216
21.681
21.211
16.207
15.389
190
170
150
130
110
90
80
70
60
50
40
30
20
10
0
f1 (ppm)

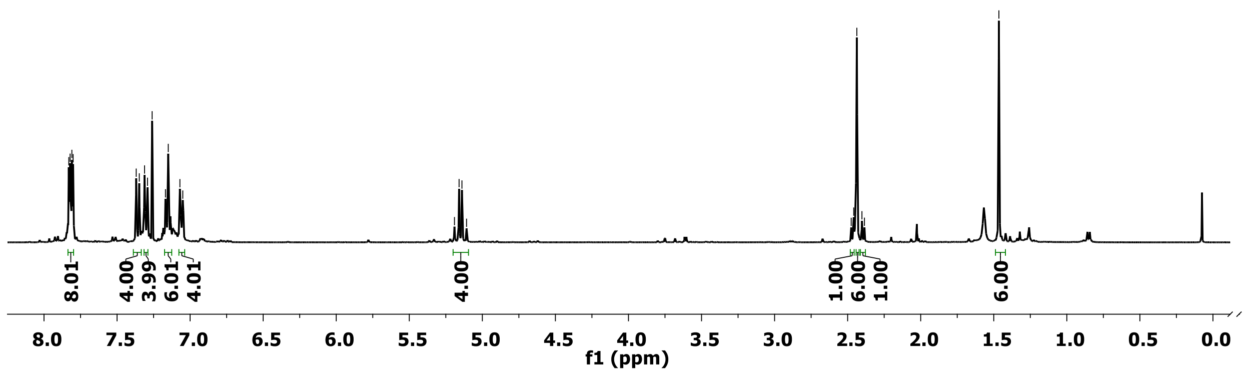
7.830
7.821
7.809
7.801
7.369
7.348
7.312
7.292
7.260 CDCl3
7.169
7.150
7.070
7.050
5.191
5.159
5.140
5.108
2.476
2.459
2.437
2.402
2.385
1.465
8.01
4.00
3.99
6.01
4.01
4.00
1.00
6.00
1.00
6.00
f1 (ppm)

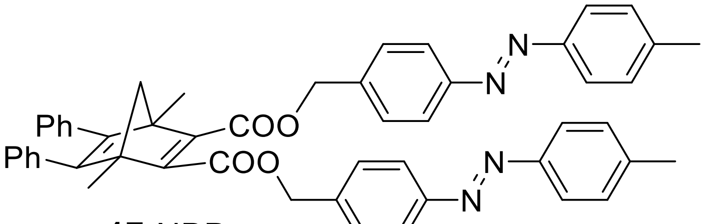
Ph
Ph
COO
COO
N
N
N
N

17-NBD

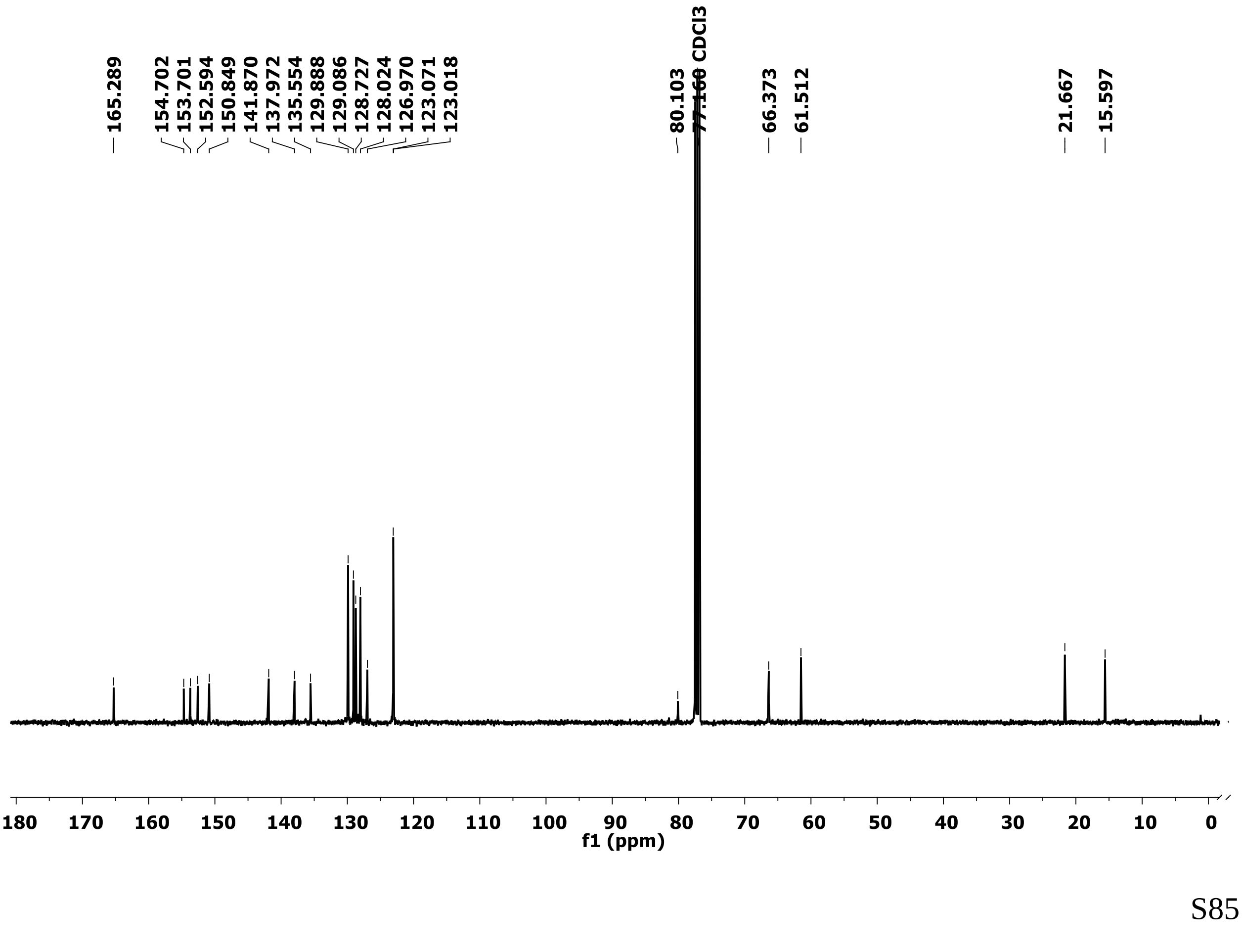
165.289
154.702
153.701
152.594
150.849
141.870
137.972
135.554
129.888
129.086
128.727
128.024
126.970
123.071
123.018
80.103
77.160 CDCl3
66.373
61.512
21.667
15.597
f1 (ppm)

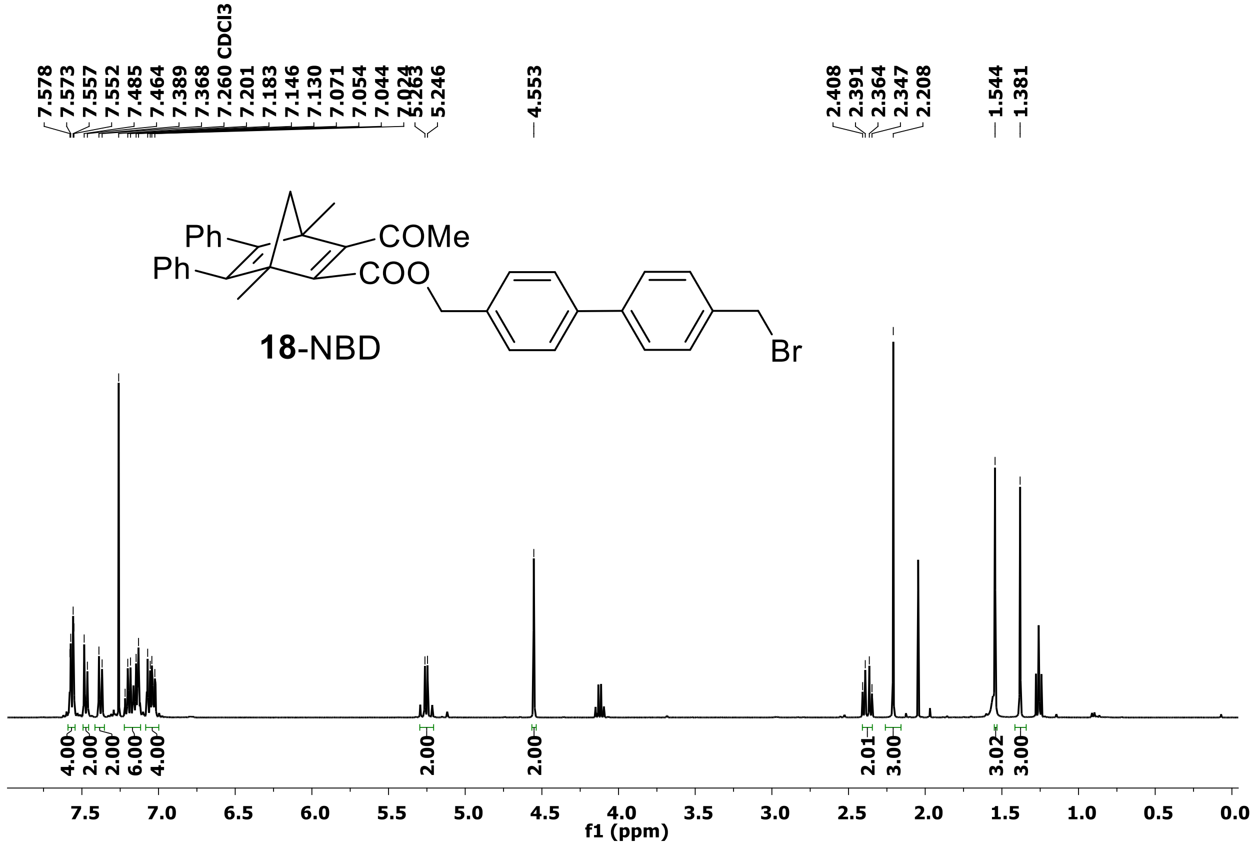
7.578
7.573
7.557
7.552
7.485
7.464
7.389
7.368
7.260 CDCl3
7.201
7.183
7.146
7.130
7.071
7.054
7.044
7.024
5.263
5.246
4.553
2.408
2.391
2.364
2.347
2.208
1.544
1.381
Ph
Ph
COMe
COO
Br
18-NBD
4.00
2.00
2.00
6.00
4.00
2.00
2.00
2.01
3.00
3.02
3.00
f1 (ppm)

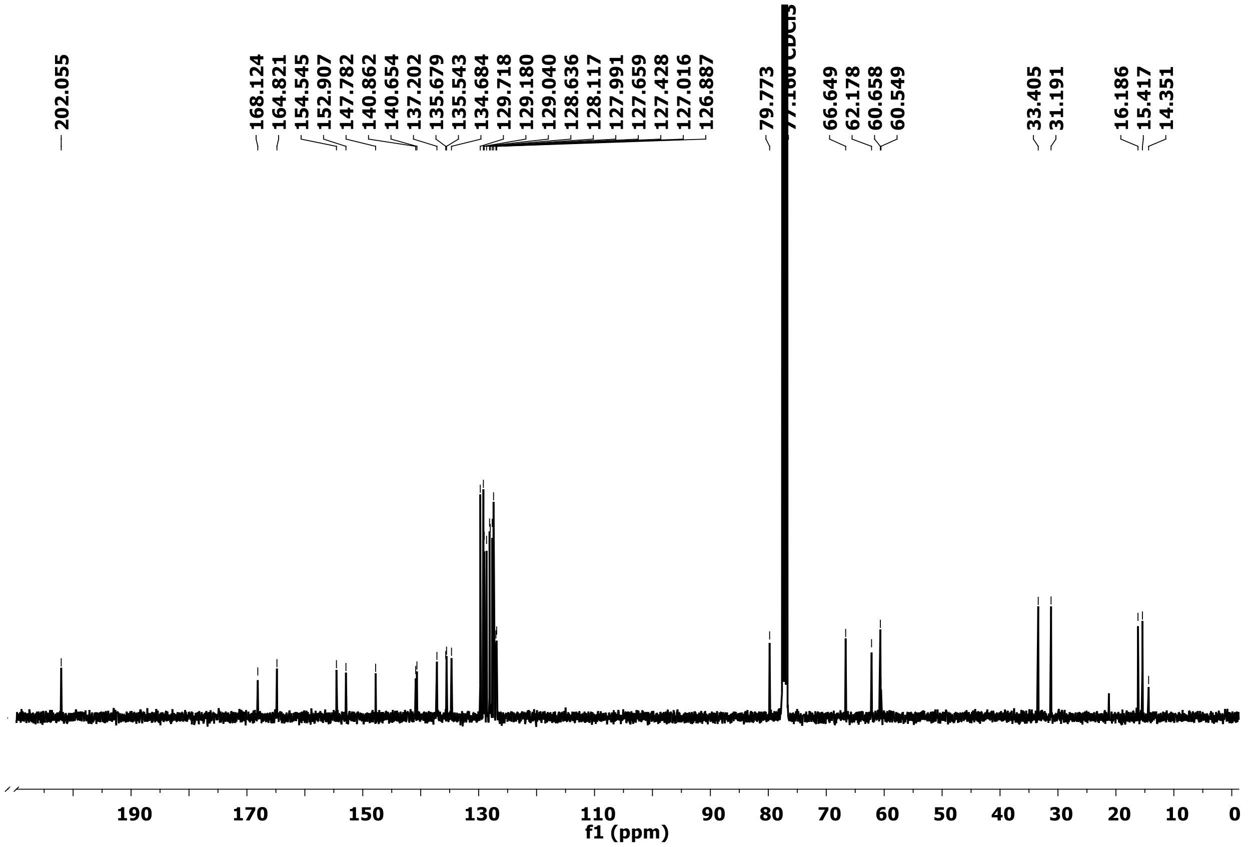
202.055
168.124
164.821
154.545
152.907
147.782
140.862
140.654
137.202
135.679
135.543
134.684
129.718
129.180
129.040
128.636
128.117
127.991
127.659
127.428
127.016
126.887
79.773
77.160 CDCl3
66.649
62.178
60.658
60.549
33.405
31.191
16.186
15.417
14.351
f1 (ppm)

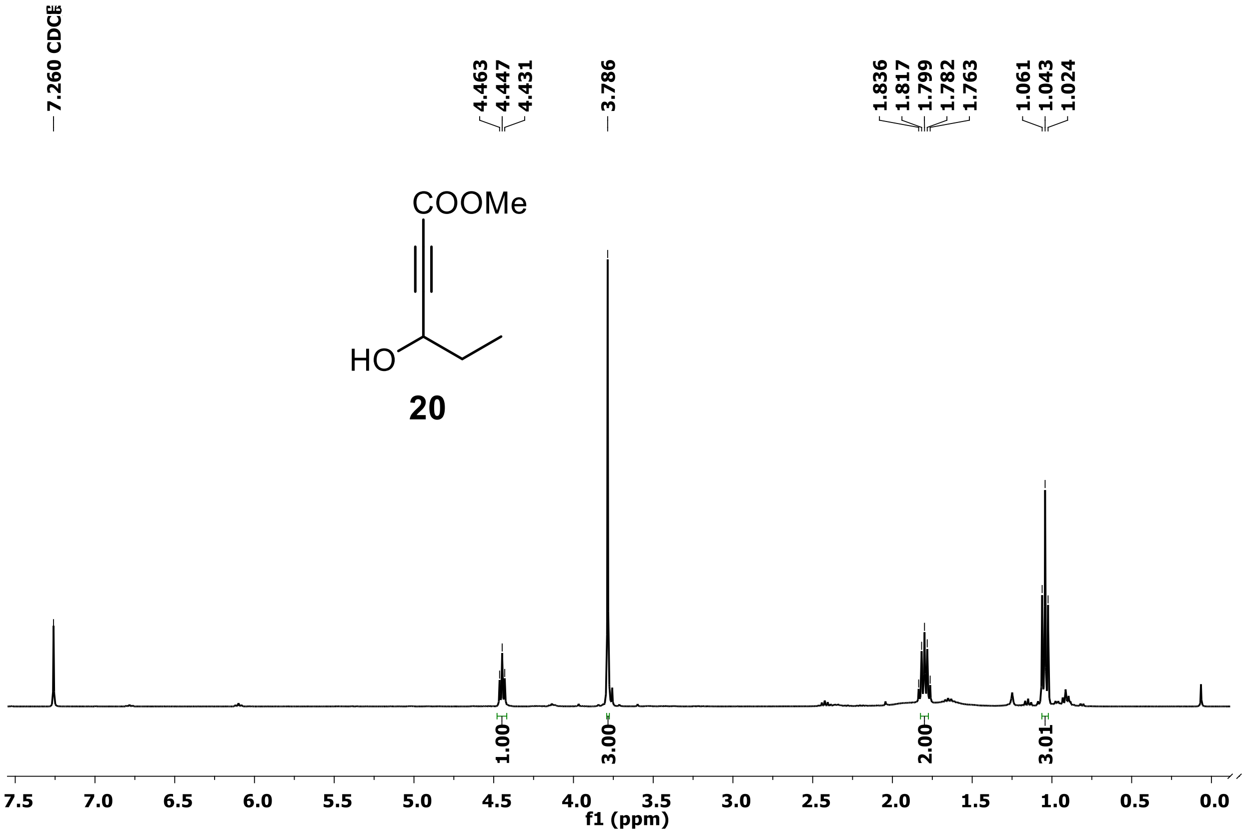
7.260 CDCl3
4.463
4.447
4.431
3.786
1.836
1.817
1.799
1.782
1.763
1.061
1.043
1.024
COOMe
HO
20
1.00
3.00
2.00
3.01
7.5 7.0 6.5 6.0 5.5 5.0 4.5 4.0 3.5 3.0 2.5 2.0 1.5 1.0 0.5 0.0
f1 (ppm)

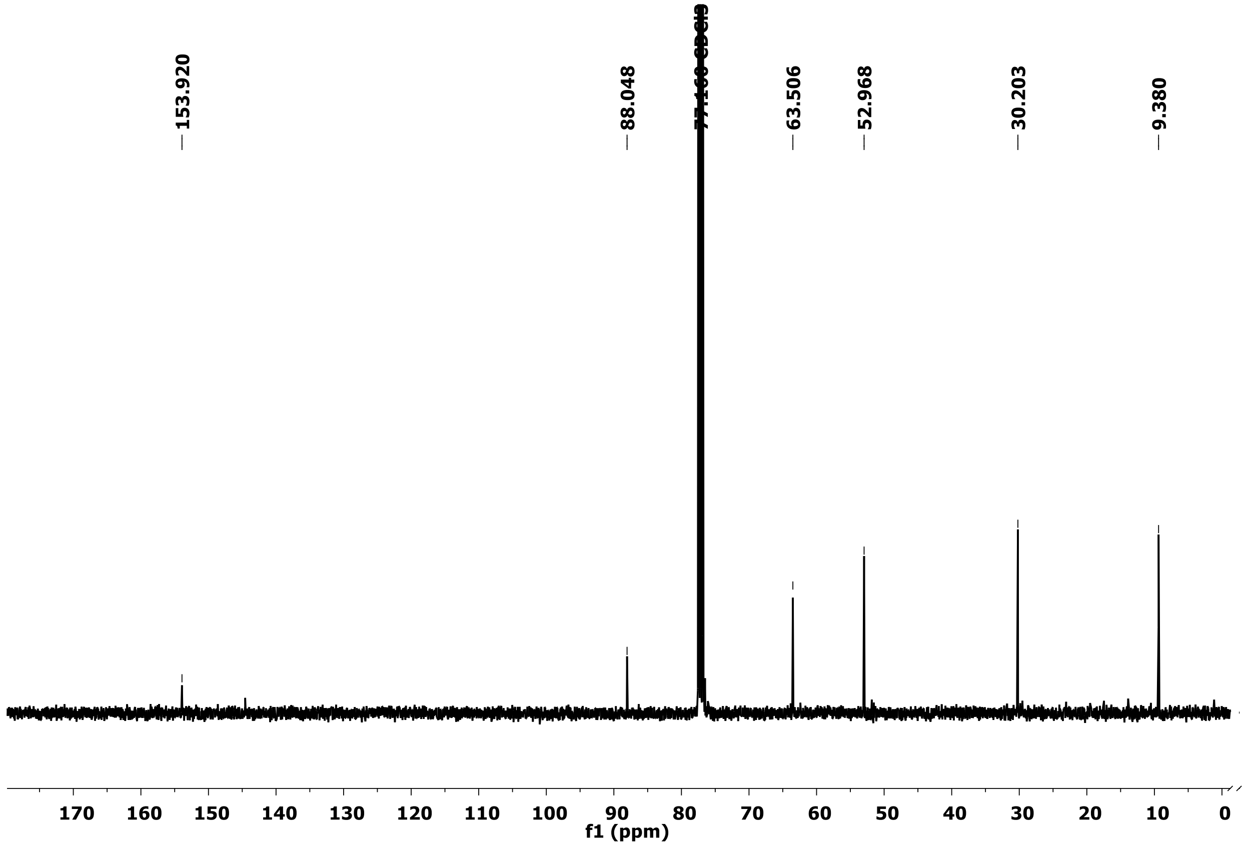
153.920
88.048
77.160 CDCl3
63.506
52.968
30.203
9.380
170 160 150 140 130 120 110 100 90 80 70 60 50 40 30 20 10 0
f1 (ppm)

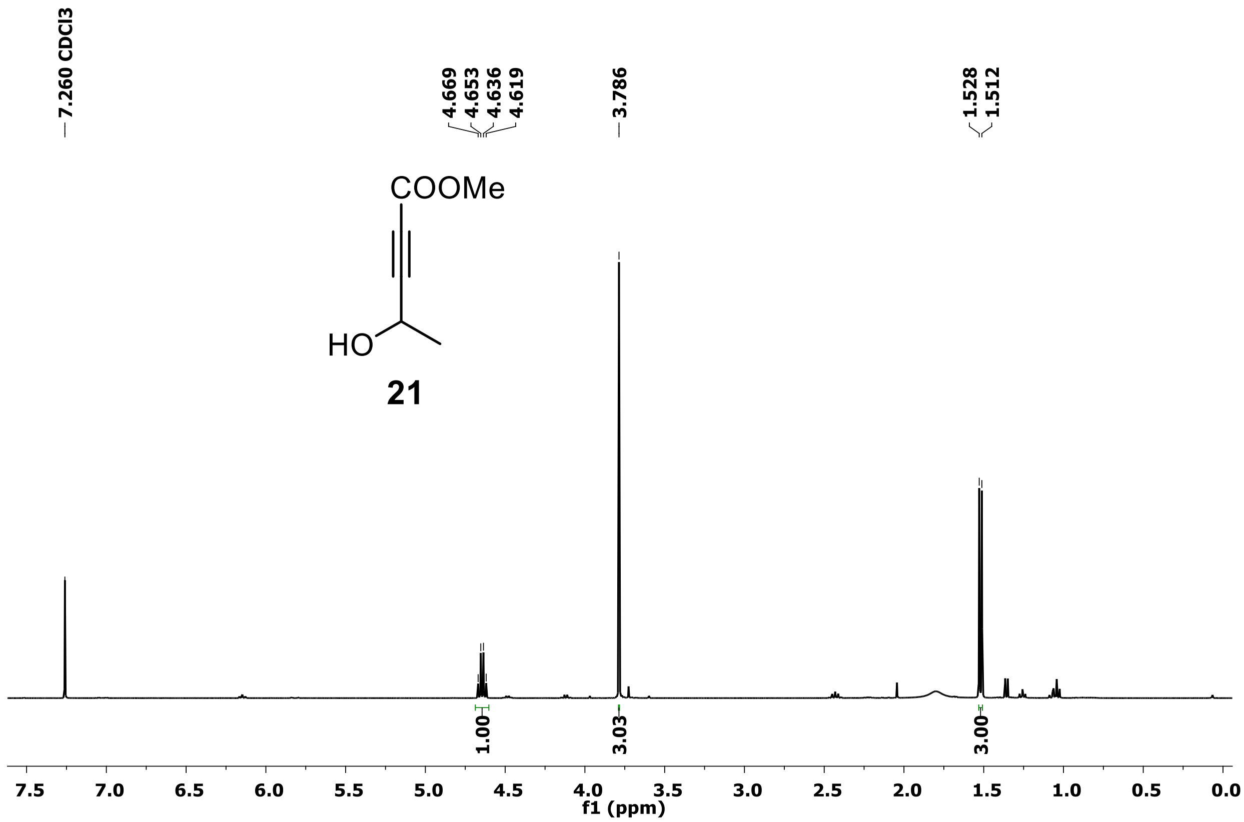
7.260 CDCl3
4.669
4.653
4.636
4.619
3.786
1.528
1.512
COOMe
HO
21
1.00
3.03
3.00
7.5 7.0 6.5 6.0 5.5 5.0 4.5 4.0 3.5 3.0 2.5 2.0 1.5 1.0 0.5 0.0
f1 (ppm)

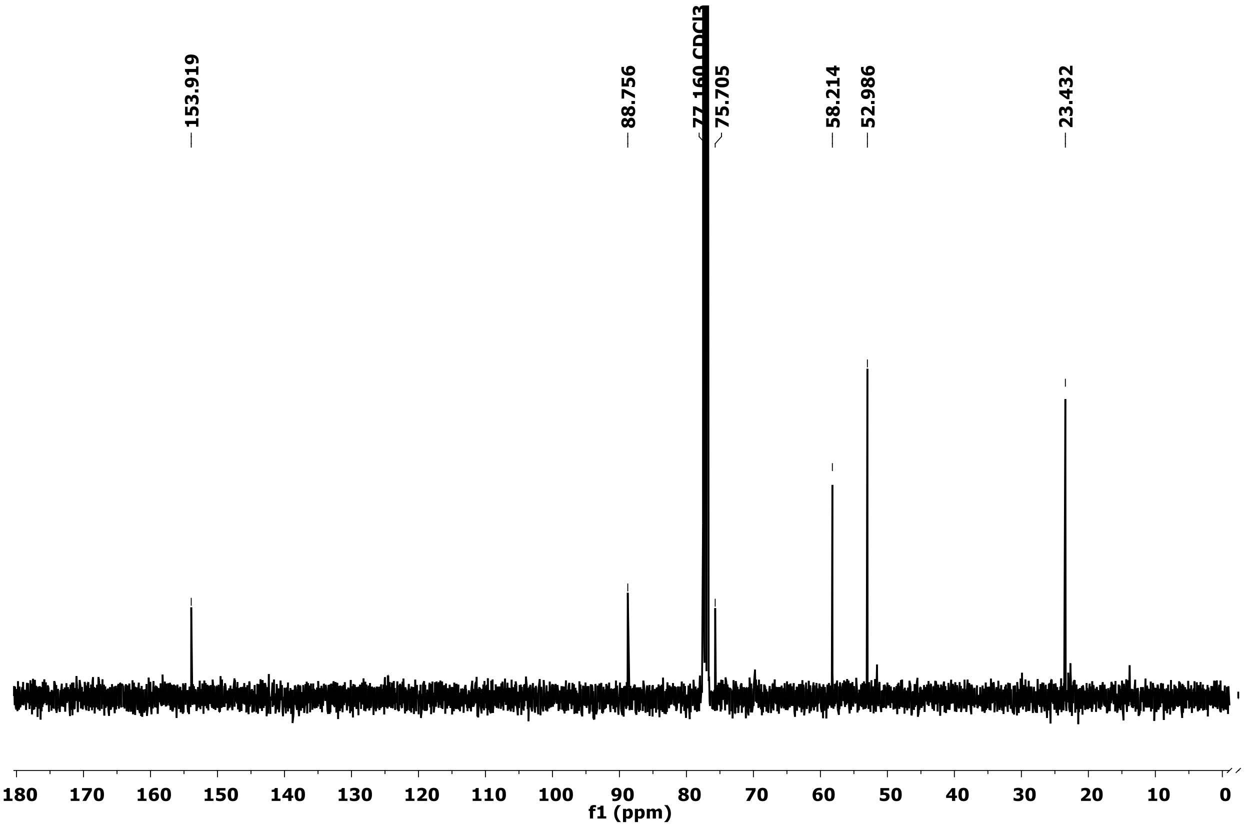
153.919
88.756
77.160 CDCl3
75.705
58.214
52.986
23.432
180 170 160 150 140 130 120 110 100 90 80 70 60 50 40 30 20 10 0
f1 (ppm)

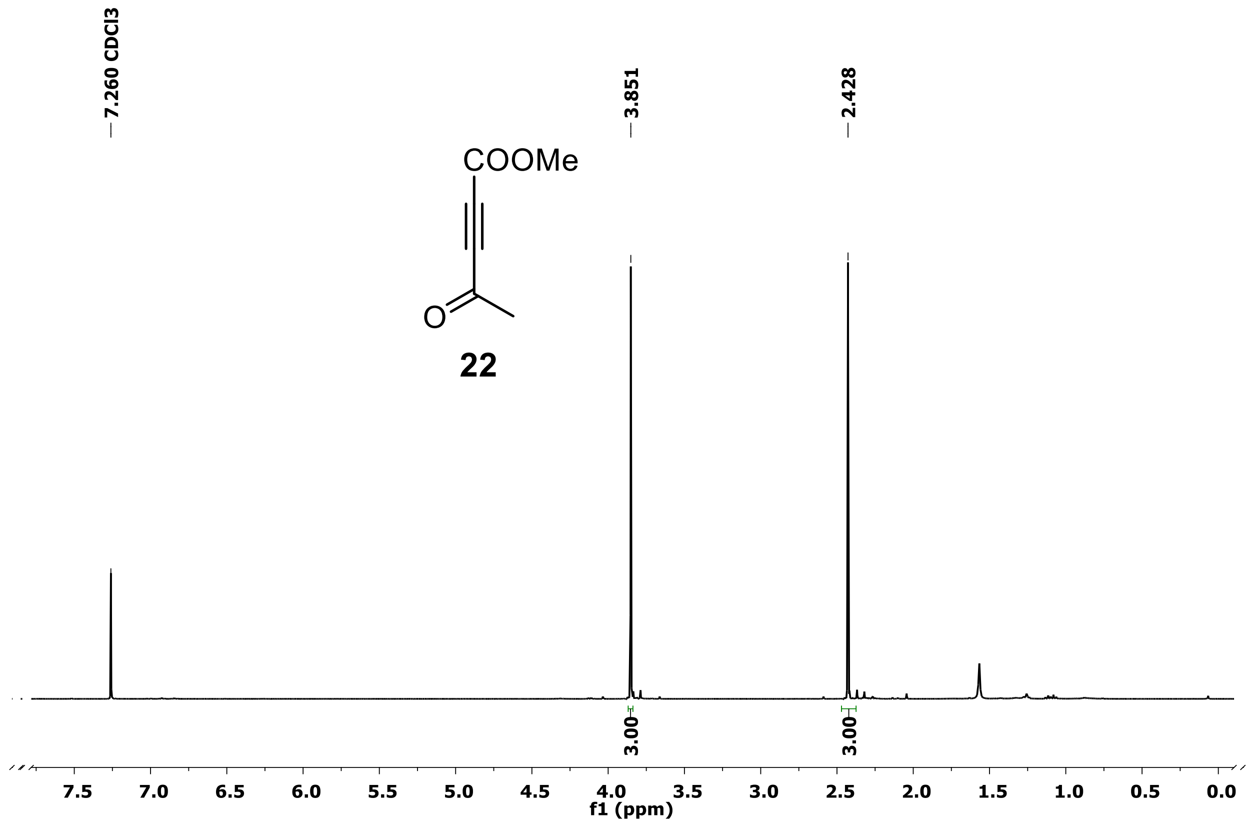
7.260 CDCl3
3.851
2.428
COOMe
O
22
3.00
3.00
7.5 7.0 6.5 6.0 5.5 5.0 4.5 4.0 3.5 3.0 2.5 2.0 1.5 1.0 0.5 0.0
f1 (ppm)

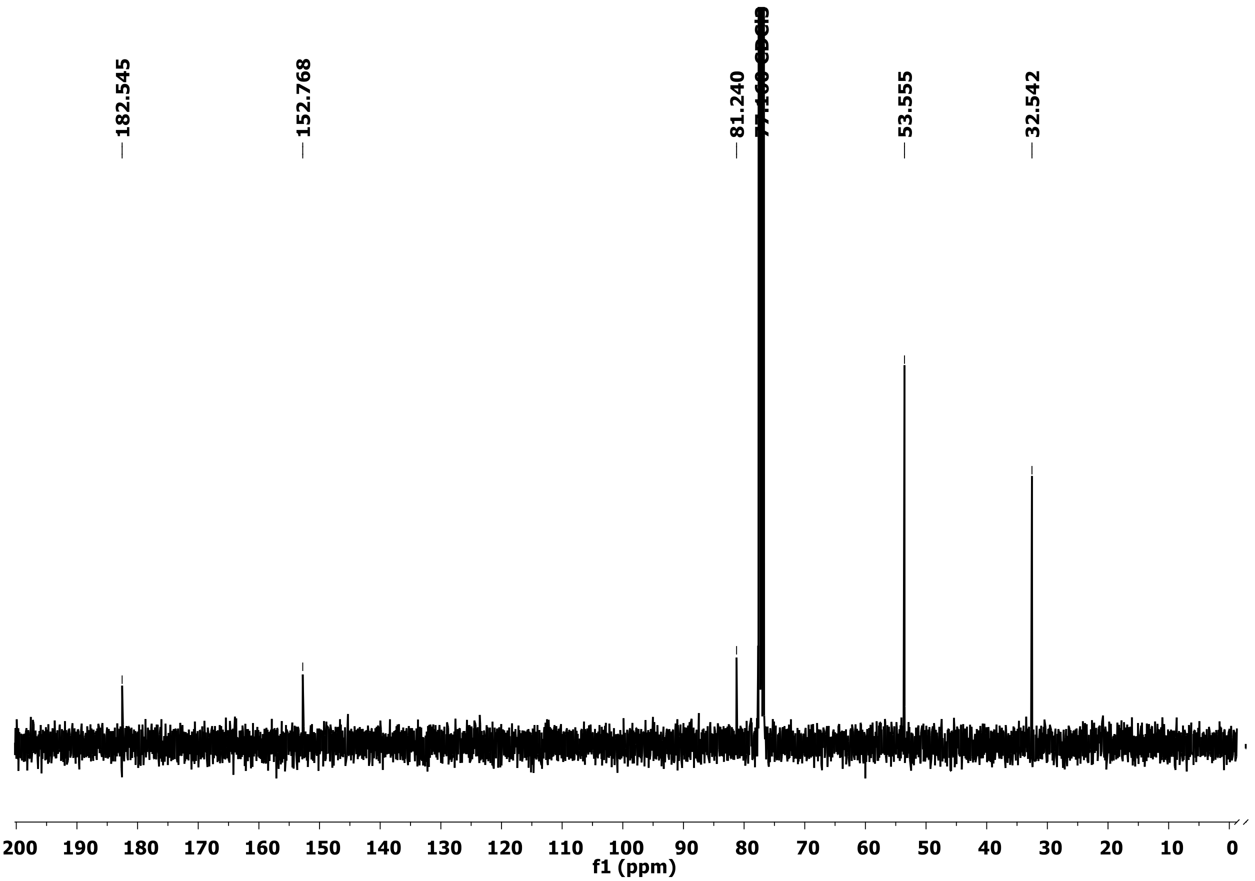
182.545
152.768
81.240
53.555
32.542
200 190 180 170 160 150 140 130 120 110 100 90 80 70 60 50 40 30 20 10 0
f1 (ppm)

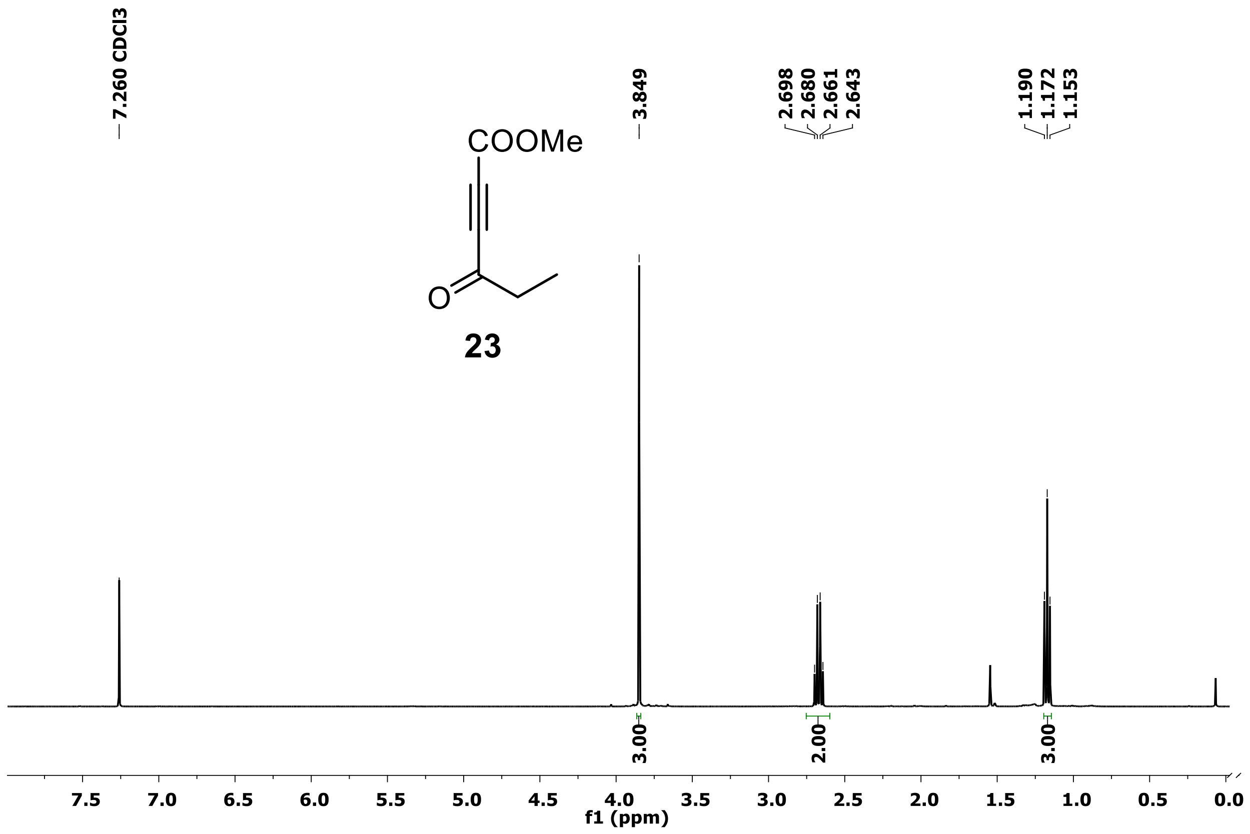
7.260 CDCl3
3.849
2.698
2.680
2.661
2.643
1.190
1.172
1.153
COOMe
O
23
3.00
2.00
3.00
7.5 7.0 6.5 6.0 5.5 5.0 4.5 4.0 3.5 3.0 2.5 2.0 1.5 1.0 0.5 0.0
f1 (ppm)

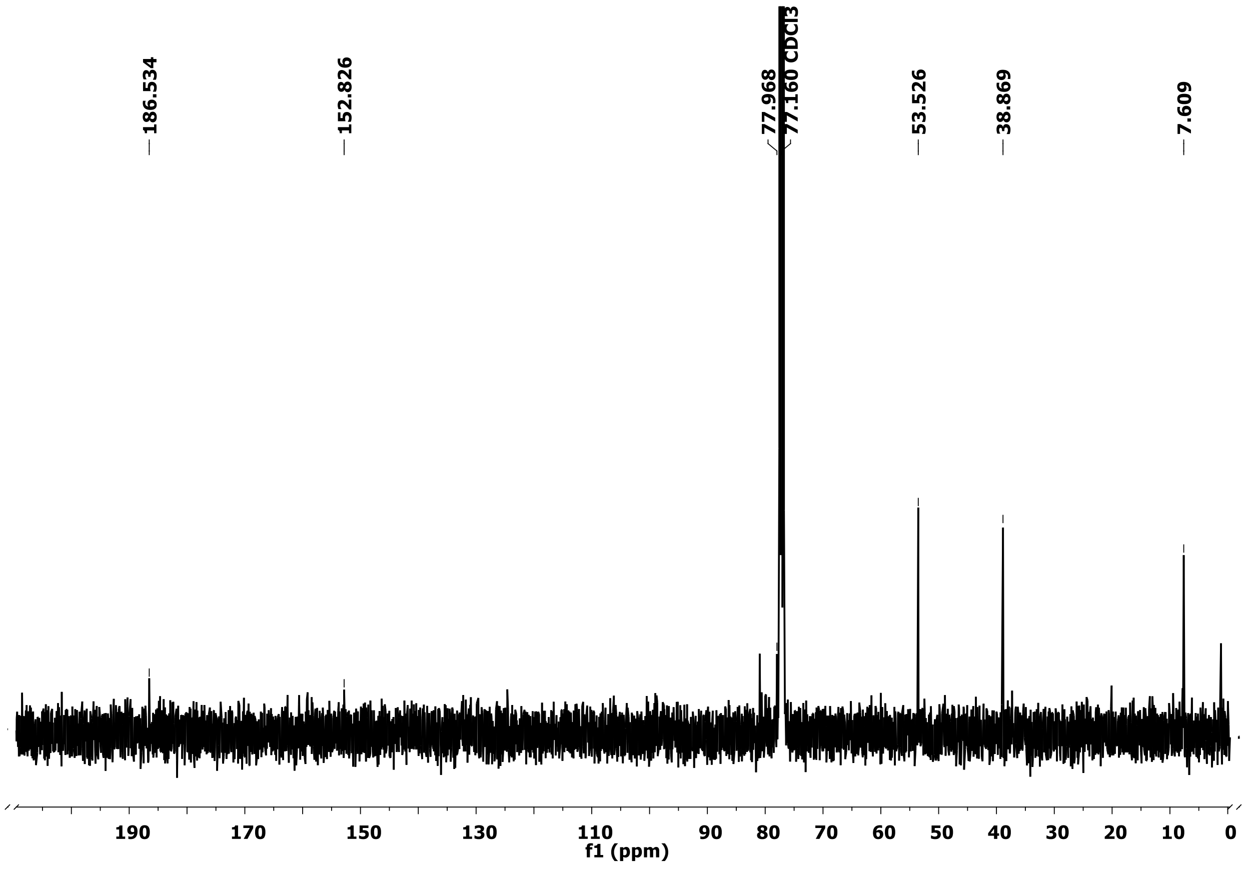
186.534
152.826
77.968
77.160 CDCl3
53.526
38.869
7.609
190 170 150 130 110 90 80 70 60 50 40 30 20 10 0
f1 (ppm)

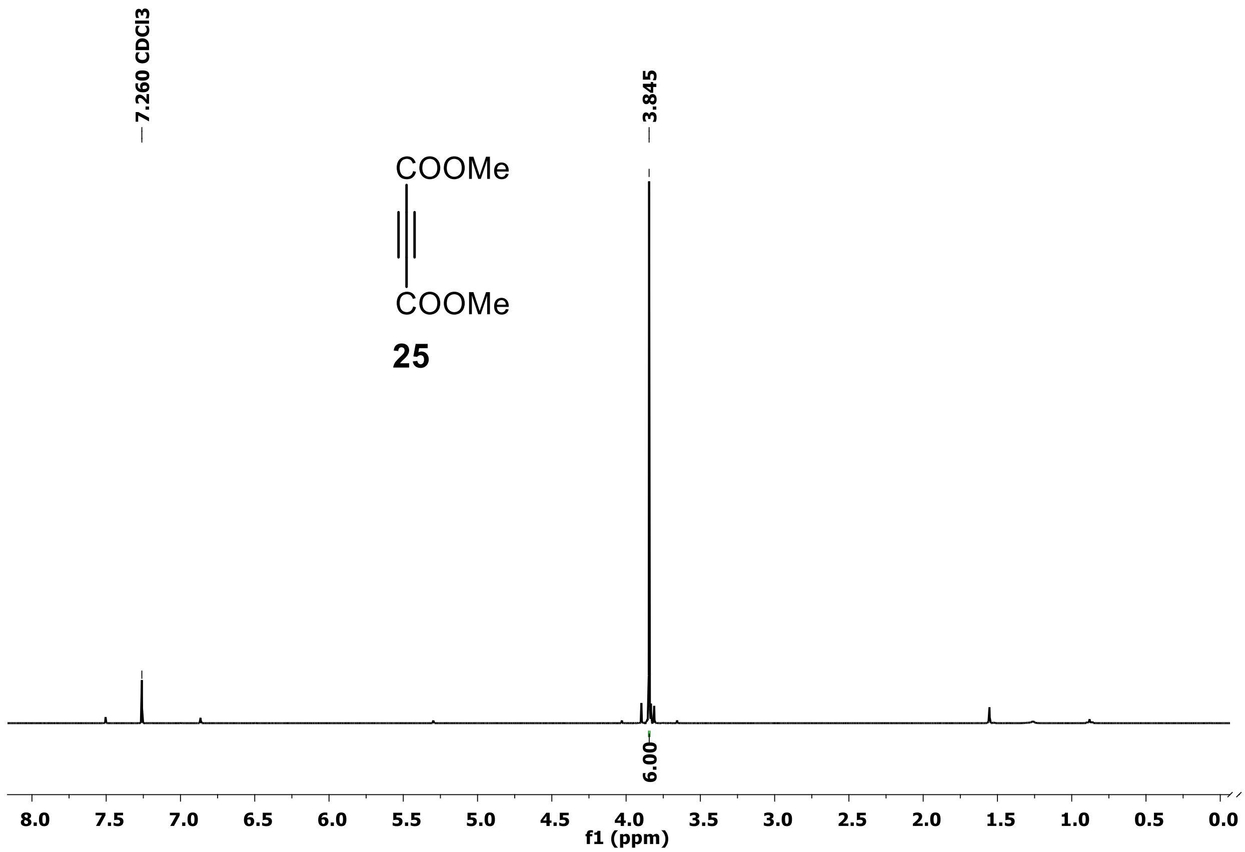

7.260 CDCl3
3.845
COOMe
COOMe
25
6.00
8.0 7.5 7.0 6.5 6.0 5.5 5.0 4.5 4.0 3.5 3.0 2.5 2.0 1.5 1.0 0.5 0.0
f1 (ppm)

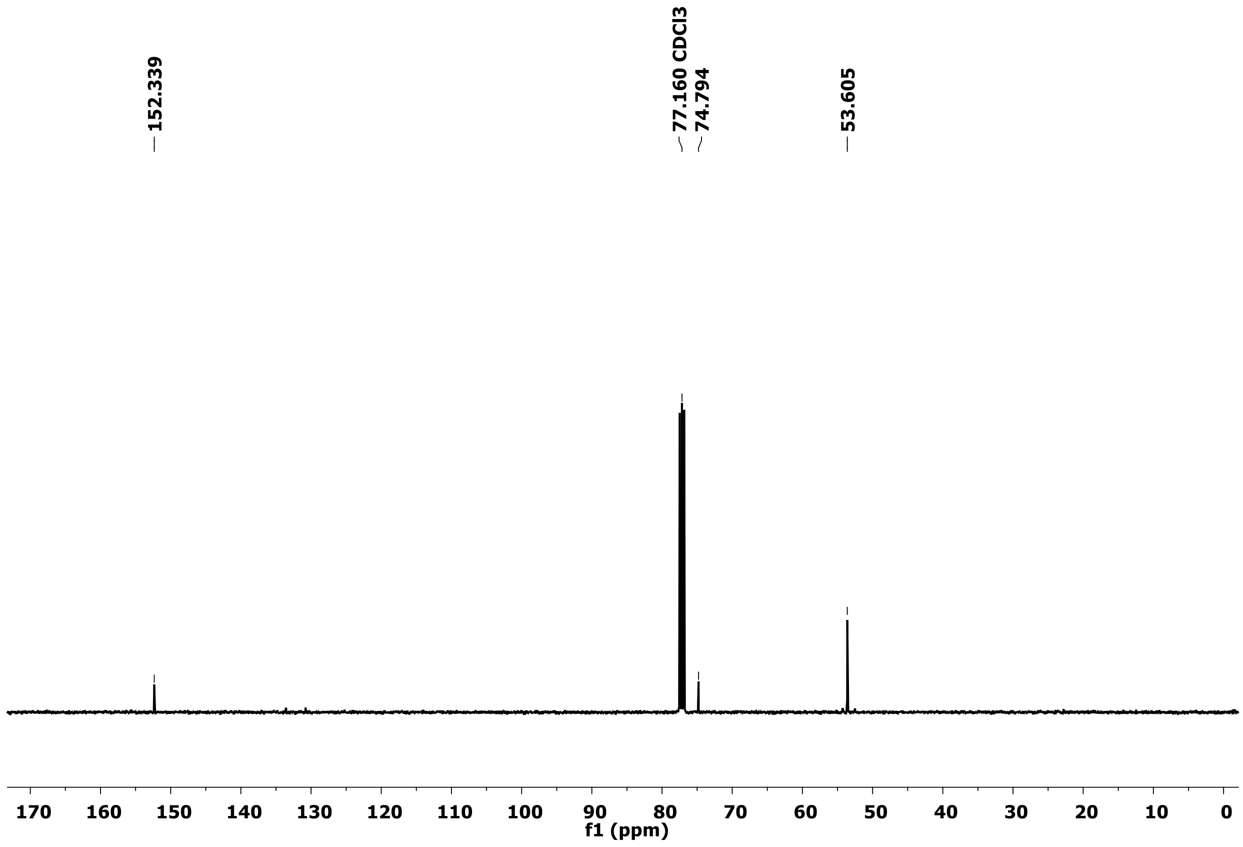

152.339
77.160 CDCl3
74.794
53.605
170 160 150 140 130 120 110 100 90 80 70 60 50 40 30 20 10 0
f1 (ppm)

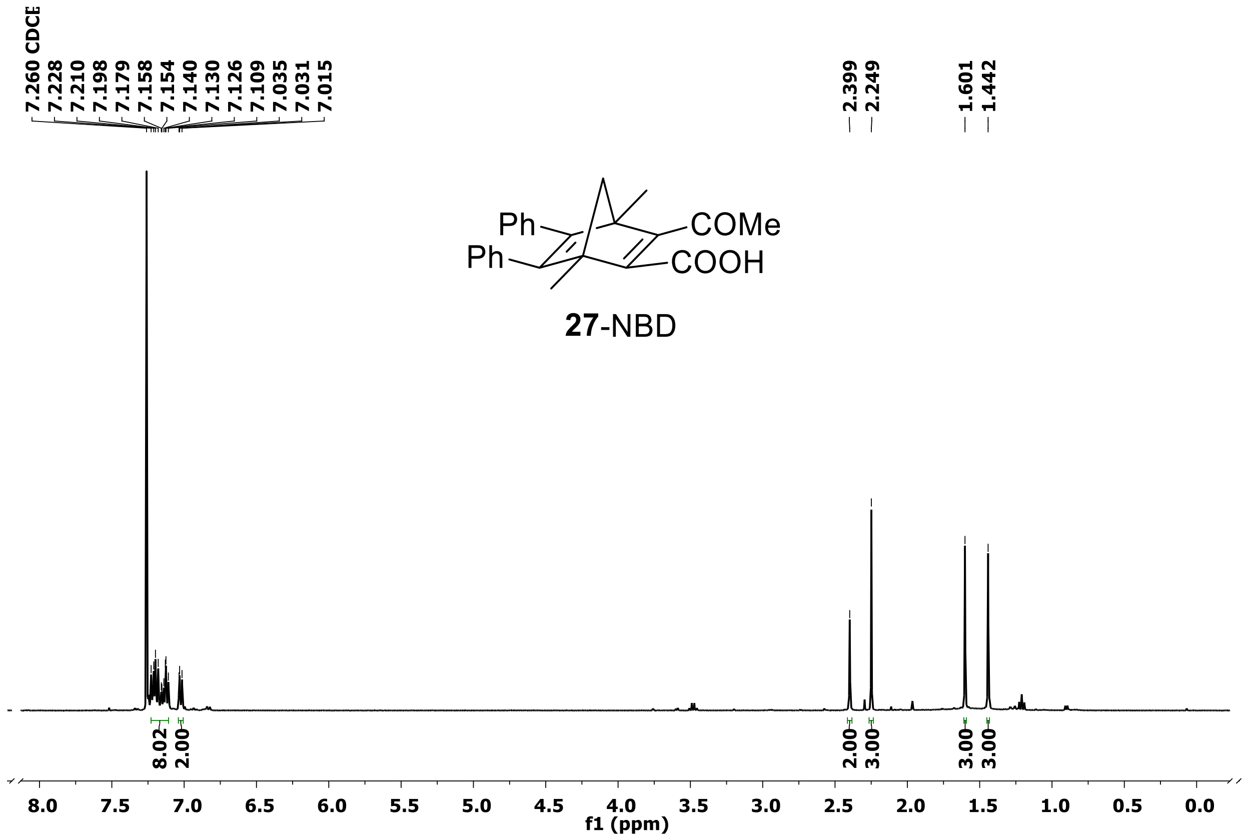

7.260 CDCl3
7.228
7.210
7.198
7.179
7.158
7.154
7.140
7.130
7.126
7.109
7.035
7.031
7.015
2.399
2.249
1.601
1.442
Ph
Ph
COMe
COOH
27-NBD
8.02
2.00
2.00
3.00
3.00
3.00
8.0 7.5 7.0 6.5 6.0 5.5 5.0 4.5 4.0 3.5 3.0 2.5 2.0 1.5 1.0 0.5 0.0
f1 (ppm)

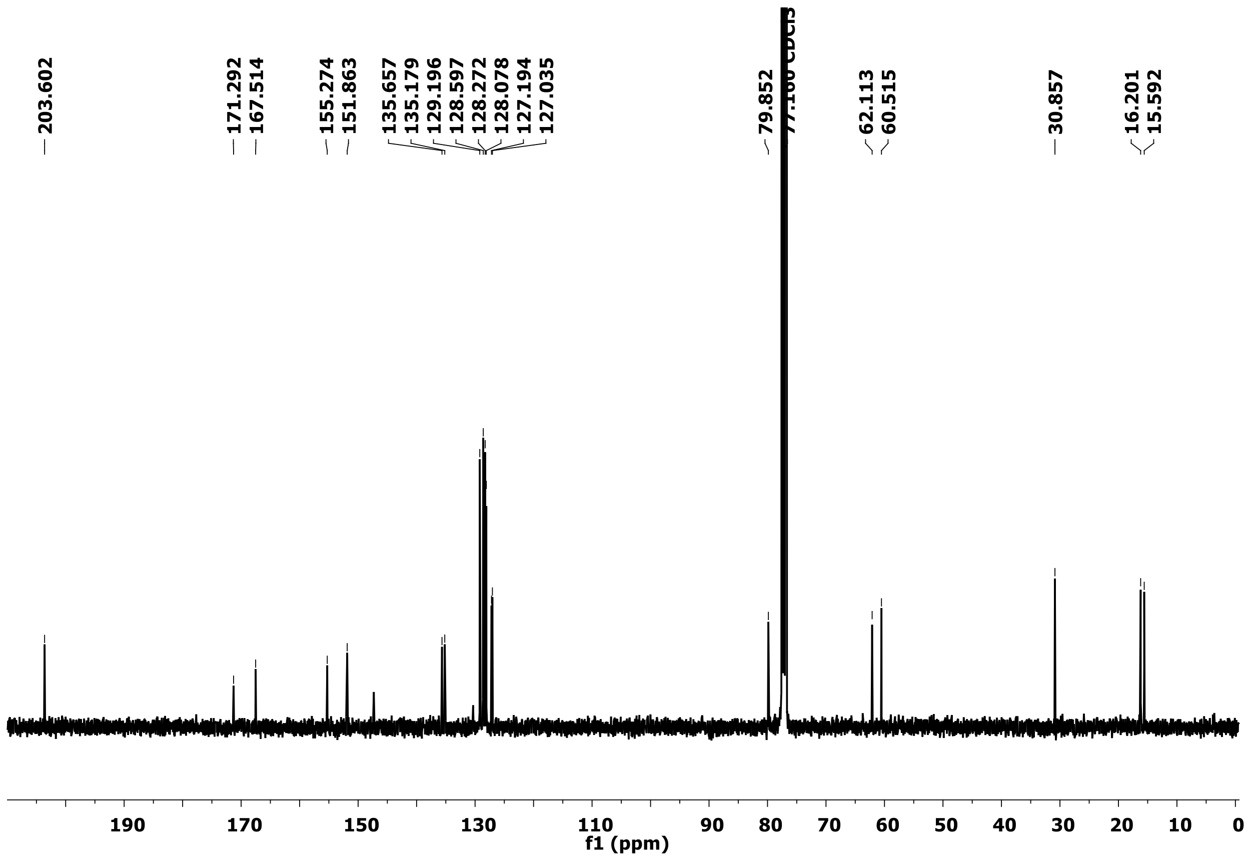

203.602
171.292
167.514
155.274
151.863
135.657
135.179
129.196
128.597
128.272
128.078
127.194
127.035
79.852
62.113
60.515
30.857
16.201
15.592
190 170 150 130 110 90 80 70 60 50 40 30 20 10 0
f1 (ppm)

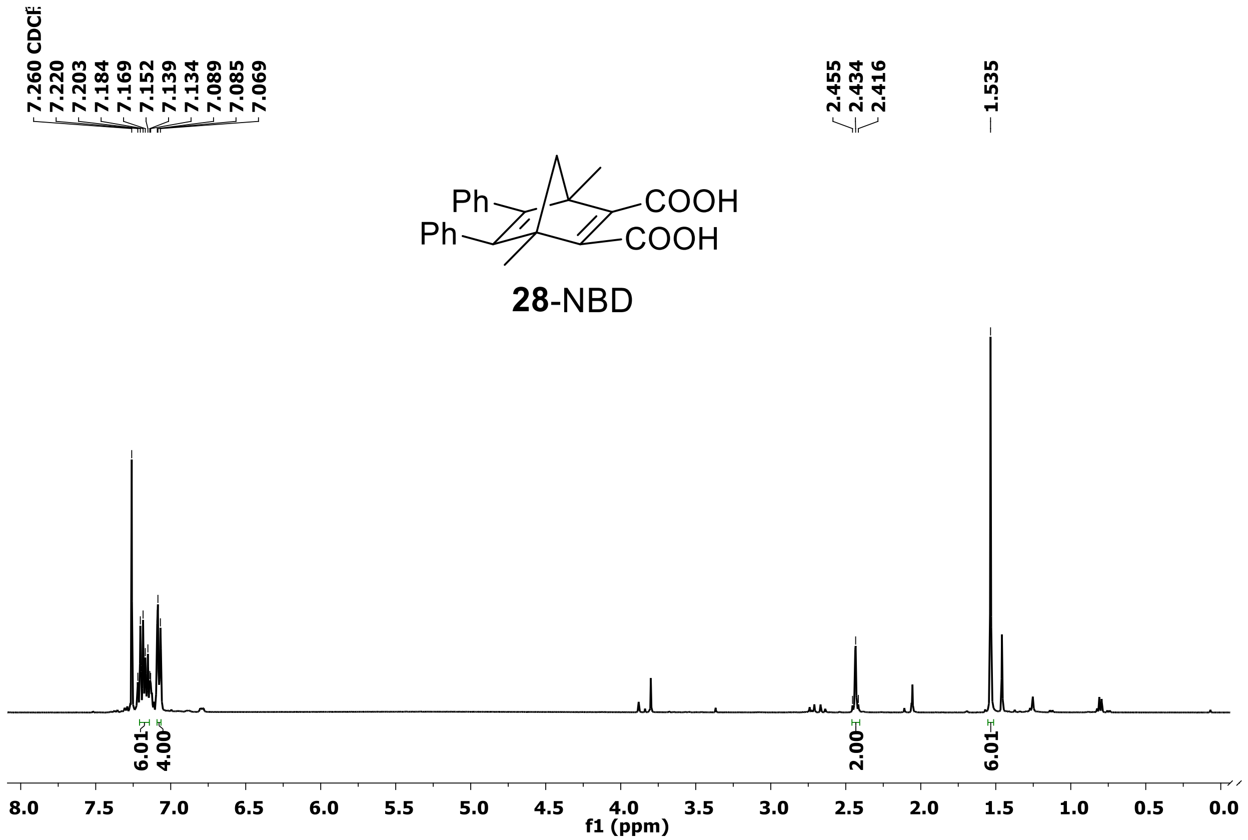
7.260 CDCl3
7.220
7.203
7.184
7.169
7.152
7.139
7.134
7.089
7.085
7.069
2.455
2.434
2.416
1.535
Ph
Ph
COOH
COOH
28-NBD
6.01
4.00
2.00
6.01
8.0 7.5 7.0 6.5 6.0 5.5 5.0 4.5 4.0 3.5 3.0 2.5 2.0 1.5 1.0 0.5 0.0
f1 (ppm)

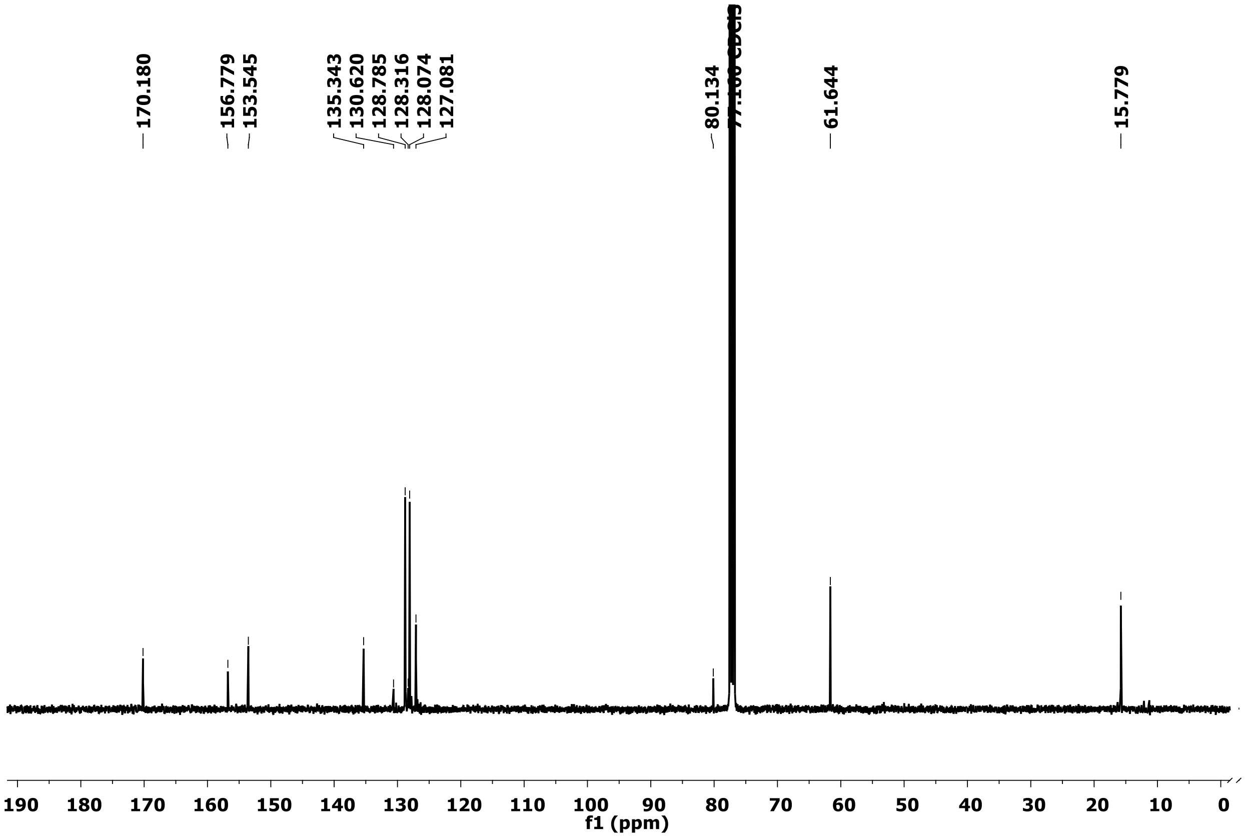
170.180
156.779
153.545
135.343
130.620
128.785
128.316
128.074
127.081
80.134
77.160 CDCl3
61.644
15.779
190 180 170 160 150 140 130 120 110 100 90 80 70 60 50 40 30 20 10 0
f1 (ppm)

8. Supporting Information References

[1] C. A. Parker, E. J. Bowen, *Proc. R. Soc. Lond. A* **1953**, *220*, 104-116.
[2] H. J. Kuhn, S. E. Braslavsky, R. Schmidt, *Pure Appl. Chem.* **2004**, *76*, 2105-2146.
[3] S. Goldstein, J. Rabani, *J. Photochem. Photobiol., A: Chem.* **2008**, *193*, 50-55.
[4] Z.-Z. Huang, S.-R. Sheng, X.-L. Liu, S.-Y. Lin, R.-S. Huang, *J. Chin. Chem. Soc.* **2006**, *53*, 991-994.
[5] K.-i. Hirao, A. Yamashita, A. Ando, T. Hamada, O. Yonemitsu, *J. Chem. Soc., Perkin Trans. 1* **1988**, 2913-2916.
[6] J. Liu, H. Li, C. Zheng, S. Lu, X. Guo, X. Yin, R. Na, B. Yu, M. Wang, *Molecules* **2017**, *22*, 364.
[7] L. L. Rodrigues, A. S. Micallef, M. C. Pfrunder, V. X. Truong, J. C. McMurtrie, T. R. Dargaville, A. S. Goldmann, F. Feist, C. Barner-Kowollik, *J. Am. Chem. Soc.* **2021**, *143*, 7292-7297.
[8] A. C. Gomes, A. J. Demuner, E. S. Alvarenga, J. P. Gondim, A. R. Fonseca, D. S. Buonicontro, E. J. Pilau, E. Silva, *J. Braz. Chem. Soc.* **2020**, *31*, 1805-1814.
[9] Schrödinger Release 2021-4: Maestro and MacroModel, Schrödinger, LLC, New York, NY, **2021**.
[10] M. J. Frisch, G. W. Trucks, H. B. Schlegel, G. E. Scuseria, M. A. Robb, J. R. Cheeseman, G. Scalmani, V. Barone, G. A. Petersson, H. Nakatsuji, X. Li, M. Caricato, A. V. Marenich, J. Bloino, B. G. Janesko, R. Gomperts, B. Mennucci, H. P. Hratchian, J. V. Ortiz, A. F. Izmaylov, J. L. Sonnenberg, D. Williams-Young, F. Ding, F. Lipparini, F. Egidi, J. Goings, B. Peng, A. Petrone, T. Henderson, D. Ranasinghe, V. G. Zakrzewski, J. Gao, N. Rega, G. Zheng, W. Liang, M. Hada, M. Ehara, K. Toyota, R. Fukuda, J. Hasegawa, M. Ishida, T. Nakajima, Y. Honda, O. Kitao, H. Nakai, T. Vreven, K. Throssell, J. A. Montgomery, Jr.; , J. E. Peralta, F. Ogliaro, M. J. Bearpark, J. J. Heyd, E. N. Brothers, K. N. Kudin, V. N. Staroverov, T. A. Keith, R. Kobayashi, J. Normand, K. Raghavachari, A. P. Rendell, J. C. Burant, S. S. Iyengar, J. Tomasi, M. Cossi, J. M. Millam, M. Klene, C. Adamo, R. Cammi, J. W. Ochterski, R. L. Martin, K. Morokuma, O. Farkas, J. B. Foresman, D. J. Fox, Gaussian, Inc., Wallingford CT, **2016**.
[11] C. Lu, C. Wu, D. Ghoreishi, W. Chen, L. Wang, W. Damm, G. A. Ross, M. K. Dahlgren, E. Russell, C. D. Von Bargen, R. Abel, R. A. Friesner, E. D. Harder, *J. Chem. Theory Comput.* **2021**, *17*, 4291-4300.
[12] A. D. Becke, *Phys. Rev. A* **1988**, *38*, 3098-3100.
[13] C. Lee, W. Yang, R. G. Parr, *Phys. Rev. B* **1988**, *37*, 785-789.
[14] S. Grimme, S. Ehrlich, L. Goerigk, *J. Comp. Chem.* **2011**, *32*, 1456-1465.
[15] F. Weigend, R. Ahlrichs, *Phys. Chem. Chem. Phys.* **2005**, *7*, 3297-3305.
[16] F. Weigend, *Phys. Chem. Chem. Phys.* **2006**, *8*, 1057-1065.
[17] A. V. Marenich, C. J. Cramer, D. G. Truhlar, *J. Phys. Chem. B* **2009**, *113*, 6378-6396.
